# Diastereoselective synthesis of new O-alkylated and C-branched inositols and their corresponding fluoro analogues

**DOI:** 10.3762/bjoc.12.39

**Published:** 2016-02-25

**Authors:** Charlotte Collet, Françoise Chrétien, Yves Chapleur, Sandrine Lamandé-Langle

**Affiliations:** 1 Université de Lorraine, Vandoeuvre-les-Nancy F-54500, France; 2 CNRS, UMR 7565, Vandoeuvre-les-Nancy F-54506, France

**Keywords:** C-branched, diastereoselective, fluoroinositols, inositols, O-alkylated

## Abstract

Efficient routes were developed for the diastereoselective synthesis of new O-alkylated and C-branched inositols and their corresponding fluoro analogues. The key steps of the synthesis were the easy accessibility of different types of arms in term of configuration (*myo* and *scyllo*), the linking method and length, which could modulate the biological properties. These inositol derivatives, bearing an arm terminated either with a hydroxy group or a fluorine atom, could be interesting candidates for diastereoisomeric intermediates and biological evaluations, especially for PET imaging experiments.

## Introduction

Inositol is a trivial name used to describe cyclohexanehexol compounds. Nine stereoisomers exist differing in the relative orientation of the hydroxy groups, the most naturally abounding and biologically important is *myo*-inositol [1–2]. The chemistry of inositols has been the theme of several investigations in the past few years [3–4]. Indeed inositols and their phosphorylated or glycosylated [5] derivatives are involved in various biological processes [6–19]. In recent years, studies have shown that inositol derivatives interact with the β-amyloid protein (Aβ), characteristic aggregation in Alzheimer disease (AD), and more specifically Aβ42 peptide allowing its conformational change and its stabilization into small, nonfibrillar complexes, less toxic for neuronal cells [20–23]. The most efficient representative, *scyllo*-inositol, was shown to be able to reduce plaque burden and to improve the performance on memory tasks in animal models of Alzheimer disease [24–29]. *Scyllo*-inositol was recently used in human clinical trials for the treatment of patients suffering from AD [30–31].

McLaurin has also demonstrated that the corresponding fluoroinositol (1-fluoro-1-deoxy-*scyllo*-inositol) was also a good candidate to limit this aggregation. However, in vivo studies in animal models of Alzheimer disease with 1-[^18^F]fluoro-1-deoxy-*scyllo*-inositol used as radiotracer for positron emission tomography (PET) have shown that this compound crosses the blood brain barrier (BBB) with difficulty [32]. The same PET imaging studies using the diastereoisomeric compound of *myo* configuration (2-[^18^F]fluoro-2-deoxy-*myo*-inositol), did not get better results [33]. On the other hand, these radiotracers have shown interesting features in the diagnosis of breast cancer with this imaging technique [33–34].

Considering the synthetic and biological importance of inositol analogues, the design of new inositol derivatives to study their biological properties and to understand their binding mechanisms with molecular targets, for instance through radiolabeled compounds, seems judicious.

**Figure 1:**
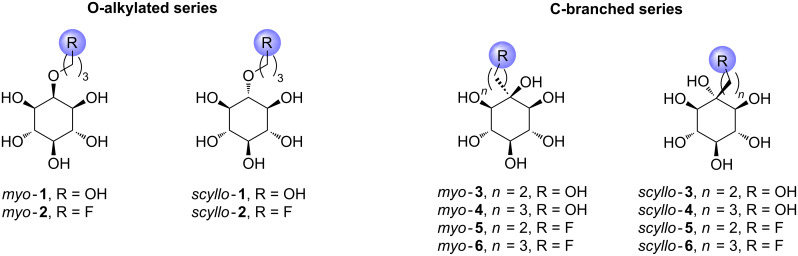
Structures of targeted synthetic inositol derivatives.

Thus we planned to study new synthetic inositols of *myo*- and *scyllo*-configuration equipped with different carbon chains linked either on one hydroxy group, i.e., O-alkylated, or C-branched derivatives retaining the six hydroxy groups of the inositol (Figure 1). These new inositol members could represent attractive tools for the development of further inositol analogues. Indeed, this arm could improve some properties as the lipophilicity for example and should allow the easy anchoring of various groups or molecules, such as carbohydrates for example.

Two arm lengths of two or three carbons were envisioned, on the C2 for the *myo*-series and on the C1 for the *scyllo*-series, ending with a hydroxy group or a fluorine atom. Indeed the addition of fluorine could improve chemical properties including lipophilicity, brain penetration, enhanced binding interaction and metabolic stability [35–40]. Moreover fluorinated inositols could be useful for PET imaging applications.

## Results and Discussion

Our strategy to prepare new alkylated inositol compounds and their corresponding fluoro analogues was based on the use of the known benzylated *myo*-2-inosose **7** [41] that can be easily obtained at large scale from commercially available *myo*-inositol. The introduction of the arm into the inositol backbone through an O–C linker (O-alkylated) or a C–C linker (C-branched) to afford diastereoselectively *myo-* and *scyllo-*derivatives was the key step. In the case of the O-alkylated inositols a three carbon arm was envisioned whereas for the C-branched derivatives two and three carbon atom arms were used. For the fluorinated inositols, we developed a synthetic route with easily removing protecting groups, as acetates, suitable for PET imaging application [42].

### Synthesis of O-alkylated *myo-* and *scyllo-*inositol derivatives bearing a hydroxylated arm

**Scheme 1:**
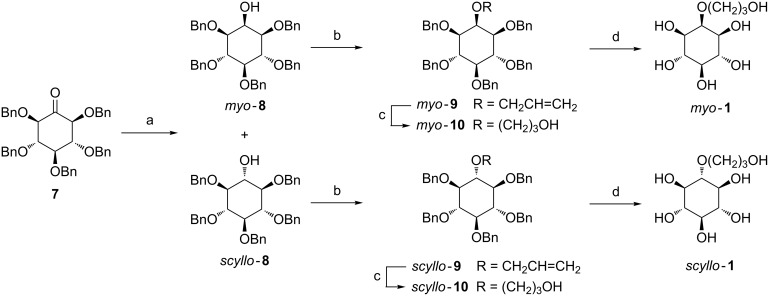
Synthesis of O-alkylated inositol derivatives **1**. Reagents and conditions: a) NaBH_4_, iPrOH, rt, 2 h, 58 and 30%; b) allyl bromide, NaH, 70 °C, 2 h, 80–82%; c) BH_3_, THF, rt, 6 h then H_2_O_2_, NaOH, rt, 12 h, 75–78%; d) Pd(OH)_2_, MeOH/CH_2_Cl_2_/H_2_O 10:10:1 (v/v/v), 45 psi, 18 h, quantitative.

The synthesis of the targeted hydroxylated O-alkylated inositols *myo***-1** and *scyllo***-1** (Scheme 1, see Supporting Information File 1 for full experimental data) started with a sodium borohydride reduction of the benzylated *myo*-2-inosose **7** in isopropyl alcohol at room temperature to give a mixture of the *myo-* and *scyllo*-inositol diastereoisomers *myo***-8** and *scyllo***-8**. The mixture was separated by column chromatography to afford the pure *myo*- and *scyllo*-inositol derivatives in 58% and 30% yields, respectively. The selectivity of this reduction was lower compared to previously published results [43]. A diastereoisomeric ratio of 4:1 *myo***-8**/*scyllo***-8** was estimated by ^1^H NMR when the same reduction was done in a dichloromethane/methanol mixture. This selectivity can be explained by a chelation of the sodium ion with the two OBn adjoining the ketone, which hindered the upper face. For our strategy having a moderate selectivity is of great advantage to continue the synthesis with both isolated diastereoisomers *myo***-8** and *scyllo***-8**. Next, the ether link was created by the reaction of alcohols *myo***-8** and *scyllo***-8** with allyl bromide under classical conditions in the presence of NaH at reflux [44] to afford *myo***-9** and *scyllo***-9** in good yield of 80% and 82%, respectively. The hydroxylated arm was obtained by hydroboration using BH_3_ on the unsaturated compounds *myo***-9** and *scyllo***-9** followed by an oxidation with H_2_O_2_/NaOH allowing the access to *myo***-10** and *scyllo***-10** in 78% and 75% yield, respectively. The O-alkylated inositol derivatives *myo***-1** and *scyllo***-1** were obtained quantitatively by removing of the benzyl protecting groups of compounds *myo***-10** and *scyllo***-10** by hydrogenation under pressure [45] using palladium hydroxide as catalyst.

### Synthesis of O-alkylated *myo-* and *scyllo-*inositol derivatives bearing a fluorinated arm

**Scheme 2:**
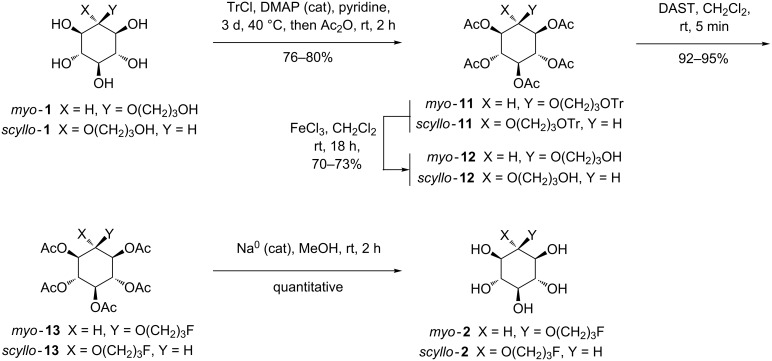
Synthesis of O-alkylated fluorinated inositol derivatives **2**.

The fluoro analogues of *myo***-1** and *scyllo***-1** were then prepared through a synthetic strategy that involves easily removable acetates on the inositol ring (Scheme 2, see Supporting Information File 1 for full experimental data).

The compounds *myo***-11** and *scyllo***-11** were obtained in 80% and 76% yield, respectively in a one pot reaction by the treatment of *myo***-1** and *scyllo***-1** with triphenylmethyl chloride in pyridine and a catalytic amount of DMAP at 40 °C for three days followed by the addition of acetic anhydride [46]. The trityl group in *myo***-11** and *scyllo***-11** was cleaved without acetyl migration using FeCl_3_ [47] to afford *myo***-12** and *scyllo***-12** in good yields. The primary hydroxy groups can be easily converted into fluorine by action of DAST in dichloromethane in less than 5 minutes to give *myo***-13** and *scyllo***-13** in 95 and 92% yield, respectively [48]. All protecting groups of *myo***-13** and *scyllo***-13** were removed by the treatment with Na^0^ in MeOH [49], which gave quantitatively the fully deprotected fluorinated O-alkylated inositols *myo***-2** and *scyllo***-2**.

### Synthesis of C-branched *myo-* and *scyllo-*inositol derivatives bearing a hydroxylated arm

**Scheme 3:**
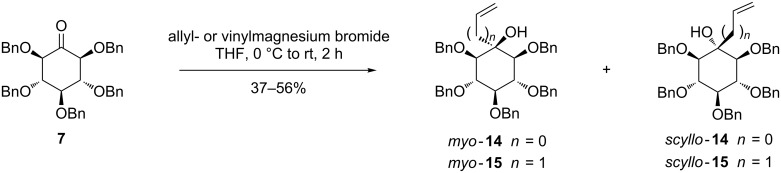
Synthesis of C-alkenylated inositol intermediates.

The synthesis of C-branched inositols was investigated using two different arm lengths, with either two or three carbon atoms. The introduction of these substituents was performed by a Grignard reaction in THF with vinyl- or allylmagnesium bromide on benzylated *myo*-2-inosose **7** (Scheme 3, see Supporting Information File 1 for full experimental data). The reaction gave in both cases diastereoisomeric *myo-* and *scyllo-*inositols, in a 60:40 proportion, respectively. The *myo*-inositol diastereoisomers were obtained in a larger proportion, due to the favored equatorial attack as compared to the axial one. Fortunately, this poor selectivity was associated with an easy separation by column chromatography giving *myo*-**14** and *scyllo*-**14** bearing a two carbon chain in 56% and 37% yield, respectively. The corresponding compounds *myo*-**15** and *scyllo*-**15** having an allyl substituent were obtained in 53% and 38% yields, respectively.

**Figure 2:**
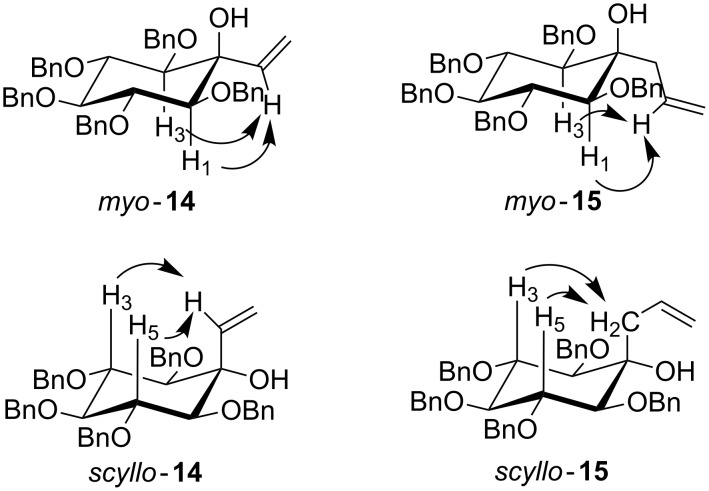
nOe correlations for C-alkenylated inositol intermediates.

The configuration of the diastereoisomers was determined with ^1^H and nOe NMR experiments (Figure 2). For compounds *myo*-**14** and *myo*-**15** correlations between H1/H3 and the alkenyl proton of the arm were observed confirming the equatorial orientation of the substituent and therefore the *myo*-inositol configuration. The same experiments were performed with compounds *scyllo***-14** and *scyllo*-**15** and showed correlations between H3/H5 and the hydrogen on the vinylic or allylic arm respectively fully in agreement with a *scyllo*-inositol configuration.

**Scheme 4:**
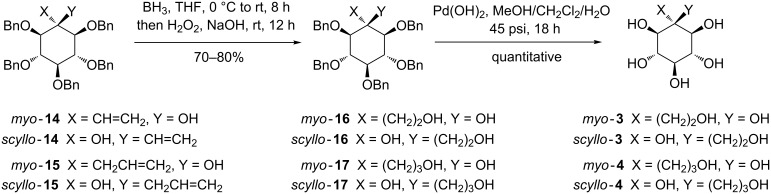
Synthesis of C-branched inositol derivatives **3** and **4**.

Having the C-alkenylated inositols in hand, the synthesis of C-branched inositol compounds with hydroxylated two or three carbon atom arms could be achieved (Scheme 4, see Supporting Information File 1 for full experimental data).

Unsaturated C-branched inositols **14** and **15** lead to the corresponding hydroxylated compounds **16** and **17**, after hydroboration using BH_3_ in THF at 0 °C followed by conventional oxidation using H_2_O_2_/NaOH, in 70–80% yield. Subsequent catalytic hydrogenation under pressure with Pd(OH)_2_ as the catalyst [45] allowed to obtaining quantitatively the fully hydroxylated inositols *myo*-**3**, *scyllo***-3**, *myo***-4** and *scyllo***-4** as a new hydroxylated inositol family.

### Synthesis of C-branched *myo-* and *scyllo-*inositol derivatives bearing a fluorinated two carbons arm

**Scheme 5:**
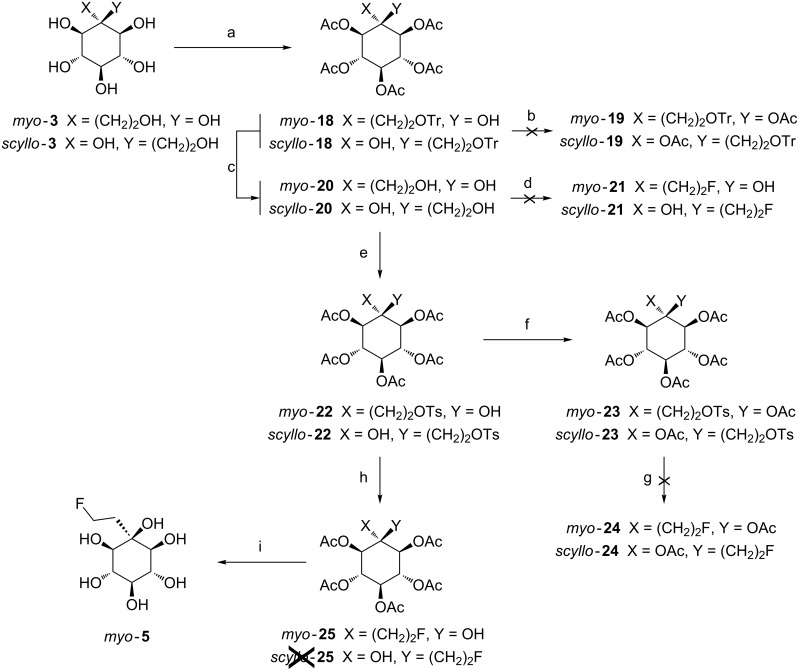
Synthesis of C-branched fluorinated inositol derivatives **5**. Reagents and conditions: a) TrCl, DMAP (cat), pyridine, 3 days, 70 °C, then Ac_2_O, rt, 2 h, 70–74%; b) isopropenyl acetate, *p*-TSA, 80 °C, 2 h; c) FeCl_3_, CH_2_Cl_2_, rt, 18 h or 15 min, 45–72%; d) DAST, CH_2_Cl_2_, rt; e) TsCl, Et_3_N, CH_2_Cl_2_, rt, 3 h, 70–71%; f) isopropenyl acetate, *p*-TSA, 80 °C, 2 h, 78–99%; g) KF, 18-crown-6, CH_3_CN or TBAF, CH_3_CN or KHF_2_, 18-crown-6, CH_3_CN; h) KF; 18-crown-6; CH_3_CN, rt, 2 h, 70%; i) Na^0^ (cat), MeOH, rt, 2 h, quantitative.

For the synthesis of fluorinated C-branched inositols with a two carbon atoms arm, a similar strategy as developed for the O-alkylated inositols was chosen to access the fluorinated compounds bearing easily removable acetyl protecting groups (Scheme 5, see Supporting Information File 1 for full experimental data). Starting from the fully hydroxylated inositols *myo***-3** and *scyllo***-3**, the first step was the protection of the primary hydroxy group with trityl chloride, followed by peracetylation with acetic anhydride in a one pot reaction [46]. *Myo***-18** and *scyllo***-18** were obtained in 74 and 70% yield, respectively. It is worthy to note that the tertiary hydroxy group in *myo***-18** and *scyllo***-18** remained unprotected under these conditions. The literature data are rather contradictory concerning the acetylation of this tertiary alcohol using acetic anhydride [50–51]. With regard to a PET application, the protection of this hydroxy group is of paramount importance since the free hydroxy group could impair the incorporation of the radioactive fluorine. Therefor several methods of acetylation were carried out on *myo***-14**, which was used as model. The best results for the acetylation of the tertiary alcohol in good yield was obtained with the transesterification method using isopropenyl acetate as acylating agent and *p*-toluenesulfonic acid as catalyst at 80 °C for 2 h [52]. Unfortunately, when these reaction conditions were applied on the tritylated compounds *myo***-18** and *scyllo***-18** we were unable to obtain the fully protected products *myo***-19** and *scyllo***-19**; we only got several compounds without trityl group due to the acidic reaction conditions. So we decided to cleanly remove this troublesome group using FeCl_3_ in dichloromethane for 18 h at room temperature [47]. Under these conditions the compound *scyllo***-20** was obtained in a good yield (72%) while *myo***-20** was obtained only in a poor yield (15%) due to the formation of byproducts proceeding from cyclization and/or acetate migration. By reducing the reaction time to 15 minutes the byproducts formation could be lowered to 10% and *myo***-2** was obtained in 45% isolated yield. It is worthy to note that 41% of the starting material was recovered.

At this stage, the fluorination was tested using DAST [48] on *myo***-20** and *scyllo***-20**. Unfortunately, the desired fluorinated products *myo***-21** and *scyllo***-21** were not obtained; instead elimination reaction and the formation of unidentified products were observed. Hence, tosylation was next performed, in order to allow the nucleophilic substitution with fluoride at a later stage. For this, compounds *myo*-**20** and *scyllo***-20** were reacted with tosyl chloride in the presence of a catalytic amount of triethylamine at room temperature for 3 h to give tosylated *myo***-22** and *scyllo***-22** in 70 and 71% yield, respectively. Next, acetylation of the tertiary alcohol was performed before fluorination. The utilization of isopropenyl acetate as solvent in the presence of *p*-TSA at 80 °C for 2 h [52] gave the corresponding acetylated compounds *myo***-23** and *scyllo***-23** in good to excellent yields. The access to fluorinated compounds by nucleophilic substitution was investigated starting from these fully acetylated inositols. The fluorination reactions using potassium fluoride, 18-crown-6 in acetonitrile at 70 °C [53], TBAF in acetonitrile at 60 °C for 2 h [54] or KHF_2_ and 18-crown-6 in acetonitrile for 2 h at 60 °C [55] were performed. However, ^19^F NMR analyses of the reaction mixtures did not show any fluorine incorporation. Various types of compounds resulting from the migration of the tertiary acetate to the primary position, elimination reaction or cyclization were identified depending on the experimental procedure.

From these results, the fluorination was finally investigated on *myo***-22** and *scyllo*-**22** derivatives, which were not acetylated on the tertiary position; in order to avoid the acyl migration. The treatment of *myo***-22** with KF, 18-crown-6 in acetonitrile [53] at room temperature (optimal conditions) furnished the fluorinated compound *myo***-25** in 70% yield. The ^19^F NMR spectrum showed only one signal at −217 ppm (triplet of triplet, *J* = 48 Hz, *J* = 28 Hz) establishing the formation of the expected fluorinated product. Unfortunately, in the case of the isomeric *scyllo***-22** whatever the conditions used, the desired fluoro compound *scyllo***-25** could not be obtained; three different cyclized byproducts were formed. Finally, Zemplen [49] deprotection was performed on compound *myo***-25** to afford *myo***-5** quantitatively.

### Synthesis of C-branched *myo-* and *scyllo-*inositol derivatives bearing a fluorinated three carbon atoms arm

**Scheme 6:**
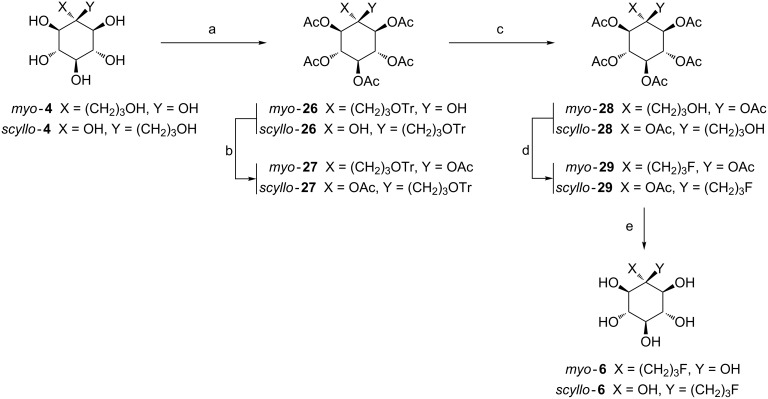
Synthesis of C-branched fluorinated inositol derivatives **6**. Reagents and conditions: a) TrCl, DMAP (cat), pyridine, 3 d, rt, then Ac_2_O, rt, 2 h, 67–70%; b) isopropenyl acetate, *p*-TSA, 80 °C, 2 h, 52–56%; c) FeCl_3_, CH_2_Cl_2_, rt, 18 h, 63–70%; d) DAST, CH_2_Cl_2_, rt, 5 min, 91–95%; e) Na^0^ (cat), MeOH, rt, 2 h, quantitative.

Having in hand *myo***-4** and *scyllo***-4**, the development of C-branched inositols fluorinated on the three carbon atoms arm was carried out by the same methodology as used for the C-branched two carbon atoms chain (Scheme 6, see Supporting Information File 1 for full experimental data).

The selective protection of the primary hydroxy group with trityl chloride, followed by peracetylation with acetic anhydride in one pot was carried out [46]. Compounds *myo***-26** and *scyllo***-26** were obtained in 67% and 70% yields, respectively. However, as already observed for the two carbon-atoms-arm-branched compounds, the tertiary hydroxy group remained unprotected. As explained above, the protection of this hydroxy group is mandatory for labeling with [^18^F]-fluoride with regard to a PET application. Therefore the acetylation of *myo***-26** and *scyllo***-26** using the transacetylization protocol [52] gave the fully acetylated products *myo***-27** and *scyllo***-27** in 56% and 52% isolated yields, respectively. The moderate yields could be explained by the presence of detritylated fully acetylated byproducts due to the acidic reaction conditions. Next, deprotection of the trityl group from *myo***-27** and *scyllo*-**27** was performed using FeCl_3_ in dry dichloromethane at room temperature overnight [47]. The hydroxylated products *myo***-28** and *scyllo***-28** were obtained in 63% and 70% yields, respectively. The fluorination of compounds **28** was carried out with DAST as fluorinating agent in dichloromethane at room temperature for 5 min [48] and the fluoro-compounds *myo***-29** and *scyllo***-29** were obtained in excellent yields of 95% and 91%, respectively. Finally, Zemplen deprotection [49] of the acetyl groups was carried out to furnish quantitatively the fully deprotected fluoro-inositols *myo***-6** and *scyllo***-6**. The obtained diastereoisomeric compounds *myo*-**6** and *scyllo*-**6** having a C–C linked three carbon-atoms arm represent a new class of inositols.

The above results demonstrate that the new O-alkylated and C-branched inositols with *myo*- and *scyllo*-configuration were available in a highly diastereoselective way. Different types of arm were easily introduced on the inositol ring. For the O-alkylated series, the hydroxylated (*myo***-1** and *scyllo***-1**) as well as fluorinated derivatives (*myo***-2** and *scyllo***-2**) were synthesized in a simple and efficient manner. Concerning the C-branched fluorinated inositols, the synthesis of compounds having a three carbon atoms arm (*myo***-6** and *scyllo***-6**) was easier than the corresponding compounds substituted with the two carbon atoms arm where only compound *myo***-5** could be obtained. Indeed with the chain being longer, the formation of byproducts originating from cyclization and migration, favored by the proximity of the inositol ring and preferential transition states, was avoided. The synthetic routes are convenient and could provide sufficient material for biological study.

## Conclusion

In conclusion, novel families of O-alkylated and C-branched inositols with a two or three carbon atoms arm and their corresponding fluoro analogues were synthesized in an efficient and diastereoselective manner. Noteworthy aspects of this methodology are the easy accessibility of different types of arms in term of configuration (*myo* and *scyllo*), linking method and length, which could modulate the biological properties. The series bearing an arm terminated by a hydroxy group could provide interesting candidates for biological evaluations but also as useful diastereoisomeric intermediates to anchor various groups such as carbohydrates, for example, opening up new avenues for the total synthesis of new inositol derivatives. The synthesized inositol derivatives with fluorinated arms could be more specifically interesting candidates for PET imaging experiments. Their potential use as radiotracers is under current investigation.

## Supporting Information

File 1 Experimental procedures, characterization data and copies of ^1^H, ^13^C, and ^19^F NMR spectra of all new compounds.

## References

[1] Posternak T (1962). The cyclitols.

[2] Anderson L, Pigman W, Horton D (1972). The Cyclitols. The Carbohydrates.

[3] Kılbaş B, Balci M (2011). Tetrahedron.

[4] Potter B V L, Lampe D (1995). Angew Chem, Int Ed Engl.

[5] McConville M J, Ferguson M A (1993). Biochem J.

[6] Bleasdale J E, Eichberg J, Hauser G (1985). Inositol and Phophoinositides.

[7] Billington D C (1993). The inositol Phosphates. Chemical Synthesis and Biological Significance.

[8] Dalko O P, Sinay P, Schmalz H G, Wirth T (2003). Organic Synthesis Highlights V.

[9] Michell R H, Drumond A H, Downes C P (1989). Inositol Lipids in Cell Signalling.

[10] Schedler D J A, Baker D C (2004). Carbohydr Res.

[11] Roussel F, Moitessier N, Hilly M, Chrétien F, Mauger J-P, Chapleur Y (2002). Bioorg Med Chem.

[12] Bennett M, Onnebo S M N, Azevedo C, Saiardi A (2006). Cell Mol Life Sci.

[13] Barker C J, Illies C, Gaboardi G C, Berggren P-O (2009). Cell Mol Life Sci.

[14] Roussel F, Hilly M, Chrétien F, Mauger J-P, Chapleur Y (1999). J Carbohydr Chem.

[15] Hilton J M, Plomann M, Ritter B, Modregger J, Freeman H N, Falck J R, Krishna U M, Tobin A B (2001). J Biol Chem.

[16] Tsui M M, York J D (2010). Adv Enzyme Regul.

[17] Vajanaphanich M, Schultz C, Rudolf M T, Wasserman M, Enyedi P, Craxton A, Shears S B, Tsien R Y, Barrett K E, Traynor-Kaplan A (1994). Nature.

[18] Shi Y, Azab A N, Thompson M N, Greenberg M L, Majumder A L, Biswas B B (2006). Biology of Inositol phosphates and phosphoinositides. Subcellular Biochemistry.

[19] Chen W, Deng Z, Chen K, Dou D, Song F, Li L, Xi Z (2015). Eur J Med Chem.

[20] McLaurin J, Golomb R, Jurewicz A, Antel J P, Fraser P E (2000). J Biol Chem.

[21] Nitz M, Fenili D, Darabie A A, Wu L, Cousins J E, McLaurin J (2008). FEBS J.

[22] Hawkes C A, Ng V, McLaurin J (2009). Drug Dev Res.

[23] Sun Y, Zhang G, Hawkes C A, Shaw J E, McLaurin J, Nitz M (2008). Bioorg Med Chem.

[24] McLaurin J, Kierstead M E, Brown M E, Hawkes C A, Lambermon M H, Phinney A L, Darabie A A, Cousins J E, French J E, Lan M F (2006). Nat Med.

[25] Townsend M, Cleary J P, Mehta T, Hofmeister J, Lesne S, O'Hare E, Walsh D M, Selkoe D J (2006). Ann Neurol.

[26] Choi J-K, Carreras I, Dedeoglu A, Jenkins B G (2010). Neuropharmacology.

[27] Aytan N, Choi J-K, Carreras I, Kowall N W, Jenkins B G, Dedeoglu A (2013). Exp Neurol.

[28] Fenili D, Brown M, Rappaport R, McLaurin J (2007). J Mol Med (Heidelberg, Ger).

[29] Fenili D, Keran M A, Mclaurin J, Martinez A (2010). scyllo-Inositol: A Potential Therapeutic for Alzheimer’s Disease. Emerging Drugs and Targets for Alzheimer’s Disease, Vol. 2: Neuronal Plasticity, Neuronal Protection and Other Miscellaneous Strategies.

[30] Salloway S, Sperling R, Keren R, Porsteinsson A P, van Dyck C H, Tariot P N, Gilman S, Arnold D, Abushakra S, Hernandez C (2011). Neurology.

[31] Ma K, Thomason L A M, McLaurin J (2012). Adv Pharmacol (San Diego, CA, U S).

[32] Vasdev N, Chio J, van Oosten E M, Nitz M, McLaurin J, Vines D C, Houle S, Reilly R M, Wilson A A (2009). Chem Commun.

[33] Carroll L, Perumal M, Vasdev N, Robins E, Aboagye E O (2012). Bioorg Med Chem Lett.

[34] McLarty K, Moran M D, Scollard D A, Chan C, Sabha N, Mukherjee J, Guha A, McLaurin J, Nitz M, Houle S (2011). Nucl Med Biol.

[35] Hiyama T, Yamamoto H (2000). Organofluorine Compounds – Chemistry and Applications.

[36] Yokohama M (2000). Carbohydr Res.

[37] Toshima K (2000). Carbohydr Res.

[38] Dax K, Albert M, Ortner J, Paul B J (2000). Carbohydr Res.

[39] Plantier-Royon R, Portelle C (2000). Carbohydr Res.

[40] Hagmann W K (2008). J Med Chem.

[41] Lowe G, McPhee F (1991). J Chem Soc, Perkin Trans 1.

[42] Chapleur Y, Lamande-Langle S, Collet C, Chretien F (2013). Radiotracer, in particular for Alzheimer's disease. WO Patent.

[43] Jagdhane R C, Patil M T, Krishnaswamy S, Shashidhar M S (2013). Tetrahedron.

[44] Koto S, Hirooka M, Yoshida T, Takenaka K, Asai C, Nagamitsu T, Sakuma H, Sakurai M, Masuzawa S, Komiya M (2000). Bull Chem Soc Jpn.

[45] Vishwakarma R A, Vehring S, Mehta A, Sinha A, Pomorski T, Herrmann A, Menon A K (2005). Org Biomol Chem.

[46] Eguchi T, Sasaki S, Huang Z, Kakinuma K (2002). J Org Chem.

[47] Zhang F, Zhang W, Zhang Y, Curran D P, Liu G (2009). J Org Chem.

[48] Kováč P (1986). Carbohydr Res.

[49] Zemplén G, Pacsu E (1929). Chem Ber.

[50] Kiely D E, Cantrell C E (1972). Carbohydr Res.

[51] Sarmah M P, Shashidhar M S, Sureshan K M, Gonnade R G, Bhadbhade M M (2005). Tetrahedron.

[52] Gula M J, Vitale D E, Dostal J M, Trometer J D, Spencer T A (1988). J Am Chem Soc.

[53] Vogel P, Figueira S, Muthukrishnan S, Mack J (2009). Tetrahedron Lett.

[54] Kim K-Y, Kim B C, Lee H B, Shin H (2008). J Org Chem.

[55] Yang S S, Min J M, Beattie T R (1988). Synth Commun.

