# Supplementary material for: Diastereoselective synthesis of new O-alkylated and C-branched inositols and their corresponding fluoro analogues
Source: Beilstein J Org Chem. 2016 Feb 25;12:353–61. doi: 10.3762/bjoc.12.39 (PMC4778503; doi:10.3762/bjoc.12.39)
Supplement: File 1 — Experimental procedures, characterization data and copies of 1H, 13C, and 19F NMR spectra of all new compounds. [file Beilstein_J_Org_Chem-12-353-s001.pdf]

Supporting Information File 1

for

Diastereoselective synthesis of new O-alkylated and C-branched inositols and their corresponding fluoro analogues

Charlotte Collet[*1,2,§], Françoise Chrétien[1,2], Yves Chapleur[1,2] and Sandrine Lamandé-Langle[*1,2]

Address: [1]Université de Lorraine, Vandoeuvre-les-Nancy F-54500 – France and [2]CNRS, UMR 7565, Vandoeuvre-les-Nancy F-54506 - France

[§]Present address: Nancyclotep, Plateforme d'imagerie moléculaire, Vandoeuvre-les-Nancy F-54500 - France

Email: Charlotte Collet - c.collet@nancyclotep.com; Sandrine Lamandé-Langle - sandrine.langle@univ-lorraine.fr

[*]Corresponding author

Experimental procedures, characterization data of new compounds and ^{1}H, ^{13}C, ^{19}F NMR spectra of synthesized compounds

Contents

General experimental details S3

Experimental procedures and physico-chemical characterization S3

^{1}H, ^{13}C, ^{19}F NMR spectra of compounds *myo*-**1** to *myo*-**6** and *scyllo*-**1** to *scyllo*-**6** S31

^{1}H, ^{13}C spectra of compounds *myo*-**9** to *myo*-**12** and *scyllo*-**9** to *scyllo*-**12** S56

^{1}H, ^{13}C, ^{19}F NMR spectra of compounds *myo*-**13** and *scyllo*-**13** S70

^{1}H, ^{13}C NMR spectrum of compounds *myo*-**14**, *myo*-**15**, *scyllo*-**14** and *scyllo*-**15** S76

^{1}H, ^{13}C spectra of compounds *myo*-**16**, *myo*-**17**, *scyllo*-**16** and *scyllo*-**17** S84

^{1}H, ^{13}C spectra of compounds *myo*-**18**, *myo*-**20**, *scyllo*-**18** and *scyllo*-**20** S90

^{1}H, ^{13}C spectra of compounds *myo*-**22**, *myo*-**23**, *scyllo*-**22** and *scyllo*-**23** S96

^{1}H, ^{13}C ^{19}F NMR spectra of compound *myo*-**25** S104

^{1}H, ^{13}C spectra of compounds *myo*-**26**, *myo*-**27**, *scyllo*-**26** and *scyllo*-**27** S107

^{1}H, ^{13}C spectra of compounds *myo*-**28** and *scyllo*-**28** S115

^{1}H, ^{13}C, ^{19}F NMR spectra of compounds *myo*-**29** and *scyllo*-**29** S119

General experimental details

Solvents and liquid reagents were purified and dried according to recommended procedures. TLC analyses were performed using standard procedures on Kieselgel 60 F_{254} plates (Merck). Compounds were visualized using UV light (254 nm) or a methanolic solution of sulfuric acid and charring. Column chromatography and flash chromatography were performed on silica gel SI 60 (63–200 μm) or (40–63 μM) (Merck), respectively. Melting points were determined with a Tottoli apparatus and are uncorrected. FTIR spectra were recorded on a Perkin-Elmer Spectrum 100 by using NaCl windows (film) or KBr pellets (cm^{-1}). ^{1}H, ^{13}C NMR and ^{19}F spectra were recorded on a Bruker spectrometer DPX250 (250 MHz, 62.9 MHz and 235 MHz, respectively) or a DRX400 (400 MHz and 100.6 MHz, respectively). For complete assignment of ^{1}H and ^{13}C signals, two-dimensional ^{1}H, ^{1}H COSY and ^{1}H, ^{13}C correlation spectra were recorded. Chemical shifts (δ) are given in ppm relative to the solvent residual peak. The following abbreviations are used for multiplicity of NMR signals: s = singlet, d = doublet, t = triplet, q = quadruplet, m = multiplet, b = broad signal and app = apparent multiplicity. The given *J* values refer to apparent multiplicities and do not represent the true coupling constants. In the NMR description the protons and carbons atom of the ethyl arm are numbered 7 and 8, those of the propyl arm are numbered 7, 8 and 9. High resolution mass spectrometry (HRMS) was carried out by electrospray ionization (ESI) on a Bruker MicrOTOF$_{Q}$ apparatus.

Experimental procedures and physico-chemical characterization

2-*O*-(3-Hydroxypropyl)-*myo*-inositol (*myo*-1)

Compound *myo*-**10** (2 g, 2.9 mmol) and $Pd(OH)_2$ (500 mg, 20 mol %) were dissolved in MeOH (20 mL), CH_2Cl_2 (20 mL) and H_2O (2 mL). The reaction mixture was introduced in a Parr apparatus and stirred under a hydrogen atmosphere at 45 psi overnight. After completion of the reaction, the catalyst was filtered off through a pad of Celite®, washed with water and

the filtrate was concentrated under reduced pressure. Compound *myo*-**1** (700 mg, quantitative yield) was used without further purification. ^{1}H NMR (400 MHz, D_2O) δ 1.85 (qt, 2H, $J_{8,7}$ = $J_{8,9}$ = 6.0 Hz, H-8), 3.25 (app t, 1H, $J_{5,4}$ = $J_{5,6}$ = 9.5 Hz, H-5), 3.56 (dd, 2H, $J_{3,4}$ = $J_{1,6}$ = 9.5 Hz, $J_{3,2}$ = $J_{1,2}$ = 2.0 Hz, H-1 and H-3), 3.60 (app t, 2H, H-4 and H-6), 3.73 (t, 2H, H-9), 3.86 (m, 3H, H-7 and H-2); ^{13}C NMR (100.6 MHz, D_2O) δ 32.0 (C-8), 59.6 (C-9), 71.9 (C-7), 72.0 (C-3 and C-1), 73.1 (C-4 and C-6), 74.8 (C-5), 81.5 (C-2); IR (film, ν, cm^{-1}) 3400, 2901, 1049; HRMS ESI *m/z* : calculated for $C_9H_{18}NaO_7$ [M+Na]$^+$ 261.0945. Found: 261.0903.

1-*O*-(3-Hydroxypropyl)-*scyllo*-inositol (*scyllo*-1)

Compound *scyllo*-**10** (2 g, 2.9 mmol) and $Pd(OH)_2$ (500 mg, 20 mol %) were dissolved in MeOH (20 mL), CH_2Cl_2 (20 mL) and H_2O (2 mL). The reaction mixture was introduced in a Parr apparatus and stirred under hydrogen atmosphere at 45 psi overnight. After completion of the reaction, the catalyst was filtered off through a Celite® pad, washed with water and the filtrate was concentrated under reduced pressure. Compound *scyllo*-**1** (700 mg, quantitative yield) was used without further purification. ^{1}H NMR (400 MHz, D_2O) δ 1.92 (qt, 2H, $J_{7,8}$ = $J_{8,9}$ = 6.5 Hz, H-8), 3.27 (app t, 1H, $J_{1,2}$ = $J_{1,6}$ = 9.5 Hz, H-1), 3.36-3.50 (m, 5H, H-inositol), 3.78 (t, 2H, H-9), 3.95 (t, 2H H-7); ^{13}C NMR (100.6 MHz, D_2O) δ 32.2 (C-8), 59.4 (C-9), 70.6 (C-7), 73.6 (C-2 and C-6 or C-3 and C-5), 73.9 (C-4), 73.9 (C-2 and C-6 or C-3 and C-5), 82.8 (C-1); IR (KBr, ν, cm^{-1}) 3400, 2923, 1105, 996; HRMS ESI *m/z* : calculated for $C_9H_{18}NaO_7$ [M+Na]$^+$ 261.0945. Found: 261.0904.

2-*O*-(3-Fluoropropyl)-*myo*-inositol (*myo*-2)

To a solution of *myo*-**13** (100 mg, 0.22 mmol) in dry MeOH (10 mL) was added under argon at 0 °C, a catalytic amount of sodium. The reaction mixture was stirred for 2 h at room temperature, then neutralized by Amberlite IR-120. Water was added to solubilize the compound, then the solution was filtered and evaporated under reduced pressure to give *myo*-

2 (50 mg, quantitative) as an oil R_f 0.43 (9/1 CH_3CN/H_2O); ^1H NMR (400 MHz, D_2O) δ 1.99 (dqt, 2H, $J_{H,F}$ = 27.5 Hz, $J_{8,7}$ = $J_{8,9}$ = 6.0 Hz, H-8), 3.21 (app t, 1H, $J_{5,4}$ = $J_{5,6}$ = 9.5 Hz, H-5), 3.53 (dd, 2H, $J_{3,4}$ = $J_{1,6}$ = 9.5 Hz, $J_{3,2}$ = $J_{1,2}$ = 2.5 Hz, H-1 and H-3), 3.59 (app t, 2H, H-4 and H-6), 3.82 (app t, 1H, H-2), 3.85 (t, 2H, H-7), 4.61 (dt, 2H, $J_{H,F}$ = 47.0 Hz, $J_{8,9}$ = 6.0 Hz, H-9); ^{13}C NMR (100.6 MHz, D_2O) δ 30.8 (d, $J_{C,F}$ = 19.0 Hz, C-8), 70.4 (d, $J_{C,F}$ = 6.0 Hz, C-7), 71.9 (C-4 and C-6), 73.0 (C-1 and C-3), 74.9 (C-5), 81.4 (C-2), 82.9 (d, $J_{C,F}$ = 157.0 Hz, C-9); NMR (^{19}F, 235 MHz D_2O) δ -219.4 (tt, $J_{H,F}$ = 47.0 Hz, $J_{H,F}$ = 27.5 Hz); IR (film, ν, cm^{-1}) 3400, 2924, 999; HRMS ESI *m/z* : calculated for $C_9H_{17}FNaO_6$ $[M+Na]^+$ 263.0901. Found: 263.0913.

1-*O*-(3-Fluoropropyl)-*scyllo*-inositol (*scyllo*-2)

To a solution of *scyllo*-**13** (100 mg, 0.22 mmol) in dry MeOH (10 mL) was added under argon at 0 °C, a catalytic amount of sodium. The reaction mixture was stirred for 2 h at room temperature, then neutralized by Amberlite IR-120. Water was added to solubilize the compound, then the solution was filtered and evaporated under reduced pressure to give *scyllo*-**2** (50 mg, quantitative) as an oil R_f 0.43 (9/1 CH_3CN/H_2O); ^1H NMR (400 MHz, D_2O) δ 2.01 (dqt, 2H, $J_{H,F}$ = 26.0 Hz, $J_{7,8}$ = $J_{8,9}$ = 6.0 Hz, H-8), 3.27 (app t, 1H, $J_{1,2}$ = $J_{1,6}$ = 9.0 Hz, H-1), 3.32-3.49 (m, 5H, H-inositol), 3.97 (t, 2H, H-7), 4.69 (dt, 2H, $J_{H,F}$ = 47.0 Hz, H-9); ^{13}C NMR (100.6 MHz, D_2O) δ 30.8 (d, $J_{C,F}$ = 17.0 Hz, C-8), 69.5 (d, $J_{C,F}$ = 5.5 Hz, C-7), 73.6 (C-2 and C-6 or C3 and C-5), 73.8 (C-4), 73.9 (C-2 and C-6 or C3 and C-5), 82.8 (d, $J_{C,F}$ = 157.0 Hz, C-9), 82.8 (C-1); NMR (^{19}F, 235 MHz D_2O) δ -219.6 (tt, $J_{H,F}$ = 47.0 Hz, $J_{H,F}$ = 28.0 Hz); IR (film, ν, cm^{-1}) 3410, 2923,1002; HRMS ESI *m/z* : calculated for $C_9H_{17}FNaO_6$ $[M+Na]^+$ 263.0901. Found : 263.0907.

2-*C*-(2-Hydroxyethyl)-*myo*-inositol (*myo*-3)

To a solution of compound *myo*-**16** (2 g, 2.9 mmol) in MeOH (20 mL), CH_2Cl_2 (20 mL) and H_2O (2 mL) was added $Pd(OH)_2$ (500 mg, 20 mol %). The reaction mixture was placed in a Parr apparatus and was stirred under a hydrogen atmosphere at 45 psi overnight. After completion of the reaction, the mixture was filtered through a Celite® pad, washed with water and the filtrate was concentrated under reduced pressure. Compound *myo*-**3** (670 mg) was obtained in quantitative yield and used without further purification. ^{1}H NMR (400 MHz, D_2O) δ 2.00 (t, 2H, $J_{7,8}$ = 7.0 Hz, H-7), 3.27 (app t, 1H, $J_{4,5}$ = $J_{5,6}$ = 9.5 Hz, H-5), 3.34 (d, 2H, $J_{1,6}$ = $J_{3,4}$ = 9.5 Hz, H-1 and H-3), 3.59 (app t, 2H, H-4 and H-6), 3.69 (t, 2H, H-8); ^{13}C NMR (100.6 MHz, D_2O) δ 36.5 (C-7), 57.4 (C-8), 73.0 (C-1 and C-3), 73.4 (C-4 and C-6), 74.1 (C-5), 76.0 (C-2); IR (KBr, ν, cm^{-1}) 3398, 2923, 1654, 1458, 1121; HRMS ESI *m/z* : calculated for $C_8H_{16}NaO_7$ $[M+Na]^+$ 247.0788. Found: 247.0798.

1-*C*-(2-Hydroxyethyl)-*scyllo*-inositol (*scyllo*-3)

Compound *scyllo*-**16** (2 g, 2.9 mmol) was dissolved in MeOH (20 mL) then CH_2Cl_2 (20 mL), H_2O (2 mL) and $Pd(OH)_2$ (500 mg, 20 mol %) were added. The reaction mixture was placed in a Parr apparatus and stirred under a hydrogen atmosphere at 45 psi overnight. After completion of the reaction, the mixture was filtered through a Celite® pad, washed with water and the filtrate was concentrated under reduced pressure. Compound *scyllo*-**3** (670 mg, quantitative yield) was used without further purification. m.p. 88-89 °C; R_f 0 (9/1 CH_3CN/H_2O); ^{1}H NMR (400 MHz, D_2O) δ 1.81 (t, 2H, $J_{7,8}$ = 6.0 Hz, H-7), 3.27-3.48 (m, 5H, H-inositol), 3.83 (t, 2H, H-8); ^{13}C NMR (100.6 MHz, D_2O) δ 32.1 (C-7), 57.3 (C-8), 73.1 (C-3 and C-5), 75.4 (C-4), 75.8 (C-2), 76.4 (C-2 and C-6); IR (NaCl, ν, cm^{-1}) 3400, 2926, 1654, 1438; HRMS ESI *m/z* : calculated for $C_8H_{16}LiO_7$ $[M+Li]^+$ 231.1051. Found: 231.1059.

2-*C*-(3-Hydroxypropyl)-*myo*-inositol (*myo*-4)

Compound *myo*-**17** (2 g, 2.9 mmol) was dissolved in MeOH (20 mL) then CH_2Cl_2 (20 mL), H_2O (2 mL) and $Pd(OH)_2$ (500 mg, 20 mol %) were added. The reaction mixture was introduce in a Parr apparatus and was stirred under hydrogen atmosphere at 45 Psi overnight. After completion of the reaction, the mixture was filtered through a Celite pad, washed with water and the filtrate was concentrated under reduced pressure. Compound *myo*-**4** (700 mg) was obtained in quantitative yield and was used without purification. m.p. 151-152 °C; ^1H NMR (400 MHz, D_2O) δ 1.43-1.55 (m, 2H, H-8), 1.68-1.75 (m, 2H, H-7), 3.25 (app t, 1H, $J_{4,5} = J_{5,6}$ = 9.5 Hz, H-5), 3.36 (d, 2H, $J_{1,6} = J_{3,4}$ = 9.5 Hz, H-1 and H-3), 3.59 (t, 2H, H-4 and H-6), 3.59 (app t, 2H, $J_{8,9}$ = 7.0 Hz, H-9); ^{13}C NMR (100.6 MHz, D_2O) δ 26.0 (C-8), 29.9 (C-7), 62.1 (C-9), 71.6 (C-1 and C-3), 73.6 (C-4 and C-6), 74.2 (C-5), 76.9 (C-2); IR (KBr, ν, cm^{-1}) 3467, 3060, 1490, 1445; HR-ESI *m/z* : calculated for $C_9H_{18}NaO_7$ $[M+Na]^+$ 261.0945. Found: 261.0939.

1-*C*-(3-Hydroxypropyl)-*scyllo*-inositol (*scyllo*-4)

Compound *scyllo*-**17** (2 g, 2.9 mmol) was dissolved in a mixture of MeOH (20 mL), CH_2Cl_2 (20 mL) and H_2O (2 mL) and then $Pd(OH)_2$ (500 mg, 20 mol %) was added. The reaction mixture was introduced in a Parr apparatus and stirred under a hydrogen atmosphere at 45 psi overnight. After completion of the reaction, the mixture was filtered through a Celite® pad, washed with water and the filtrate was concentrated under reduced pressure. Compound *scyllo*-**4** (700 mg, quantitative yield) was used without further purification. m.p. 104-105° °C; R_f 0 (9/1 CH_3CN/H_2O); ^1H NMR (400 MHz, D_2O) δ 1.55-1.64 (m, 2H, H-7), 1.79-1.89 (m, 2H, H-8), 3.30-3.47 (m, 5H, H-inositol), 3.58 (t, 2H, $J_{8,9}$ = 5.0 Hz, H-9); ^{13}C NMR (100.6 MHz, D_2O) δ 26.4 (C-8), 26.4 (C-7), 63.0 (C-9), 73.2 (C-3 and C-5), 75.5 (C-4), 75.6 (C-1), 77.2 (C-2 and C-6); IR (KBr, ν, cm^{-1}) 3400, 2922, 1654, 1458, 1080.

2-*C*-(2-Fluoroethyl)-*myo*-inositol (*myo*-5)

To a solution of *myo*-**25** (50 mg, 0.097 mmol) in dry MeOH (10 mL) under argon, a catalytic amount of sodium was added at 0 °C and the mixture was stirred for 2 h at room temperature. Afterwards, the reaction mixture was neutralized by Amberlite IR-120 and water was added to solubilize the compound. The resin was filtered off and the filtrate was evaporated under reduced pressure to give *myo*-**5** (25mg, quantitative) R_f 0.30 (9/1 CH_3CN/H_2O); ^{1}H NMR (250 MHz, D_2O) δ 2.21 (dt, 2H, $J_{H,F}$ = 28.0 Hz, $J_{7,8}$ = 6.0 Hz, H-7), 3.33 (app t, 1H, $J_{5,4}$ = $J_{5,6}$ = 9.5 Hz, H-5), 3.46 (d, 2H, $J_{1,6}$ = $J_{3,4}$ = 9.5 Hz, H-1 and H-3), 3.64 (app t, 2H, H-4 and H-6), 4.67 (dt, 2H, $J_{H,F}$ = 47.0 Hz, H-8); ^{13}C NMR (62.9 MHz, D_2O) δ 34.5 (d, $J_{C,F}$ = 19.0 Hz, C-7), 72.7 (C-4 and C-6), 73.4 (C-1 and C-3), 74.0 (C-5), 75.9 (d, $J_{C,F}$ = 2.5 Hz, C-2), 81.5 (d, $J_{C,F}$ = 158.0 Hz, C-8); NMR (^{19}F, 235 MHz D_2O) δ -216.7 (tt, $J_{H,F}$ = 47.0 Hz, $J_{H,F}$ = 28.0 Hz); IR (KBr, ν, cm^{-1}) 3400, 1637, 1384; HRMS ESI *m/z* : calculated for $C_8H_{15}FNaO_6$ $[M+Na]^+$ 249.0745. Found: 429.0745.

2-*C*-(3-Fluoropropyl)-*myo*-inositol (*myo*-6)

To a solution of *myo*-**29** (48 mg, 0.097 mmol) in dry MeOH (10 mL) under argon a catalytic amount of sodium was added at 0 °C and the mixture was stirred for 2 h at room temperature. Afterwards, the reaction mixture was neutralized by Amberlite IR-120 and water was added to solubilize the precipitate. The resin was filtered off and the solvent was evaporated under reduced pressure to give *myo*-**6** (23 mg) in quantitative yield. R_f 0.38 (9/1 CH_3CN/H_2O); ^{1}H NMR (400 MHz, D_2O) δ 1.61-1.78 (m, 2H, H-9), 1.78-1.85 (m, 2H, H-7), 3.29 (app t, 1H, $J_{4,5}$ = $J_{5,6}$ = 10.0 Hz, H-5), 3.38 (d, 2H, $J_{1,6}$ = $J_{3,4}$ = 10.0 Hz, H-1 and H-3), 3.61 (app t, 2H, H-4 and H-6), 4.54 (dt, 2H, $J_{H,F}$ = 47.0 Hz and $J_{8,9}$ = 6.0 Hz, H-9); ^{13}C NMR (62.9 MHz, D_2O) δ 24.5 (d, $J_{C,F}$ = 20.0 Hz, C-8), 29.2 (d, $J_{C,F}$ = 5.0 Hz, C-7), 71.6 (C-1 and C-3), 73.5 (C-4 and C-6), 74.1 (C-5), 76.6 (C-2), 85.5 (d, $J_{C,F}$ = 159.0 Hz, C-9); NMR (^{19}F, 235 MHz D_2O) δ -

216.6 (tt, $J_{H,F}$ = 47.0 Hz and $J_{H,F}$ = 25.0 Hz); IR (film, ν, cm^{-1}) 3436, 2924, 1384, 1124; HRMS ESI *m/z* : calculated for $C_9H_{17}FNaO_6$ $[M+Na]^+$ 263.0901. Found: 263.0896.

1-*C*-(3-Fluoropropyl)-*scyllo*-inositol (*scyllo*-6)

To a solution of *scyllo*-**29** (100 mg, 0.097 mmol) in dry MeOH (10 mL) under argon a catalytic amount of sodium was added at 0 °C and the mixture was stirred for 2 h at room temperature. Afterwards the reaction mixture was neutralized by Amberlite IR-120 and water was added to solubilize the compound. The resin was filtered off and the filtrate was evaporated under reduced pressure to give *scyllo*-**6** (50 mg, quantitative) as an oil R_f 0.42 (9/1 CH_3CN/H_2O); ^1H NMR (250 MHz, D_2O) δ 1.63-1.75 (m, 2H, H-7), 1.85-2.16 (m, 2H, H-8), 3.35-3.55 (m, 5H, H-inositol), 4.55 (dt, 2H, $J_{H,F}$ = 47 Hz, $J_{8,9}$ = 6.0 Hz, H-9); ^{13}C NMR (62.9 MHz, D_2O) δ 25.0 (d, $J_{C,F}$ = 19.0 Hz, C-8), 25.6 (d, $J_{C,F}$ = 5.0 Hz, C-7), 73.1 (C-3 and C-5), 75.4 (C-1 and C-4), 77.1 (C-2 and C-6), 86.2(d, $J_{C,F}$ = 158.0 Hz, C-9); NMR (^{19}F, 235 MHz D_2O) δ -216.9 (tt, $J_{H,F}$ = 47.0 Hz, $J_{H,F}$ = 27.0 Hz); IR (KBr, ν, cm^{-1}) 3394, 2923, 1654, 1005; HRMS ESI *m/z* : calculated for $C_9H_{17}FNaO_6$ $[M+Na]^+$ 263.0901. Found: 263.0905.

1,3,4,5,6-Pentakis-*O*-(phenylmethyl)-2-*O*-(2-propen-1-yl)-*myo*-inositol (*myo*-9)

To a solution of *myo*-**8** (8 g, 12.7 mmol) in allylbromide (200 mL) was added NaH (3 g, 12.7 mmol) and the mixture was stirred for 2 h at 70 °C. The solvent was removed under reduced pressure, and the residue was diluted and extracted in CH_2Cl_2 (3 x 50 mL). The combined organic layers were dried over $MgSO_4$, filtered and the solvent was removed under reduced pressure. The crude product was purified by flash chromatography (silica gel, hexane 100% to hexane/EtOAc 80:20) to give *myo*-**9** (6.8 g, 80% yield) as a white solid m.p. 82-83 °C; R_f 0.80 (8/2 H/A); ^1H NMR (400 MHz, $CDCl_3$) δ 3.34 (dd, 2H, $J_{3,4}$ = $J_{1,6}$ = 9.5 Hz, $J_{3,2}$ = $J_{1,2}$ = 2.0 Hz, H-1 and H-3), 3.48 (app t, 1H, $J_{5,4}$ = $J_{5,6}$ = 9.5 Hz, H-5), 3.98 (app t, 1H, H-2), 4.05 (app t, 2H, H-4 and H-6), 4.35 (bd, 2H, $J_{7,8}$ = 5.5 Hz, H-7), 4.69 (s, 4H, CH_2Ph), 4.86 (d, 2H,

J = 10.5 Hz, CH_2Ph), 4.89 (s, 2H, CH_2Ph), 4.93 (d, 2H, J = 10.5 Hz, CH_2Ph), 5.19 (dd, 1H, $J_{8,9}$ = 10.5 Hz, $J_{9,9'}$ = 2.0 Hz, H-9), 5.31 (dd, 1H, $J_{8,9'}$ = 16.0 Hz, $J_{9,9'}$ = 2.0 Hz, H-9'), 6.00 (ddt, 1H, H-8), 7.24-7.42 (m, 25H, H-Ar); ^{13}C NMR (100.6 MHz, $CDCl_3$) δ 72.9 ($2CH_2$), 73.6 (C-7), 74.1 (C-2), 76.0 ($2CH_2$), 76.0 (CH_2), 80.9 (C-3 and C-1), 81.8 (C-4 and C-6), 83.8 (C-5), 116.9 (C-9), 127.6 (3C-Ar), 127.8 (5C-Ar), 127.9 (2C-Ar), 128.2 (3C-Ar), 128.4 (3C-Ar), 128.5 (1C-Ar), 128.5 (3C-Ar), 135.9 (C-8), 138.5 (2Cq-Ar), 139.0 (Cq-Ar), 139.0 (2Cq-Ar); IR (film, ν, cm^{-1}) 3030, 2868, 1454, 1071; HRMS ESI *m/z* : calculated for $C_{44}H_{46}NaO_6$ $[M+Na]^+$ 693.3187. Found: 693.3177.

1,3,4,5,6-Pentakis-*O*-(phenylmethyl)-2-*O*-(2-propen-1-yl)-*scyllo*-inositol (*scyllo*-**9**)

To a solution of *scyllo*-**8** (8 g, 12.7 mmol) in allylbromide (200 mL) was added NaH (3 g, 12.7 mmol) and the mixture was stirred for 2 h at 70 °C. The solvent was removed under reduced pressure, and the residue was diluted and extracted in CH_2Cl_2 (3 x 50 mL). The combined organic layers were dried over $MgSO_4$, filtered and the solvent was removed under reduced pressure. The crude product was purified by flash chromatography (silica gel, hexane 100% to hexane/EtOAc 80:20) to give *scyllo*-**9** (7.0 g, 82% yield) as an oil R_f 0.71 (8/2 H/A); ^1H NMR (400 MHz, $CDCl_3$) δ 3.39-3.45 (m, 1H, H-4), 3.48-3.57 (m, 5H, H-inositol), 4.36 (bd, 2H, $J_{7,8}$ = 5.5 Hz, H-7), 4.82-4.90 (m, 10H, $5CH_2Ph$), 5.17 (dd, 1H, $J_{8,9}$ = 10.5 Hz, $J_{9,9'}$ = 1.5 Hz, H-9), 5.28 (dd, 1H, $J_{8,9'}$ = 17.0 Hz, $J_{9,9'}$ = 1.5 Hz, H-9'), 5.97 (ddt, 1H, H-8), 7.18-7.42 (m, 25H, H-Ar); ^{13}C NMR (100.6 MHz, $CDCl_3$) δ 74.8 (C-7), 76.1 ($3CH_2$), 76.2 ($3CH_2$), 82.7 (C-4), 83.0 (C-2 and C-6 or C-3 and C-5), 83.1 (C-1, C-2 and C-6 or C-3 and C-5), 117.0 (C-9), 127.8 (3C-Ar), 127.8 (2C-Ar), 128.0 (4C-Ar), 128.2 (3C-Ar), 128.5 (8C-Ar), 135.2 (C-8), 138.7 (5Cq-Ar); IR (film, ν, cm^{-1}) 2925, 1638, 1067; HRMS ESI *m/z* : calculated for $C_{44}H_{46}NaO_6$ $[M+Na]^+$ 693.3187. Found: 693.3185.

2-*O*-(3-Hydroxypropyl)-1,3,4,5,6-pentakis-*O*-(phenylmethyl)-*myo*-inositol (***myo*-10**)

To a solution of *myo*-**9** (7 g, 10 mmol) in dry THF (250 mL) was added dropwise BH_3 (120 mL of a 1 M solution in hexane, 120 mmol) under argon at 0 °C. The mixture was stirred for 6 h at room temperature, then H_2O_2 (30%, 500 mL) and an aqueous solution of 2 M NaOH (600 mL) were added slowly. After 12 h, the mixture was diluted with ethyl acetate (200 mL) and extracted. The remaining aqueous phase was further extracted with ethyl acetate (2 x 100 mL), the combined organic layers were dried over $MgSO_4$, filtered and the solvent removed under reduced pressure. The crude product was purified by column chromatography (silica gel, hexane 100% to hexane/EtOAc 70:30) to give *myo*-**10** (5.6 g, 78% yield) as a white solid m.p. 70-71 °C; R_f 0.3 (7/3 H/A); ^{1}H NMR (400 MHz, $CDCl_3$) δ 1.78 (qt, 2H, $J_{8,7} = J_{8,9}$ = 5.5 Hz, H-8), 3.32 (dd, 2H, $J_{3,4} = J_{1,6}$ = 9.5 Hz, $J_{3,2} = J_{1,2}$ = 2.0 Hz, H-1 and H-3), 3.43 (app t, 1H, $J_{5,4} = J_{5,6}$ = 9.5 Hz, H-5), 3.74 (app t, 1H, H-2), 3.78 (t, 2H, H-7), 3.92 (t, 2H, H-9), 3.94 (app t, 2H, H-4 and H-6), 4.65 (d, 2H, *J* = 11.5 Hz, CH_2Ph), 4.71 (d, 2H, *J* = 11.5 Hz, CH_2Ph), 4.83 (d, 2H, *J* = 10.5 Hz, CH_2Ph), 4.86 (s, 2H, CH_2Ph), 4.87 (d, 2H, *J* = 11.5 Hz, CH_2Ph), 7.24-7.38 (m, 25H, H-Ar); ^{13}C NMR (100.6 MHz, $CDCl_3$) δ 32.2 (C-8), 60.8 (C-9), 72.2 (C-7), 73.4 (2CH_2), 76.1 (2CH_2), 76.1 (CH_2), 76.8 (C-2), 80.5 (C-3 and C-1), 81.8 (C-4 and C-6), 83.5 (C-5), 127.7 (3C-Ar), 128.0 (2C-Ar), 128.1 (5C-Ar), 128.2 (3C-Ar), 128.5 (3C-Ar), 128.5 (1C-Ar), 128.7 (3C-Ar), 138.0 (2Cq-Ar), 138.8 (3Cq-Ar); IR (KBr, ν, cm^{-1}) 3463, 3030, 2871, 1070; HRMS ESI *m/z* : calculated for $C_{44}H_{48}NaO_7$ $[M+Na]^+$ 711.3292. Found: 711.3292.

1-*O*-(3-Hydroxypropyl)-2,3,4,5,6-pentakis-*O*-(phenylmethyl)-*scyllo*-inositol (***scyllo*-10**)

Obtained in 75% yield starting from *scyllo*-**9** using the above protocol m.p. 108-109 °C; R_f 0.18 (7/3 H/A); ^{1}H NMR (400 MHz, $CDCl_3$) δ 1.83 (qt, 2H, $J_{8,7} = J_{8,9}$ = 6.0 Hz, H-8), 2.05 (brs, 1H, OH), 3.37 (app t, 1H, $J_{3,4} = J_{4,5}$ = 9.5 Hz, H-4), 3.46-3.59 (m, 5H, H-inositol), 3.71 (t, 2H, H-9), 4.00 (t, 2H, H-7), 4.86 (d, 2H, *J* = 11.0 Hz, CH_2Ph), 4.87 (d, 2H, *J* = 11.0 Hz,

CH_2Ph), 4.89 (s, 2H, CH_2Ph), 4.90 (d, 2H, J = 11.0 Hz, CH_2Ph), 4.92 (d, 2H, J = 11.0 Hz, CH_2Ph), 7.26-7.40 (m, 25H, H-Ar); ^{13}C NMR (100.6 MHz, $CDCl_3$) δ 33.0 (C-8), 61.5 (C-9), 72.7 (C-7), 75.9 ($2CH_2$), 76.0 ($2CH_2$), 76.1 (CH_2), 82.8 (C-2 and C-6 or C-3 and C-5), 82.9 (C-1), 83.0 (C-2 and C-6 or C-3 and C-5), 83.1 (C-4), 127.7 (3C-Ar), 127.7 (2C-Ar), 127.8 (4C-Ar), 127.9 (4C-Ar), 128.0 (2C-Ar), 128.5 (5C-Ar), 128.6 (5C-Ar), 138.4 (3Cq-Ar), 138.5 (2Cq-Ar); IR (film, ν, cm^{-1}) 3468, 3030, 2823, 1070; HRMS ESI *m/z* : calculated for $C_{44}H_{48}NaO_7$ $[M+Na]^+$ 711.3292. Found: 711.3259.

1,3,4,5,6-Pentaacetyl-2-*O*-(3-triphenylmethyloxypropyl)-*myo*-inositol (*myo*-11)

To a solution of *myo*-**1** (190 mg, 0.80 mmol) in pyridine (20 mL) were added trityl chloride (675 mg, 2.42 mmol) and a catalytic amount of DMAP. The mixture was stirred at 40 °C for 3 d and then the reaction was allowed to reach room temperature. Acetic anhydride (10 mL) was added and the solution was stirred for further 2 h. The volatiles were removed under reduced pressure and the residue was purified by flash column chromatography (silica gel, hexane/EtOAc 80:20 to 60:40) to give *myo*-**11** (440 mg, 80% yield). m.p. 128-130 °C; R_f 0.55 (1/1 H/A); ^1H NMR (400 MHz, D_2O) δ 1.84 (qt, 2H, $J_{8,7} = J_{8,9}$ = 6.5 Hz, H-8), 1.95 (s, 6H, 2Me), 1.99 (s, 3H, Me), 1.99 (s, 6H, 2Me), 3.19 (t, 2H, H-9), 3.72 (t, 2H, H-7), 3.94 (app t, 1H, $J_{2,1} = J_{2,3}$ = 2.5 Hz, H-2), 4.93 (dd, 2H, $J_{3,4} = J_{1,6}$ = 10.0 Hz, $J_{3,2} = J_{1,2}$ = 2.5 Hz, H-1 and H-3), 5.10 (app t, 1H, $J_{5,4} = J_{5,6}$ = 10.0 Hz, H-5), 5.48 (app t, 2H, H-4 and H-6), 7.22 (brt, 3H, J = 7.5 Hz, H-Ar), 7.29 (brt, 6H, J = 7.5 Hz, H-Ar), 7.41 (brd, 6H, H-Ar); ^{13}C NMR (100.6 MHz, D_2O) δ 20.6 (Me), 20.7 (4Me), 30.8 (C-8), 60.5 (C-9), 70.0 (C-4 and C-6), 71.0 (C-1 and C-3), 71.2 (C-5), 71.4 (C-7), 76.0 (C-2), 127.0 (3C-Ar), 127.9 (6C-Ar), 128.7 (6C-Ar), 144.3 (3Cq-Ar), 169.7 (2CO), 169.9 (2CO), 170.3 (CO); IR (film, ν, cm^{-1}) 2936, 1756, 1368, 1230; HRMS ESI *m/z* : calculated for $C_{38}H_{42}NaO_{12}$ $[M+Na]^+$ 713.2568. Found: 713.2568.

1,3,4,5,6-Pentaacetyl-2-*O*-(3-triphenylmethyloxypropyl)-*scyllo*-inositol (***scyllo*-11**)

Scyllo-**11** was obtained as a white solid in 76% yield starting from *scyllo*-**1** using the above protocol. R_f 0.62 (1/1 H/A); ^{1}H NMR (400 MHz, $CDCl_3$) δ 1.75 (qt, 2H, $J_{7,8} = J_{8,9} = 6.5$ Hz, H-8), 1.95 (s, 3H, Me), 1.98 (s, 6H, 2Me), 2.00 (s, 3H, Me), 2.05 (s, 3H, Me), 3.09 (t, 2H, H-9) 3.50 (app t, 1H, $J_{1,2} = J_{1,6} = 7.0$ Hz, H-1), 3.68 (t, 2H, H-7), 5.08-5.21 (m, 5H, H-inositol), 7.20-7.41 (mt, 15H, H-Ar); ^{13}C NMR (100.6 MHz, $CDCl_3$) δ 20.5 (Me), 20.6 (2Me), 20.7 (2Me), 31.0 (C-8), 60.5 (C-9), 70.2 (C-7), 70.6 (C-2 and C-6 or C-3 and C-5), 70.8 (C-4), 71.7 (C-2 and C-6 or C-3 and C-5), 78.1 (C-1), 86.7 (Cq-Tr), 127.1 (3C-Ar), 127.9 (6C-Ar), 128.7 (6C-Ar), 144.3 (3Cq-Ar), 169.3 (2CO), 169.5 (CO), 169.9 (2CO); IR (KBr, ν, cm^{-1}) 3061, 1755, 1446, 1228; HRMS ESI *m/z* : calculated for $C_{38}H_{42}NaO_{12}$ $[M+Na]^+$ 729.2308. Found: 729.2307.

2-*O*-(3-Hydroxypropyl)-1,3,4,5,6-pentaacetyl-*myo*-inositol (***myo*-12)**

A solution of *myo*-**11** (1 g, 1.45 mmol) and $FeCl_3$ (1.01 g, 6.8 mmol) diluted in dry CH_2Cl_2 (10 mL) was stirred at room temperature overnight. The reaction was quenched by adding water and extracted with CH_2Cl_2 (3 x 100 mL). The combined organic layers were dried over $MgSO_4$, filtered and the solvent was removed under reduced pressure. The crude product was purified by flash column chromatography (silica gel, hexane/EtOAc 80:20 to 20:80) to give *myo*-**12** (455 mg, 70% yield) as a white solid m.p. 148-150 °C; R_f 0.70 (A); ^{1}H NMR (400 MHz, $CDCl_3$) δ 1.83 (qt, 2H, $J_{8,7} = J_{8,9} = 6.0$ Hz, H-8), 1.99 (s, 9H, 3Me), 2.07 (s, 6H, 2Me), 3.78 (t, 2H, H-7), 3.82 (t, 2H, H-9), 4.00 (app t, 1H, $J_{2,1} = J_{2,3} = 2.5$ Hz, H-2), 4.97 (dd, 2H, $J_{3,4} = J_{1,6} = 9.5$ Hz, $J_{3,2} = J_{1,2} = 2.5$ Hz, H-1 and H-3), 5.13 (app t, 1H, $J_{5,4} = J_{5,6} = 9.5$ Hz, H-5), 5.53 (app t, 2H, H-4 and H-6); ^{13}C NMR (100.6 MHz, $CDCl_3$) δ 20.6 (Me), 20.7 (2Me), 20.8 (2Me), 32.6 (C-8), 60.2 (C-9), 69.9 (C-4 and C-6), 71.1 (C-1, C-3 and C-5), 71.7 (C-7),

76.4 (C-2), 169.9 (2CO), 169.9 (2CO), 169.9 (CO); IR (KBr, ν, cm^{-1}) 3448, 2944, 1755, 1229; HRMS ESI *m/z* : calculated for $C_{19}H_{28}NaO_{12}$ $[M+Na]^+$ 471.1473. Found: 471.1473.

1-*O*-(3-Hydroxypropyl)-2,3,4,5,6-pentaacetyl-*scyllo*-inositol (*scyllo*-12)

Scyllo-**12** was obtained as a white solid in 73% yield starting from *scyllo*-**11** using the above protocol. m.p. 184-185 °C; R_f 0.75 (A); ^1H NMR (400 MHz, $CDCl_3$) δ 1.74 (qt, $J_{7,8}$ = $J_{8,9}$ = 6.0 Hz, H-8), 2.02 (s, 3H, Me), 2.03 (s, 6H, 2Me), 2.10 (s, 6H, 2Me), 3.58 (app t, 1H, $J_{1,2}$ = $J_{1,6}$ = 10.0 Hz, H-1), 3.67 (t, 2H, H-9), 3.73 (t, 2H, H-7), 5.14-5.27 (m, 5H, H-inositol); ^{13}C NMR (100.6 MHz, $CDCl_3$) δ 20.6 (Me), 20.6 (2Me), 20.8 (2Me), 32.7 (C-8), 60.2 (C-9), 70.2 (C-7), 10.5 (C-2 and C-6 or C-3 and C-5), 70.7 (C-4), 71.5 (C-2 and C-6 or C-3 and C-5), 78.3 (C-1), 169.5 (CO), 169.7 (2CO), 169.9 (2CO); IR (KBr, ν, cm^{-1}) 3472, 2941, 1747, 1226; HRMS ESI *m/z* : calculated for $C_{19}H_{28}NaO_{12}$ $[M+Na]^+$ 471.1473. Found: 471.1471.

2-*O*-(3-Fluoroypropyl)-1,3,4,5,6-pentaacetyl-*myo*-inositol (*myo*-13)

To a solution of *myo*-**12** (50 mg, 0.1 mmol) in dry CH_2Cl_2 (8 mL) was added DAST (0.2 mL). The mixture was stirred at room temperature for 5 min, then quenched by adding MeOH and diluted with CH_2Cl_2 (20 mL). The solution was washed with a saturated aqueous solution of sodium hydrogencarbonate and water, dried over $MgSO_4$ and filtered, and the solvent was removed under reduced pressure. The crude product was purified by flash column chromatography (silica gel, hexane/EtOAc 80:20 to 40:60) to give *myo*-**13** (48 mg, 95% yield) as a white solid. m.p. 156-157 °C; R_f 0.30 (1/1 H/A); ^1H NMR (400 MHz, $CDCl_3$) δ 1.95 (dqt, 2H, $J_{H,F}$ = 27.0 Hz, $J_{8,7}$ = $J_{8,9}$ = 5.5 Hz, H-8), 1.99 (s, 3H, Me), 2.00(s, 6H, 2Me), 2.06 (s, 6H, 2Me), 3.73 (t, 2H, H-7), 3.99 (app t, 1H, $J_{2,1}$ = $J_{2,3}$ = 2.5 Hz, H-2), 4.63 (dt, 2H, $J_{H,F}$ = 47.0 Hz, $J_{8,9}$ = 5.5 Hz, H-9), 4.95 (dd, 2H, $J_{3,4}$ = $J_{1,6}$ = 10.0 Hz, $J_{3,2}$ = $J_{1,2}$ = 2.5 Hz, H-1 and H-3), 5.12 (app t, 1H, $J_{5,4}$ = $J_{5,6}$ = 10.0 Hz, H-5), 5.53 (app t, 2H, H-4 and H-6); ^{13}C NMR (100.6 MHz, $CDCl_3$) δ 20.6 (Me), 20.7 (4Me), 31.3 (d, $J_{C,F}$ = 20.0 Hz, C-8), 69.7 (d,

$J_{C,F}$ = 4.5 Hz, C-7), 69.9 (C-4 and C-6), 71.1 (C-1 and C-3), 71.2 (C-5), 76.8 (C-2), 80.4 (d, $J_{C,F}$ = 164.0 Hz, C-9), 169.9 (2CO), 169.9 (CO), 170.0 (2CO); NMR (^{19}F, 235 MHz CDCl$_3$) δ -223.3 (tt, $J_{H,F}$ = 47.0 Hz, $J_{H,F}$ = 27.0 Hz); IR (film, ν, cm^{-1}) 2971, 1755, 1640, 1229, 2971, 1755, 1640, 1229; HRMS ESI *m/z* : calculated for $C_{19}H_{27}FNaO_{11}$ [M+Na]$^+$ 473.1430. Found: 473.1410.

1-*O*-(3-Fluoropropyl)-2,3,4,5,6-pentaacetyl-*scyllo*-inositol (***scyllo*-13**)

Scyllo-**13** was obtained as a white solid in 92% yield starting from *scyllo*-**12** using the above protocol. m.p. 199-200 °C; R$_f$ 0.33 (1/1 H/A); ^{1}H NMR (400 MHz, CDCl$_3$) δ 1.84 (dt, 2H, $J_{H,F}$ = 27.0 Hz, $J_{7,8}$ = $J_{8,9}$ = 6.0 Hz, H-8), 1.98 (s, 3H, Me), 1.99 (s, 6H, 2Me), 2.05 (s, 6H, 2Me), 3.54 (app t, 1H, $J_{1,2}$ = $J_{1,6}$ = 9.0 Hz , H-1), 3.68 (t, 2H, H-7), 4.44 (dt, $J_{H,F}$ = 47.0 Hz, 2H, H-9), 5.09-5.25 (m, 5H, H-inositol); ^{13}C NMR (100.6 MHz, CDCl$_3$) δ 20.6 (Me), 20.6 (2Me), 20.7 (2Me), 31.1 (d, $J_{C,F}$ = 20.0 Hz, C-8), 68.6 (d, $J_{C,F}$ = 5.0 Hz, C-7), 70.3 (C-4), 70.5 (C-2 and C-6 or C3 and C-5), 71.7 (C-2 and C-6 or C-3 and C-5),78.2 (C-1), 80.3 (d, $J_{C,F}$ = 163.0 Hz, C-9), 169.5 (CO), 169.6 (2CO), 169.9 (2CO); NMR (^{19}F, 235 MHz CDCl$_3$) δ -223.5 (tt, $J_{H,F}$ = 47.0 Hz, $J_{H,F}$ = 27.0 Hz); IR (film, ν, cm^{-1}) 2961, 1745, 1227; HRMS ESI *m/z* : calculated for $C_{19}H_{27}FNaO_{11}$ [M+Na]$^+$ 473.1430. Found: 473.1451.

1-*C*-(1-Vinyl)-2,3,4,5,6-pentakis-*O*-(phenylmethyl)-*scyllo*-inositol (***scyllo*-14**)

To a solution of **7** (10 g, 15.9 mmol) in dry THF (50 mL) was added dropwise vinylmagnesium bromide (105 mL of a 1 M solution in diethyl ether, 105 mmol) under argon at 0 °C. The mixture was stirred for 2 h at room temperature, then quenched by adding a saturated aqueous solution of NH_4Cl (100 mL) and extracted with EtOAc (200 mL). The remaining aqueous phase was further extracted with ethyl acetate (2 x 50 mL), the combined organic layers dried over $MgSO_4$ and filtered. The solvent was removed under reduced

pressure. The crude product was purified by flash column chromatography (silica gel, hexane 100% to hexane/EtOAc 85:15) to give *scyllo*-**14** (3.8 g, 37%) as a white solid and ***myo*-14** (5.7 g, 56%) as a yellow oil. R_f 0.62 (8/2 H/A); ^{1}H NMR (400 MHz, $CDCl_3$) δ 2.21(s, 1H, OH), 3.61 (brd, 2H, $J_{2,3}$ = $J_{5,6}$ = 9.0 Hz, H-2 and H-6), 3.70-3.80 (m, 3H, H-3, H-4 and H-5), 4.89 (d, 2H, J = 11.0 Hz, CH_2Ph), 4.90 (d, 2H, J = 11.0 Hz, CH_2Ph), 4.93 (d, 2H, J = 11.0 Hz, CH_2Ph), 4.95 (d, 2H, J = 11.0 Hz, CH_2Ph), 4.98 (s, 2H, CH_2Ph), 5.56 (dd, 1H, $J_{7,8}$ = 11.0 Hz et J8,8' = 1.5 Hz, H-8), 5.72 (dd, 1H, $J_{7,8'}$ = 17.0 Hz, H-8'), 6.08 (dd, 1H, H-7), 7.31-7.40 (m, 25H, H-Ar); ^{13}C NMR (100.6 MHz, $CDCl_3$) δ 75.9(4CH_2), 76.2 (CH_2), 78.6 (C-1), 82.5 (C-3 and C-5), 83.7 (C-4), 84.7 (C-2 and C-6), 119.2 (C-8), 127.8 (4C-Ar), 127.8 (5C-Ar), 128.0 (6C-Ar), 128.5 (4C-Ar), 128.5 (6C-Ar), 135.3 (C-7), 138.5 (3Cq-Ar), 138.8 (2Cq-Ar); IR (film, ν, cm^{-1}) 3552, 3030, 2910, 1497, 1066; HRMS ESI *m/z* : calculated for $C_{43}H_{44}NaO_6$ $[M+Na]^+$ 679.3030. Found: 679.3050.

2-*C*-(1-Vinyl)-1,3,4,5,6-pentakis-*O*-(phenylmethyl)-*myo*-inositol (***myo*-14**)

m.p. 119-120 °C; R_f 0.77 (8/2 H/A); ^{1}H NMR (400 MHz, $CDCl_3$) δ 3.45 (d, 2H, $J_{1,6}$ = $J_{3,4}$ = 10.0 Hz, H-1 and H-3), 3.65 (app t, 1H, $J_{4,5}$ = $J_{5,6}$ = 10.0 Hz, H-5), 4.06 (app t, 2H, H-4 and H-6), 4.70 (d, 2H, J = 11.0 Hz, CH_2Ph), 4.85 (d, 2H, J = 10.0 Hz, CH_2Ph), 4.93 (d, 2H, J = 11.0 Hz, CH_2Ph), 4.98 (d, 2H, J = 10.0 Hz, CH_2Ph), 4.99 (s, 2H, CH_2Ph), 5.43 (dd, 1H, $J_{7,8}$ = 10.5 Hz et J8,8' = 1.0 Hz, H-8), 5.64 (dd, 1H, $J_{7,8'}$ = 17.0 Hz, H-8'), 5.82 (dd, 1H, H-7), 7.30-7.40 (m, 25 H, H-Ar); ^{13}C NMR (100.6 MHz, $CDCl_3$) δ 76.0 (3CH_2), 76.0 (2CH_2), 78.1 (C-2), 82.0 (C-1 and C-3), 82.6 (C-4 and C-6), 83.0 (C-5), 116.8 (C-8), 127.6 (3C-Ar), 127.9 (3C-Ar), 127.9 (4C-Ar), 128.3 (4C-Ar), 128.4 (6C-Ar), 128.5 (5C-Ar), 137.9 (2Cq-Ar), 138.6 (Cq-Ar), 138.7 (2Cq-Ar), 140.0 (C-7); IR (film, ν, cm^{-1}) 3551, 3031, 2864, 1453, 1356, 1060; HRMS ESI *m/z* : calculated for $C_{43}H_{44}NaO_6$ $[M+Na]^+$ 679.3030. Found : 679.3022.

2-*C*-(2-Allyl)-1,3,4,5,6-pentakis-*O*-(phenylmethyl)-*myo*-inositol (***myo*-15**)

To a solution of **7** (10 g, 15.9 mmol) in dry THF (50 mL) was added dropwise allylmagnesium bromide (127 mL of a 1 M solution in diethyl ether, 127.2 mmol) under argon at 0 °C. The mixture was stirred for 2 h at room temperature, then quenched by adding a saturated aqueous solution of NH_4Cl (100 mL) and extracted with EtOAc (200 mL). The remaining aqueous phase was further extracted with ethyl acetate (2 x 50 mL) and the combined organic layers were dried over $MgSO_4$, and filtered. The solvents were removed under reduced pressure and the crude product was purified by flash column chromatography (silica gel, hexane 100% to hexane/EtOAc 85:15) to give *scyllo*-**15** (4.0 g, 38%) and *myo*-**15** (5.6 g, 53%) R_f 0.7 (8/2 H/A); ^{1}H NMR (400 MHz, $CDCl_3$) δ 2.36 (brs, 1H, OH), 2.66 (d, 2H, $J_{7,8}$ = 7.5 Hz, H7), 3.44 (d, 2H, $J_{1,6}$ = $J_{3,4}$ = 9.5 Hz, H-1 and H-3), 3.56 (app t, 1H, $J_{4,5}$ = $J_{5,6}$ = 9.5 Hz, H-5), 4.08 (app t, 2H, H-4 and H-6), 4.72 (d, 2H, *J* = 11.0 Hz, CH_2Ph), 4.87 (d, 2H, *J* = 11.0 Hz, CH_2Ph), 4.96 (s, 2H, CH_2Ph), 4.99 (d, 2H, *J* = 11.0 Hz, CH_2Ph), 5.06 (d, 2H, *J* = 11.0 Hz, CH_2Ph), 5.07-5.15 (m, 2H, H-9), 5.60 (ddt, 1H, $J_{8,9}$ = 17.5 Hz, $J_{8,9'}$= 9.5 Hz, H-8), 7.25-7.40 (m, 25H, H-Ar); ^{13}C NMR (100.6 MHz, $CDCl_3$) δ 39.6 (C-7), 75.4 ($2CH_2$), 75.9 ($3CH_2$), 77.7 (C-2), 79.8 (C-3 and C-1), 83.3 (C-4, C-6 and C-5), 119.5 (C-9), 127.7 (3C-Ar), 127.8 (3C-Ar), 127.9 (5C-Ar), 127.9 (4C-Ar), 128.5 (10C-Ar), 132.9 (C-8), 138.3 (2Cq-Ar), 138.6 (3Cq-Ar); IR (NaCl, ν, cm^{-1}) 3553, 3063, 3031, 2866; ESI *m/z* : calculated for $C_{44}H_{46}NaO_6$ $[M+Na]^+$. Found: 693 $[M+Na]^+$.

1-*C*-(2-Allyl)-2,3,4,5,6-pentakis-*O*-(phenylmethyl)-*scyllo*-inositol (***scyllo*-15**)

R_f 0.73 (8/2 H/A); ^{1}H NMR (400 MHz, $CDCl_3$) δ 2.57 (d, 2H, $J_{7,8}$ = 7.5 Hz, H-7), 3.54 (d, 2H, $J_{2,3}$ = $J_{5,6}$ = 8.5 Hz, H-2 and H-6), 3.60-3.70 (m, 3H, H-3, H-4 and H-5), 4.80-4.96 (m, 10H, CH_2Ph), 5.20 (brd, 1H, *J* = 17.5 Hz, H-9), 5.21 (brd, 1H, *J* = 10.0 Hz, H-9'), 6.19 (ddt, 1H, H-8), 7.25-7.42 (m, 25H, H-Ar). ; ^{13}C NMR (100.6 MHz, $CDCl_3$) δ 36.3 (C-7), 76.0 ($2CH_2$), 76.2 ($2CH_2$ and C-1),76.4 (CH_2) 82.5 (C-3 and C-5), 83.8 (C-4), 85.5 (C-2 and C-6),

119.2 (C-9), 127.6 (2C-Ar), 127.7 (4C-Ar), 127.8 (3C-Ar), 128.0 (2C-Ar), 128.1 (4C-Ar), 128.5 (4C-Ar), 128.5 (4C-Ar), 128.6 (2C-Ar), 134.7 (C-8), 138.6 (2Cq-Ar), 138.6 (Cq-Ar), 139.0 (2Cq-Ar); IR (NaCl, ν, cm^{-1}) 3554, 3064, 3030, 2913, 1497,1066; ESI *m/z* : calculated for $C_{44}H_{46}NaO_6$ $[M+Na]^+$. Found: 693 $[M+Na]^+$.

2-*C*-(2-Hydroxyethyl)-3,4,5,6-tetrakis-*O*-(phenylmethyl)-*myo*-inositol (***myo*-16**)

To a solution of *myo*-**14** (10.0 g, 15.2 mmol) in dry THF (250 mL) was added dropwise BH_3 (60 mL of a 1 M solution in hexane, 60 mmol) under argon at 0 °C. The mixture was stirred for 8 h at room temperature, then H_2O_2 (30%, 500 mL) and an aqueous solution of 2 M NaOH (600 mL) were added slowly. After 12 h, the mixture was diluted with ethyl acetate (200 mL) and extracted. The remaining aqueous phase was further extracted with ethyl acetate (2 x 100 mL), the combined organic layers were dried over $MgSO_4$, filtered and the solvent removed under reduced pressure. The crude product was purified by flash column chromatography (silica gel, hexane 100% to hexane/EtOAc 70:30) to give *myo*-**16** (8.2 g, 80% yield) as a white solid. m.p. 140-141 °C; R_f 0.55 (6/4 H/A); ^1H NMR (400 MHz, $CDCl_3$) δ 1.94 (t, 2H, $J_{7,8}$ = 6.5 Hz, H-7), 2.75 (s, 1H, OH3.37 (d, 2H, $J_{1,6} = J_{3,4}$ = 10.0 Hz, H-1 and H-3), 3.52 (t, 2H, H-8), 3.57 (app t, 1H, $J_{4,5} = J_{5,6}$ = 10.0 Hz, H-5), 4.07 (app t, 2H, H-4 and H-6), 4.70 (d, 2H, J = 11.0 Hz, CH_2Ph), 4.85 (d, 2H, J = 11.0 Hz, CH_2Ph), 4.94 (s, 2H, CH_2Ph), 4.97 (d, 2H, J = 11.0 Hz, CH_2Ph), 5.05 (d, 2H, J = 11.0 Hz, CH_2Ph), 7.25-7.35 (m, 25H, H-Ar); ^{13}C NMR (100.6 MHz, $CDCl_3$) δ 37.3 (C-7), 59.2 (C-8), 75.5 ($2CH_2$), 75.9 ($3CH_2$), 76.6 (C-2), 81.3 (C-1 and C-3), 83.2 (C-4 and C-6), 83.4 (C-5), 127.7 (3C-Ar), 127.8 (3C-Ar), 127.9 (4C-Ar), 128.0 (2C-Ar), 128.2 (4C-Ar), 128.6 (5C-Ar), 128.6 (4C-Ar), 138.2 (2Cq-Ar), 138.6 (3Cq-Ar); IR (NaCl, ν, cm^{-1}) 3448, 2927, 2875, 1065; HRMS ESI *m/z* : calculated for $C_{43}H_{46}NaO_7$ $[M+Na]^+$ 697.3136. Found: 697.3153.

1-*C*-(2-Hydroxyethyl)-2,3,4,5,6-pentakis-*O*-(phenylmethyl)-*scyllo*-inositol (***scyllo*-16**)

Scyllo-**16** was obtained as a white solid in 70% yield starting from *scyllo*-**14** using the above

protocol. R_f 0.28 (7/3 H/A); ^{1}H NMR (400 MHz, $CDCl_3$) δ 1.99 (t, 2H, $J_{7,8}$ = 5.0 Hz, H-7), 3.50-3.55 (m, 2H, H-inositol), 3.60-3.70 (m, 3H, H-inositol), 3.89 (t, 2H, H-8), 4.85-4.95 (m, 10H, CH_2Ph), 7.25-7.38 (m, 25H, H-Ar); ^{13}C NMR (62.9 MHz, $CDCl_3$) δ 34.3 (C-7), 58.3 (C-8), 75.9 ($2CH_2$), 76.1 (CH_2), 76.7 ($2CH_2$), 77.0 (C-1), 82.3 (C-3 and C-5), 84.2 (C-4), 85.6 (C-2 and C-6), 127.8 (C-Ar), 127.8 (2C-Ar), 127.9 (2C-Ar), 128.0 (4C-Ar), 128.1 (6C-Ar), 128.5 (6C-Ar), 128.7 (4C-Ar), 138.2 (2Cq-Ar), 138.4 (2Cq-Ar), 138.6 (Cq-Ar); IR (NaCl, ν, cm^{-1}) 3401, 3064, 2919, 1060; HRMS ESI *m/z* : calculated for $C_{43}H_{46}NaO_7$ $[M+Na]^+$ 697.3136. Found: 697.3150.

2-*C*-(3-Hydroxypropyl)-3,4,5,6-tetrakis-*O*-(phenylmethyl)-*myo*-inositol (*myo*-17)

To a solution of *myo*-**15** (10.0 g, 14.9 mmol) in dry THF (25 mL) was added dropwise BH_3 (60 mL of a 1 M solution in hexane, 60 mmol) under argon at 0 °C. The mixture was stirred for 8 h at room temperature, then H_2O_2 (30%, 500 mL) and a solution of 2 M NaOH (600 mL) were added slowly. After 12 h, the mixture was diluted with ethyl acetate (200 mL) and extracted. The remaining aqueous phase was further extracted with ethyl acetate (2 x 100 mL), the combined organic layers were dried over $MgSO_4$, filtered and the solvent removed under reduced pressure. The crude product was purified by flash column chromatography (silica gel, hexane 100% to hexane/EtOAc 70:30) to give *myo*-**17** (8.2 g, 80% yield) as a white solid. m.p. 121-122 °C; R_f 0.30 (7/3 Hex/EtOAc); ^{1}H NMR (400 MHz, $CDCl_3$) δ 1.15-1.20 (m, 2H, H-8), 1.71-1.78 (m, 2H, H-7), 2.35 (brs, 1H, OH), 3.29 (t, 2H, $J_{8,9}$ = 6.5 Hz, H-9), 3.37 (d, 2H, $J_{1,6}$ = $J_{3,4}$ = 9.5 Hz, H-1 and H-3), 3.54 (app t, 1H, $J_{4,5}$ = $J_{5,6}$ = 9.5 Hz, H-5), 4.08 (app t, 2H, H-4 and H-6), 4.68 (d, 2H, *J* = 11.0 Hz, CH_2Ph), 4.87 (d, 2H, *J* = 11.0 Hz, CH_2Ph), 4.95 (s, 2H, CH_2Ph), 4.97 (d, 2H, *J* = 11.0 Hz, CH_2Ph), 5.02 (d, 2H, *J* = 11.0 Hz, CH_2Ph), 7.25-7.40 (m, 25H, H-Ar); ^{13}C NMR (100.6 MHz, $CDCl_3$) δ 27.5 (C-8), 31.2 (C-7), 62.8 (C-9), 75.6 ($2CH_2$), 76.0 ($3CH_2$), 77.2 (C-2), 79.7 (C-1 and C-3), 83.4 (C-5), 83.5 (C-4 and C-6), 127.7 (C-Ar), 127.8 (2C-Ar), 127.8 (2C-Ar), 128.0 (6C-Ar), 128.4 (4C-Ar), 128.6

(10C-Ar), 138.4 (2Cq-Ar), 138.6 (2Cq-Ar), 138.7 (Cq-Ar); IR (NaCl, ν, cm^{-1}) 3435, 2926, 2868, 1065; ESI *m/z* : calculated for $C_{44}H_{48}O_7$ 689 . Found : 689 $[M]^+$, 711 $[M+Na]^+$Anal. Calcd for $C_{44}H_{48}O_7$: C, 76.71; H, 7.02 %. Found: C, 76.16; H, 7.68 %.

1-*C*-(2-Hydroxyethyl)-2,3,4,5,6-pentakis-*O*-(phenylmethyl)-*scyllo*-inositol (***scyllo*-17**)

Scyllo-**17** was obtained as a white solid in 70% yield starting from *scyllo*-**15** using the above protocol. m.p. 152-153 °C; R_f 0.2 (7/3 H/A); ^{1}H NMR (400 MHz, $CDCl_3$) δ 1.85 (t, 2H, $J_{7,8}$ = 6.5 Hz, H-7), 1.95 (app qt, 2H, J = 6.5 Hz, H-8), 3.48 (d, 2H, $J_{2,3}$ = $J_{5,6}$ = 9.0 Hz, H-2 and H-6), 3.57-3.67 (m, 5H, H-3, H-4, H-5 and H-9), 4.75-4.95 (m, 10H, CH_2Ph), 7.04-7.43 (m, 25H, H-Ar); ^{13}C NMR (100.6 MHz, $CDCl_3$) δ 26.8 (C-8), 29.0 (C-7), 64.0 (C-9), 76.0 (2CH_2), 76.1 (C-1), 76.2 (CH_2), 76.3 (2CH_2), 82.7 (C-3 and C-5), 84.1 (C-4), 86.3 (C-2 and C-6), 127.8 (2C-Ar), 127.8 (3C-Ar), 127.9 (4C-Ar), 128.0 (2C-Ar), 128.1 (4C-Ar), 128.5 (4C-Ar), 128.6 (6C-Ar), 138.6 (2Cq-Ar), 138.6 (4Cq-Ar), 138.8 (2Cq-Ar); IR (NaCl, ν, cm^{-1}) 3445, 2927, 2634, 1236; HRMS ESI *m/z* : calculated for $C_{44}H_{48}KO_7$ $[M+K]^+$ 711.3292. Found: 711.3295.

2-*C*-(Triphenylmethyloxyethyl)-1,3,4,5,6-pentaacetyl-*myo*-inositol (***myo*-18**)

To a solution of *myo*-**3** (680 mg, 2.9 mmol) in pyridine (20 mL) were added trityl chloride (2.5 g, 9 mmol) and a catalytic amount of DMAP. The mixture was stirred at 70 °C for 3 d then the reaction mixture was allowed to reach room temperature. Acetic anhydride (10 mL) was added and the solution was stirred for 2 h. The volatiles were removed under reduced pressure and the residue was purified by flash column chromatography (silica gel, hexane/EtOAc 80:20 to 60:40) to give *myo*-**18** (1.45 g, 74% yield). m.p. 187-188 °C; R_f 0.42 (6/4 H/A); ^{1}H NMR (400 MHz, $CDCl_3$) δ 1.88 (t, 2H, $J_{7,8}$ = 6.5 Hz, H-7), 1.95 (s, 6H, 2Me), 1.98 (s, 6H, 2Me), 2.00 (s, 3H, Me), 3.26 (t, 2H, H-8), 3.80 (s, 1H, OH), 4.96 (d, 2H, $J_{1,6}$ = $J_{3,4}$ = 9.5 Hz, H-1 and H-3), 5.17 (app t, 1H, $J_{4,5}$ = $J_{5,6}$ = 9.5 Hz, H-5), 5.54 (app t, 2H, H-4

and H-6), 7.29 (brt, 3H, *J* = 7.5 Hz, H-Ar), 7.33 (brt, 6H, *J* = 7.5 Hz, H-Ar), 7.38 (brd, 6H, H-Ar); ^{13}C NMR (100.6 MHz, $CDCl_3$) δ 20.6 (2Me), 20.7 (3Me), 34.5 (C-7), 60.1 (C-8), 70.7 (C-5), 70.9 (C-4 and C-6), 73.5 (C-1 and C-3), 74.3 (C-2), 88.0 (Cq-Tr), 127.4 (3C-Ar), 128.1 (6C-Ar), 128.5 (6C-Ar), 143.5 (3Cq-Ar), 169.8 (2 CO), 169.8 (2CO), 169.9 (CO); IR (KBr, ν, cm^{-1}) 3482, 2936, 2857, 1758, 1227, 1036; HRMS ESI *m/z* : calculated for $C_{37}H_{40}NaO_{12}$ $[M+Na]^+$ 699.2412. Found: 699.2441. Anal. Calculated for $C_{37}H_{40}O_{12}$: C, 65.67; H, 5.95%. Found: C, 65.75; H, 5.81%.

1-*C*-(Triphenylmetyloxyethyl)-2,3,4,5,6-penta-*O*-acetyl-*scyllo*-inositol (***scyllo*-18**)

Scyllo-**18** was obtained as a white solid in 70% yield starting from *scyllo*-**3** using the above protocol. m.p. 95-96 °C; R_f 0.51 (1/1 Hex/EtOAc); ^{1}H NMR (400 MHz, $CDCl_3$) δ 1.92 (s, 6H, 2Me), 2.00 (s, 3H, Me), 2.01 (6H, 2Me), 2.21 (t, 2H, $J_{7,8}$ = 6.5 Hz, H-7), 3.54 (t, 2H, H-8), 4.35 (s, 1H, OH) 5.13-5.25 (m, 5H, H-inositol), 7.23-7.44 (m, 15H, H-Ar); ^{13}C NMR (100.6 MHz, $CDCl_3$) δ 20.6 (2Me), 20.7 (3Me), 30.1 (C-7), 61.3 (C-8), 70.3 (C-3 and C-5), 71.5 (C-4), 74.3 (C-1), 74.6 (C-2 and C-6), 88.1 (Cq-Tr), 127.5 (3C-Ar), 128.2 (6C-Ar), 128.5 (6C-Ar), 143.5 (3Cq-Ar), 169.5 (2CO), 169.6 (CO), 170.0 (2CO); IR (film, ν, cm^{-1}) 3467, 2107, 1759, 1644, 1368, 1224; HRMS ESI *m/z* : calculated for $C_{37}H_{40}NaO_{12}$ $[M+Na]^+$ 699.2412. Found: 699.2400. Anal. Calculated for $C_{37}H_{40}O_{12}$: C, 65.67; H, 5.95%. Found: C, 63.70; H, 5.88%.

2-*C*-(2-Hydroxyethyl)-1,3,4,5,6-pentaacetyl-*myo*-inositol (***myo*-20**)

A solution of *myo*-**18** (100 mg, 0.15 mmol) and $FeCl_3$ (200 mg, 1.5 mmol) diluted in dry CH_2Cl_2 (5 mL) was stirred at room temperature for 15 min. The reaction mixture was quenched by adding water and extracted with CH_2Cl_2 (3 x 50 mL). The combined organic layers were dried over $MgSO_4$, filtered and the solvent was removed under reduced pressure. The crude product was purified by flash column chromatography (silica gel, hexane/EtOAc

80:20 to 20:80) to give *myo*-**20** (29 mg, 45% yield). R_f 0.25 (6/4 H/A); ^{1}H NMR (400 MHz, $CDCl_3$) δ 1.78 (t, 2H, $J_{7,8}$ = 6.0 Hz, H-7), 1.98 (s, 6H, 2Me), 1.99 (s, 3H, Me), 2.11 (s, 6H, 2Me), 3.73 (t, 2H, H-8), 5.16 (d, 2H, $J_{1,6}$ = $J_{3,4}$ = 10.0 Hz, H-1 and H-3), 5.23 (app t, 1H, $J_{5,4}$ = $J_{5,6}$ = 10.0 Hz, H-5), 5.54 (app t, 2H, H-4 and H-6); ^{13}C NMR (100.6 MHz, $CDCl_3$) δ 20.7 (3Me), 20.8 (2Me), 36.3 (C-7), 58.3 (C-8), 70.5 (C-5), 71.0 (C-4 and C-6), 72.9 (C-1 and C-3), 75.0 (C-2) 169.9 (3CO), 170.0 (2CO); IR (film, ν, cm^{-1}) 3403, 1729, 1370, 1227; ESI *m/z*: calculated for $C_{18}H_{26}NaO_{12}$ [M+Na]$^+$ 457. Found: 457

1-*C*-(2-Hydroxyethyl)-2,3,4,5,6-pentaacetyl-*scyllo*-inositol (***scyllo*-20**)

A solution of *scyllo*-**18** (100 mg, 0.15 mmol) and $FeCl_3$ (200 mg, 1.5 mmol) diluted in dry CH_2Cl_2 (5 mL) was stirred at room temperature for 18 h. The reaction mixture was quenched by adding water and extracted with CH_2Cl_2 (3 x 50 mL). The combined organic layers were dried over $MgSO_4$, filtered and the solvent was removed under reduced pressure. The crude product was purified by flash column chromatography (silica gel, hexane/EtOAc 80:20 to 20:80) to give *scyllo*-**20** (72% yield). R_f 0.20 (3/7 Hex/EtOAc); ^{1}H NMR (400 MHz, $CDCl_3$) δ 1.98 (s, 3H, Me), 2.01 (s, 6H, 2Me), 2.10 (s, 6H, 2Me), 2.02-2.12 (m, 2H, H-7), 2.8 (t, 1H, OH), 3.93, (s, 1H, OH), 3.98-4.04 (m, 2H, H-8), 5.20-5.25 (m, 5H, H-inositol); ^{13}C NMR (100.6 MHz, $CDCl_3$) δ 20.6 (3Me), 20.8 (2Me), 32.3 (C-7), 59.7 (C-8), 70.1 (C-3 and C-5), 71.6 (C-4), 74.9 (C-2 and C-6), 75.4 (C-1), 169.7 (CO), 169.9 (2CO), 170.2 (2CO); IR (film, ν, cm^{-1}) 3484, 2942, 1757, 1362, 1224; HRMS ESI *m/z* : calculated for $C_{18}H_{26}NaO_{12}$ [M+Na]$^+$ 457.1316. Found: 457.1319.

2-*C*-(2-Tosyloxyethyl)-1,3,4,5,6-pentaacetyl-*myo*-inositol (***myo*-22**)

A mixture of *myo*-**20** (100 mg, 0.21 mmol), tosyl chloride (107 mg, 0.63 mmol), triethylamine (0.2 mL, 0.03 mmol) in dry CH_2Cl_2 (3 mL) was stirred at room temperature for 3 h. The reaction was quenched by adding water and extracted with CH_2Cl_2 (3 x 50 mL). The

combined organic layers were dried over $MgSO_4$, filtered and the solvent was removed under reduced pressure. The crude product was purified by flash column chromatography (silica gel, hexane/EtOAc 80:20 to 20:80) to give *myo*-**22** (86 mg, 70% yield) as a white solid m.p. 88-89 °C; R_f 0.55 (6/4 H/A); ^{1}H NMR (400 MHz, $CDCl_3$) δ 1.96-2.00 (m, 2H, H-7), 1.97 (s, 6H, 2Me), 1.98 (s, 3H, Me), 2.09 (s, 6H, 2Me), 2.47 (s, 3H, MeTs), 3.98 (t, 2H, $J_{7,8}$ = 7.0 Hz, H-8), 4.90 (d, 2H, $J_{1,6}$ = $J_{3,4}$ = 9.5 Hz, H-1 and H-3), 5.12 (app t, 1H, $J_{4,5}$ = $J_{5,6}$ = 9.5 Hz, H-5), 5.48 (app t, 2H, H-4 and H-6), 7.39 (d, 2H, *J* = 8.0 Hz, H-Ar), 7.79 (d, 2H, *J* = 8.0 Hz, H-Ar); ^{13}C NMR (100.6 MHz, $CDCl_3$) δ 20.6 (5Me), 21.8 (MeTs), 34.5 (C-7), 65.1 (C-8), 70.3 (C-5), 70.8 (C-4 and C-6), 71.7 (C-1 and C-3), 74.3 (C-2), 128.2 (2C-Ar), 130.2 (2C-Ar), 132.6 (Cq-Ar), 145.4 (Cq-Ar), 169.7 (2CO), 169.8 (3CO); IR (KBr, ν, cm^{-1}) 3483, 2942, 1757, 1227; HRMS ESI *m/z* : calculated for $C_{25}H_{32}NaO_{14}S$ $[M+Na]^+$ 611.1405. Found: 611.1400.

1-*C*-(2-Tosyloxyethyl)-2,3,4,5,6-pentaacetyl-*scyllo*-inositol (***scyllo*-22**)

Scyllo-**22** was obtained as a white solid in 71% yield starting from *scyllo*-**20** using the above protocol. R_f 0.28 (1/1 Hex/EtOAc); ^{1}H NMR (250 MHz, $CDCl_3$) δ 1.96 (s, 3H, Me), 1.99 (s, 6H, 2Me), 2.04 (s, 6H, 2Me), 2.11 (t, 2H, $J_{7,8}$ = 7.5 Hz, H-7), 2.45 (Me Ts), 2.95 (brs, 1H, OH), 4.34 (t, 2H, H-8), 5.05-5.25 (m, 5H, H-inositol), 7.36 (d, 2H, *J* = 8.0 Hz ,H-Ar), 7.82 (d, 2H, H-Ar); ^{13}C NMR (62.9 MHz, $CDCl_3$) δ 20.5 (2Me), 20.6 (Me), 20.7 (2Me), 21.8 (Me-Ts), 30.3 (C-7), 66.4 (C-8), 70.7 (C-3 and C-5), 71.3 (C-1), 73.9 (C-4), 74.5 (C-2 and C-6), 128.1 (2C-Ar), 130.1 (2C-Ar), 133.3 (Cq-Ar), 145.1 (Cq-Ar), 169.7 (2CO), 169.7 (CO), 170.4 (2CO); IR (film, ν, cm^{-1}) 3485, 2945, 1760, 1368, 1222; HRMS ESI *m/z* : calculated for $C_{25}H_{32}KO_{14}S$ $[M+K]^+$ 627.1144. Found: 627.1146.

2-*C*-(2-Tosyloxyethyl)-1,2,3,4,5,6-hexaacetyl-*myo*-inositol (***myo*-23**)

To a solution of *myo*-**22** (200 mg, 0.34 mmol) in isopropenyl acetate (100 mL) was added *p*-TSA (100 mg, 0.58 mmol) and the mixture was stirred for 2 h at 80 °C. The solvent was

removed under reduced pressure, and the residue was diluted and extracted in CH_2Cl_2 (3 x 50 mL). The combined organic layers were dried over $MgSO_4$, filtered and the solvent was removed under reduced pressure. The crude product was purified by flash chromatography (silica gel, hexane/EtOAc 80:20 to 40:60) to give *myo*-**23** (170 mg, 78% yield) as a white solid. m.p. 110-111 °C; R_f 0.31 (6/4 H/A); ^{1}H NMR (400 MHz, $CDCl_3$) δ 1.96 (s, 6H, 2Me), 1.97 (s, 3H, Me), 2.05 (s, 6H, 2Me), 2.17 (s, 3H, Me), 2.45 (s, 3H, MeTs), 2.64 (t, 2H, $J_{7,8}$ = 7.0 Hz , H-7), 3.98 (t, 2H, H-8), 4.97 (d, 2H, $J_{1,6}$ = $J_{3,4}$ = 10.0 Hz, H-1 and H-3), 5.10 (appt, 1H, $J_{4,5}$ = $J_{5,6}$ = 10.0 Hz, H-5), 5.39 (app t, 2H, H-4 and H-6), 7.36 (d, 2H, J = 8.0 Hz, H-Ar), 7.78 (d, 2H, J = 8.0 Hz, H-Ar); ^{13}C NMR (100.6 MHz, $CDCl_3$) δ 20.6 (5 Me), 21.8 (Me), 22.6 (MeTs), 31.0 (C-7), 65.0 (C-8), 70.3 (C-4 and C-6), 70.4 (C-5), 71.2 (C-1 and C-3), 83.7 (C-2), 128.2 (2C-Ar), 130.1 (2C-Ar), 132.7 (Cq-Ar), 145.2 (Cq-Ar), 168.7 (CO), 169.6 (2CO), 169.7 (3CO); IR (NaCl, ν, cm^{-1}) 2928, 1760, 1369, 1178; HRMS ESI *m/z* : calculated for $C_{27}H_{34}NaO_{15}S$ [M+Na]$^+$ 653.1511. Found : 653.1527Anal. Calcd for $C_{27}H_{34}O_{15}S$: C, 51.42; H, 5.43; X, S: 5.08. Found: C, 51.49; H, 5.50; X, 1.69.

1-*C*-(2-Tosyloxyethyl)-1,2,3,4,5,6-hexaacetyl-*scyllo*-inositol (***scyllo*-23**)

Scyllo-**23** was obtained as a white solid in quantitative yield starting from *scyllo*-**22** using the above protocol. m.p. 102-104 °C; R_f 0.36 (1/1 Hex/EtOAc); ^{1}H NMR (400 MHz, $CDCl_3$) δ 1.90 (s, 3H, CH3), 1.98 (s, 3H, CH3), 1.99 (s, 6H, 2 CH3), 2.02 (s, 6H, 2 CH3), 2.31 (t, 2H, $J_{7,8}$ = 7.5 Hz, H-7), 2.46 (s, 3H, CH3-Ts), 4.29 (t, 2H, $J_{7,8}$ = 7.5 Hz, H-8), 5.11 (t, 2H, $J_{2,3}$= $J_{3,4}$= $J_{4,5}$= $J_{5,6}$= 10.0 Hz, H-3 and H-5), 5.26 (t, 1H, $J_{3,4}$= $J_{4,5}$= 10.0 Hz, H-4), 6.15 (d, 2H, $J_{2,3}$= $J_{5,6}$= 10.0 Hz, H-2 and H-6), 7.39 (d, 2H, J = 8.5 Hz, H-Ts), 7.82 (d, 2H, J = 8.5 Hz, H-Ts); ^{13}C NMR (100.6 MHz, $CDCl_3$) δ 20.6 (5Me), 21.8 (Me), 21.9 (Me-Ts), 30.3 (C-7), 65.5 (C-8), 70.1 (C-3 and C-5), 70.3 (C-2 and C-6), 70.6 (C-4), 81.6 (C-1), 128.1 (2C-Ar), 130.1 (2C-Ar), 133.3 (Cq-Ar), 145.2 (Cq-Ar), 168.8 (2CO), 169.4 (CO), 169.5 (CO), 169.7 (2CO);

IR (film, ν, cm^{-1}) 2946, 1763, 1432, 1368, 1221; HRMS ESI *m/z* : calculated for $C_{27}H_{34}NaO_{15}S$ $[M+Na]^+$ 653.1511. Found: 653.1530.

2-*C*-(2-Fluoroethyl)-1,3,4,5,6-pentaacetyl-*myo*-inositol (***myo*-25**)

To a solution of *myo*-**22** (70 mg, 0.044 mmol) in dry CH_3CN (8 mL) was added KF (220 mg, 0.44 mmol), and [18]-crown-6 (300 mg, 0.50 mmol). The mixture was stirred at room temperature for 2 h. Afterwards the residue was diluted with CH_2Cl_2, washed with water, dried over $MgSO_4$ and filtered. After the solvent was removed under reduced pressure the crude product was purified by column chromatography (silica gel, hexane/EtOAc 60:40) to give *myo*-**25** (45 mg, 70% yield). R_f 0.36 (4/6 H/A); ^{1}H NMR (400 MHz, $CDCl_3$) δ 1.94 (dt, 2H, $J_{H,F}$ = 28.0 Hz et $J_{7,8}$ = 5.5 Hz, H-7), 1.99 (s, 6H, 2Me), 2.00 (s, 3H, Me), 2.10 (s, 6H, 2Me), 2.51 (brs, 1H, OH), 4.50 (dt, 2H, $J_{H,F}$ = 47.0 Hz, H-8), 5.13 (d, 2H, $J_{1,6}$ = $J_{3,4}$ = 10.0 Hz, H-1 and H-3), 5.25 (app t, 1H, $J_{4,5}$ = $J_{5,6}$ = 10.0 Hz, H-5), 5.53 (app t, 2H, H-4 and H-6); ^{13}C NMR (100.6 MHz, $CDCl_3$) δ 20.7 (5Me), 35.6 (d, $J_{C,F}$ = 20.0 Hz, C-7), 70.3 (C-5), 71.0 (C-4 and C-6), 72.3 (C-1 and C-3), 74.6 (d, $J_{C,F}$ = 1.5 Hz, C-2), 79.3 (d, $J_{C,F}$ = 165 Hz, C-8), 169.5 (2CO), 169.8 (CO), 169.9 (2CO); NMR (^{19}F, 235 MHz $CDCl_3$) δ -216.3 (tt, $J_{H,F}$ =.48.0 Hz, $J_{H,F}$ = 28.0 Hz); IR (film, ν, cm^{-1}) 3403, 2955, 1729, 1370, 1227, 1036; HRMS ESI *m/z* : calculated for $C_{18}H_{25}FNaO_{11}$ $[M+Na]^+$ 459.1273. Found: 459.1300.

2-*C*-(3-Triphenylmethyloxypropyl)-1,3,4,5,6-pentaacetyl-*myo*-inositol (***myo*-26**)

To a solution of *myo*-**4** (700 mg, 2.9 mmol) in pyridine (20 mL) were added trityl chloride (2.5 g, 9 mmol) and a catalytic amount of DMAP. The mixture was stirred at room temperature for 3 d and then the reaction mixture was allowed to reach room temperature. Acetic anhydride (10 mL) was added and the solution was stirred for further 2 h. The volatiles were removed under reduced pressure and the residue was purified by flash column chromatography (silica gel, hexane/EtOAc 80:20 to 60:40) to give *myo*-**26** (1.3 g, 67% yield)

as a white solid. m.p. 136-137 °C; R_f 0.50 (6/4 H/A); ^{1}H NMR (400 MHz, $CDCl_3$) δ 1.55-1.70 (m, 4H, H-8 et H-7), 2.01 (2Me), 2.02 (Me), 2.07 (2Me), 2.43 (brs, 1H, OH), 3.01 (t, 2H, $J_{8,9}$ = 5.0 Hz, H-9), 5.19 (d, 2H, $J_{1,6}$ = $J_{3,4}$ = 10.0 Hz, H-1 and H-3), 5.25 (app t, 1H, $J_{4,5}$ = $J_{5,6}$ = 10.0 Hz, H-5), 5.59 (app t, 2H, H-4 and H-6), 7.24 (brt, 3H, J = 7.5 Hz, H-Ar), 7.30 (brt, 6H, J = 7.5 Hz, H-Ar), 7.39 (brd, 6H, H-Ar); ^{13}C NMR (100.6 MHz, $CDCl_3$) δ 20.7 (5Me), 23.8 (C-7), 32.1 (C-8), 63.2 (C-9), 70.6 (C-5), 71.0 (C-1 and C-3), 71.3 (C-4 and C-6), 75.8 (C-2), 86.7 (Cq-Tr), 127.1 (3C-Ar), 127.9 (6 C-Ar), 128.8 (6C-Ar), 144.2 (3 Cq-Ar), 169.5 (2CO), 169.9 (CO), 170.0 (2 CO); IR (KBr, ν, cm^{-1}) 3471, 1756, 1445, 1230, 1033; HRMS ESI *m/z* : calculated for $C_{38}H_{42}NaO_{12}$ $[M+Na]^+$ 713.2568. Found: 713.2654.

1-*C*-(3-Triphenylmethyloxypropyl)-2,3,4,5,6-pentaacetyl-*scyllo*-inositol (***scyllo*-26**)

Scyllo-**26** was obtained as a white solid in 70% yield starting from *scyllo*-**4** using the above protocol. m.p. 128-130 °C; R_f 0.53 (6/4 Hex/EtOAc); ^{1}H NMR (400 MHz, $CDCl_3$) δ 1.87-1.94 (m, 2H, H-7), 1.95-2.03 (m, 2H, H-8), 2.00 (s, 3H, Me), 2.05 (s, 6H, 2Me), 2.08 (s, 6H, 2Me), 3.15 (t, 2H, $J_{8,9}$ = 5.5 Hz, H-9), 3.40 (brs, 1H, OH)5.25-5.30 (m, 5H, H-inositol), 7.21 (m, 15H, H-Ar); ^{13}C NMR (100.6 MHz, $CDCl_3$) δ 20.5 (2Me), 20.6 (Me), 20.7 (2Me), 23.9 (C-8), 27.7 (C-7), 64.1 (C-9), 70.3 (C-3 and C-5), 71.6 (C-4), 74.0 (C-1), 75.0 (C-2 and C-6), 86.8 (Cq-Tr), 127.0 (3C-Ar), 127.9 (6C-Ar), 128.7 (6C-Ar), 144.3 (3Cq-Ar), 169.7 (CO), 169.8 (2CO), 170.3 (2CO); IR (film, ν, cm^{-1}) 3489, 2943, 1759, 1367, 1224, 1035; HRMS ESI *m/z* : calculated for $C_{38}H_{42}NaO_{12}$ $[M+Na]^+$ 713.2568. Found: 713.2582.

2-*C*-(3-Triphenylmetylloxypropyl)-1,2,3,4,5,6-hexaacetyl-*myo*-inositol (***myo*-27**)

To a solution of *myo*-**26** (1.0 g, 1.45 mmol) in isopropenyl acetate (200 mL) was added *p*-TSA (200 mg, 1.16 mmol) and the mixture was stirred for 2 h at 80 °C. Afterwards, the solvent was removed under reduced pressure, and the residue was diluted and extracted with CH_2Cl_2 (3 x 50 mL). The combined organic layers were dried over $MgSO_4$, filtered and the

solvent was removed under reduced pressure. The crude product was purified by flash chromatography (silica gel, hexane/EtOAc 80:20 to 40:60) to give *myo*-**27**(595 mg, 56% yield) as a white solid. m.p. 121-122 °C; R_f 0.55 (6/4 H/A); ^{1}H NMR (400 MHz, $CDCl_3$) δ 1.56-1.66 (m, 2H, H-8), 2.01 (s, 6H, 2Me), 2.03 (s, 3H, Me), 2.05 (s, 6H, 2Me), 2.33-2.42 (m, 2H, H-7), 2.99 (t, 2H, $J_{8,9}$ = 6.0 Hz, H-9), 5.23 (app t, 1H, $J_{4,5}$ = $J_{5,6}$ = 10.0 Hz, H-5), 5.26 (d, 2H, $J_{1,6}$ = $J_{3,4}$ = 10.0 Hz, H-1 and H-3), 5.49 (app t, 2H, H-4 and H-6), 7.23 (bt, 3H, J = 7.5 Hz, H-Ar), 7.30 (brt, 6H, J = 7.5 Hz, H-Ar), 7.39 (brd, 6H, H-Ar); ^{13}C NMR (100.6 MHz, $CDCl_3$) δ 20.6 (3Me), 20.6 (2Me), 22.9 (Me), 24.3 (C-8), 27.9 (C-7), 63.4 (C-9), 70.5 (C-1 and C-3), 70.7 (C-5), 70.8 (C-4 and C-6), 77.5 (C-2) 86.6 (Cq-Tr), 127.0 (3C-Ar), 127.8 (6C-Ar), 128.9 (6C-Ar), 144.3 (3Cq-Ar), 168.7 (CO), 169.5 (2CO), 169.8 (CO), 169.9 (2CO); IR (NaCl, ν, cm^{-1}) 3050, 1756, 1368, 1224; HRMS ESI *m/z* : calculated for $C_{40}H_{44}NaO_{13}$ $[M+Na]^+$ 755.2674. Found: 755.2671.

1-*C*-(3-Triphenylmetylloxypropyl)-1,2,3,4,5,6-hexaacetyl-*scyllo*-inositol (***scyllo*-27**)

Scyllo-**27** was obtained as a white solid in 52% yield starting from *scyllo*-**26** using the above protocol. m.p. 158-159 °C; R_f 0.60 (6/4 Hex/EtOAc); ^{1}H NMR (400 MHz, $CDCl_3$) δ 1.73-1.85 (m, 2H, H-8), 1.96 (s, 3H, Me), 1.99-2.04 (m, 2H, H-7), 1.99 (s, 3H, Me), 2.00 (s, 6H, 2Me), 2.03 (s, 6H, 2Me), 3.08 (t, 2H, $J_{8,9}$ = 6.5 Hz, H-9), 5.23 (app t, 2H, $J_{2,3}$ = $J_{3,4}$ = $J_{4,5}$ = $J_{5,6}$ = 10.0 Hz, H-3 and H-5), 5.34 (app t, 1H, H-4), 6.05 (d, 2H, H-2 and H-6), 7.24 (tt, 3H, J = 7.5 Hz, J = 2.0 Hz, H-Ar), 7.31 (brt, 6H, J = 7.5 H, H-Ar), 7.44 (brd, 6H, H-Ar); ^{13}C NMR (100.6 MHz, $CDCl_3$) δ 20.7 (3Me), 20.7 (2Me), 22.0 (Me), 24.2 (C-8), 27.3 (C-7), 63.6 (C-9), 70.8 (C-4), 70.9 (C-3 and C-5), 71.3 (C-2 and C-6), 82.7 (C-1), 86.6 (Cq-Tr), 127.1 (3C-Ar), 127.9 (6C-Ar), 128.8 (6C-Ar), 144.3 (3Cq-Ar), 169.1 (2CO), 169.6 (CO), 169.7 (CO), 169.8 (2CO); IR (KBr, ν, cm^{-1}) 2947, 1761, 1368, 1223; HRMS ESI *m/z* : calculated for $C_{40}H_{44}NaO_{13}$ $[M+Na]^+$ 755.2674. Found: 755.2674.

2-*C*-(3-Hydroxypropyl)-1,2,3,4,5,6-hexaacetyl-*myo*-inositol (*myo*-28)

A solution of *myo*-**27** (500 mg, 0.68 mmol) and $FeCl_3$ (1.01 g, 6.8 mmol) in dry CH_2Cl_2 (10 mL) was stirred at room temperature overnight. The reaction was quenched by adding water and extracted with CH_2Cl_2 (3 x 100 mL). The combined organic layers were dried over $MgSO_4$, filtered and the solvent was removed under reduced pressure. The crude product was purified by flash column chromatography (silica gel, hexane/EtOAc 80:20 to 20:80) to give *myo*-**28** (210 mg, 63% yield) as a white solid. m.p. 212-213 °C; R_f 0.2 (6/4 H/A); ^{1}H NMR (400 MHz, $CDCl_3$) δ 1.43-1.53 (m, 2H, H-8), 1.96 (s, 6H, 2Me), 1.97 (s, 3H, Me), 2.05 (s, 6H, 2Me), 2.16 (s, 3H, Me), 2.26-2.33 (m, 2H, H-7), 3.49 (t, 2H, $J_{8,9}$ = 6.0 Hz, H-9), 5.25 (app t, 1H, $J_{4,5}$ = $J_{5,6}$ = 10.0 Hz, H-5), 5.27 (d, 2H, $J_{1,6}$ = $J_{3,4}$ = 10.0 Hz, H-1 and H-3), 5.44 (app t, 2H, H-4 and H-6); ^{13}C NMR (100.6 MHz, $CDCl_3$) δ 20.6 (3 Me), 20.6 (2Me), 22.8 (Me), 26.8 (C-8), 27.2 (C-7), 62.2 (C-9), 70.6 (C-5), 70.6 (C-1 and C-3), 70.7 (C-4 and C-6), 86.5 (C-2), 168.8 (CO), 169.8 (2CO), 169.9 (CO), 170.0 (2CO); IR (KBr, ν, cm^{-1}) 3483, 2962, 1754, 1370, 1224; HRMS ESI *m/z* : calculated for $C_{21}H_{30}NaO_{13}$ $[M+Na]^+$ 513.1579. Found: 513.1586.

1-*C*-(3-Hydroxypropyl)-1,2,3,4,5,6-hexaacetyl-*scyllo*-inositol (*scyllo*-28)

Scyllo-**28** was obtained as a white solid in 70% yield starting from *scyllo*-**27** using the above protocol. m.p. 147-149 °C; R_f 0.30 (4/6 Hex/EtOAc); ^{1}H NMR (400 MHz, $CDCl_3$) δ 158-1.78 (m, 2H, H-8), 1.97 (s, 3H, Me), 1.99 (s, 3H, Me), 2.00 (s, 6H, 2Me), 2.06 (s, 6H, 2Me), 2.03-2.13 (m, 2H, H-7), 3.65 (t, 2H, $J_{8,9}$ = 5.5 Hz, H-9), 5.26 (app t, 2H, $J_{2,3}$ = $J_{3,4}$ = $J_{4,5}$ = $J_{5,6}$ = 9.0 Hz, H-3 and H-5), 5.31 (app t, 1H, H-4), 6.00 (d, 2H, H-2 and H-6); ^{13}C NMR (100.6 MHz, $CDCl_3$) δ 20.7 (3Me), 20.7 (2Me), 22.0 (Me), 26.5 (C-8), 26.9 (C-7), 62.9 (C-9), 70.9 (C-3 and C-5), 70.9 (C-4), 71.4 (C-2 and C-6), 82.8 (C-1), 169.1 (2CO), 169.7 (CO), 169.7 (CO), 169.9 (2CO); IR (film, ν, cm^{-1}) 3490, 2948, 1760, 1369, 1224; HRMS-ESI *m/z* :

calculated for $C_{21}H_{30}NaO_{13}$ $[M+Na]^+$ 513.1579. Found: 513.1582.

2-*C*-(3-Fluoropropyl)-1,2,3,4,5,6-hexaacetyl-*myo*-inositol (***myo*-29**)

To a solution of *myo*-**28** (50 mg, 0.1 mmol) in dry CH_2Cl_2 (8 mL) was added DAST (0.03 mL, 0.24 mmol). The mixture was stirred at room temperature for 5 min, then quenched by adding MeOH and diluted in CH_2Cl_2 (20 mL). The solution was washed with a saturated aqueous solution of sodium hydrogencarbonate and water. The organic phase was dried over $MgSO_4$, filtered, and the solvent was removed under reduced pressure. The crude product was purified by flash column chromatography (silica gel, hexane/EtOAc 80:20 to 20:80) to give *myo*-**29** (48 mg, 95% yield) as a white solid. m.p. 209-210 °C; R_f 0.50 (4/6 Hex/EtOAc); ^1H NMR (400 MHz, $CDCl_3$) δ 1.59-1.74 (m, 2H, H-8), 1.99 (s, 6H, 2Me), 2.00 (s, 3H, Me), 2.07 (s, 6H, 2Me), 2.19 (s, 3H, Me), 2.33-2.37 (m, 2H, H-7), 4.33 (dt, 2H, $J_{H,F}$ = 47.0 Hz et $J_{8,9}$ = 5.5 Hz, H-9), 5.18 (d, 2H, $J_{1,6}$ = $J_{3,4}$ = 10.0 Hz, H-1 and H-3), 5.20 (app t, 1H, $J_{4,5}$ = $J_{5,6}$ = 10.0 Hz, H-5), 5.47 (app t, 2H, H-4 and H-6); ^{13}C NMR (62.9 MHz, $CDCl_3$) δ 20.5 (2Me), 20.6 (3Me), 22.8 (Me), 24.7 (d, J = 18.0 Hz, C-8), 26.9 (d, $J_{C,F}$ = 5.0 Hz, C-7), 70.6 (C-1 and C-3), 70.6 (C-4 and C-6), 70.7(C-5), 83.4 (d, $J_{C,F}$ = 167.0 Hz, C-9), 86.1 (C-2), 168.8 (CO), 169.7 (2CO), 169.8 (3CO); NMR (^{19}F, 235 MHz $CDCl_3$) δ -219.7 (tt, $J_{H,F}$ = 47.0 Hz and $J_{H,F}$ = 21.0 Hz); IR (NaCl, ν, cm^{-1}) 2923, 1754, 1370, 1223, 1041; HRMS ESI *m/z* : calculated for $C_{21}H_{29}FNaO_{12}$ $[M+Na]^+$ 515.1535. Found: 515.1546.

1-*C*-(3-Fluoropropyl)-1,2,3,4,5,6-hexaacetyl-*scyllo*-inositol (***scyllo*-29**)

Scyllo-**29** was obtained as a white solid in 91% yield starting from *scyllo*-**28** using the above protocol. R_f 0.25 (6/4 Hex/EtOAc); ^1H NMR (400 MHz, $CDCl_3$) δ 1.77-1.94 (m, 2H, H-8), 1.96 (s, 3H, Me), 1.97 (s, 3H, Me), 1.99 (s, 6H, 2Me), 2.05 (s, 6H, 2Me), 2.09-2.15 (m, 2H, H-7), 4.43 (dt, 2H, $J_{H,F}$ = 47.0 Hz and $J_{8,9}$ = 6.0 Hz, H-9), 5.23 (app t, 2H, $J_{2,3}$ = $J_{3,4}$ = $J_{4,5}$ = $J_{5,6}$ = 9.5 Hz, H-3 and H-5), 5.32 (app t, 1H, H-4), 6.02 (d, 2H, H-2 and H-6); ^{13}C NMR

(100.6 MHz, $CDCl_3$) δ 20.6 (3Me), 20.7 (2Me), 22.0 (Me), 24.7 (d, $J_{C,F}$ = 20.0 Hz, C-8), 26.5 (d, $J_{C,F}$ = 5.0 Hz, C-7), 70.8 (C-3, C-4 and C-5), 71.2 (C-2 and C-6), 82.5 (C-1), 83.8 (d, $J_{C,F}$ = 166.0 Hz, C-9), 169.0 (2CO), 169.5 (CO), 169.7 (CO), 169.8 (2CO); NMR (^{19}F, 235 MHz $CDCl_3$) δ -219.1 (tt, $J_{H,F}$ = 47.0 Hz and $J_{H,F}$ = 25.0 Hz); IR (film, ν, cm^{-1}) 2963, 1760, 1369, 1224, 1036; HRMS-ESI *m/z* : calculated for $C_{21}H_{29}FNaO_{12}$ $[M+Na]^+$ 515.1535. Found: 515.1515.

myo−**1**

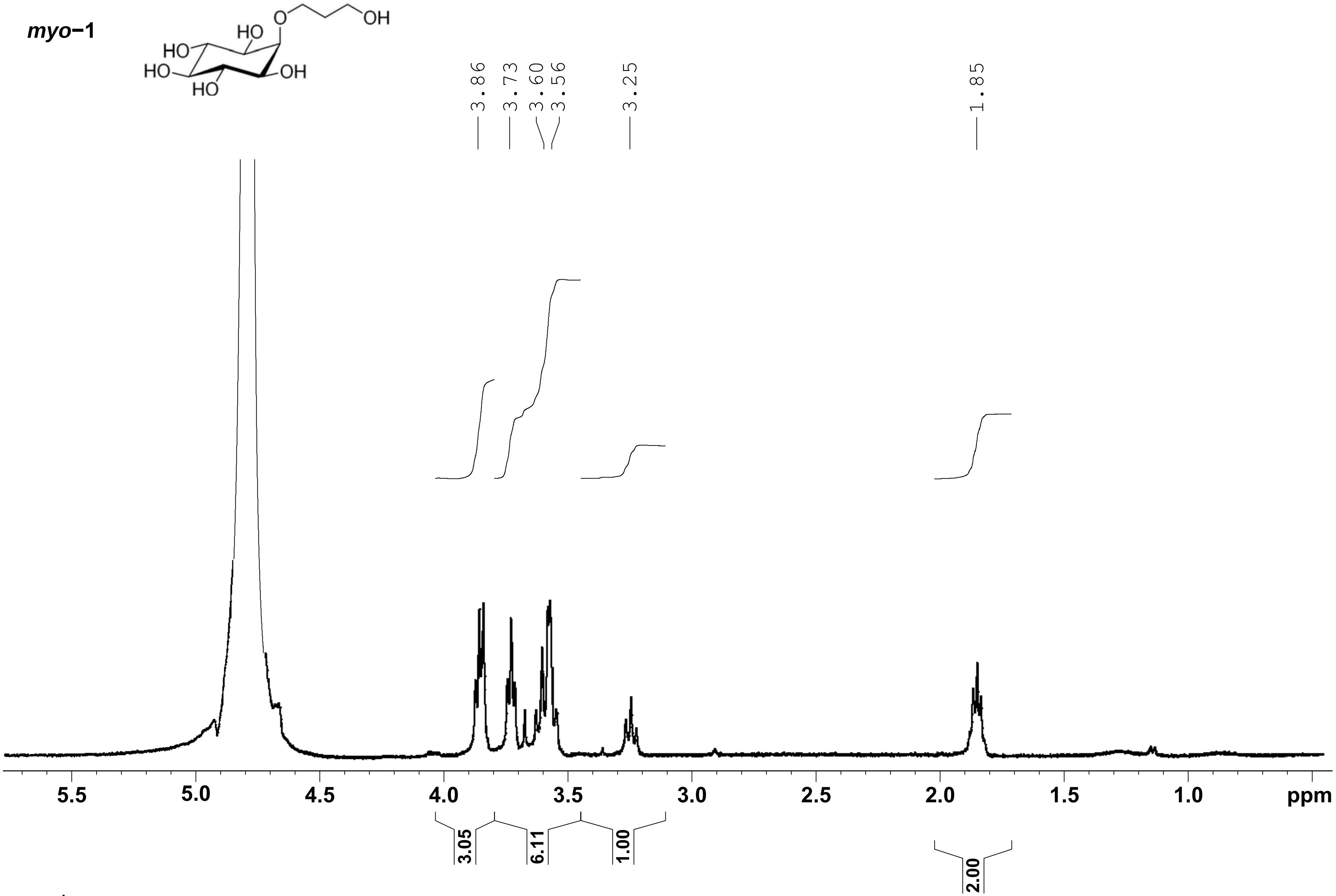

Figure 1: ^{1}H NMR spectrum

***myo*−1**

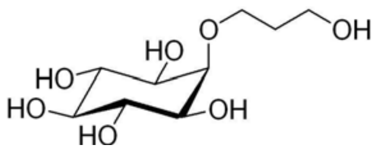

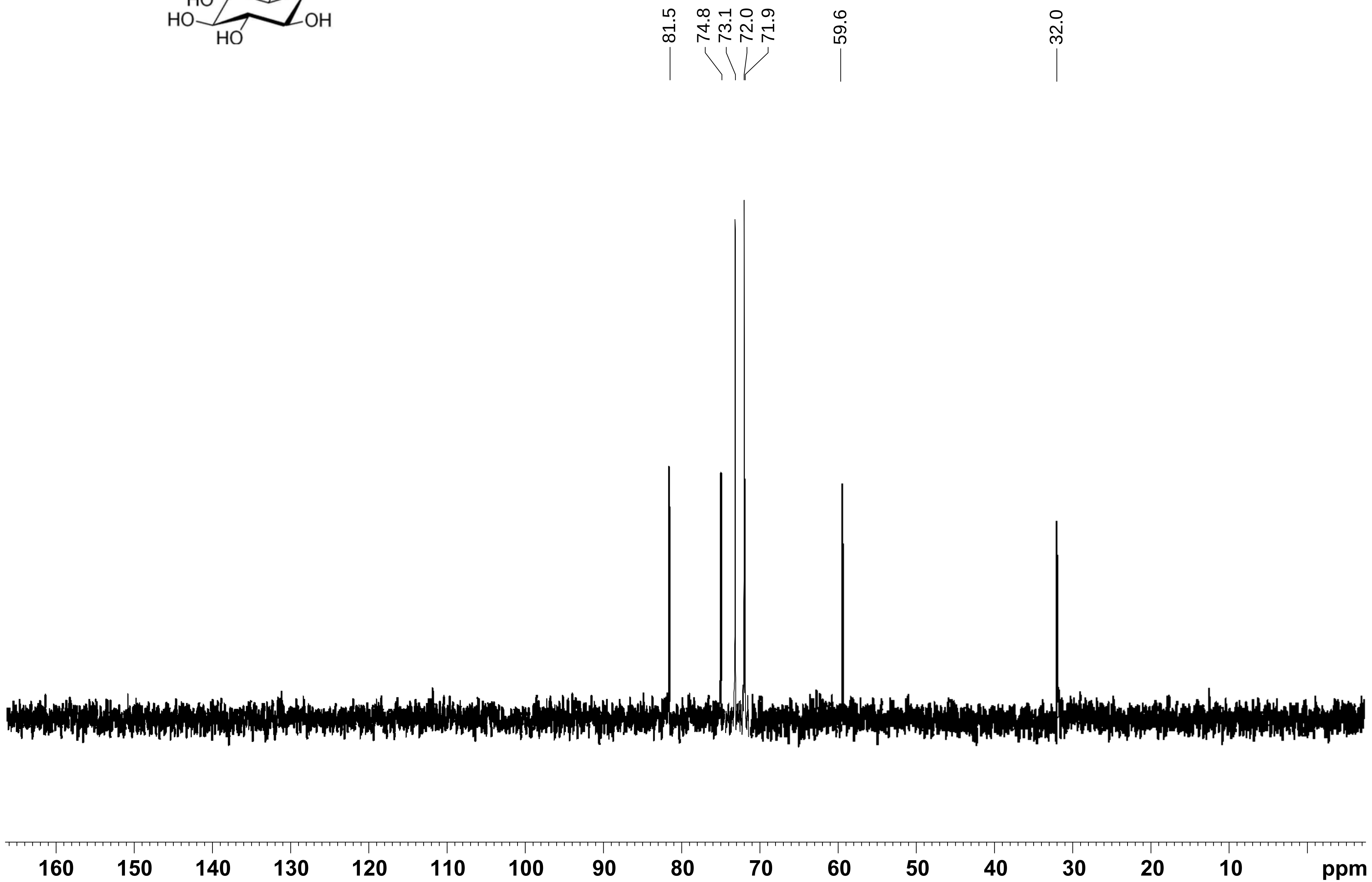

Figure 2: ^{13}C NMR spectrum

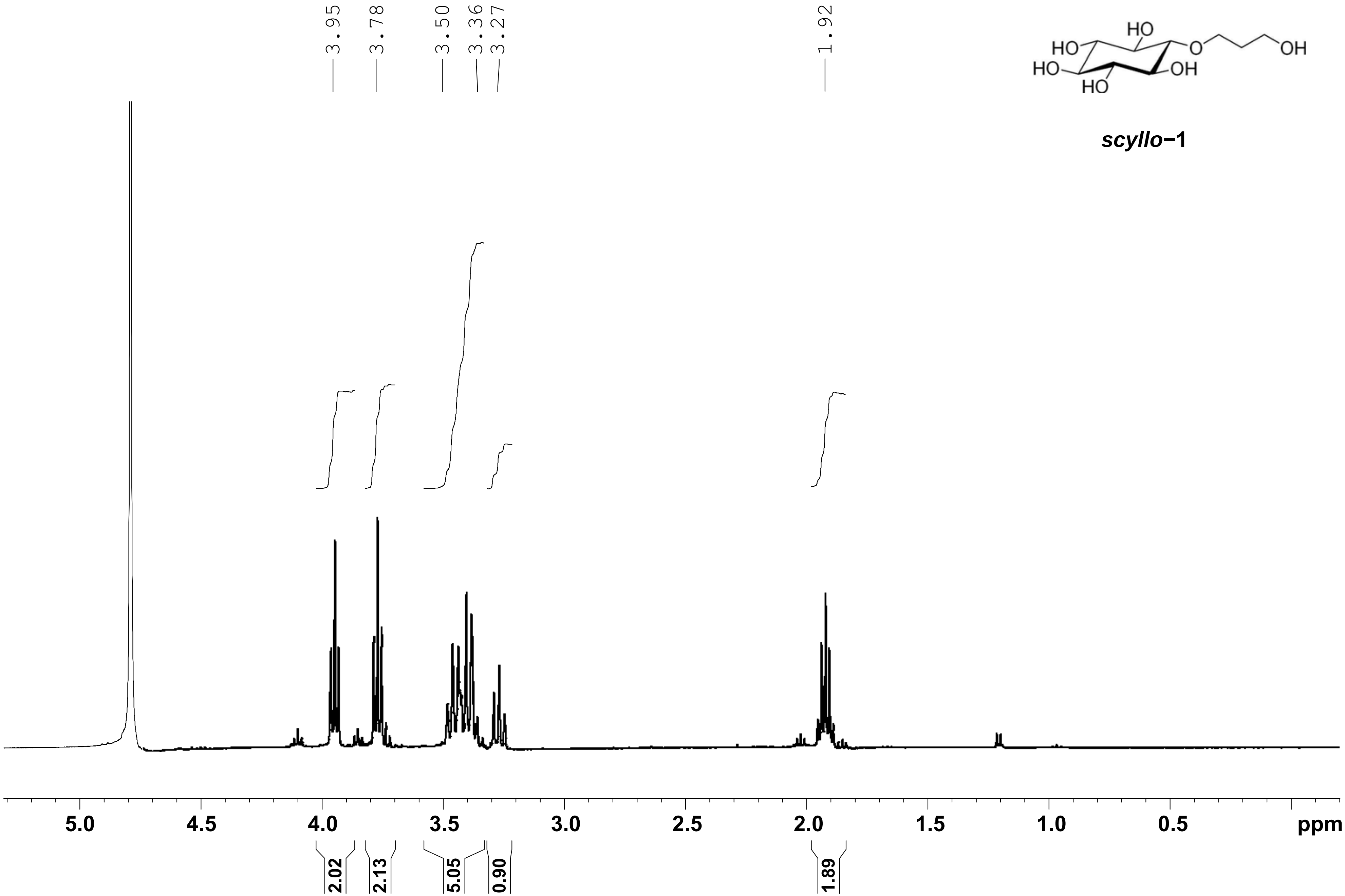

Figure 3: ^{1}H NMR spectrum

***scyllo*-1**

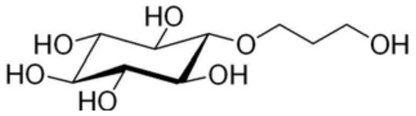

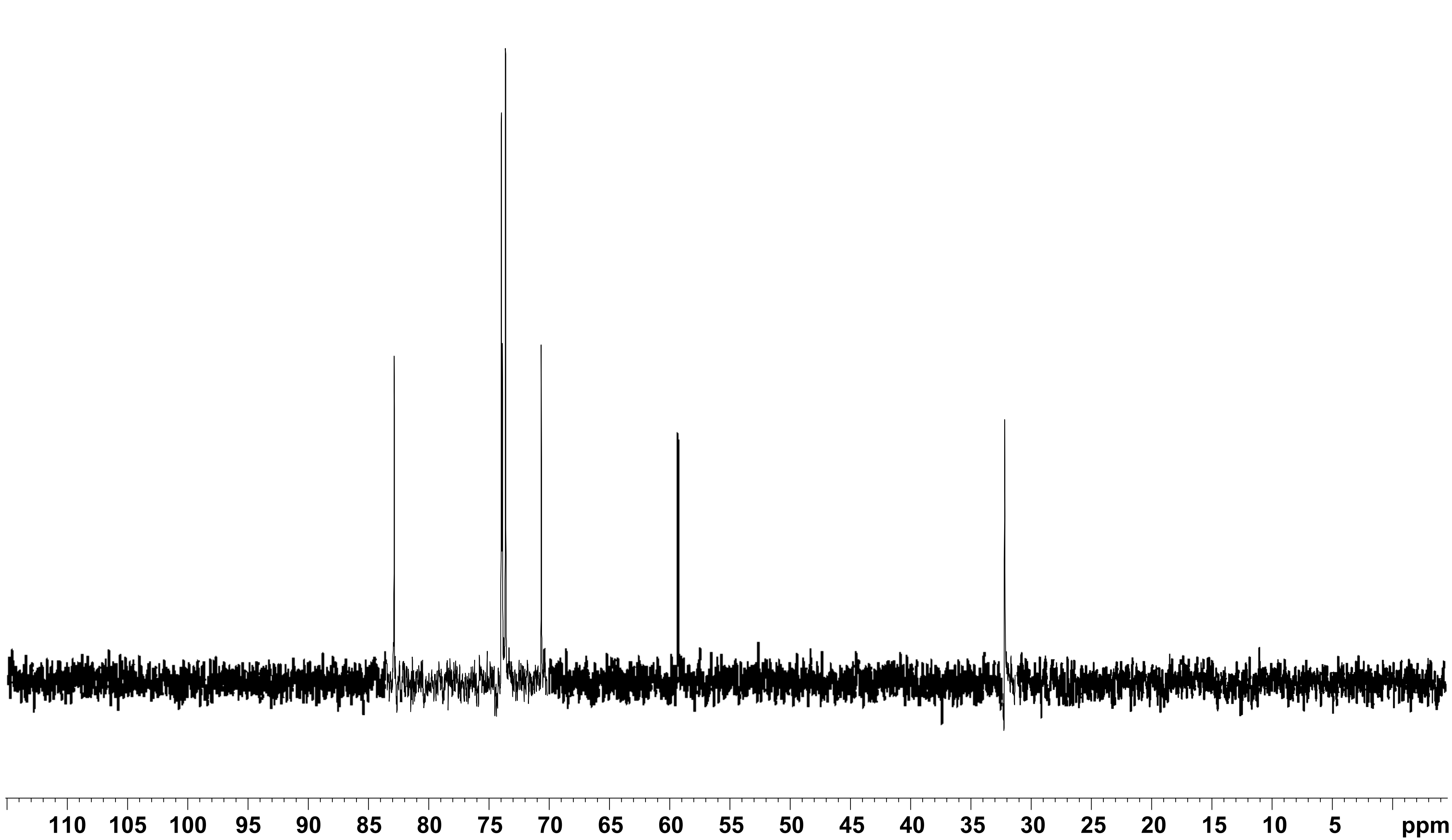

Figure 4: ^{13}C NMR spectrum

scyllo−2

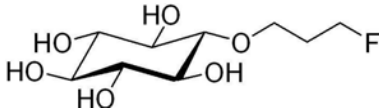

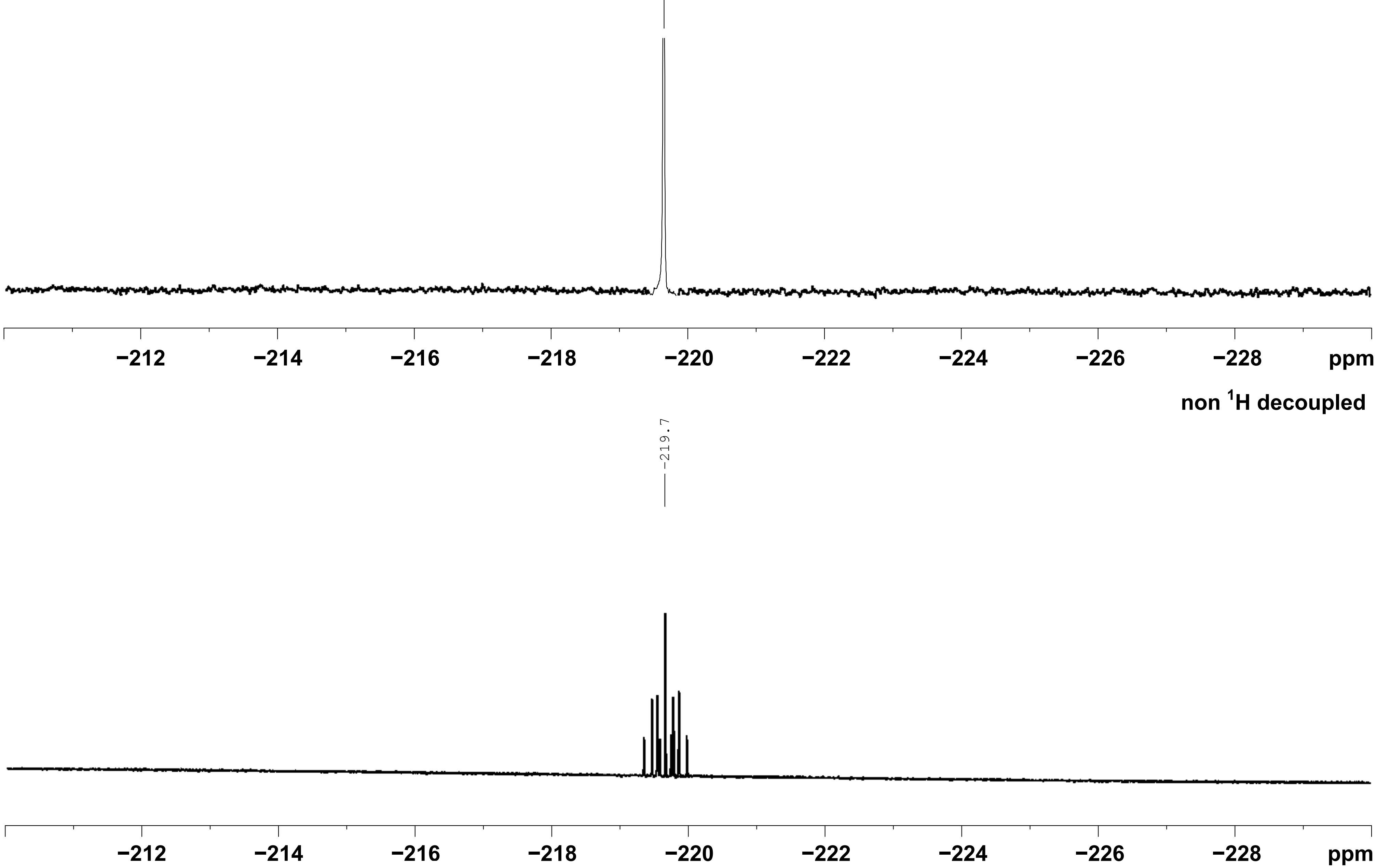

Figure 5: ^{19}F NMR spectrum

myo−2

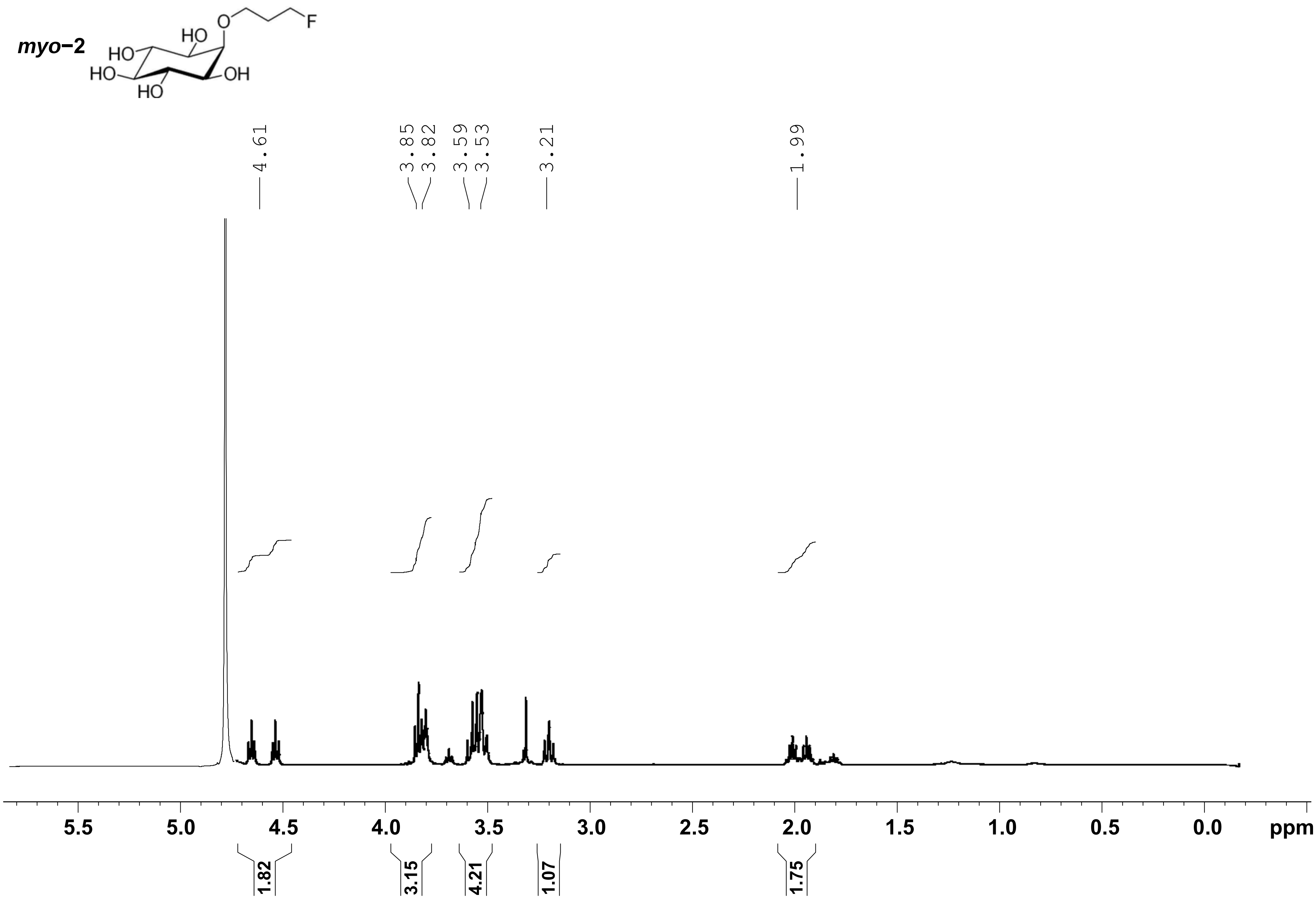

Figure 6: ^{1}H NMR spectrum

***myo*−2**

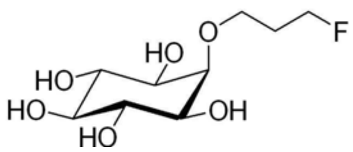

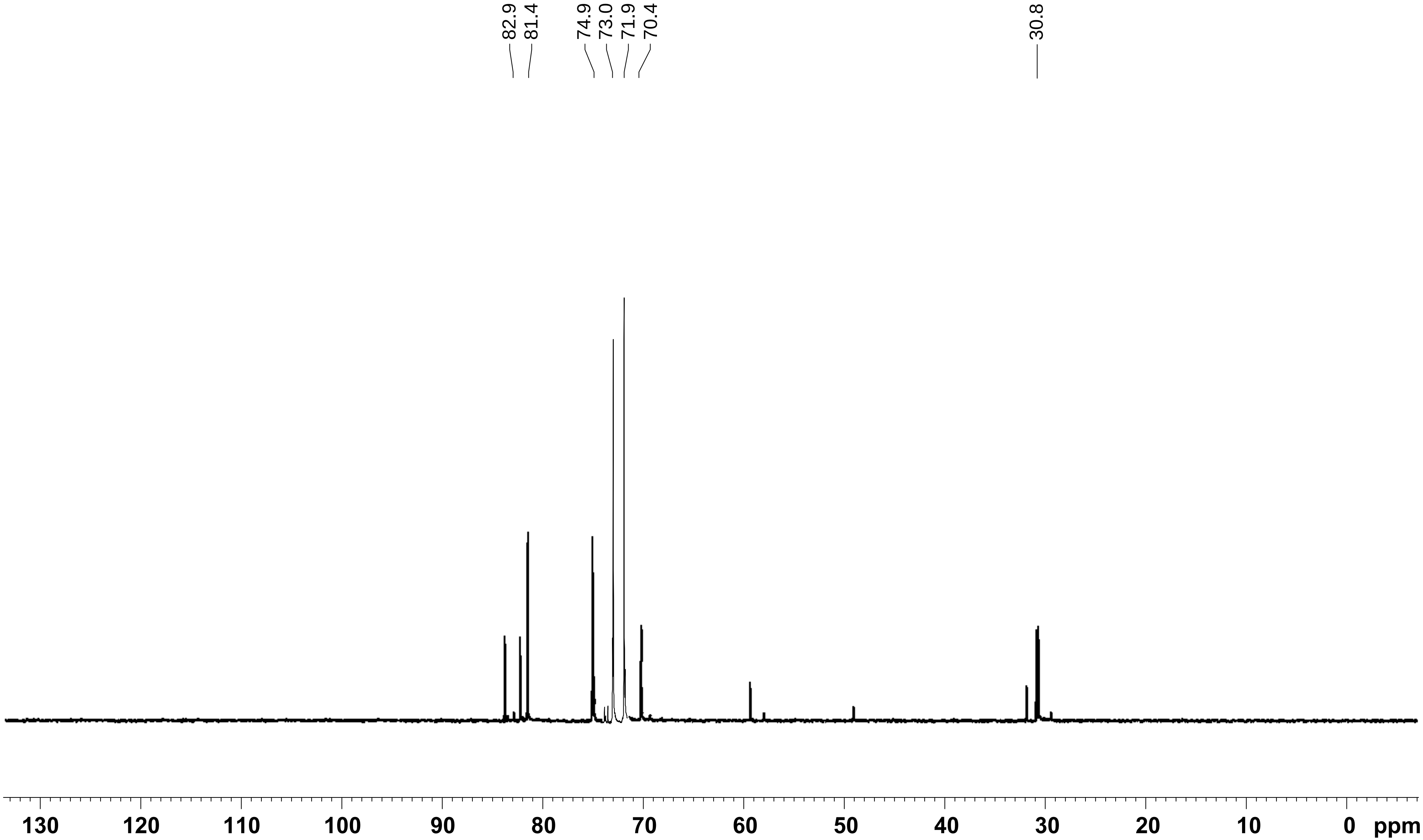

Figure 7: ^{13}C NMR spectrum

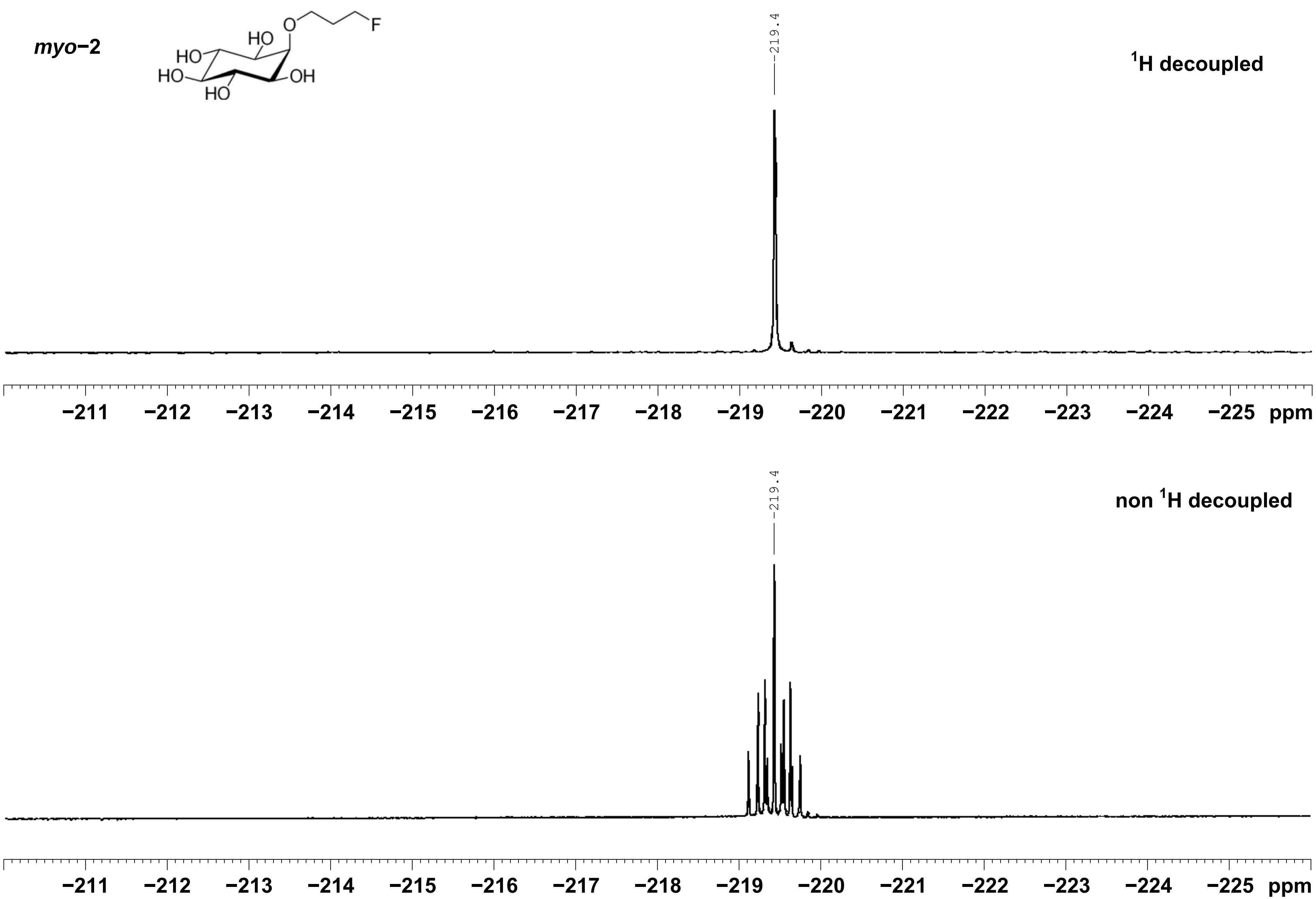

Figure 8: ^{19}F NMR spectrum

myo-**3**

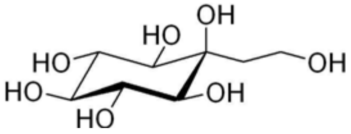

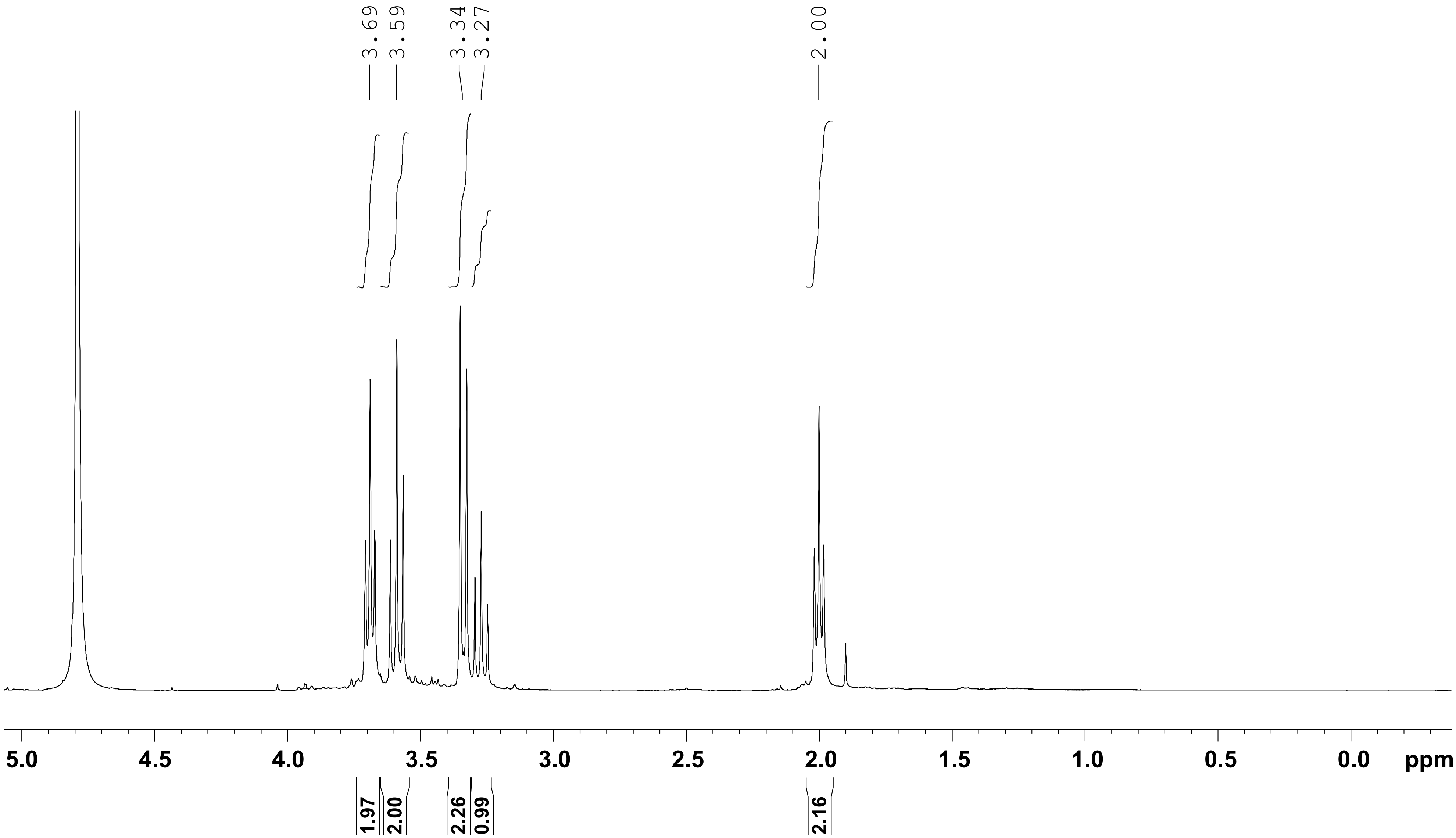

Figure 9: ^{1}H NMR spectrum

***myo*−3**

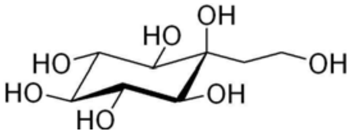

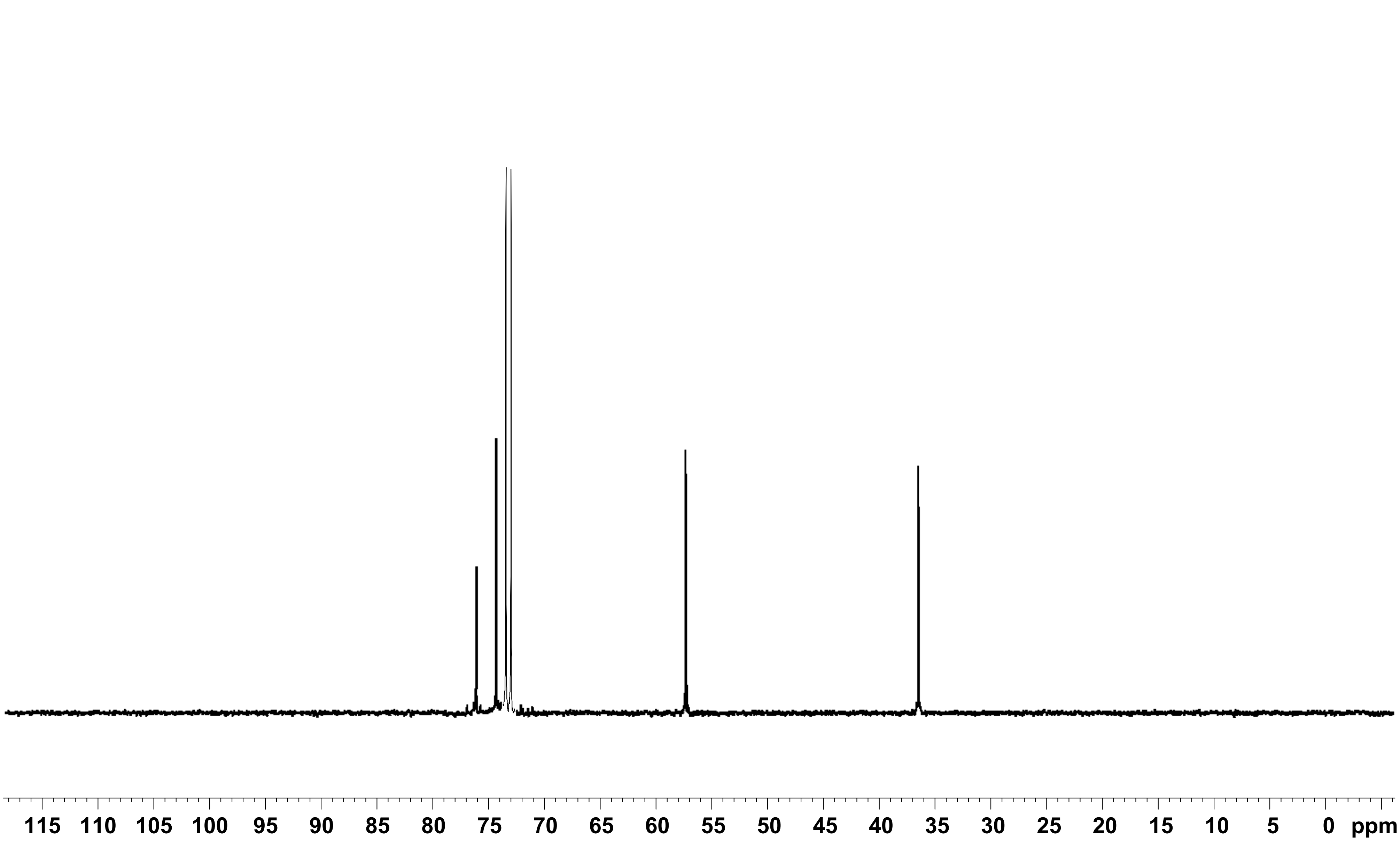

Figure 10: ^{13}C NMR spectrum

scyllo−3

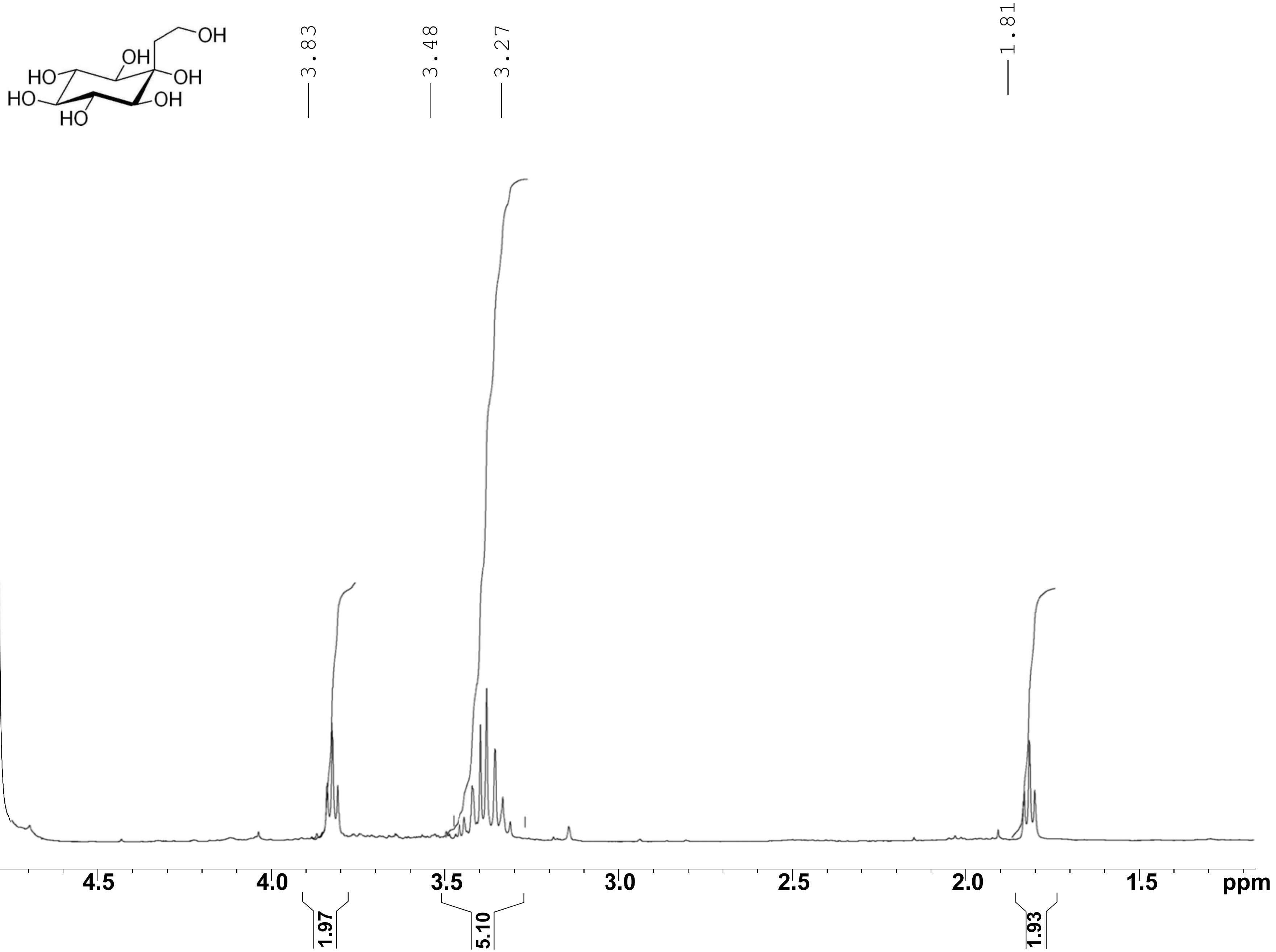

Figure 11: ^{1}H NMR spectrum

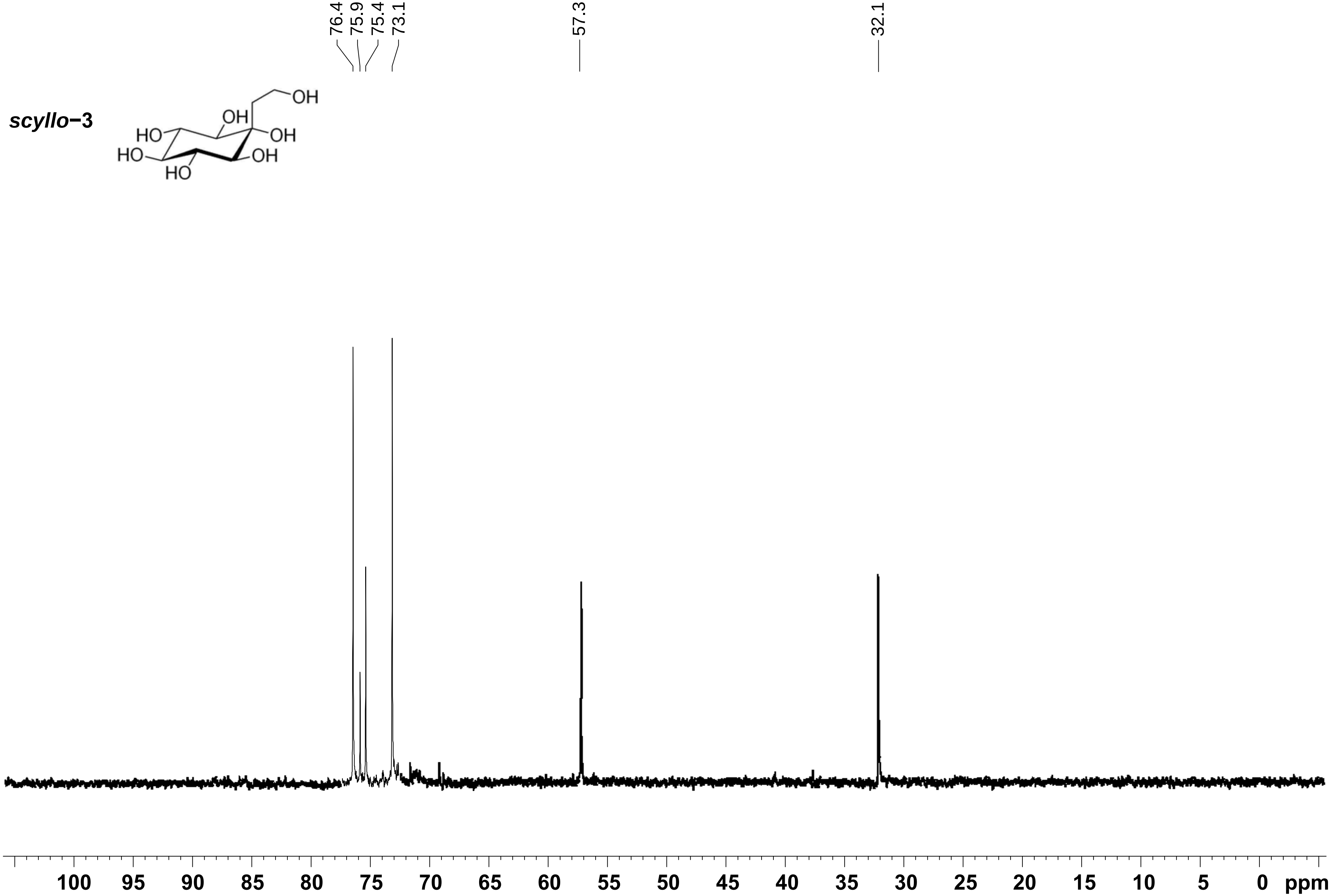

Figure 12: ^{13}C NMR spectrum

myo−**4**

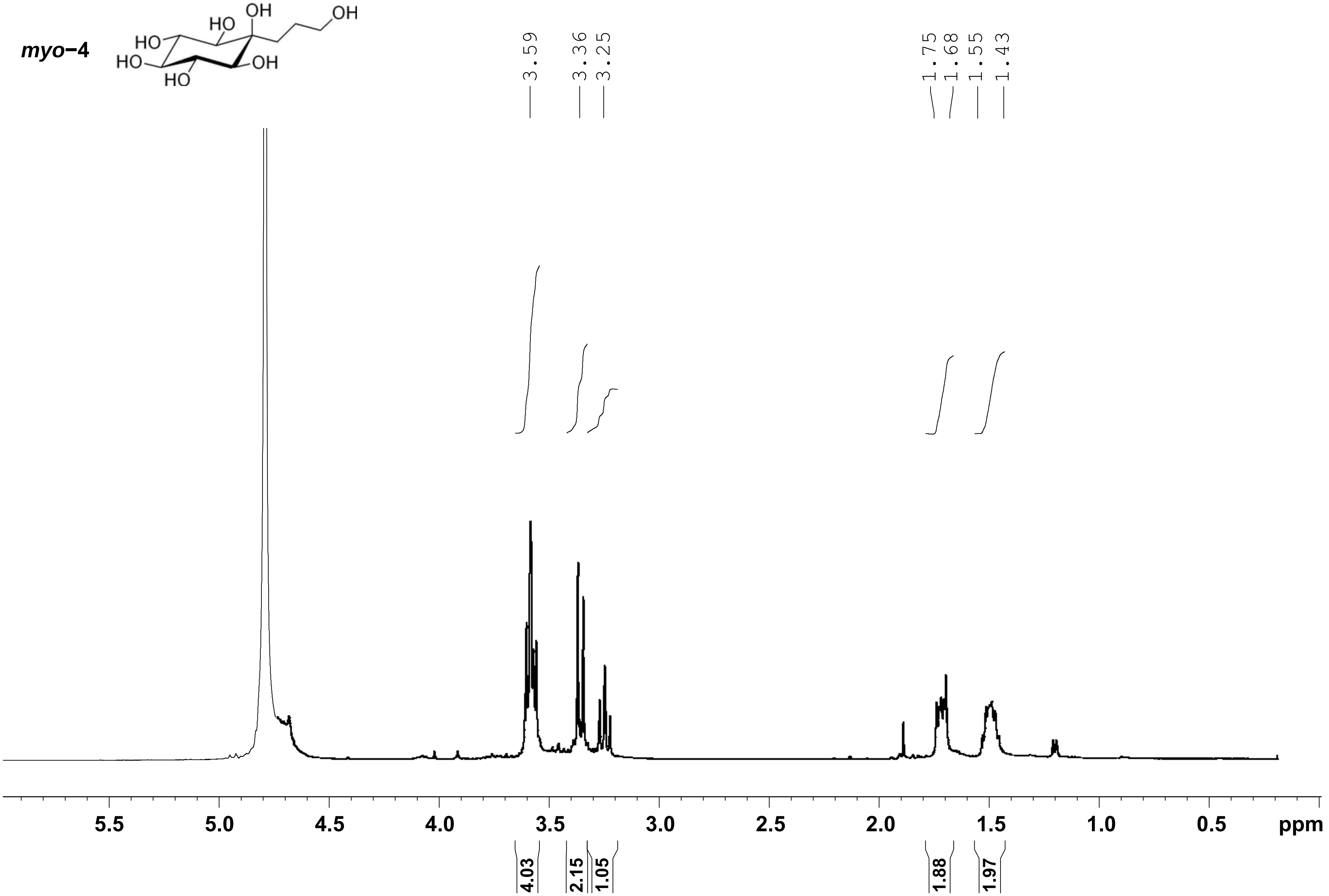

Figure 13: ^{1}H NMR spectrum

***myo*−4**

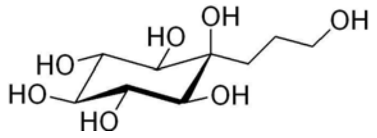

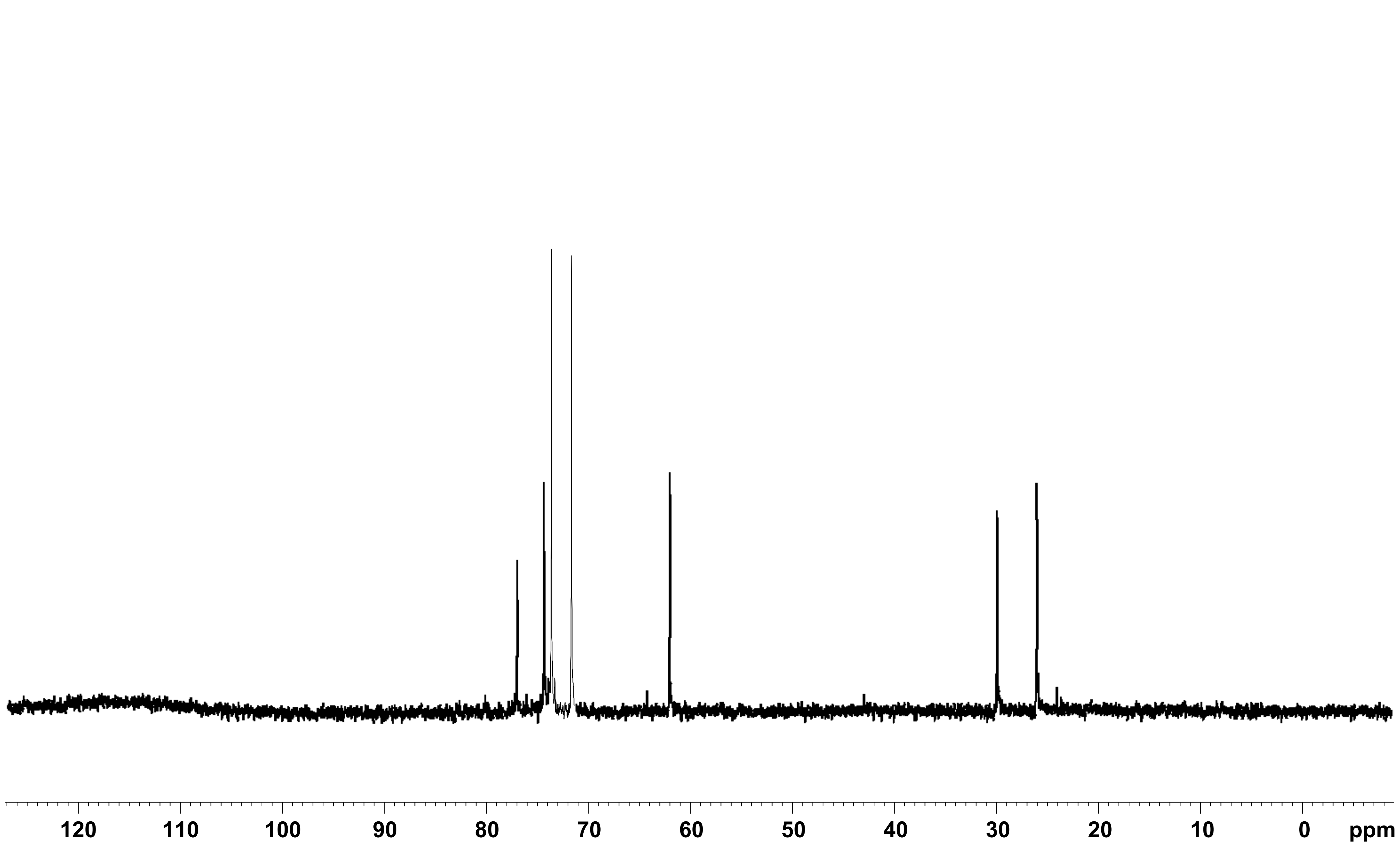

Figure 14: ^{13}C NMR spectrum

***scyllo*-4**

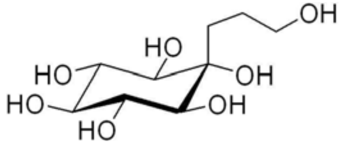

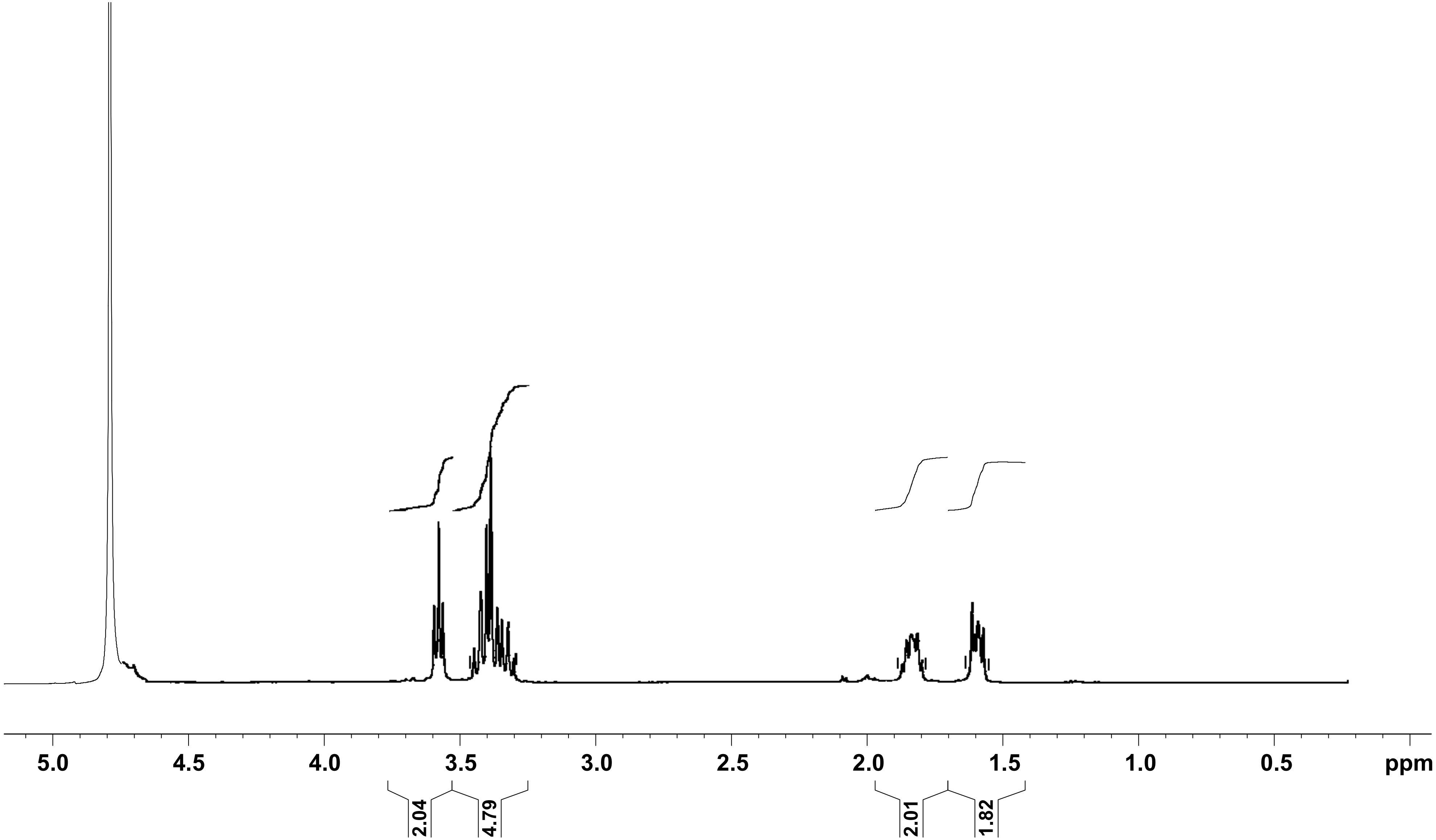

Figure 15: ^{1}H NMR spectrum

***scyllo*−4**

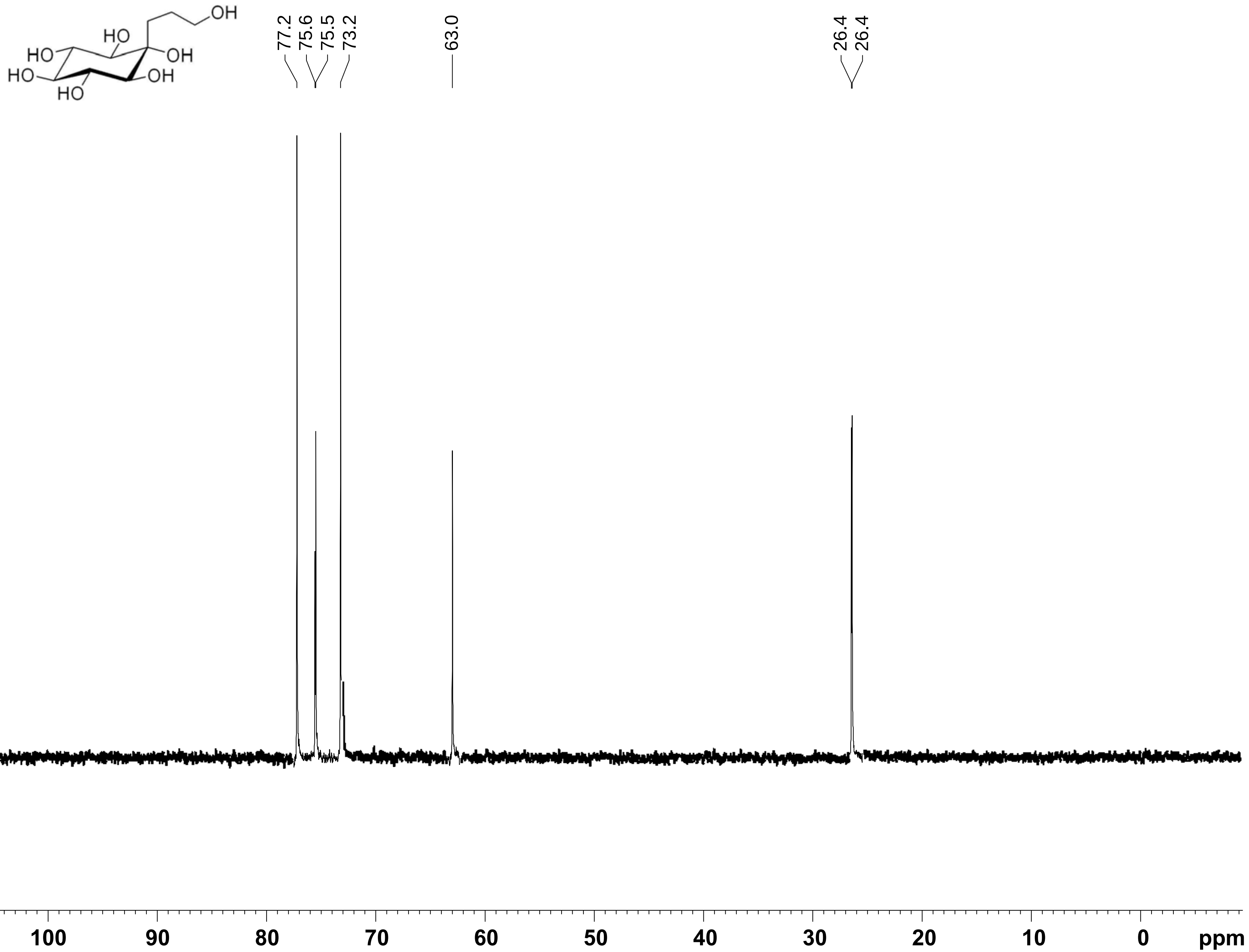

Figure 16: ^{13}C NMR spectrum

***myo*−5**

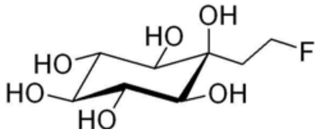

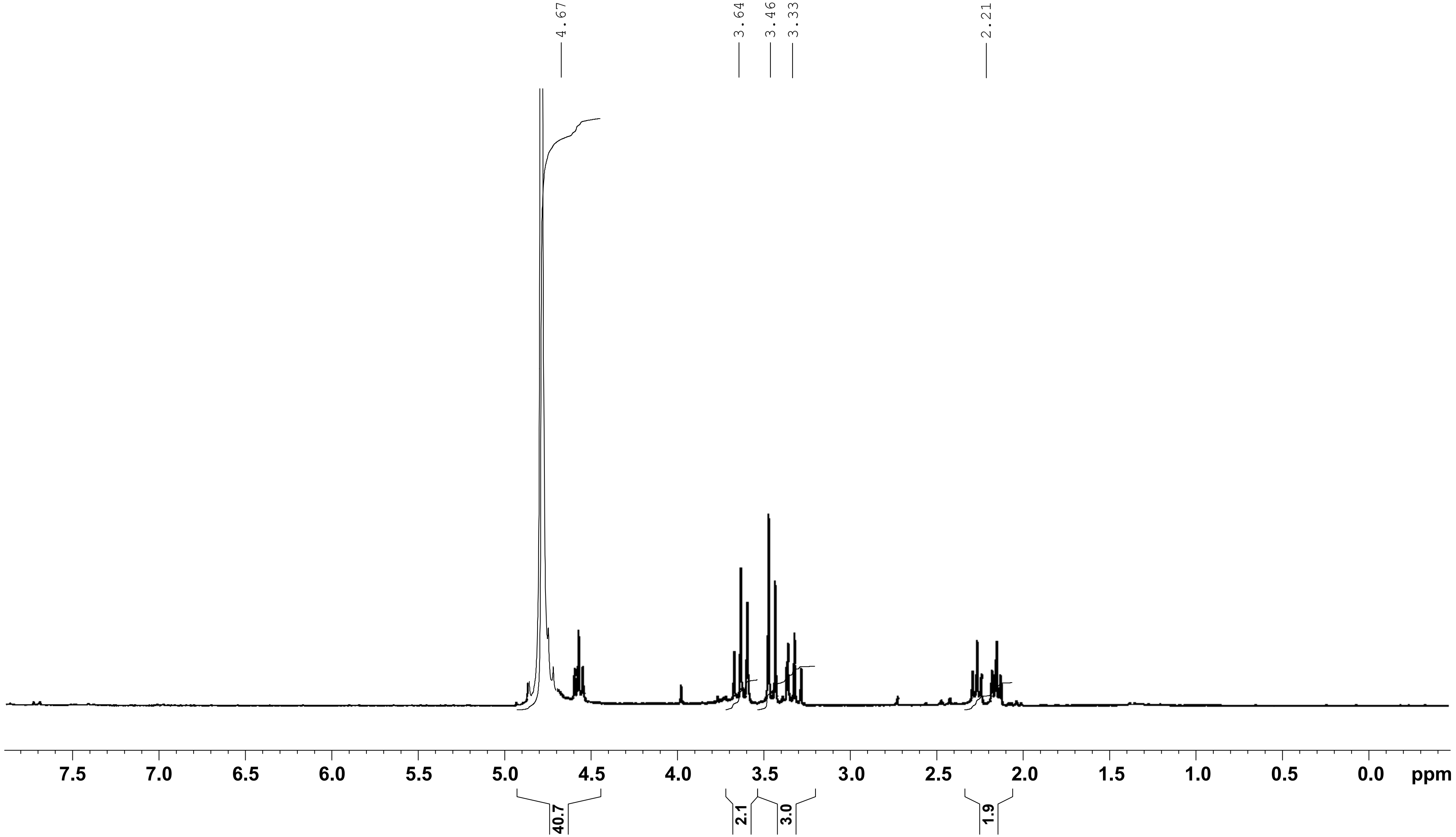

Figure 17: ^{1}H NMR spectrum

***myo*−5**

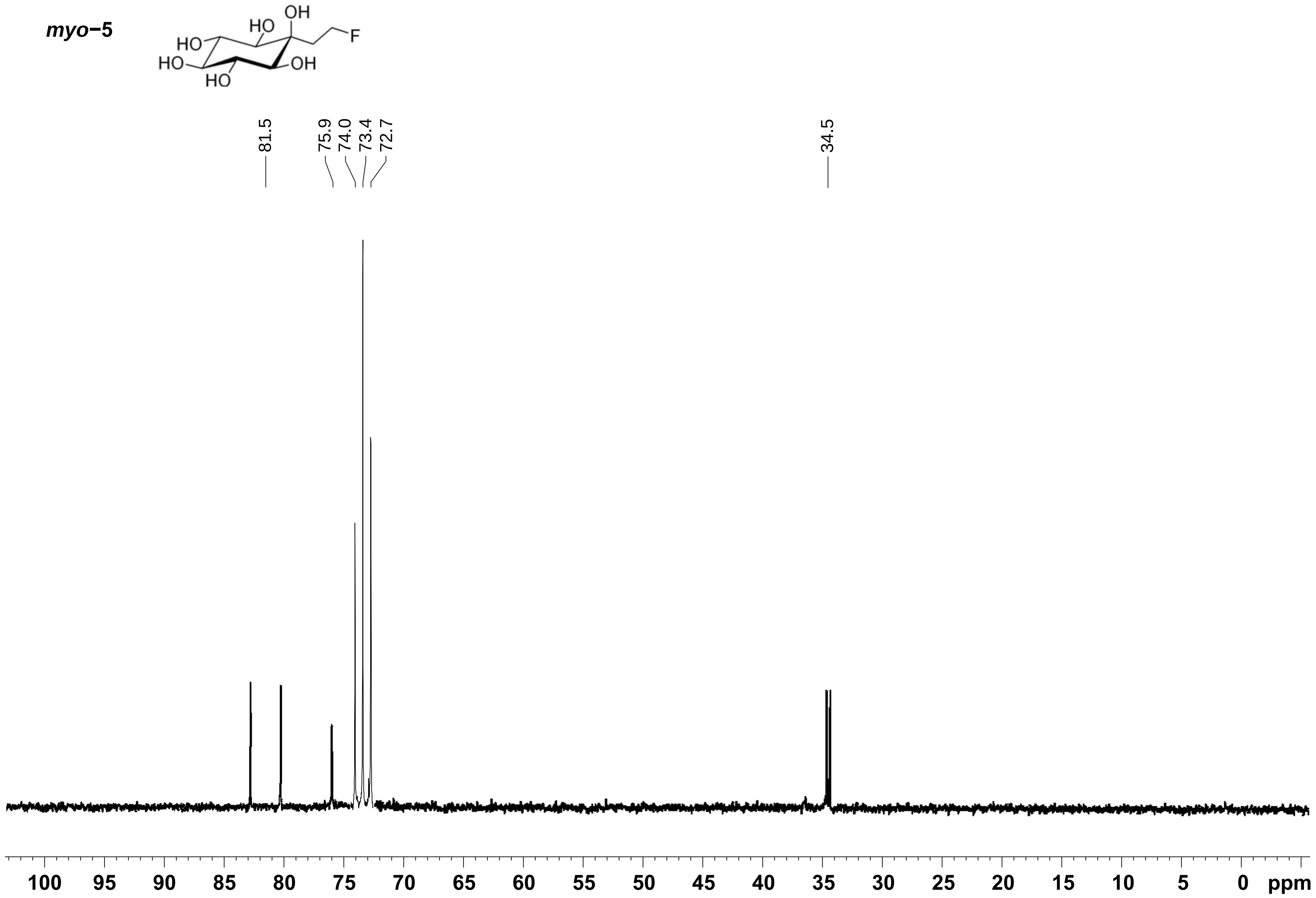

Figure 18: ^{13}C NMR spectrum

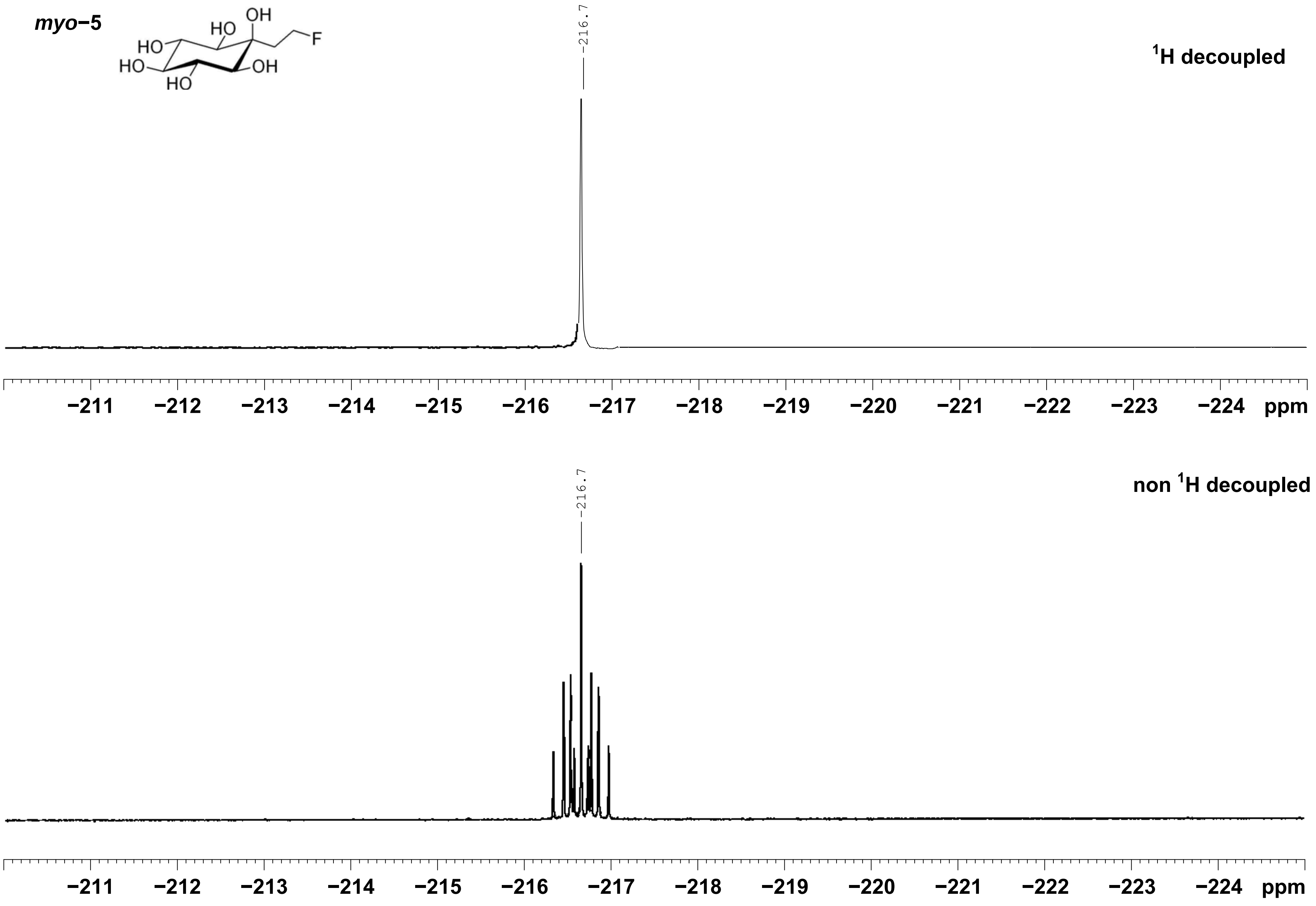

Figure 19: ^{19}F NMR spectrum

***myo*−6**

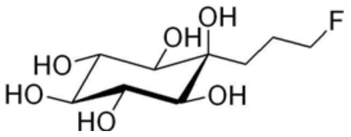

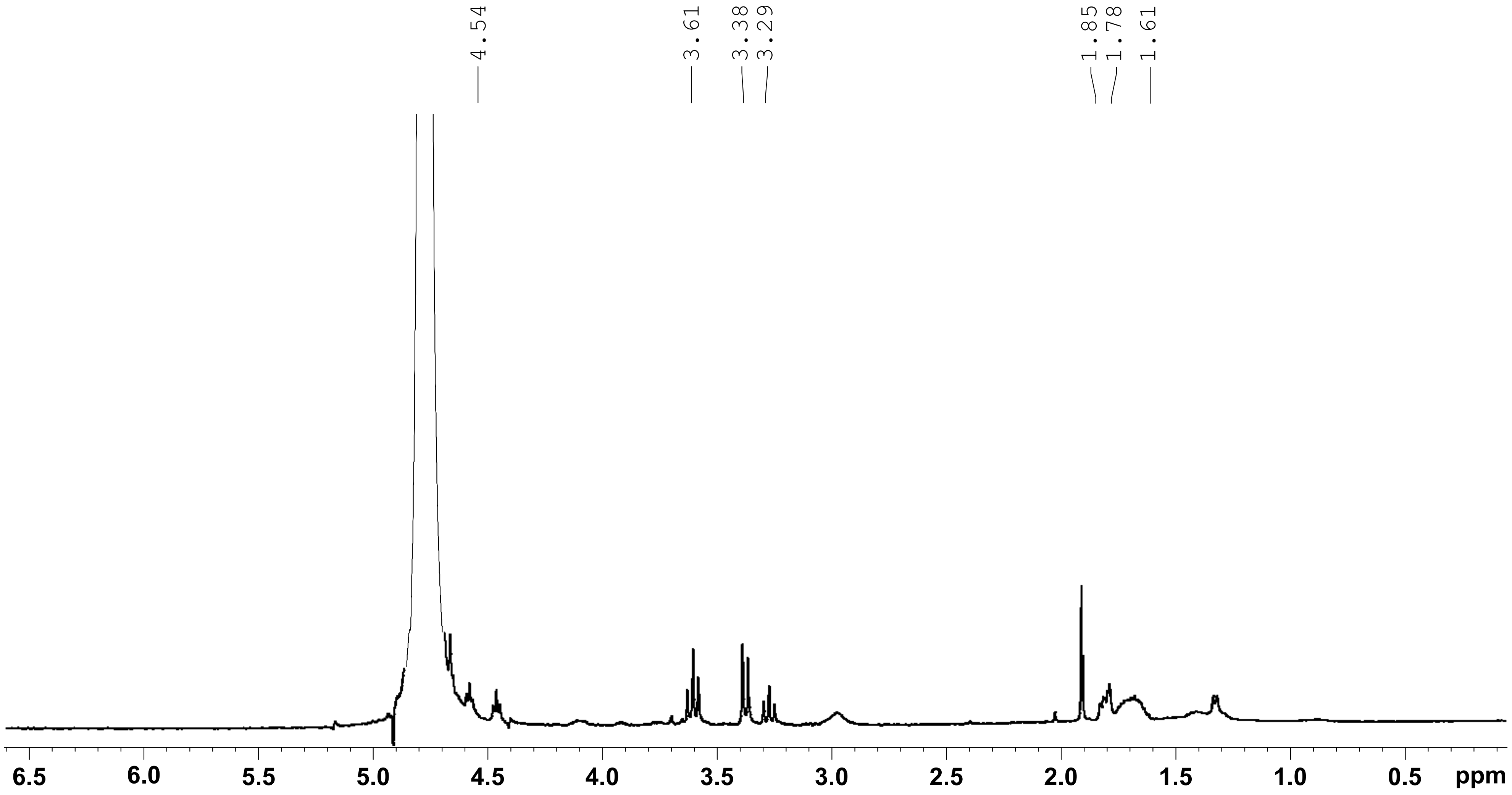

Figure 20: ^{1}H NMR spectrum

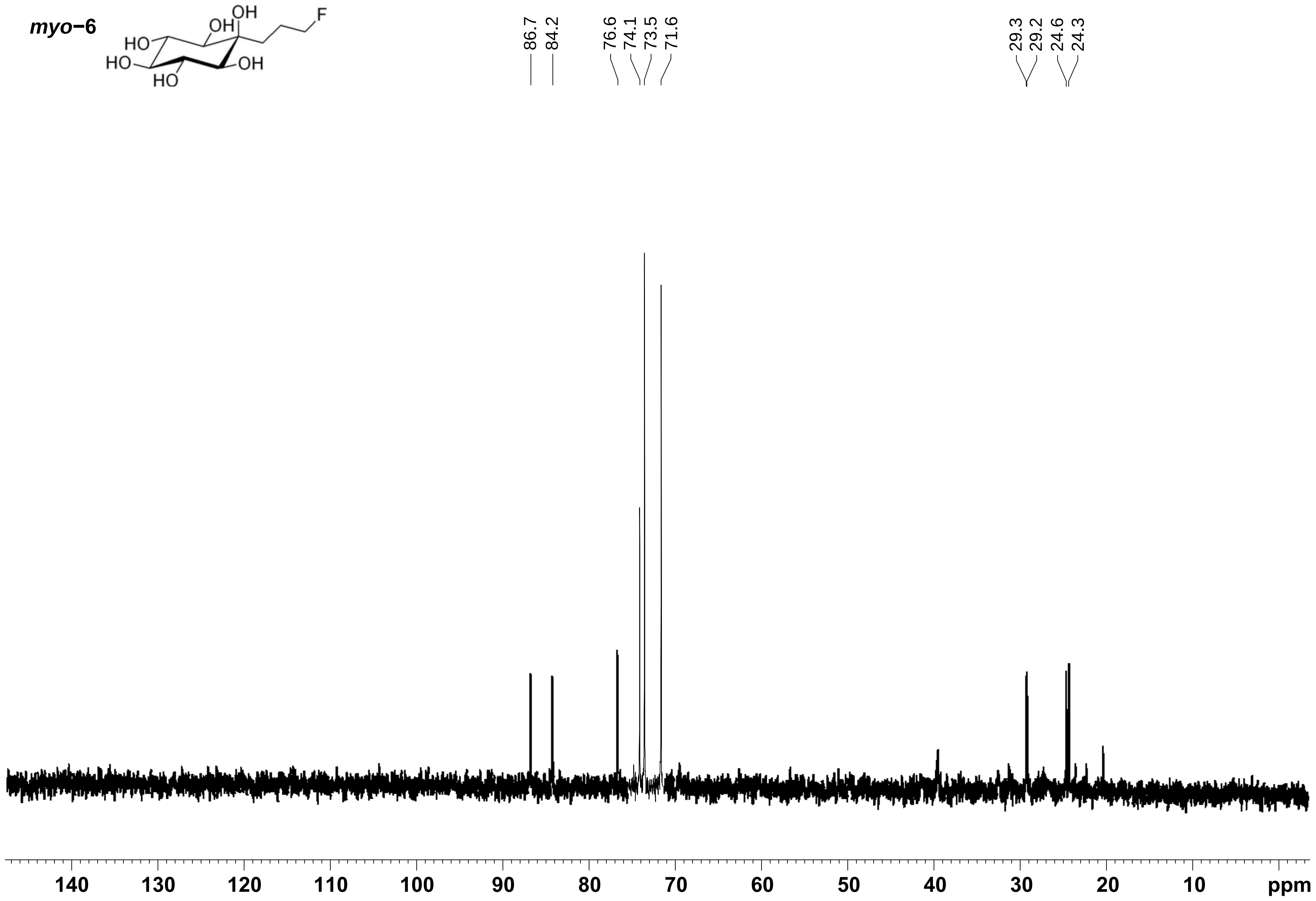

Figure 21: ^{13}C NMR spectrum

***myo*-6**

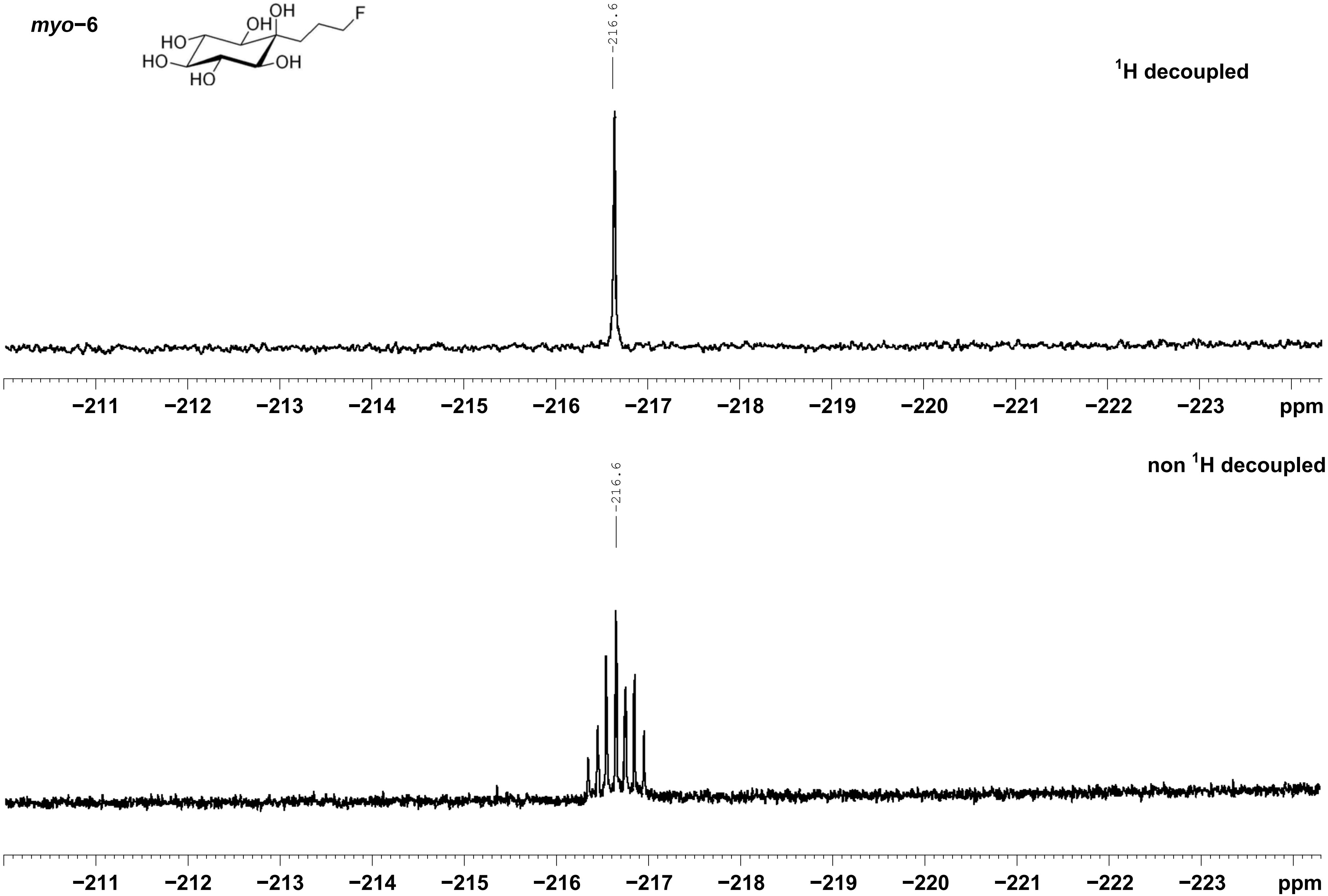

Figure 22: ^{19}F NMR spectrum

***scyllo*−6**

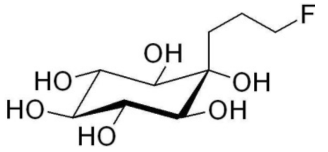

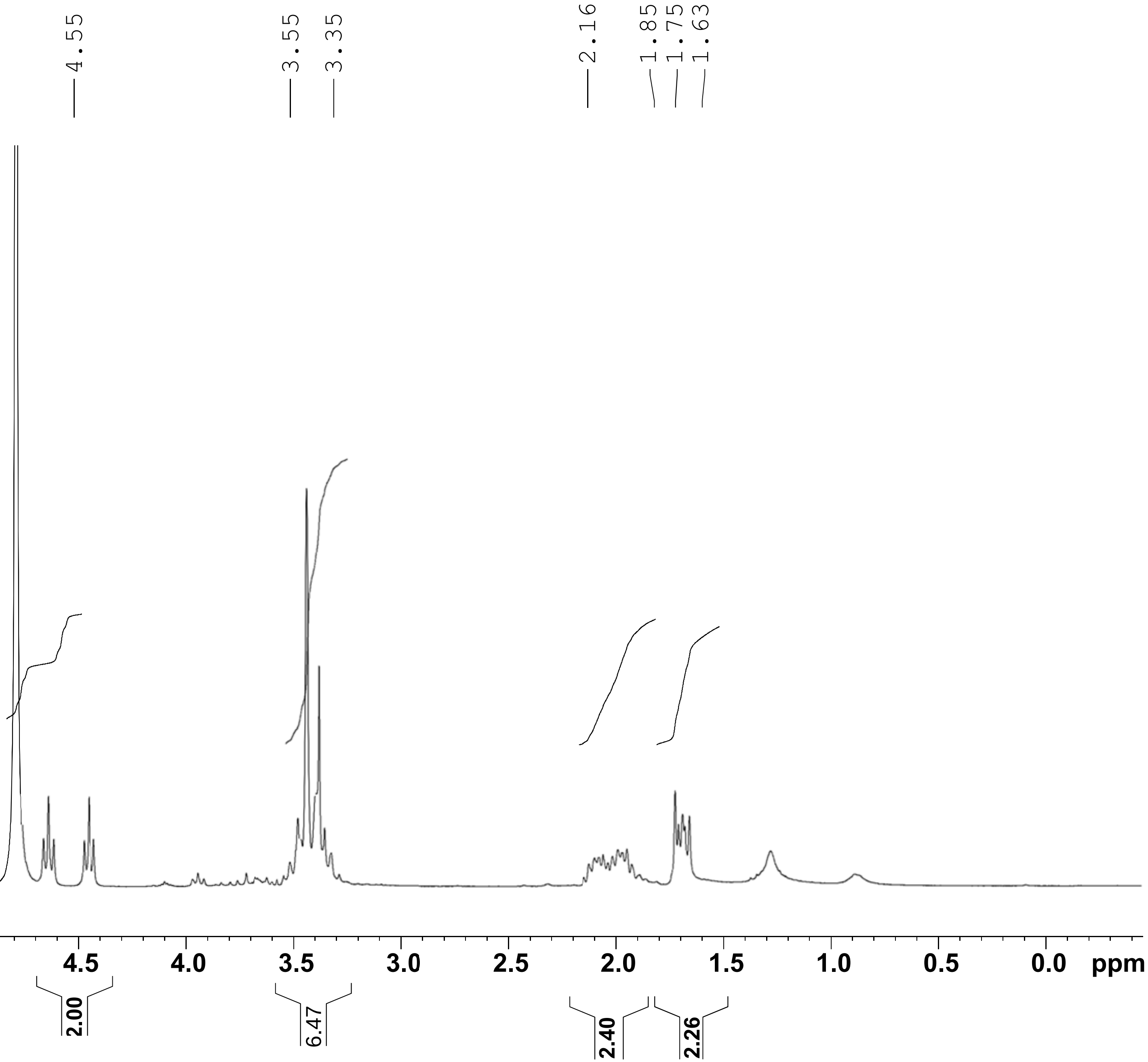

Figure 23: ^{1}H NMR spectrum

***scyllo*−6**

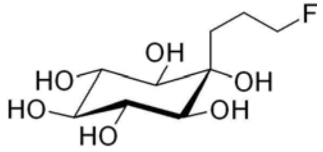

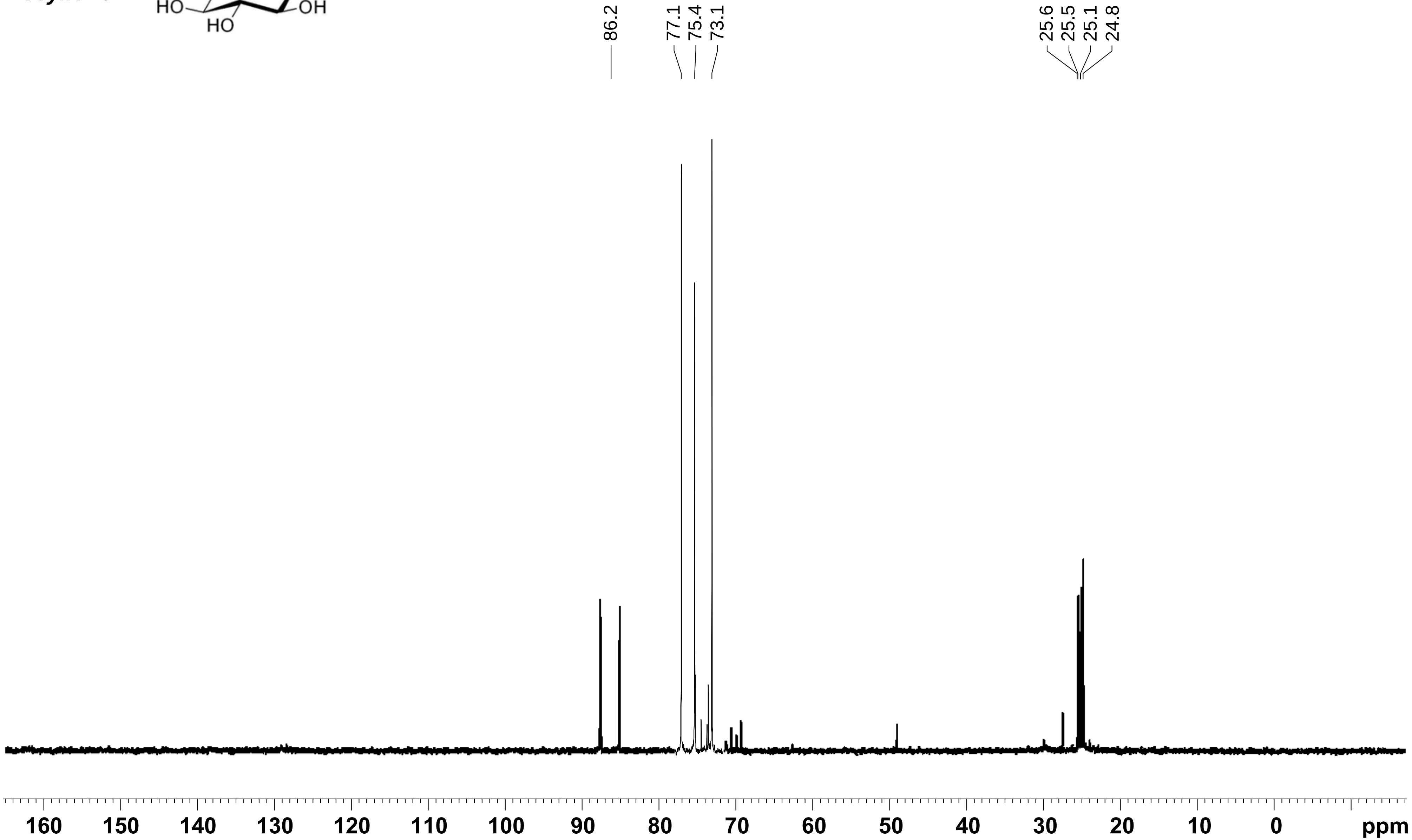

Figure 24: ^{13}C NMR spectrum

scyllo−**6**

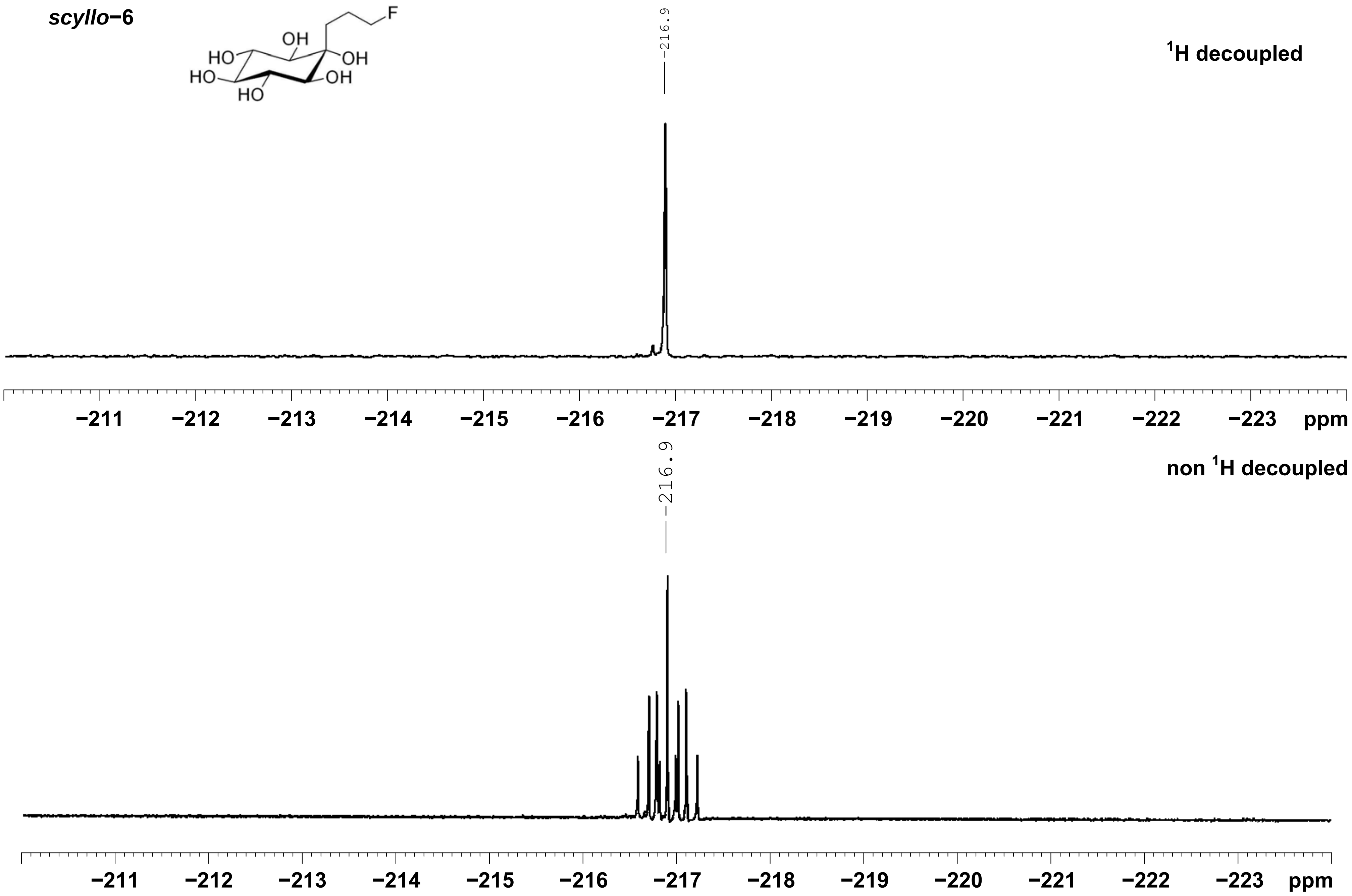

Figure 25: ^{19}F NMR spectrum

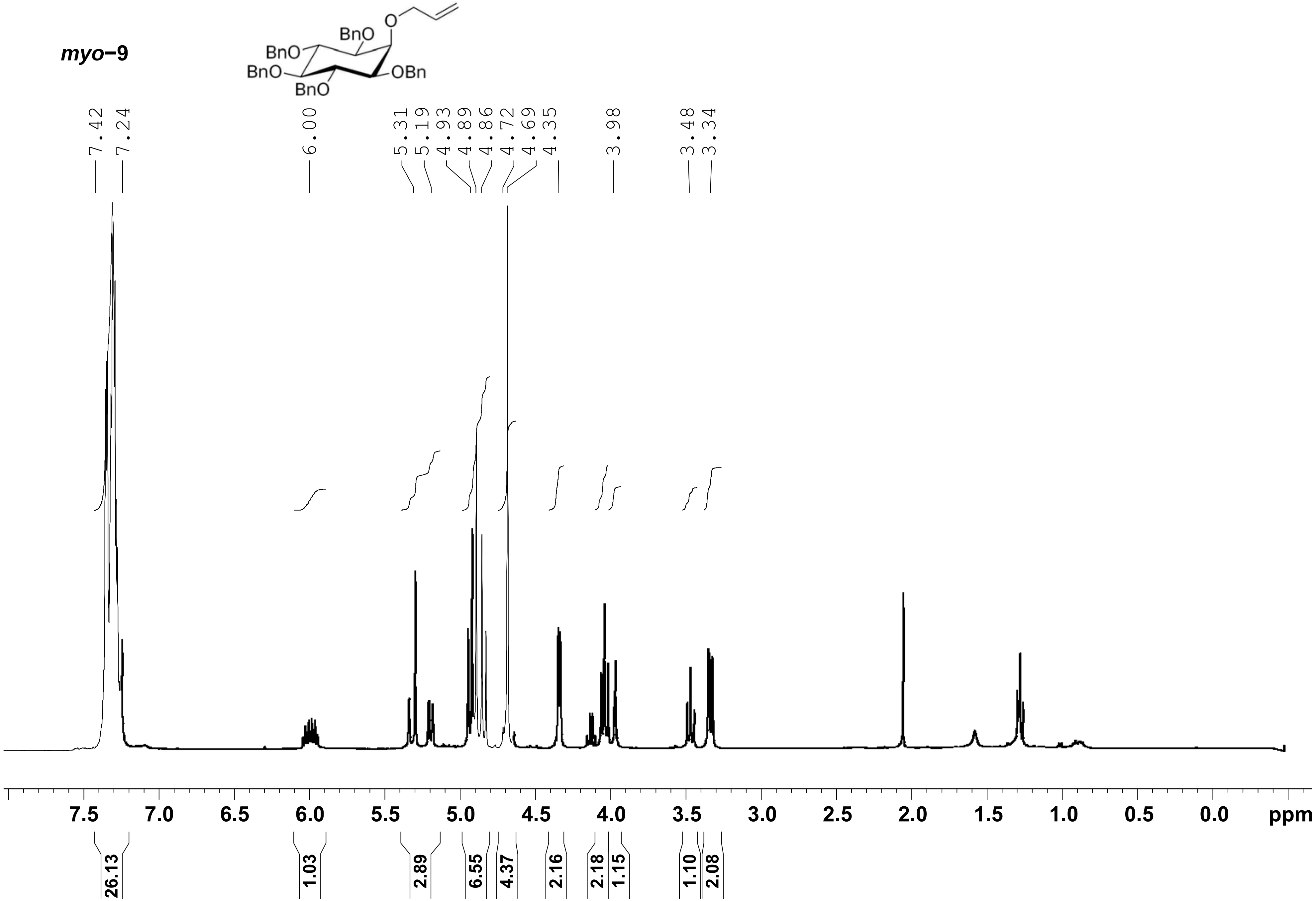

Figure 26: ^{1}H NMR spectrum

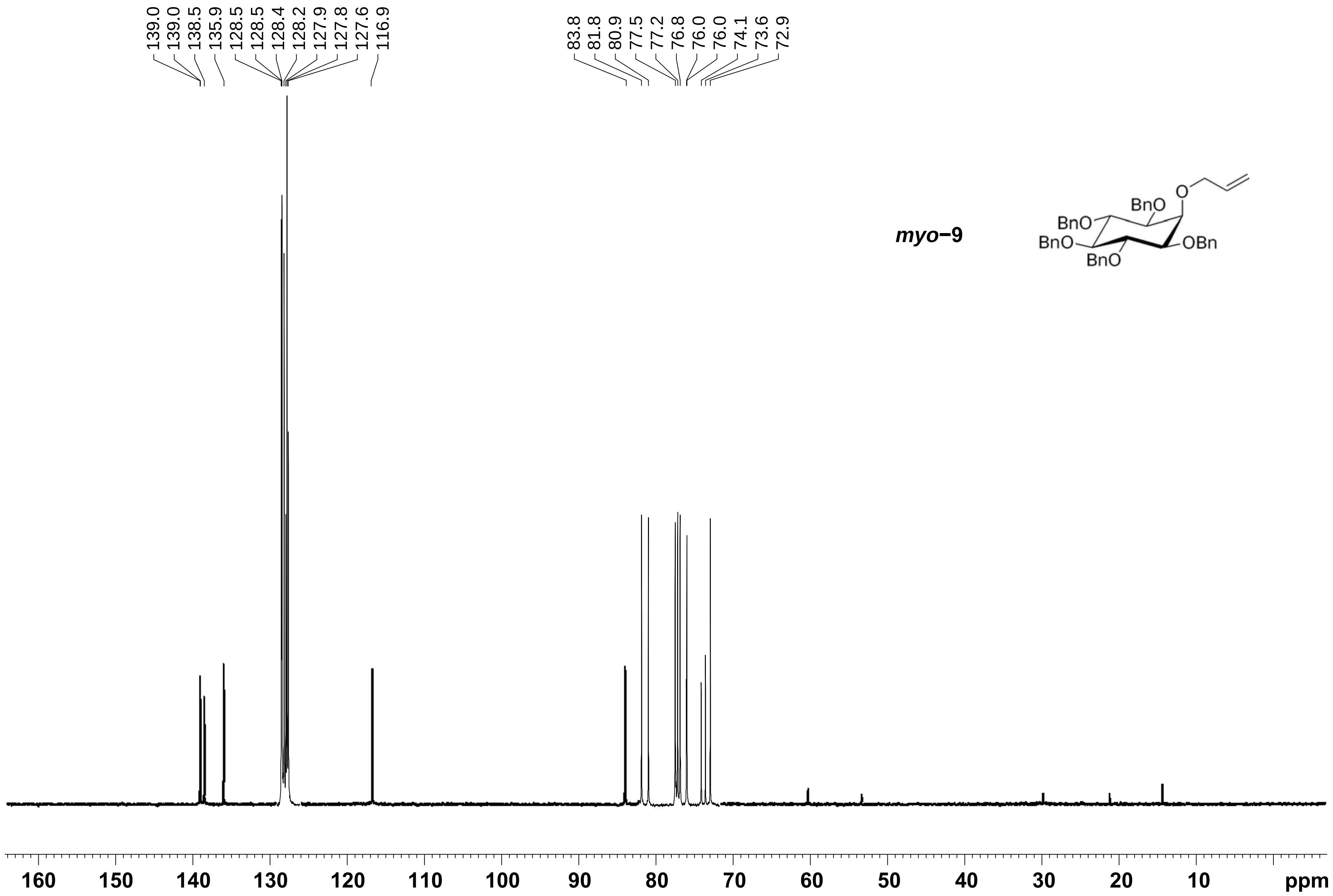

Figure 27: ^{13}C NMR spectrum

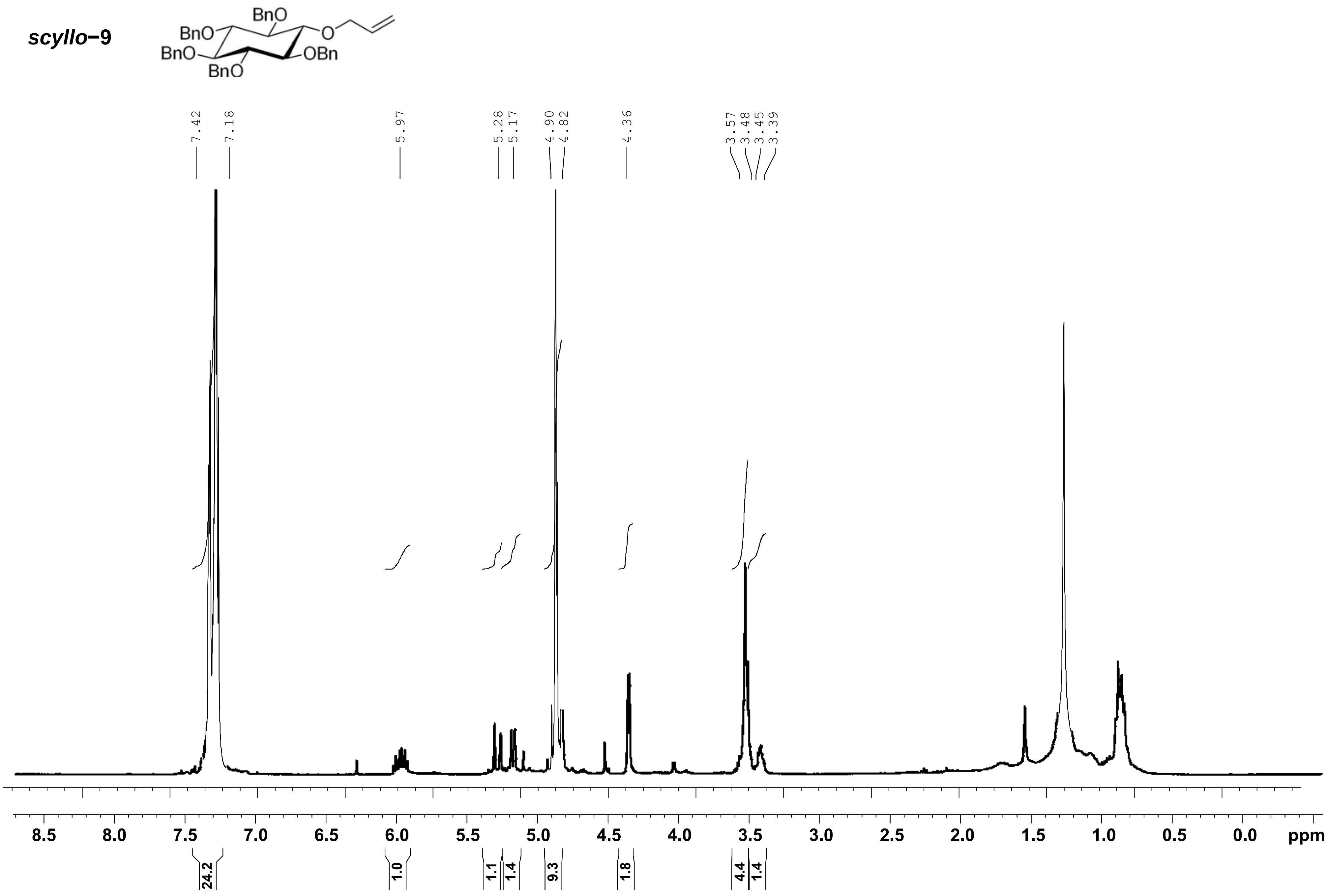

Figure 28: ¹H NMR spectrum

scyllo−**9**

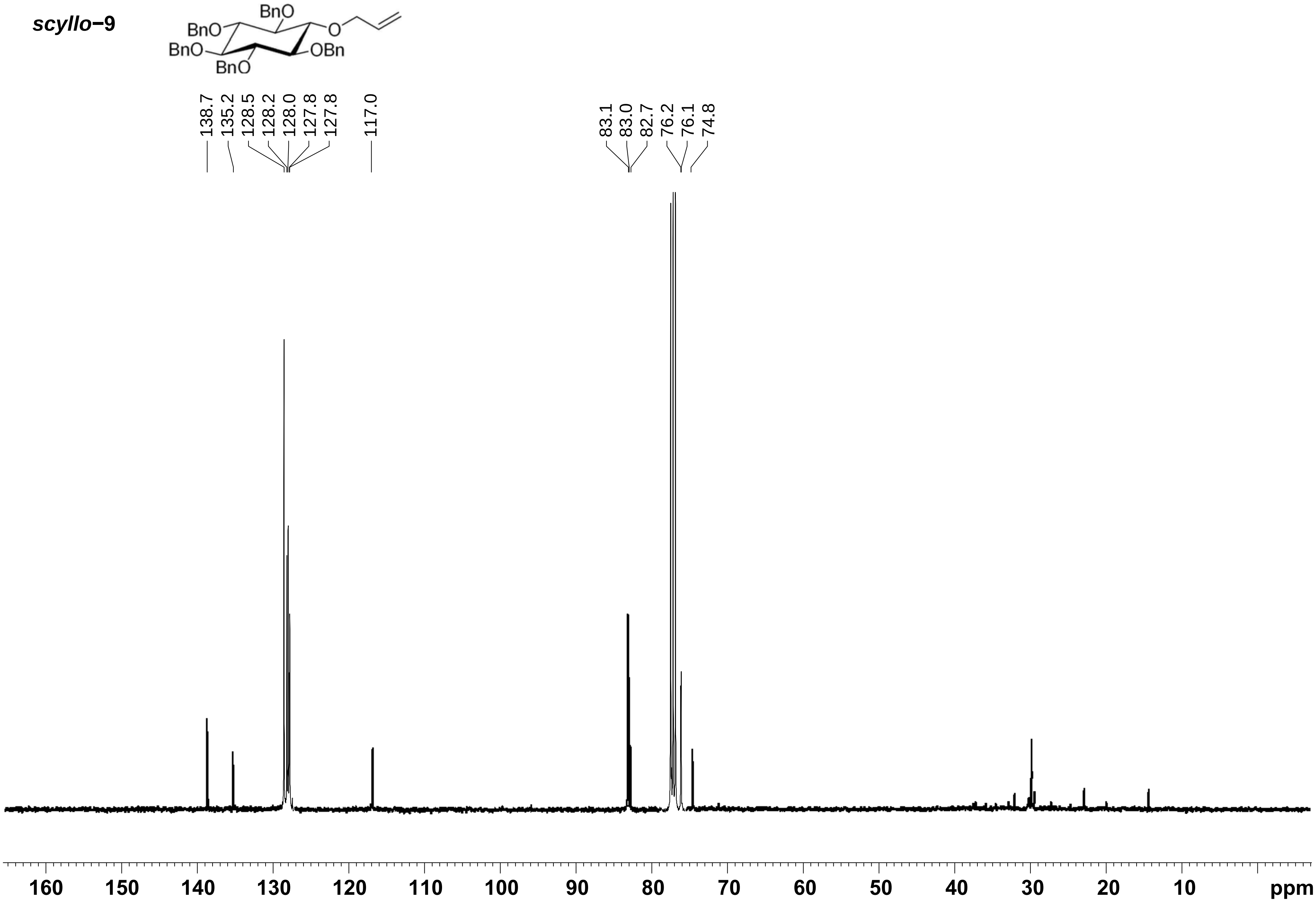

Figure 29: ^{13}C NMR spectrum

***myo*-10**

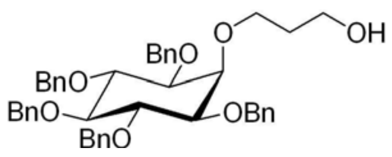

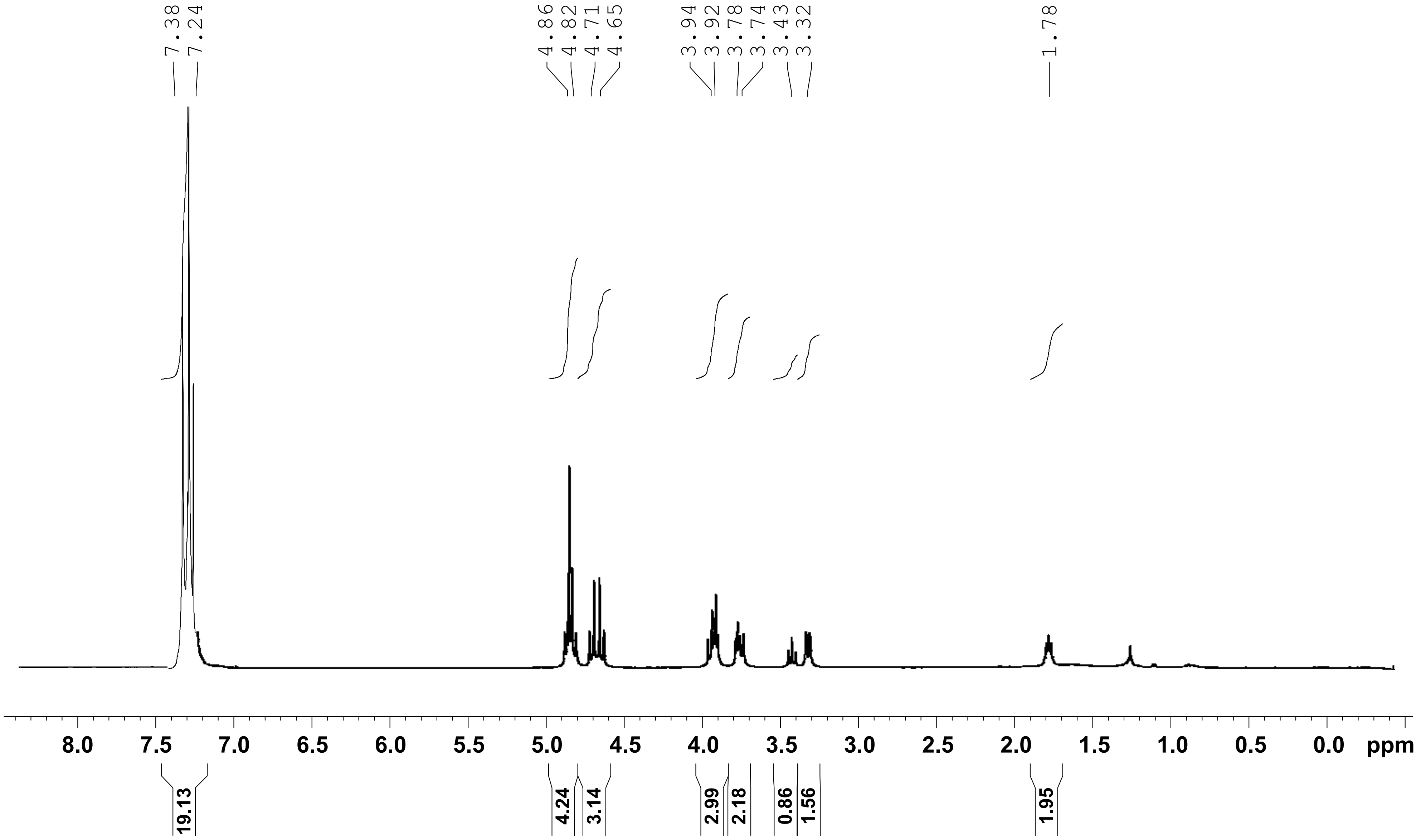

Figure 30: ^{1}H NMR spectrum

***myo*-10**

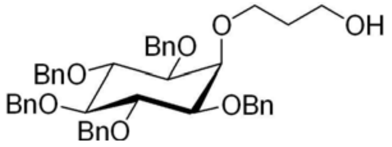

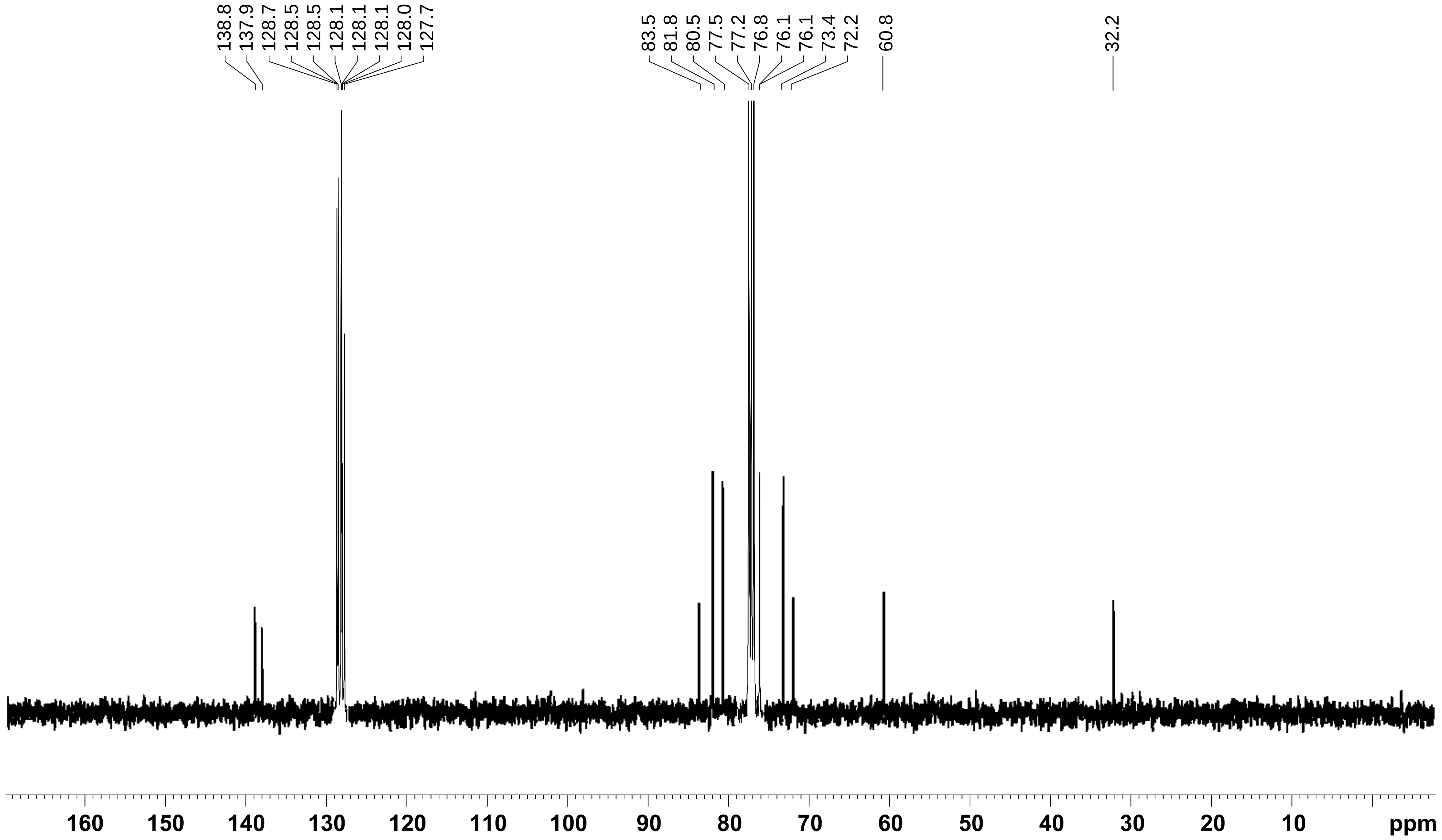

Figure 31: ^{13}C NMR spectrum

scyllo−**10**

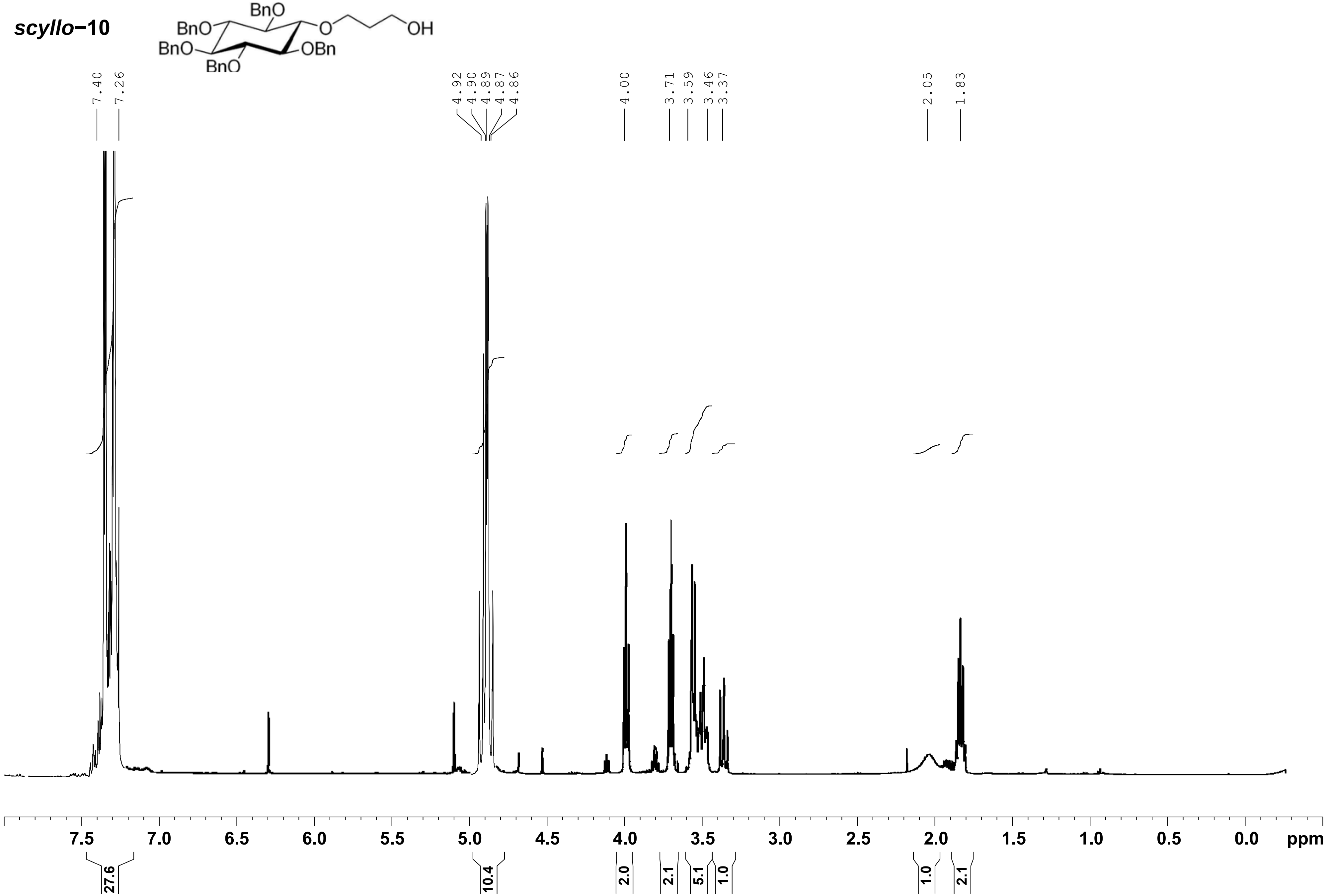

Figure 32: ^{1}H NMR spectrum

***scyllo*−10**

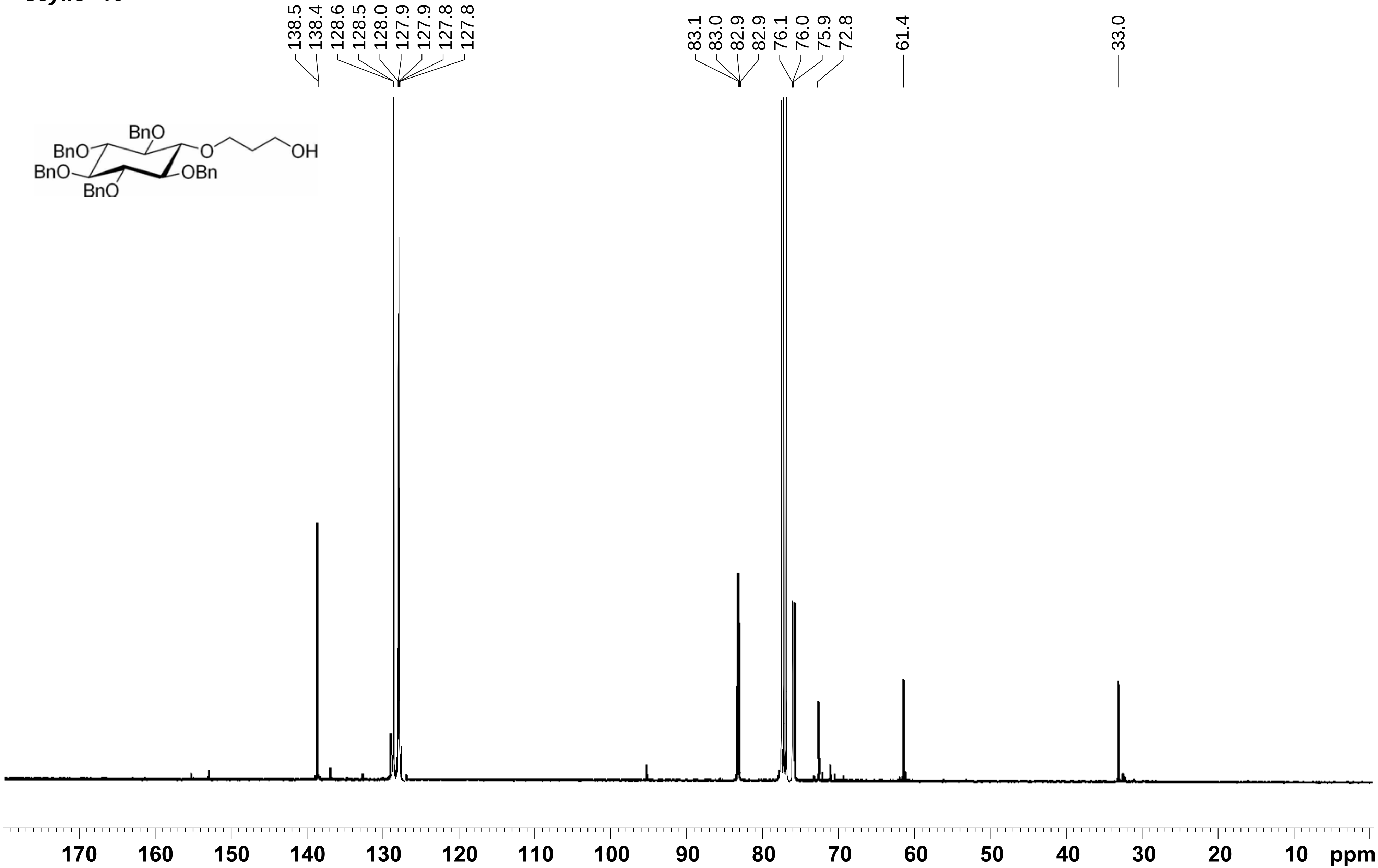

Figure 33: ^{13}C NMR spectrum

scyllo-**11**

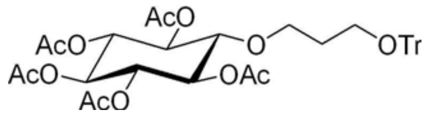

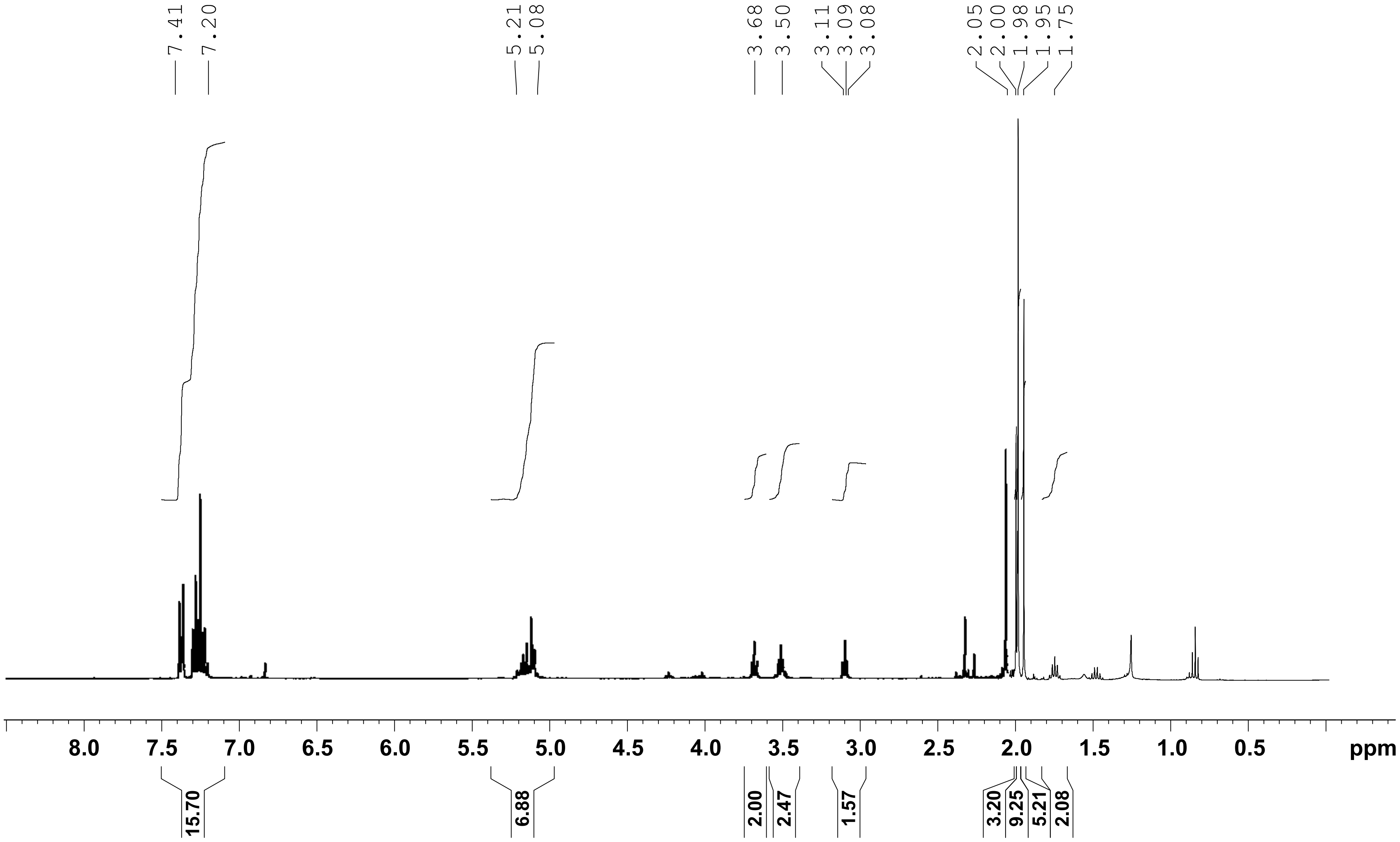

Figure 34: ^{1}H NMR spectrum

***scyllo*-11**

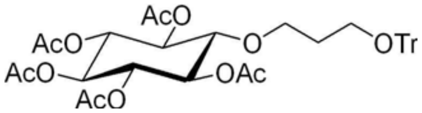

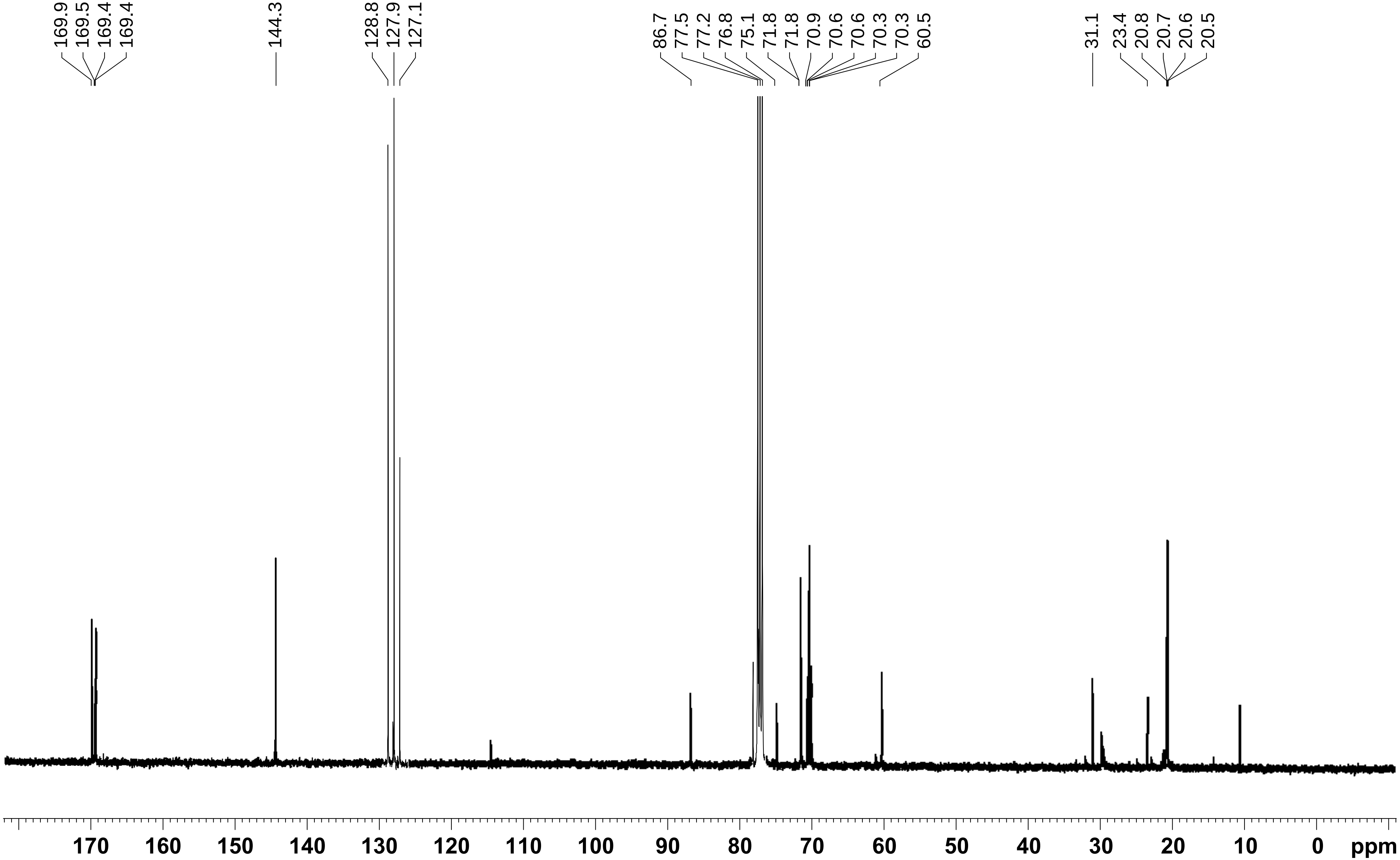

Figure 35: ^{13}C NMR spectrum

myo−12

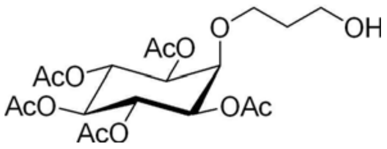

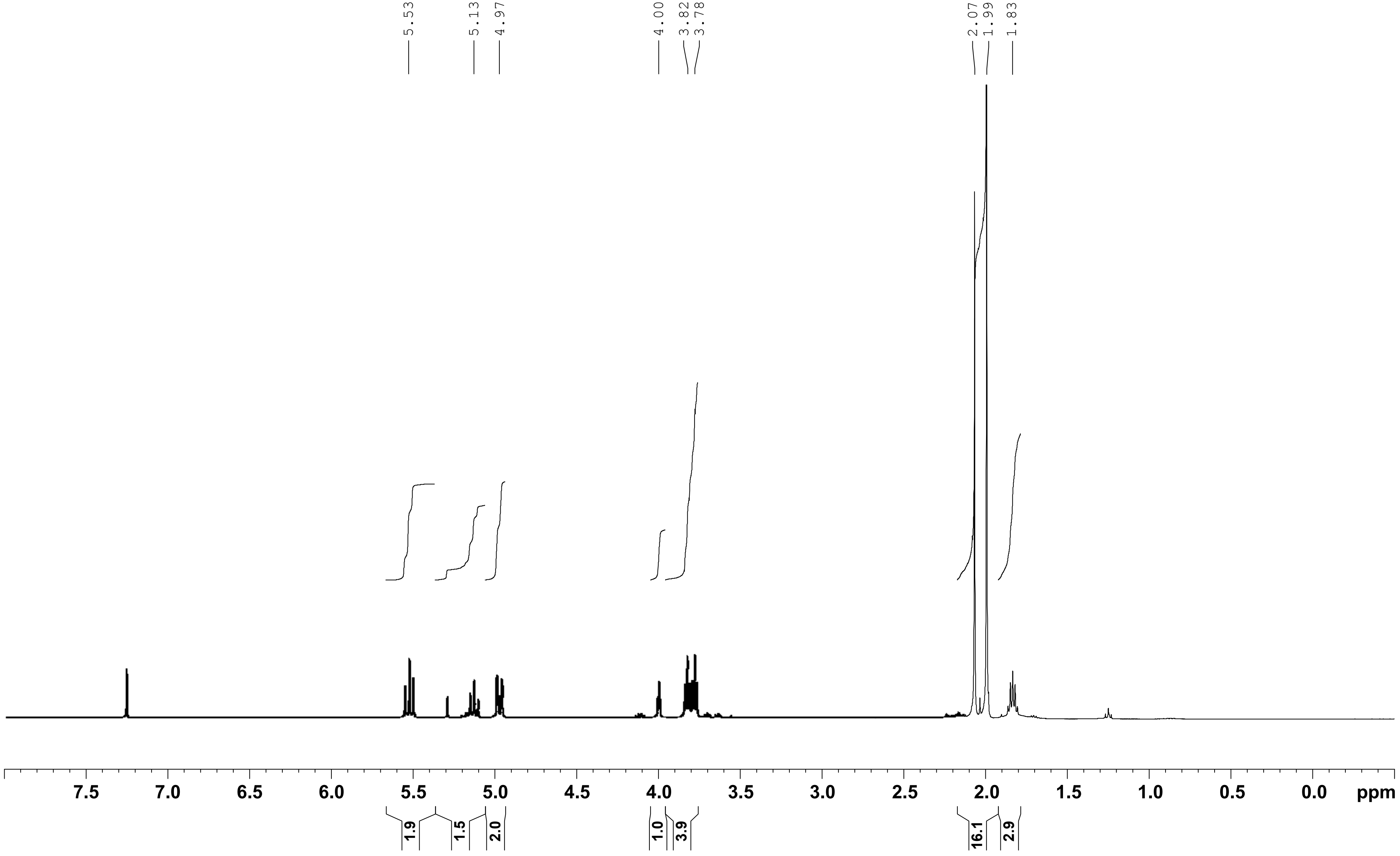

Figure 36: ^{1}H NMR spectrum

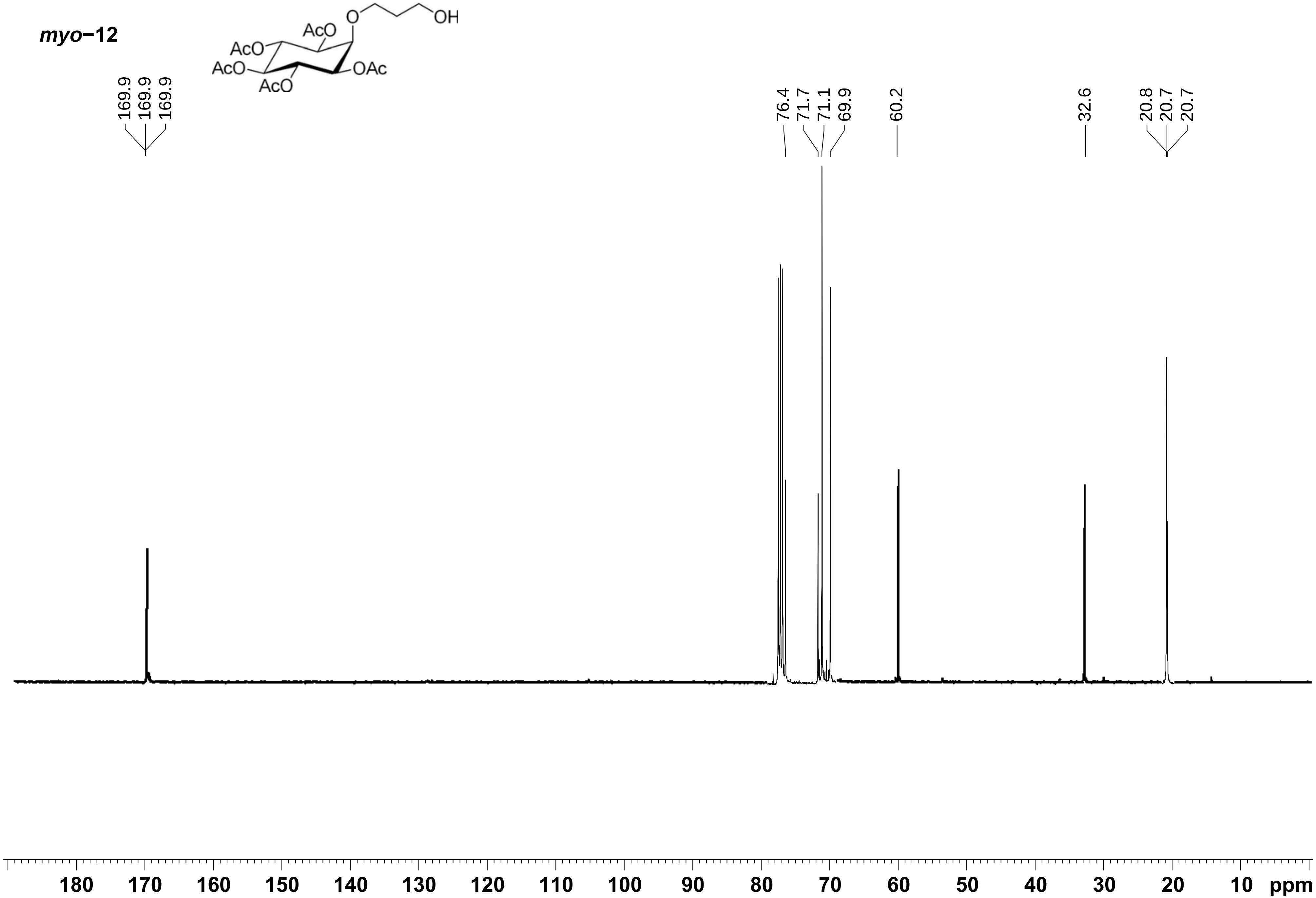

Figure 37: ^{13}C NMR spectrum

***scyllo*−12**

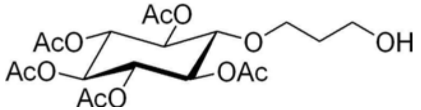

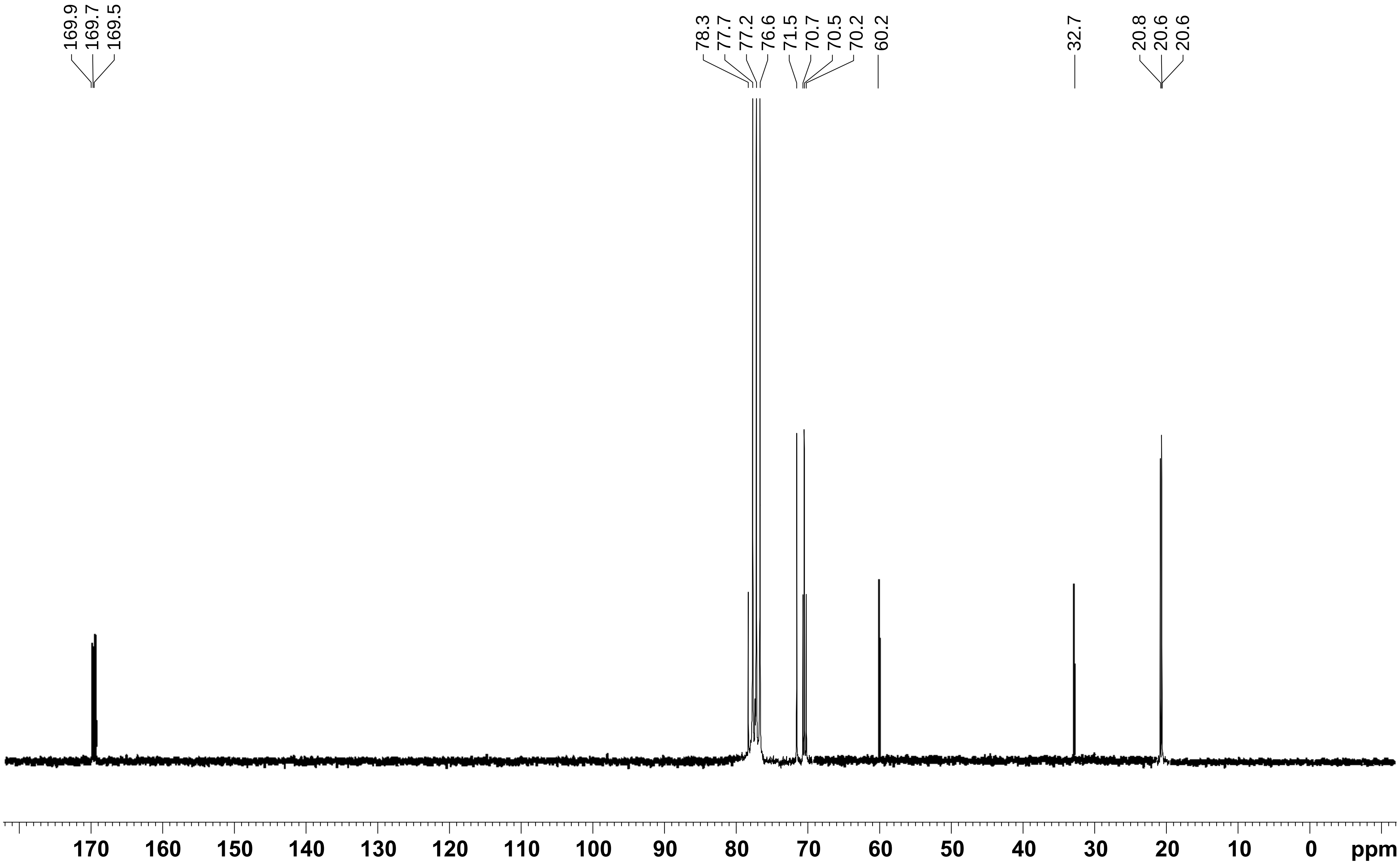

Figure 38: ^{13}C NMR spectrum

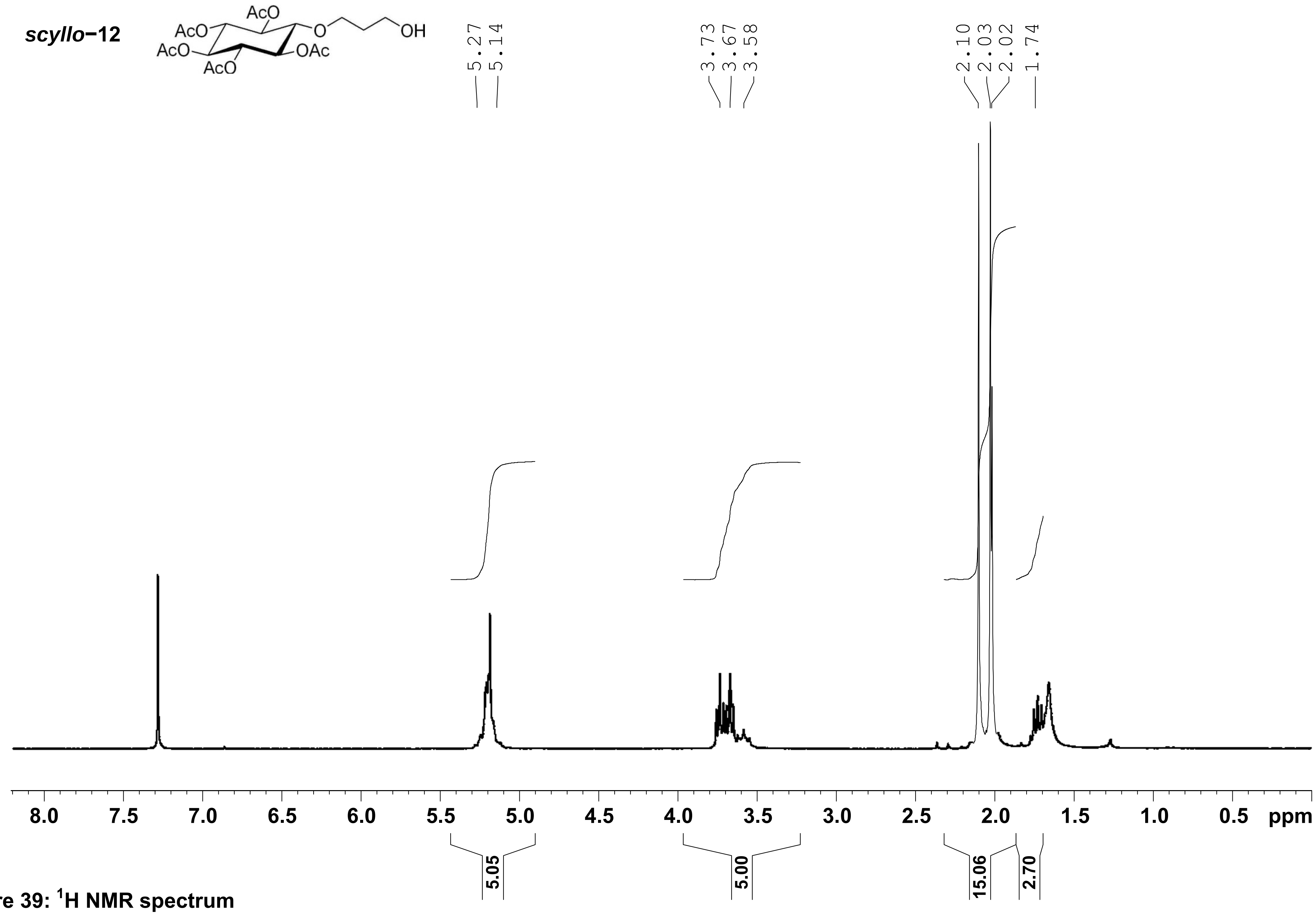

Figure 39: ^{1}H NMR spectrum

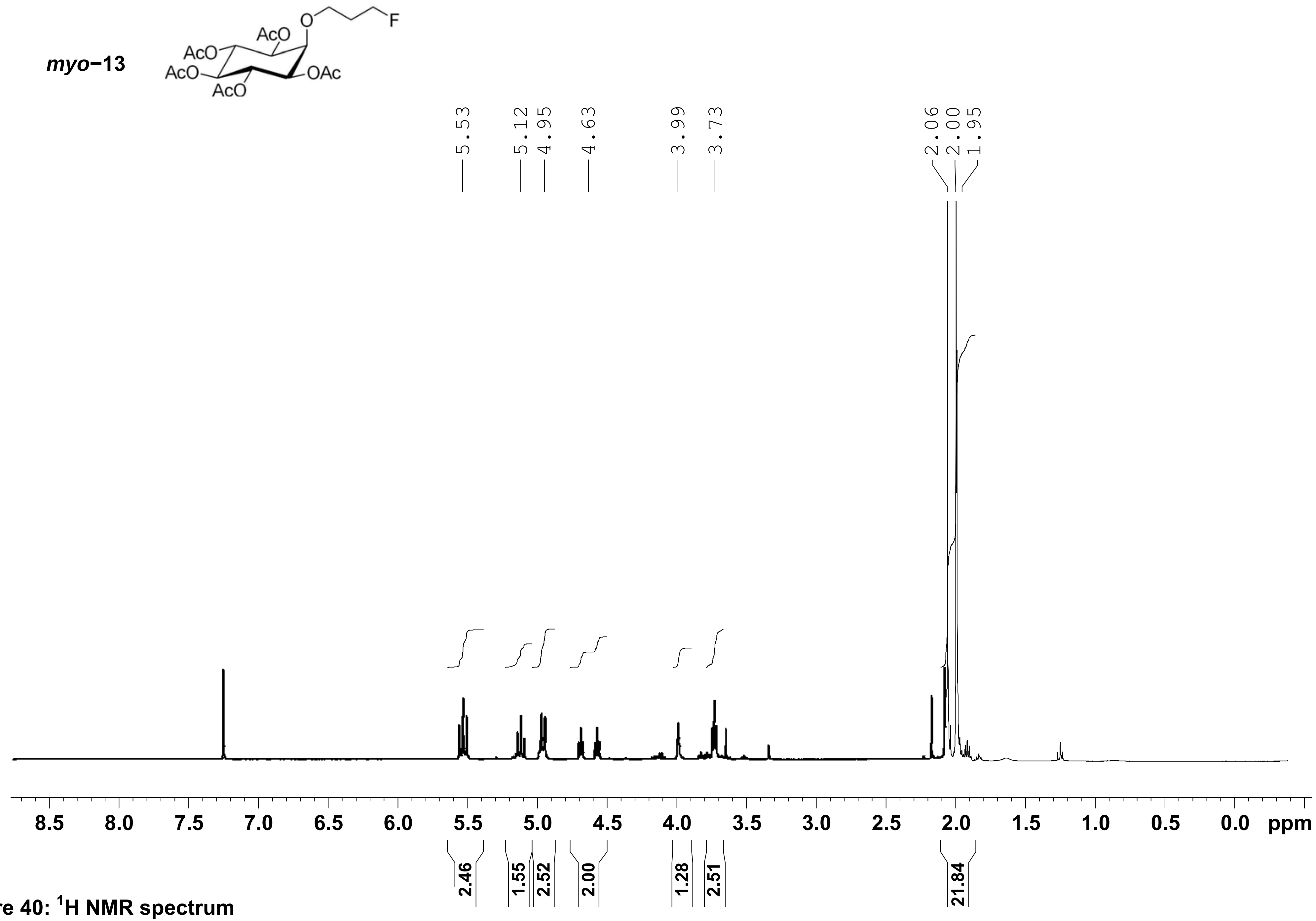

Figure 40: ^{1}H NMR spectrum

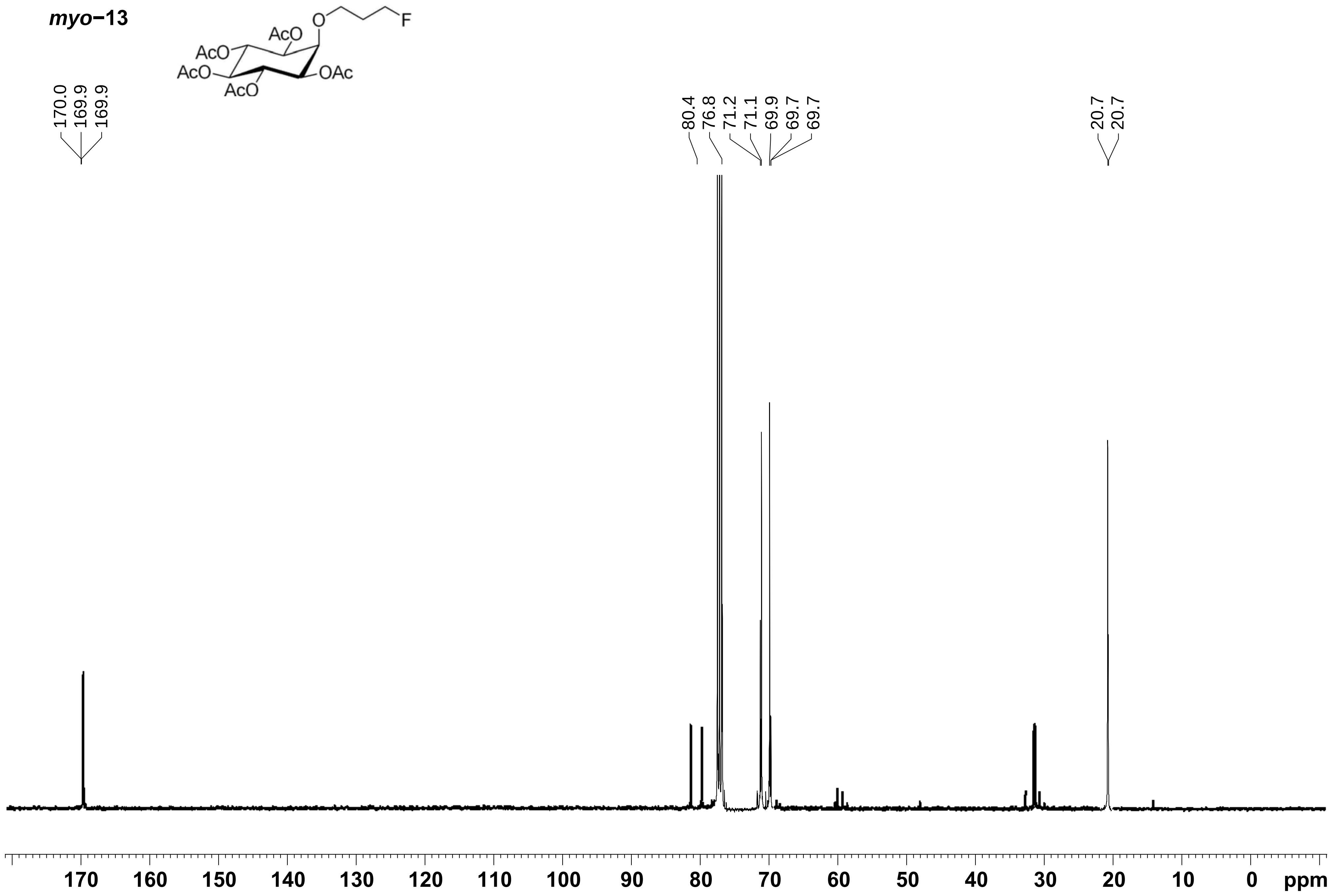

Figure 41: ^{13}C NMR spectrum

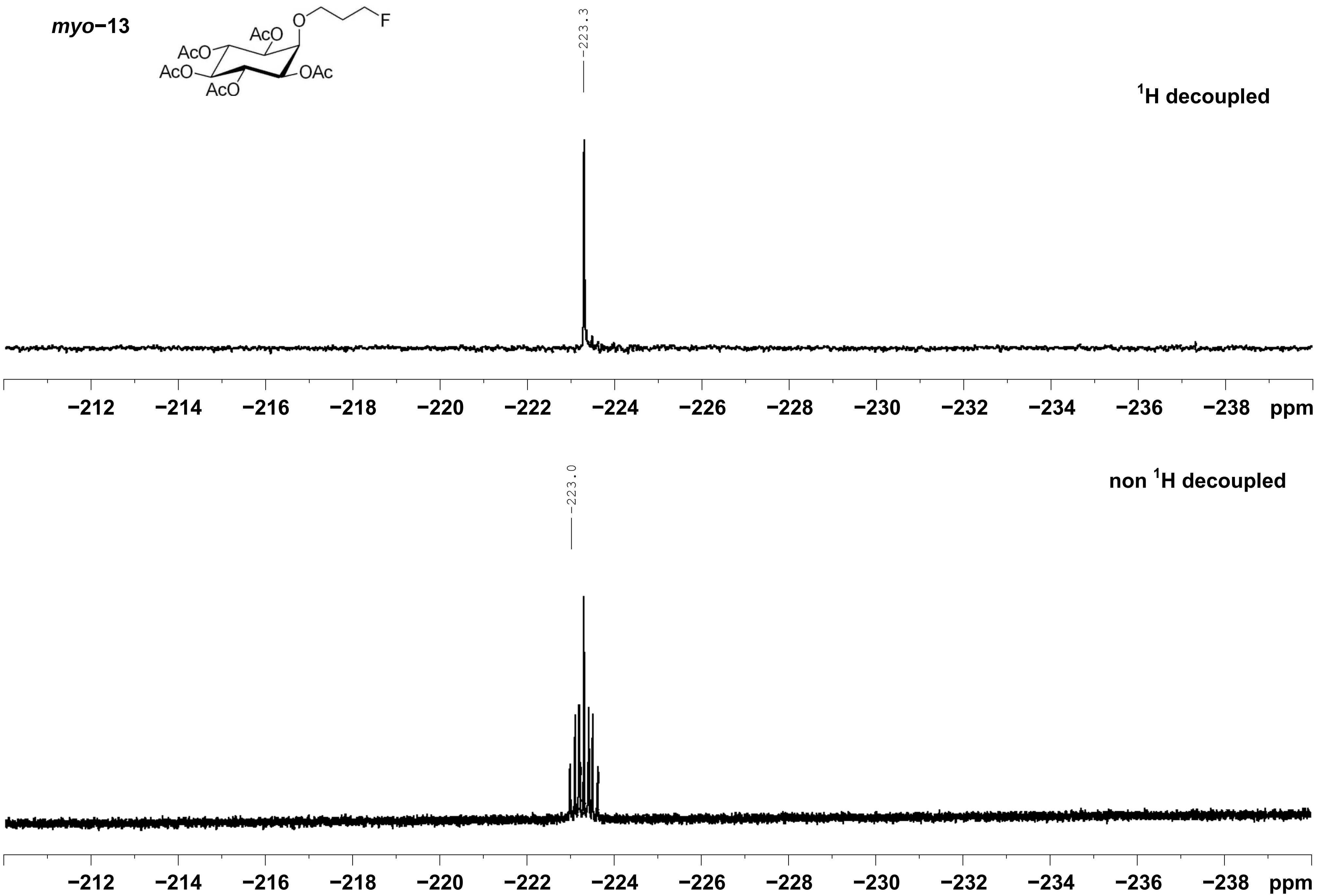

Figure 42: ^{19}F NMR spectrum

***scyllo*−13**

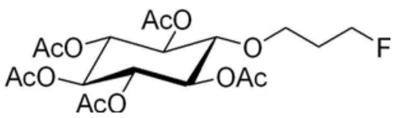

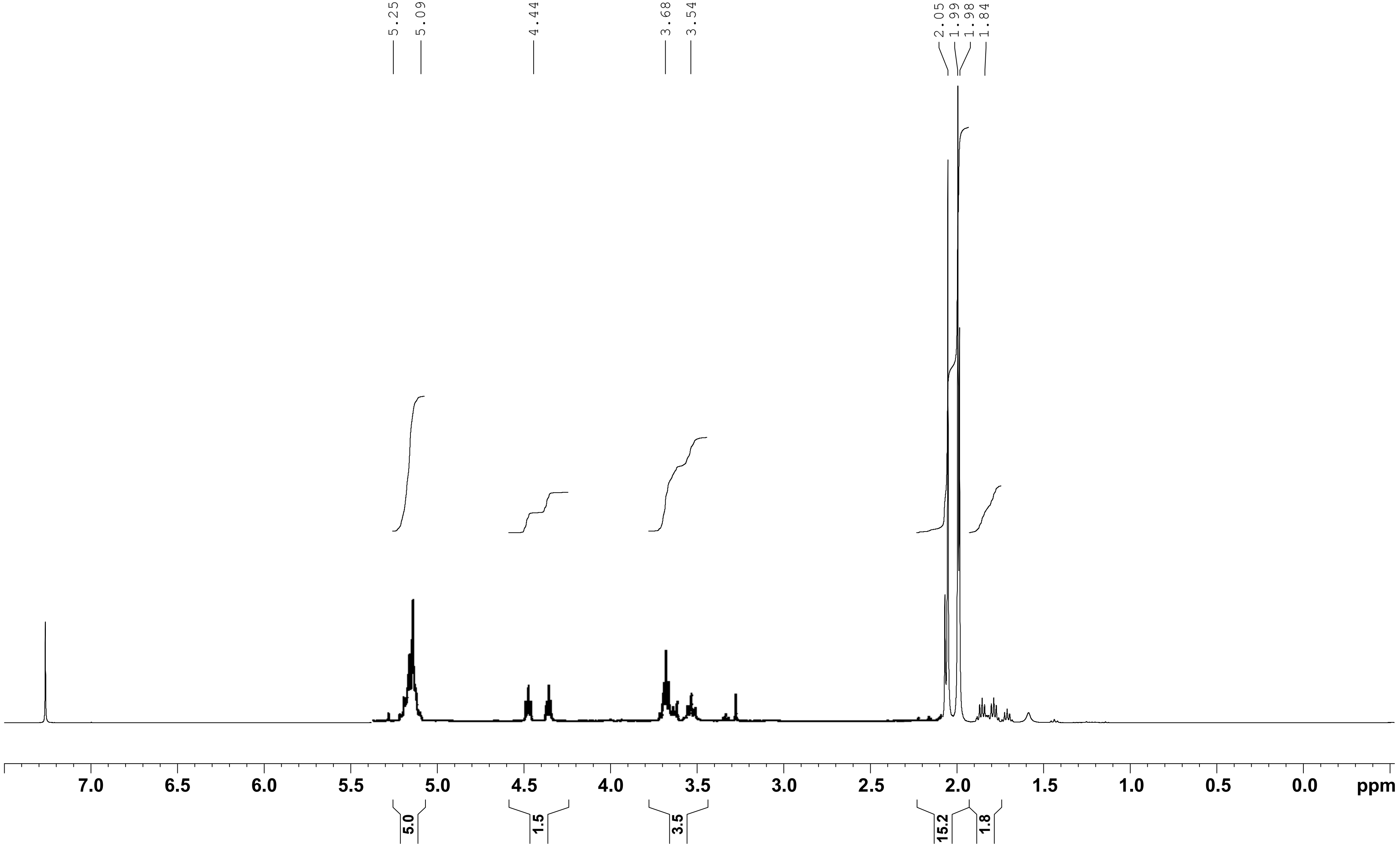

Figure 43: ^{1}H NMR spectrum

scyllo-**13**

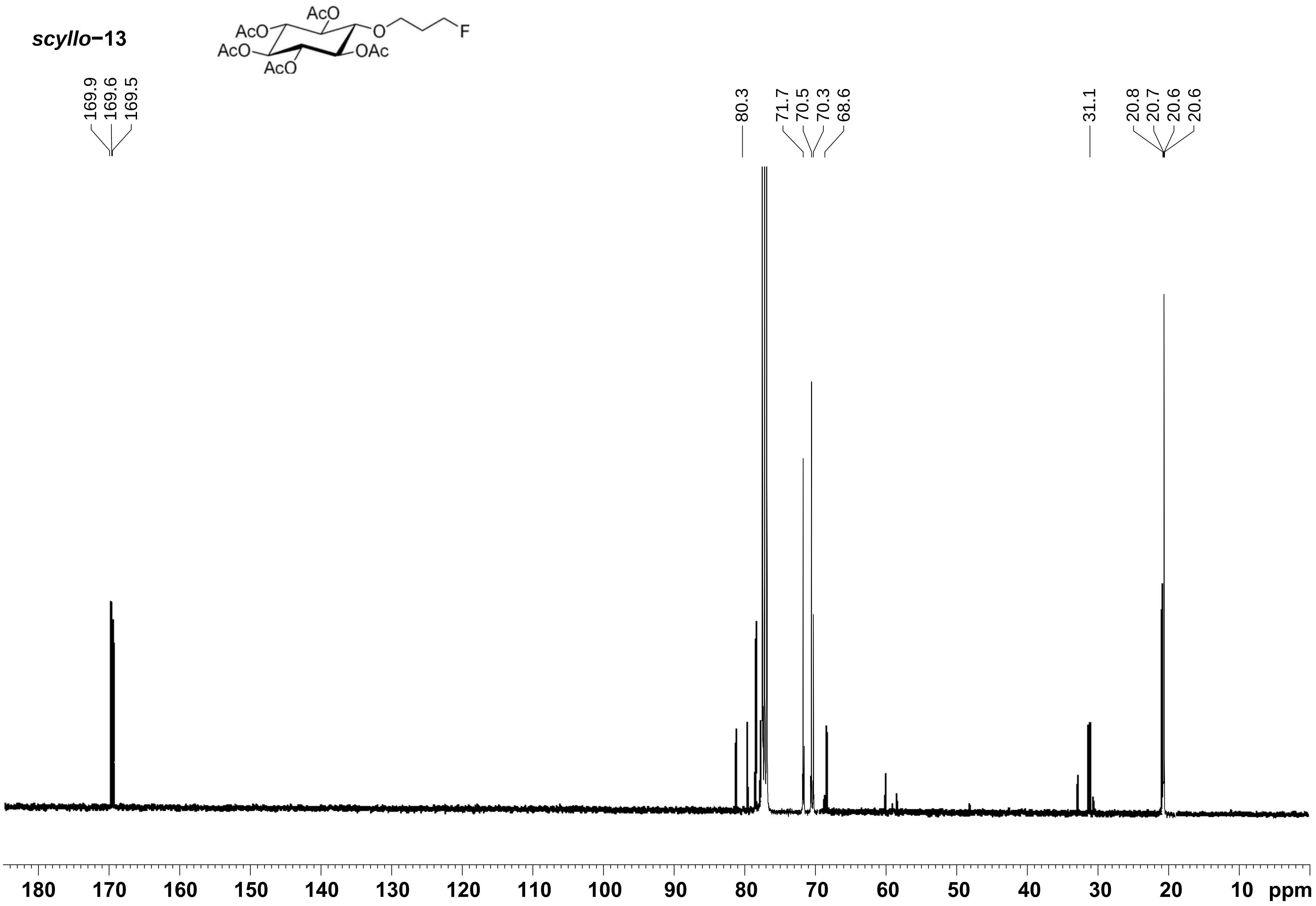

Figure 44: ^{13}C NMR spectrum

***scyllo*−13**

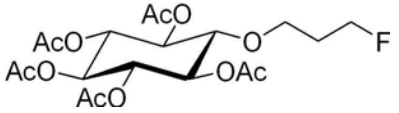

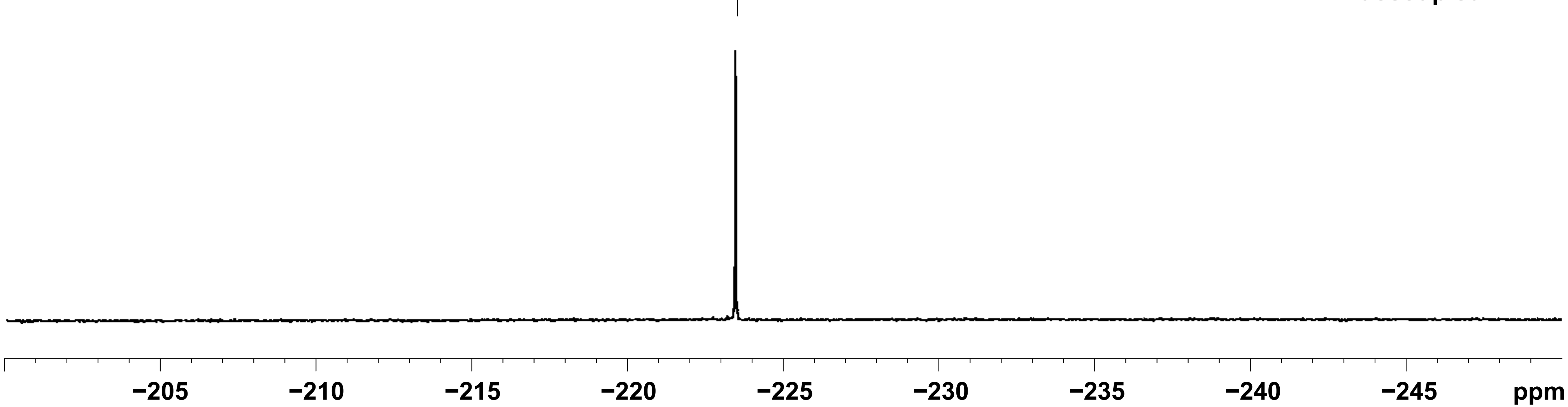

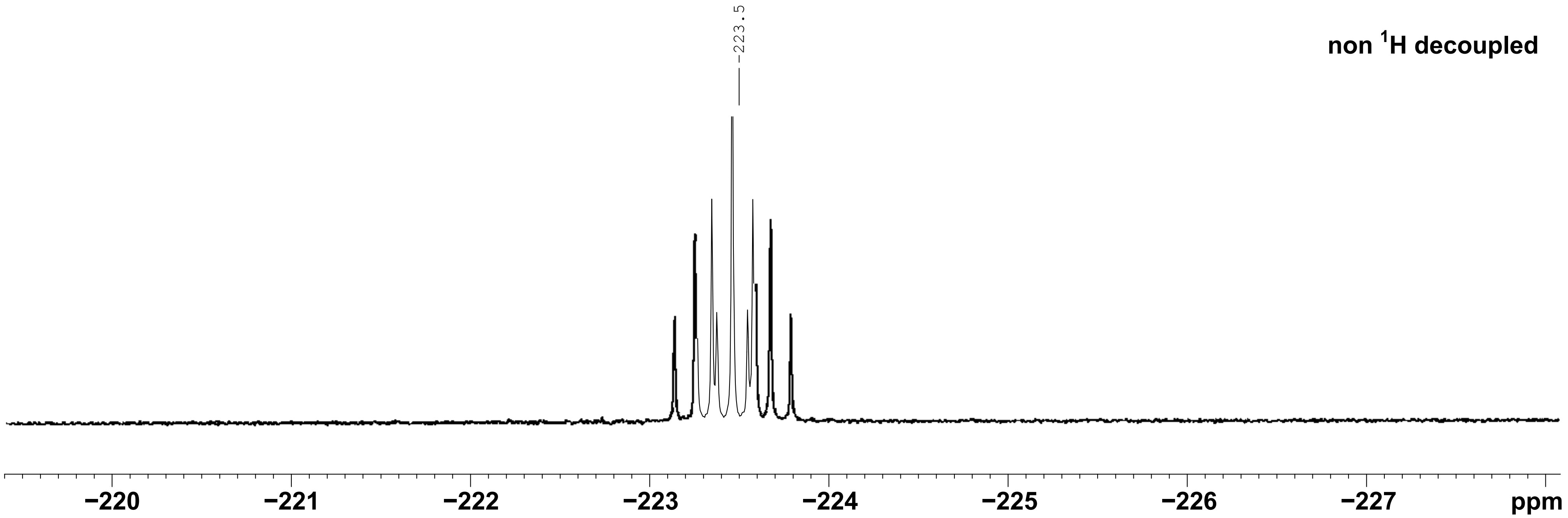

Figure 45: ^{19}F NMR spectrum

***myo*−14**

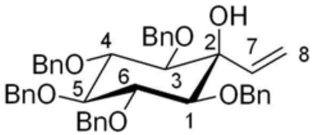

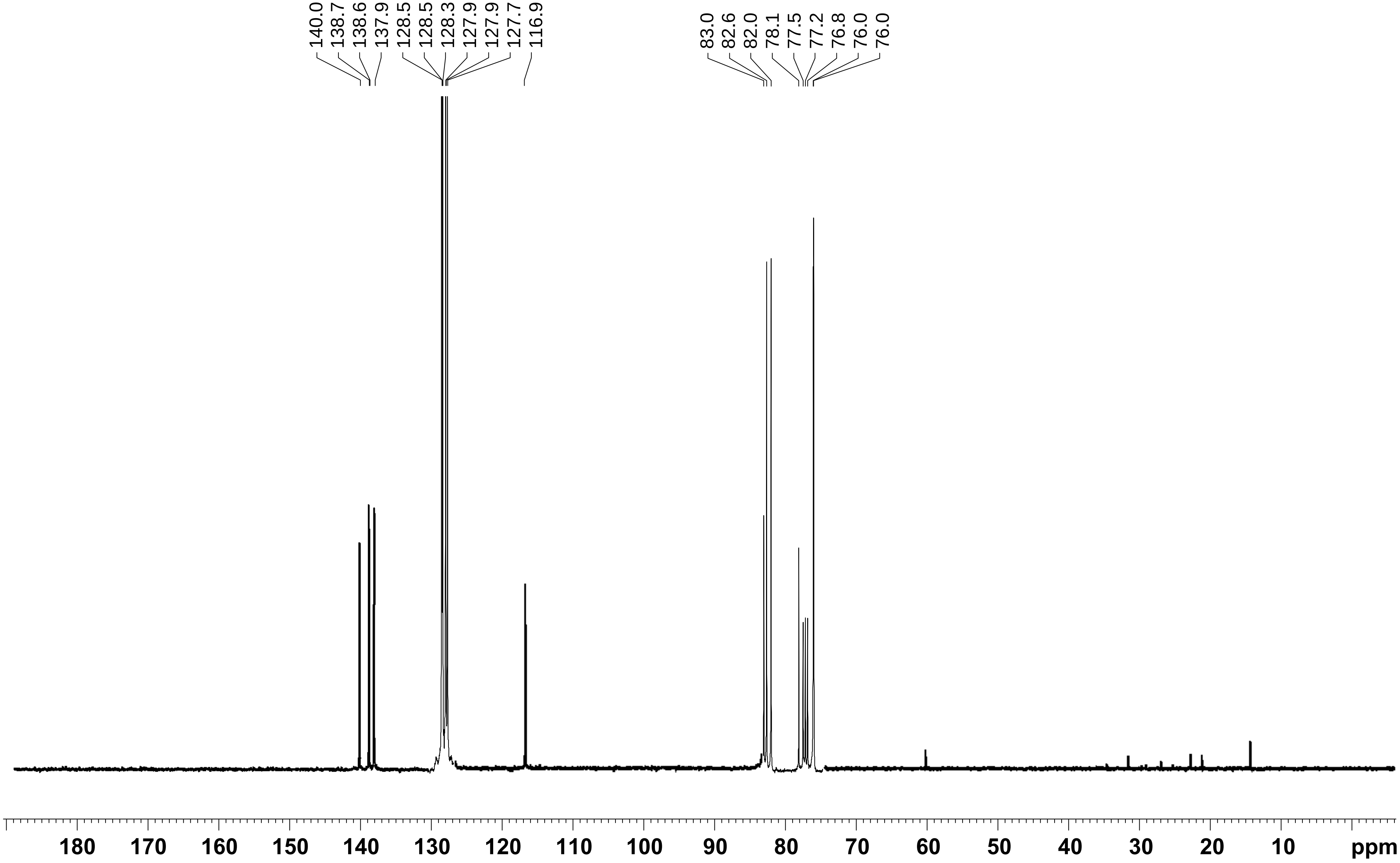

Figure 46: ^{13}C NMR spectrum

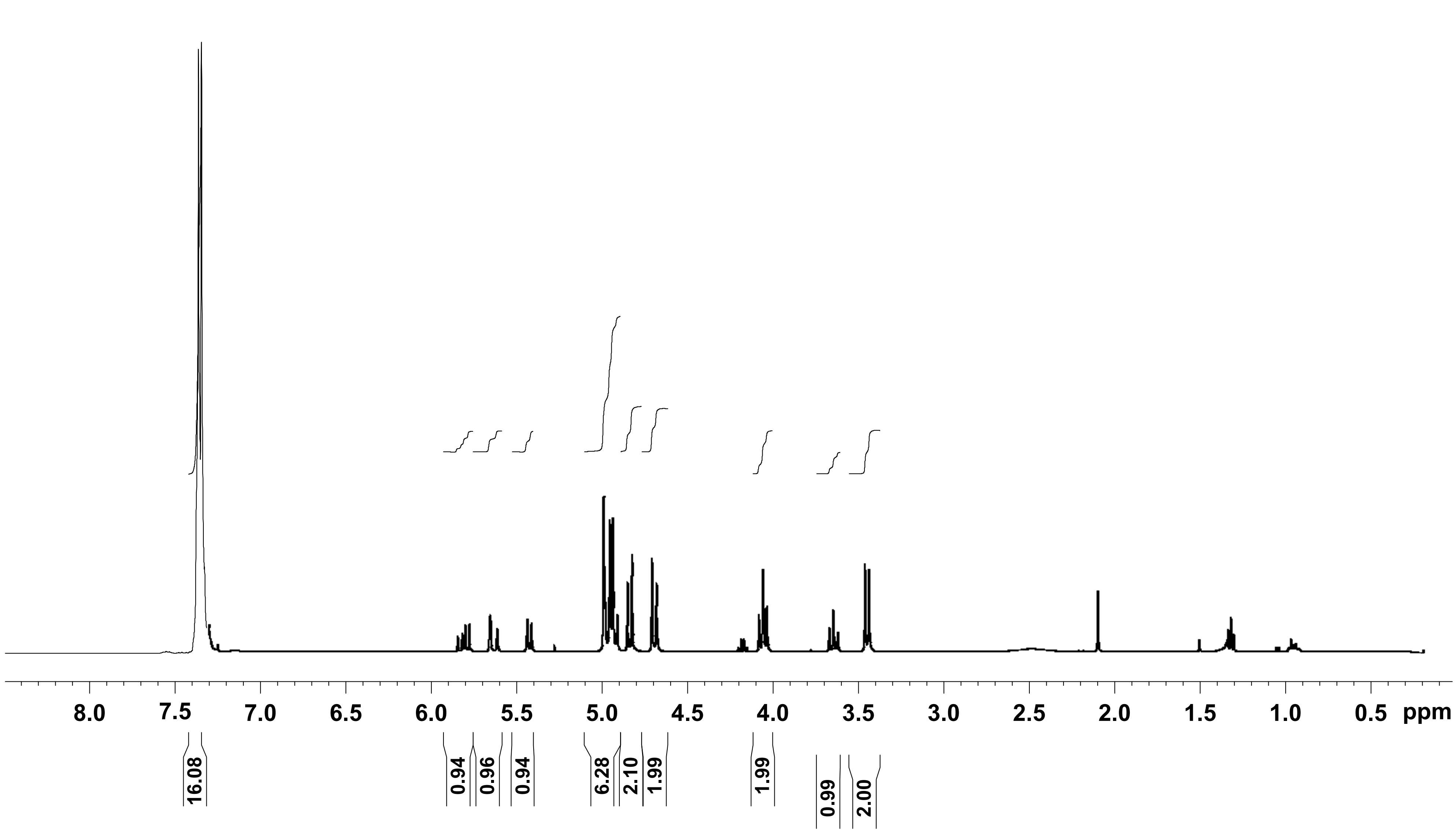

myo−**14**

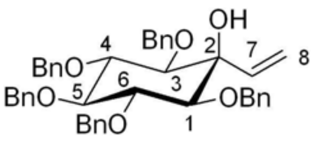

Figure 47: ^{1}H NMR spectrum

scyllo−**14**

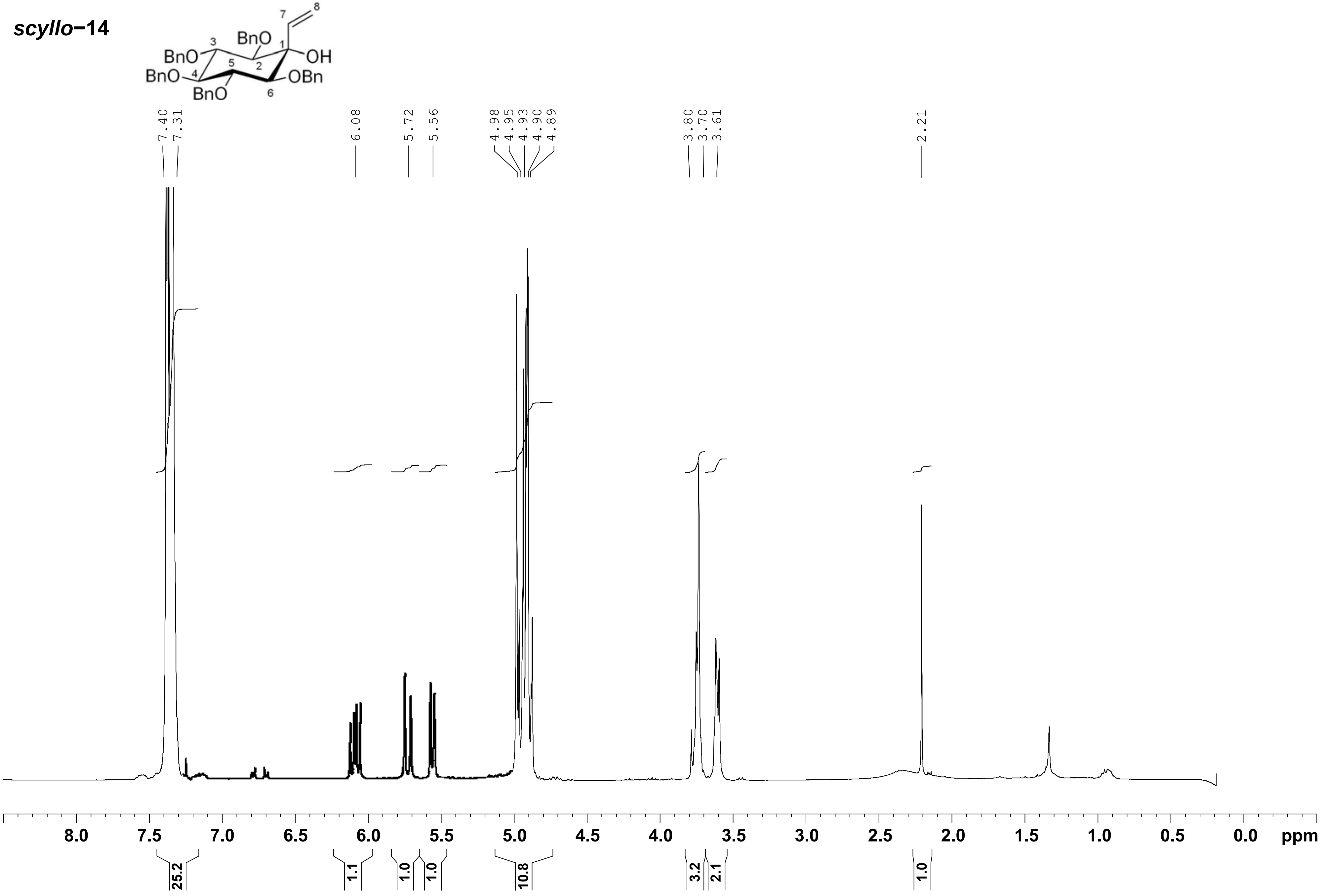

Figure 48: ^{1}H NMR spectrum

***Scyllo*−14**

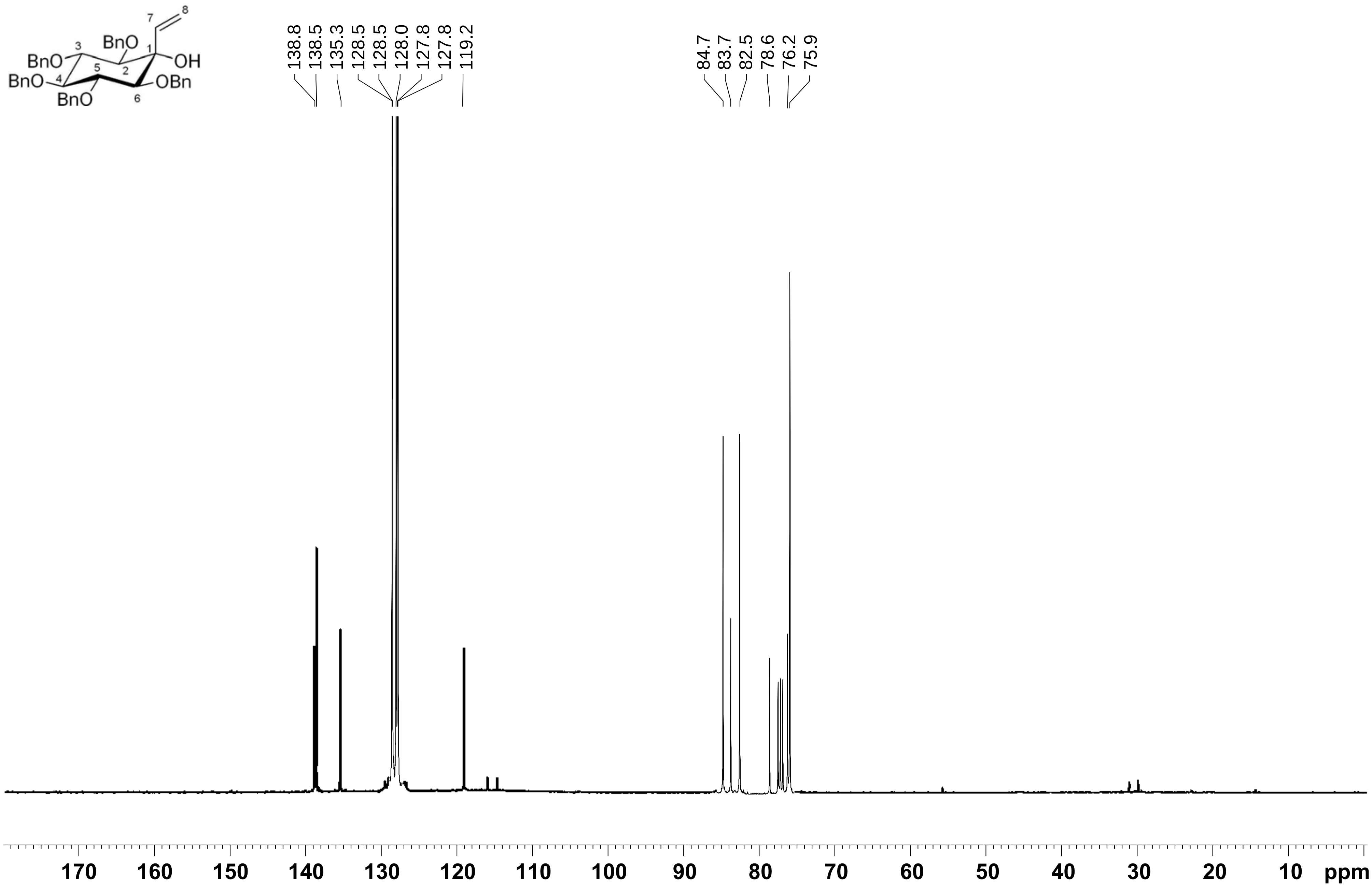

Figure 49: ^{13}C NMR spectrum

***myo*−15**

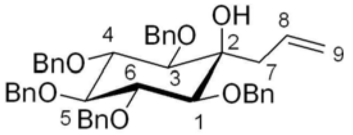

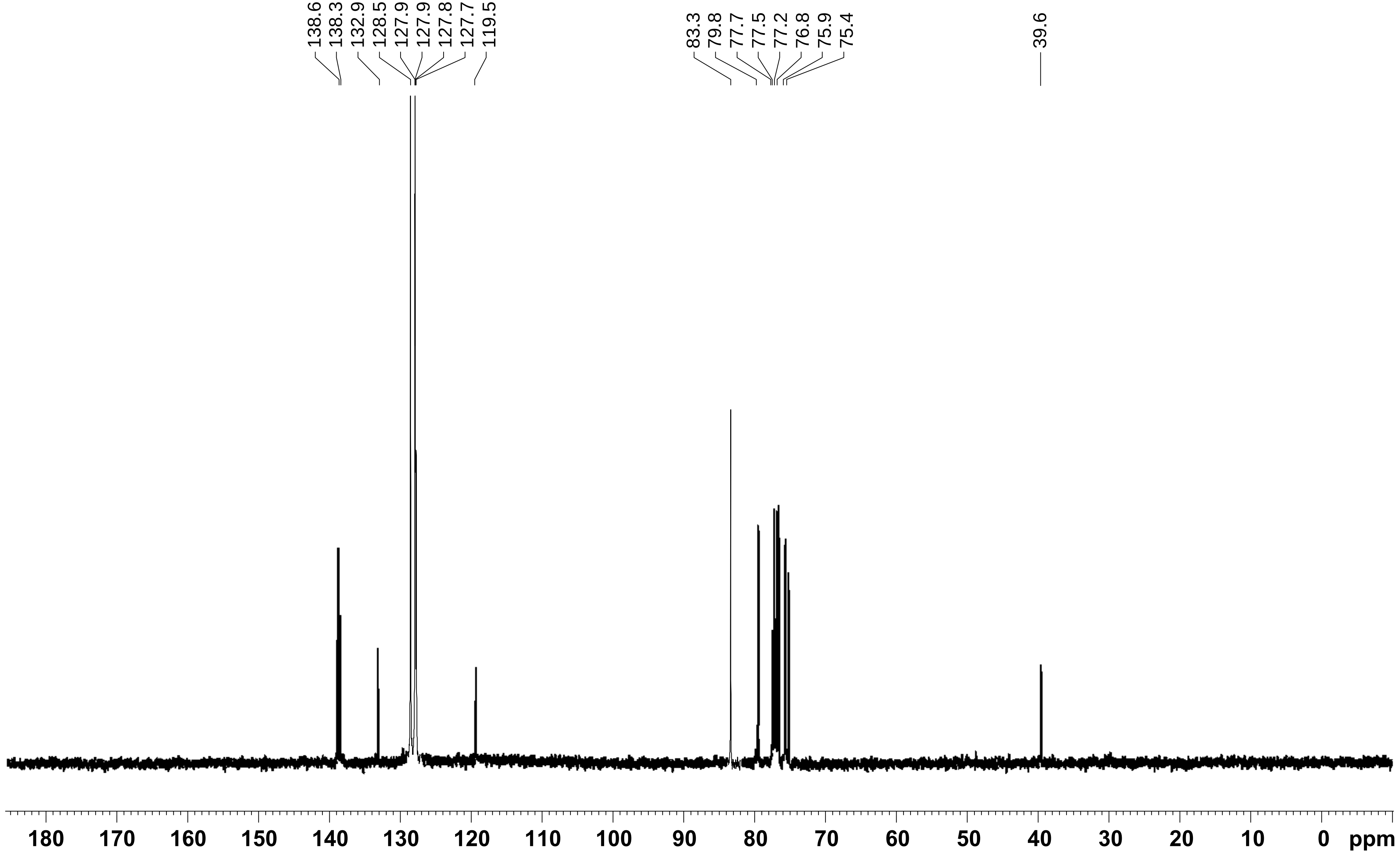

Figure 50: ^{13}C NMR spectrum

myo−15

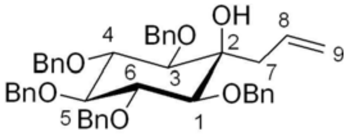

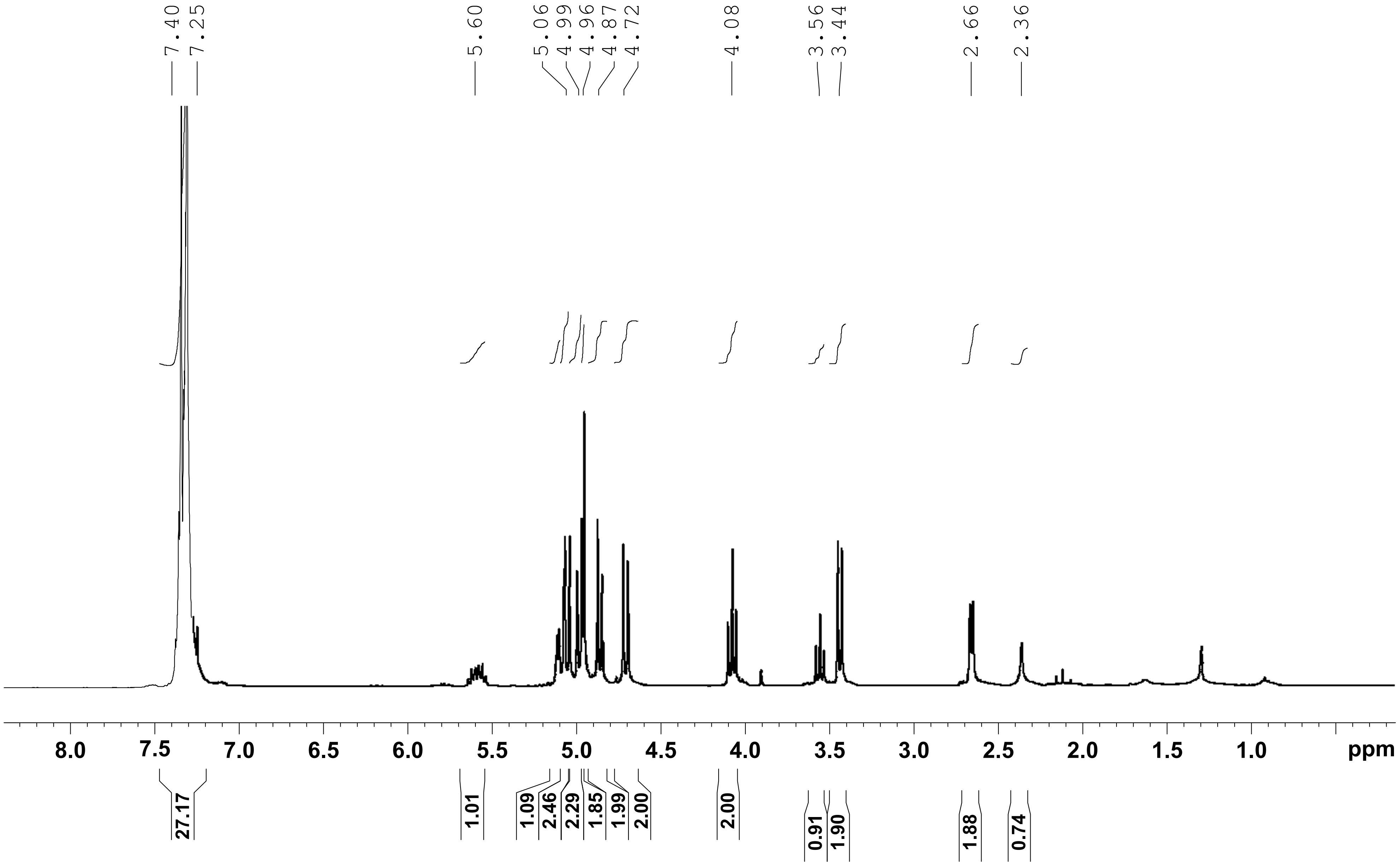

Figure 51: ^{1}H NMR spectrum

***scyllo*−15**

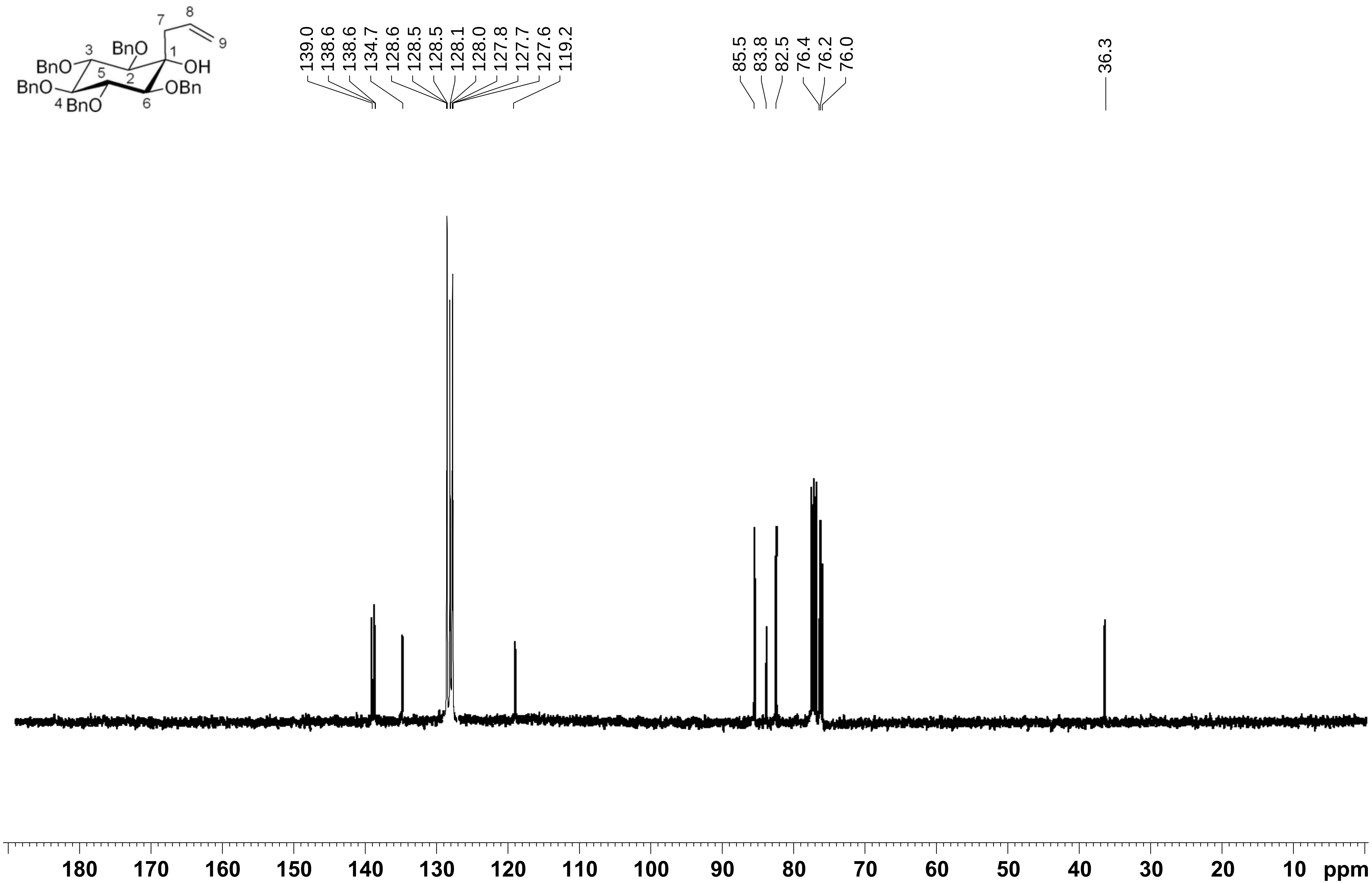

Figure 52: ^{13}C NMR spectrum

scyllo−**15**

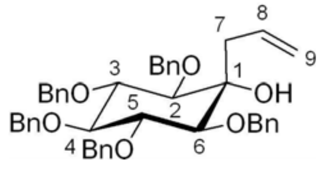

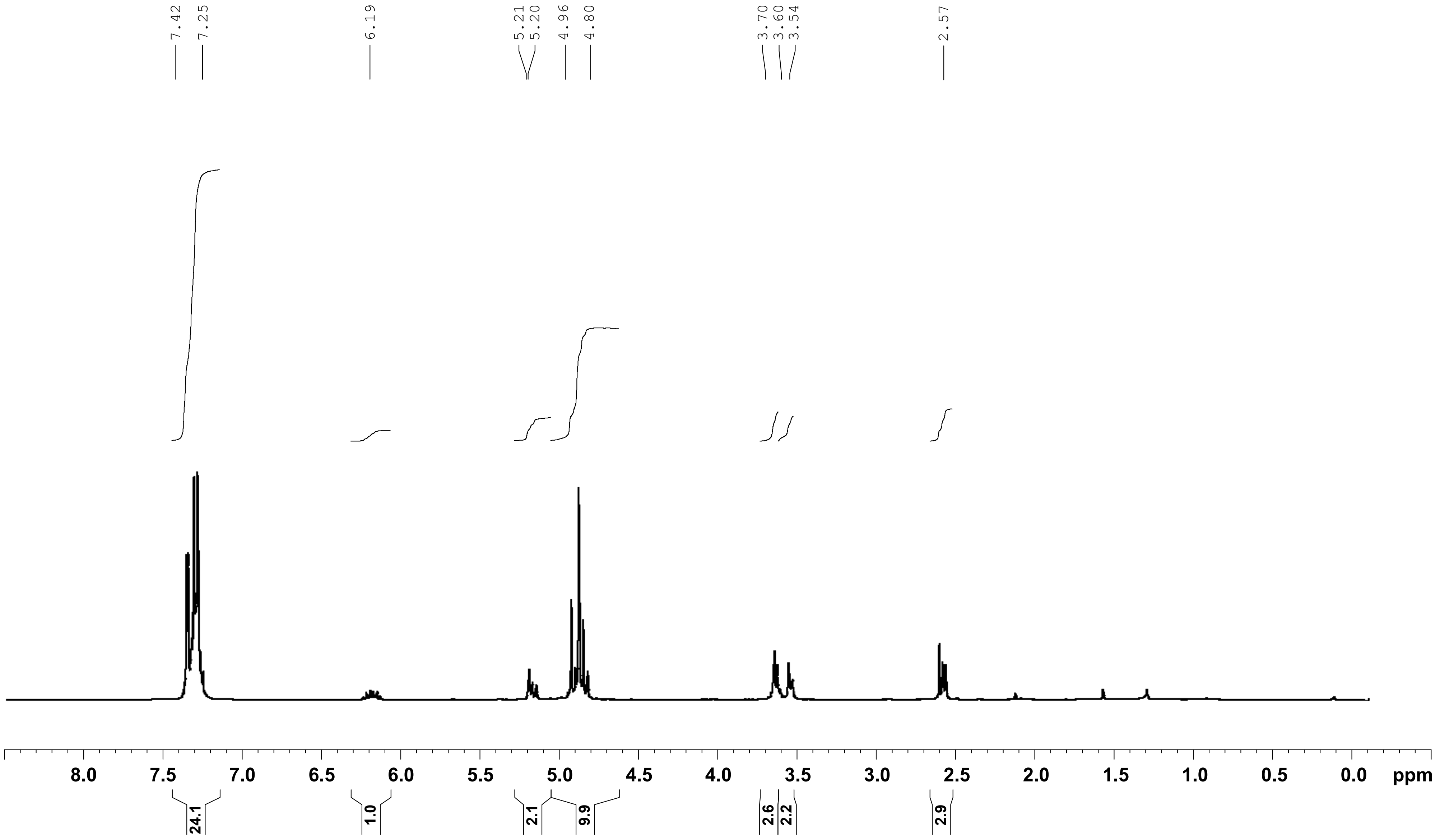

Figure 53: ¹H NMR spectrum

***myo*−16**

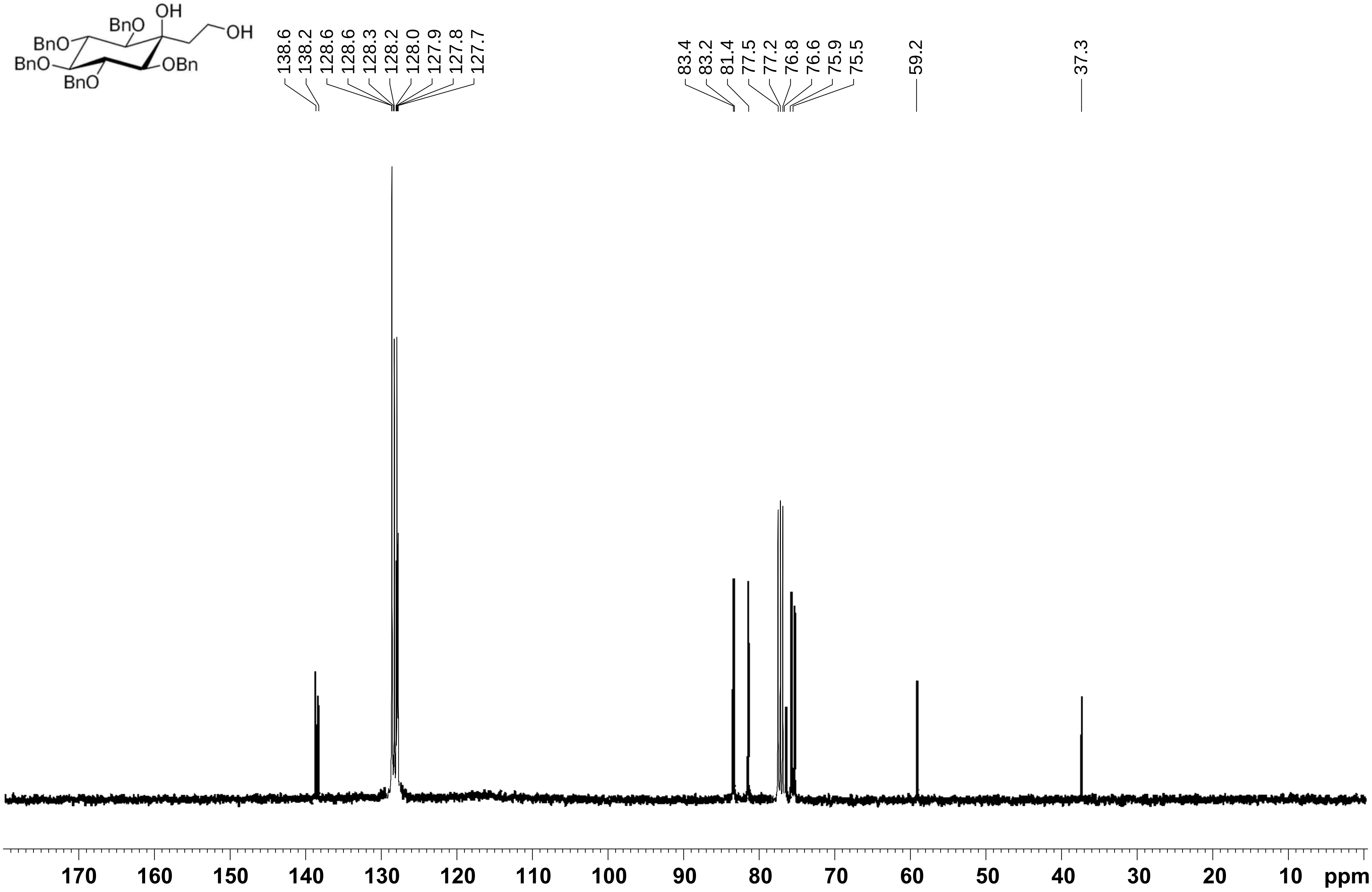

Figure 54: ^{13}C NMR spectrum

***myo*−16**

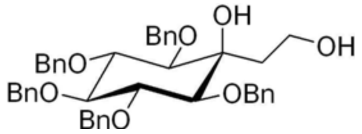

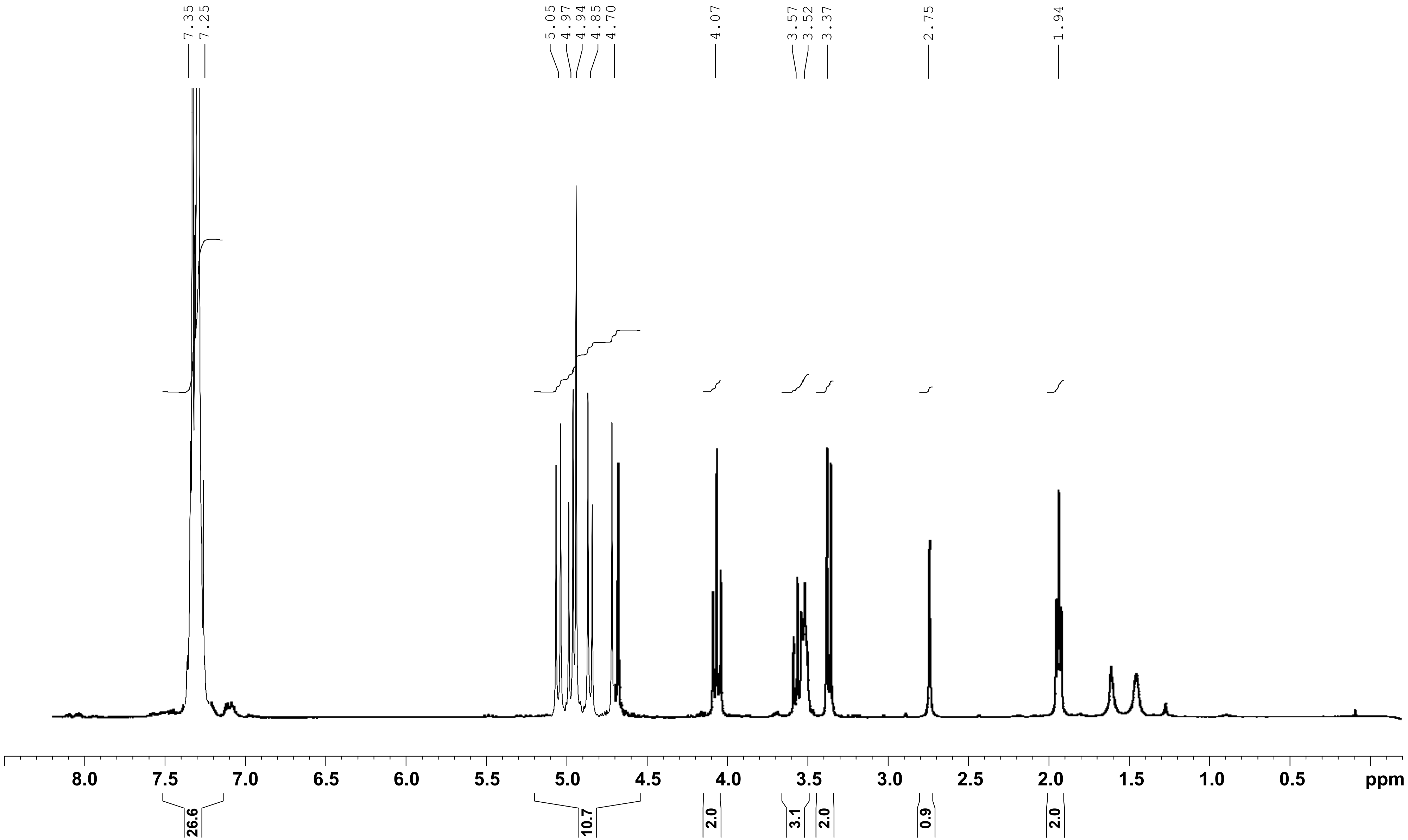

Figure 55: ^{1}H NMR spectrum

myo−**17**

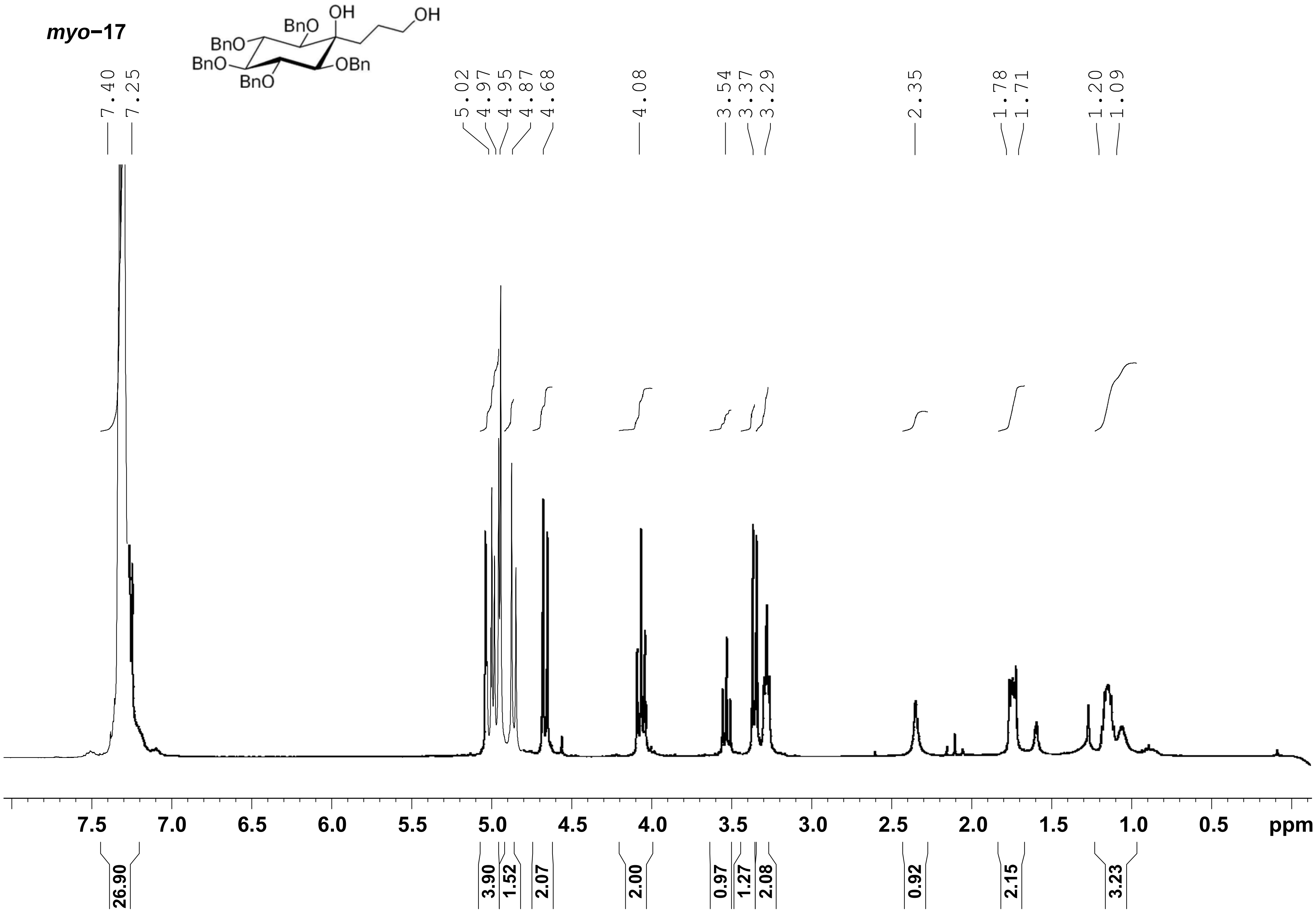

Figure 56: ^{1}H NMR spectrum

myo−**17**

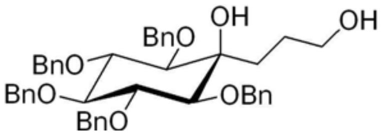

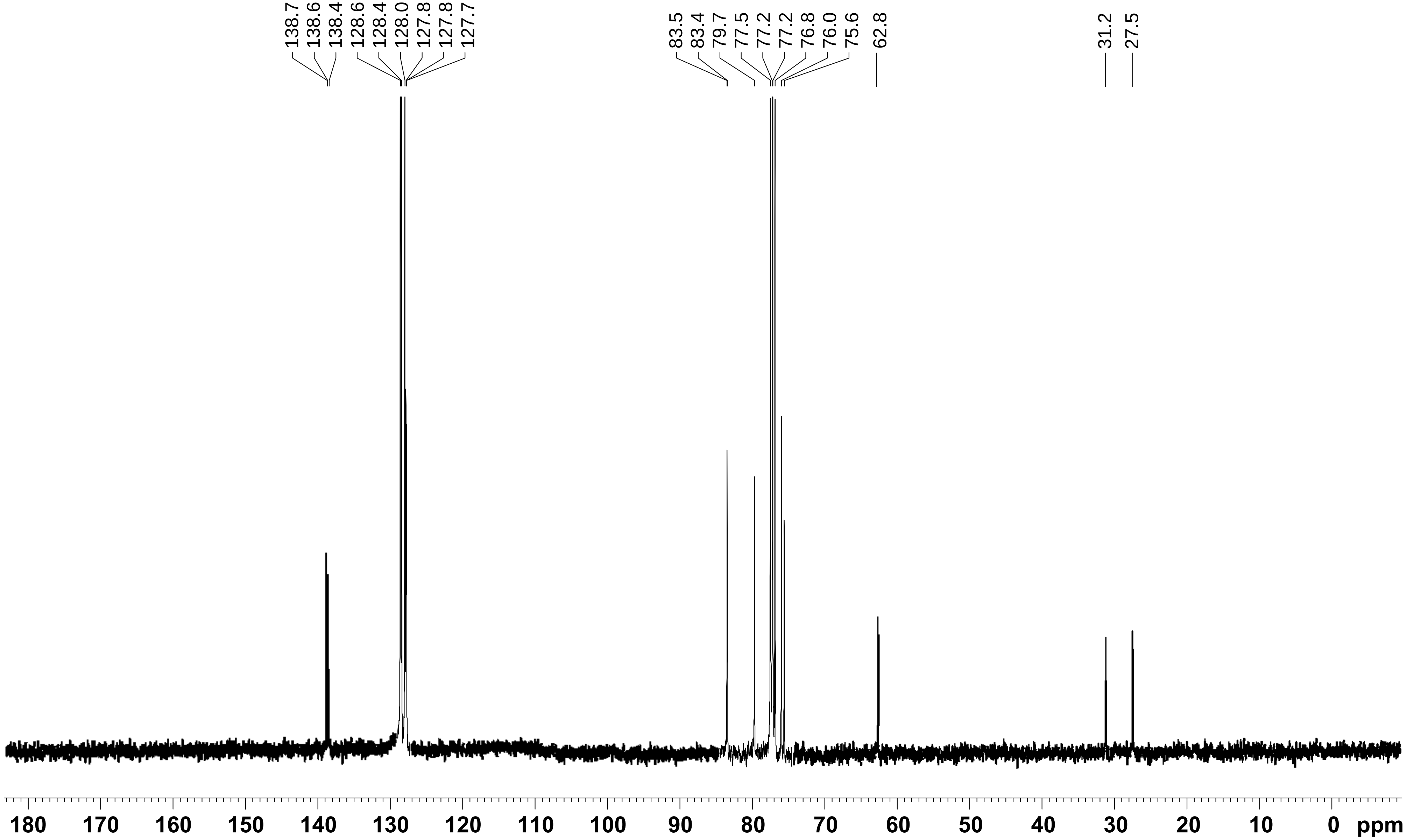

Figure 57: ^{13}C NMR spectrum

scyllo−17

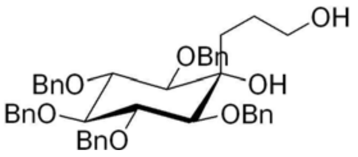

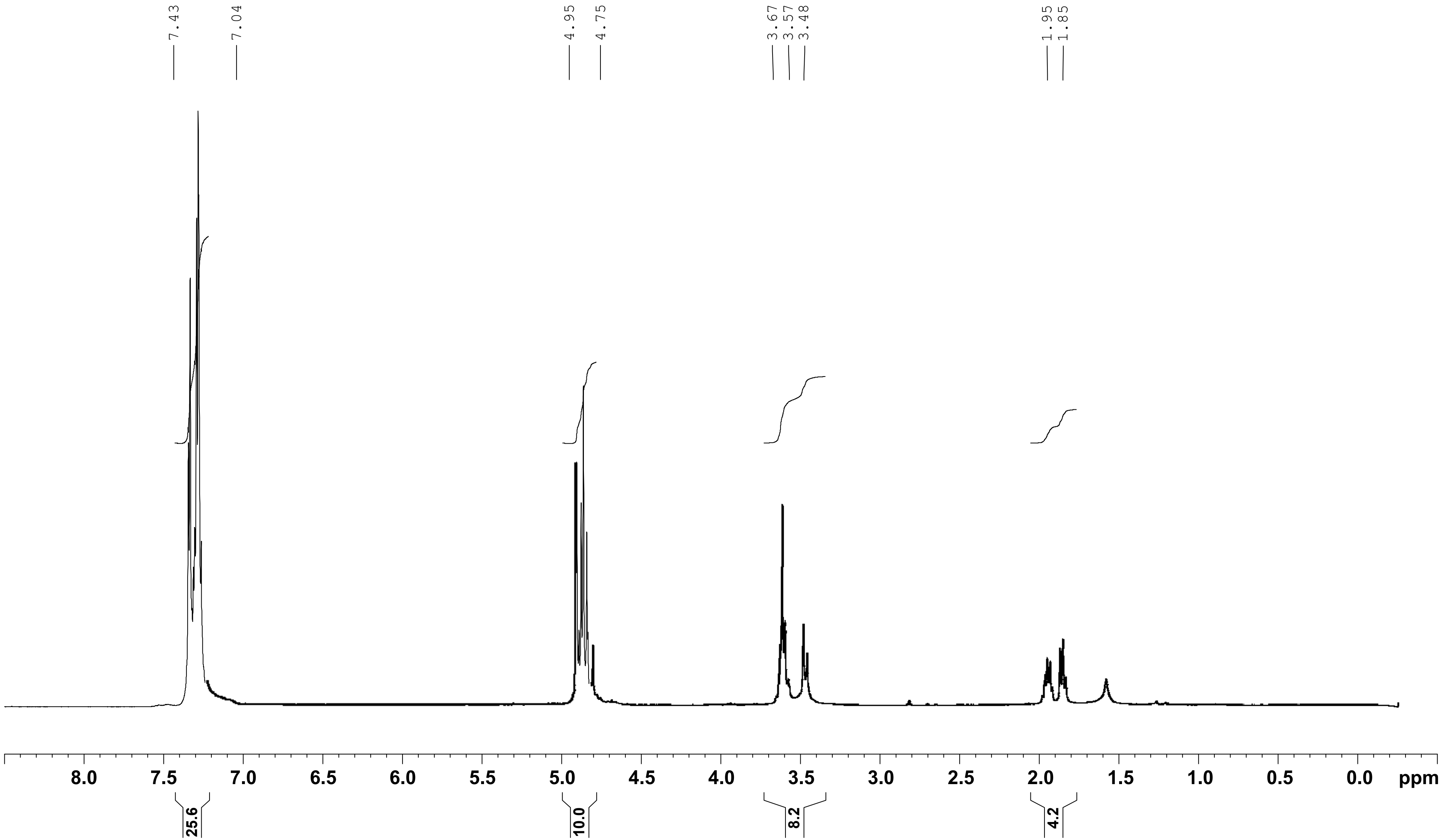

Figure 58: ^{1}H NMR spectrum

***scyllo*−17**

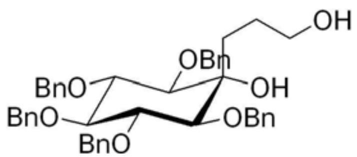

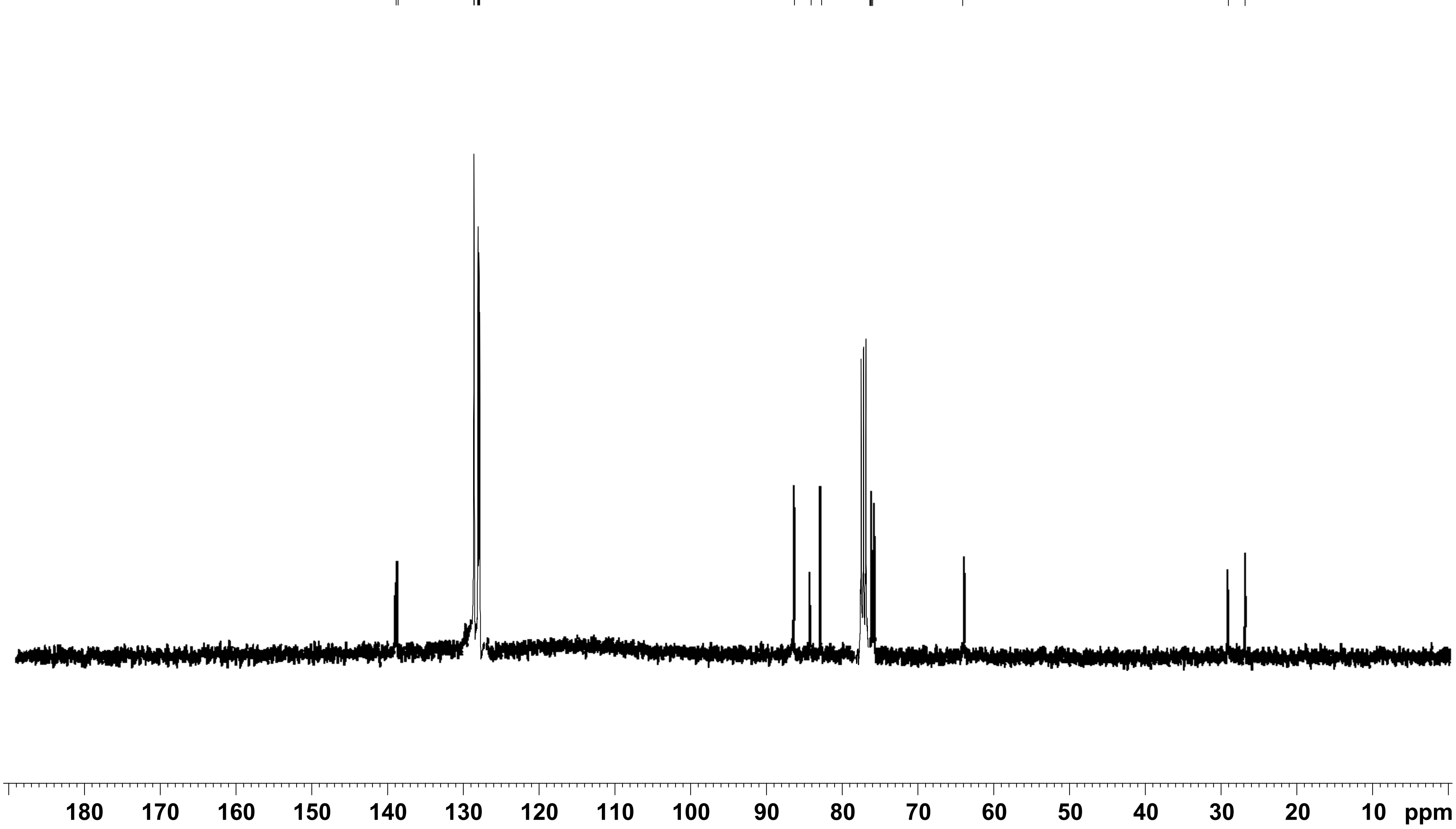

Figure 59: ^{13}C NMR spectrum

myo−**18**

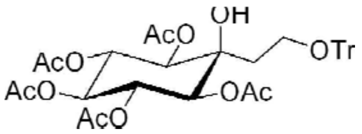

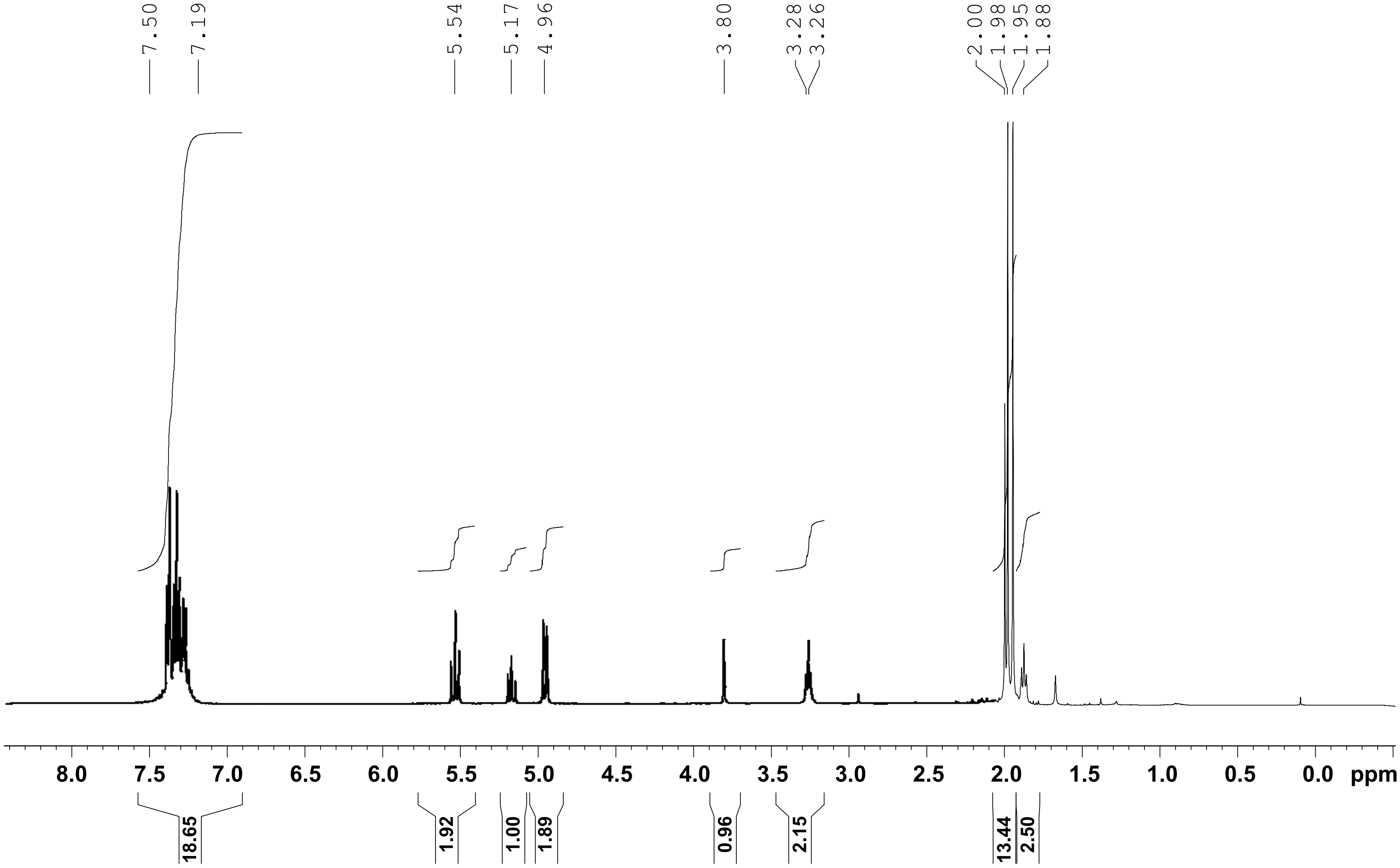

Figure 60: ^{1}H NMR spectrum

***myo*-18**

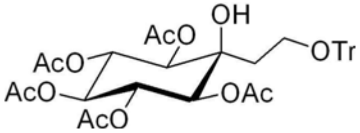

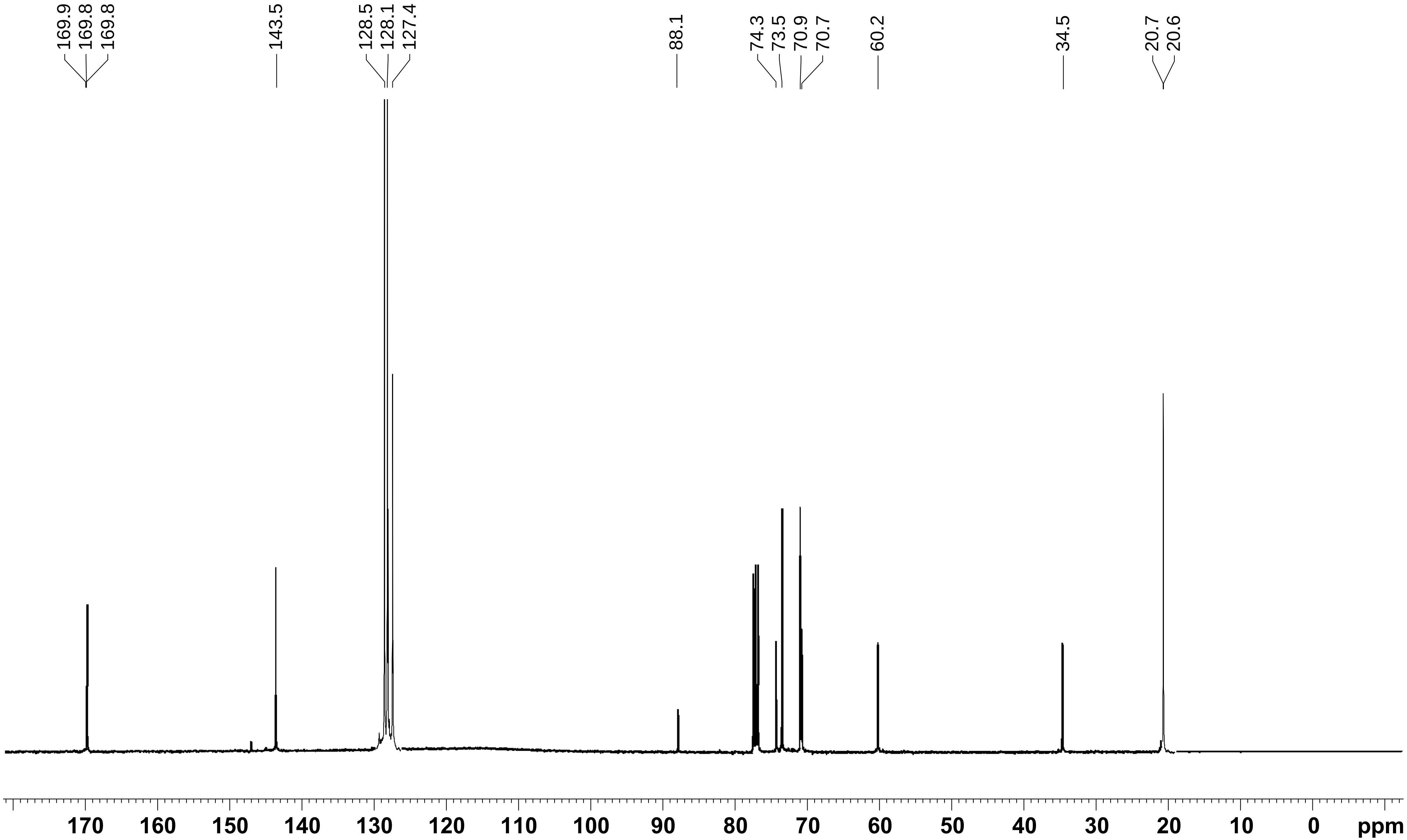

Figure 61: ^{13}C NMR spectrum

***scyllo*−18**

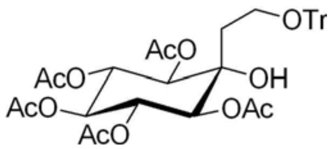

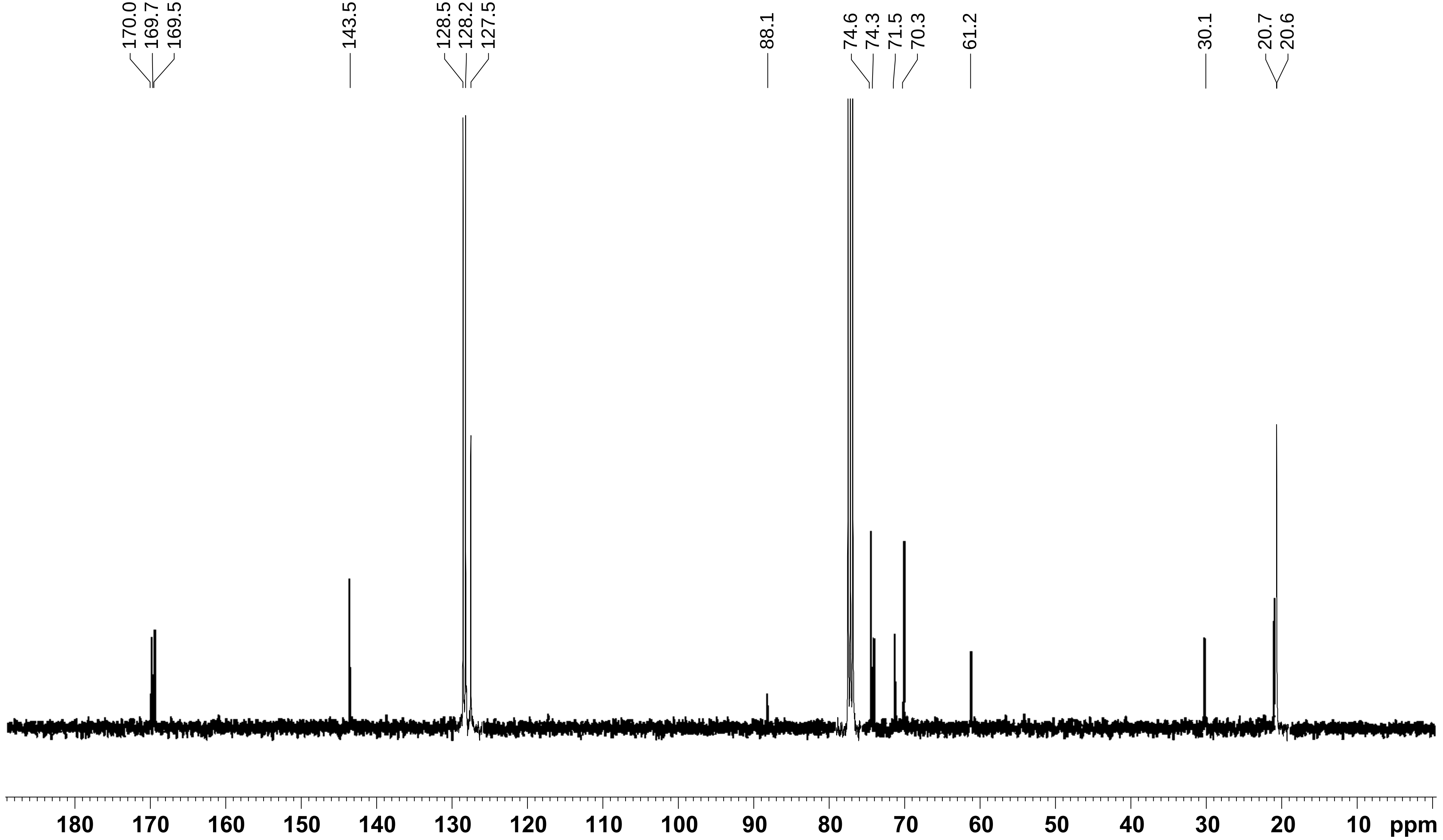

Figure 62: ^{13}C NMR spectrum

scyllo−18

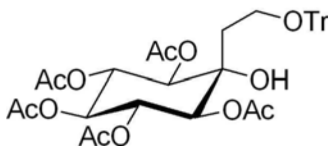

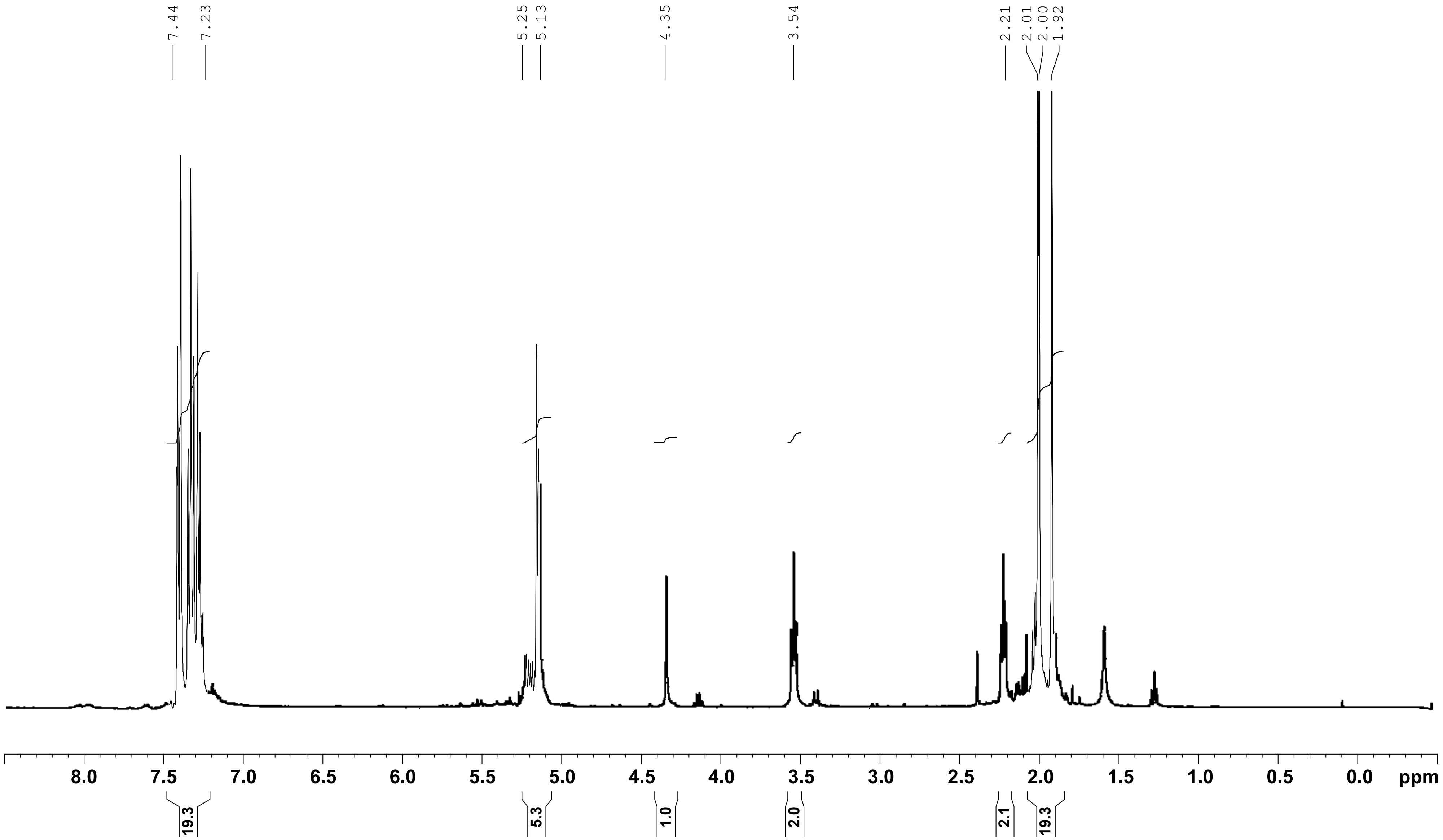

Figure 63: ^{1}H NMR spectrum

***myo*–20**

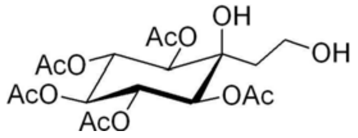

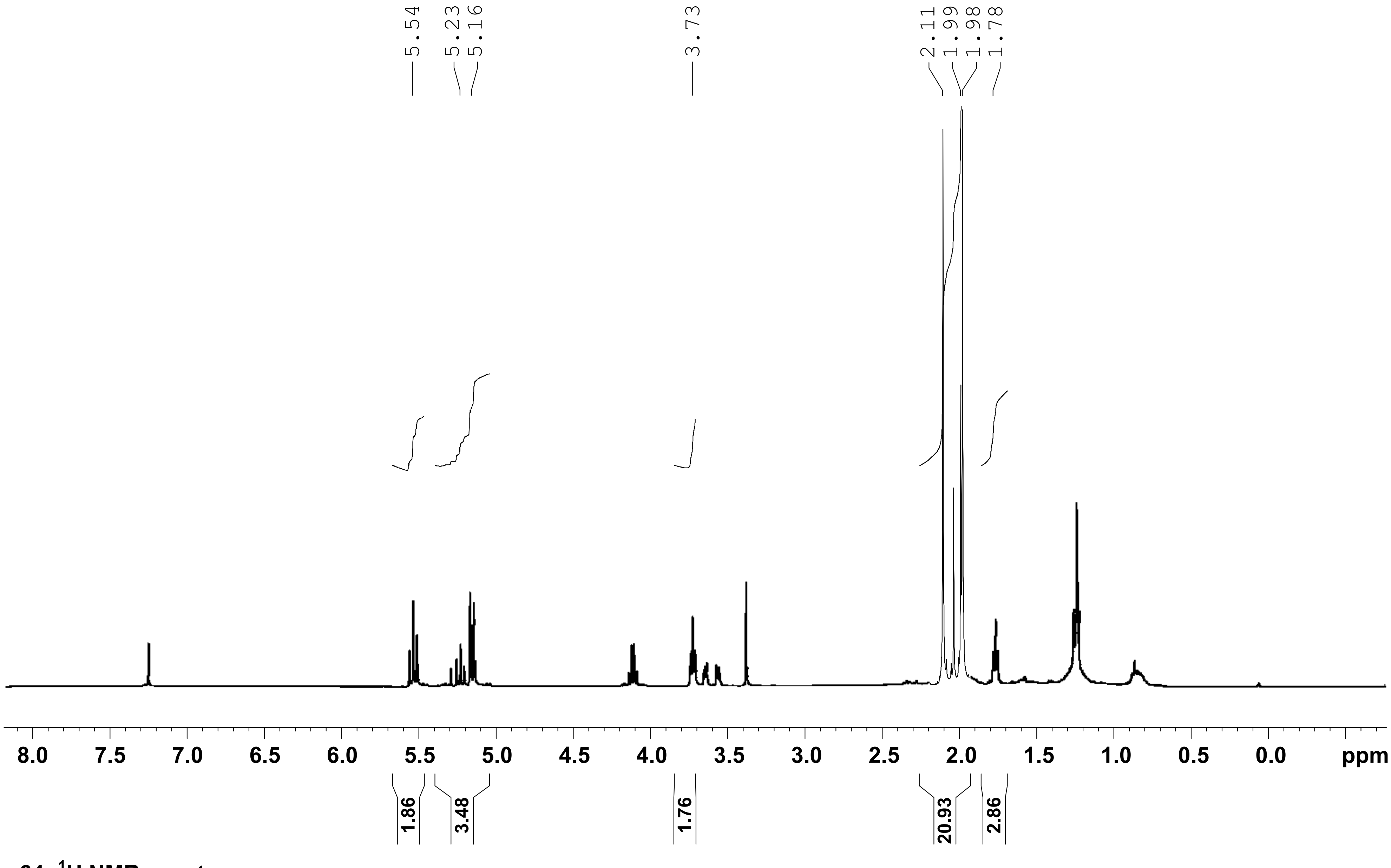

Figure 64: ^{1}H NMR spectrum

myo−**20**

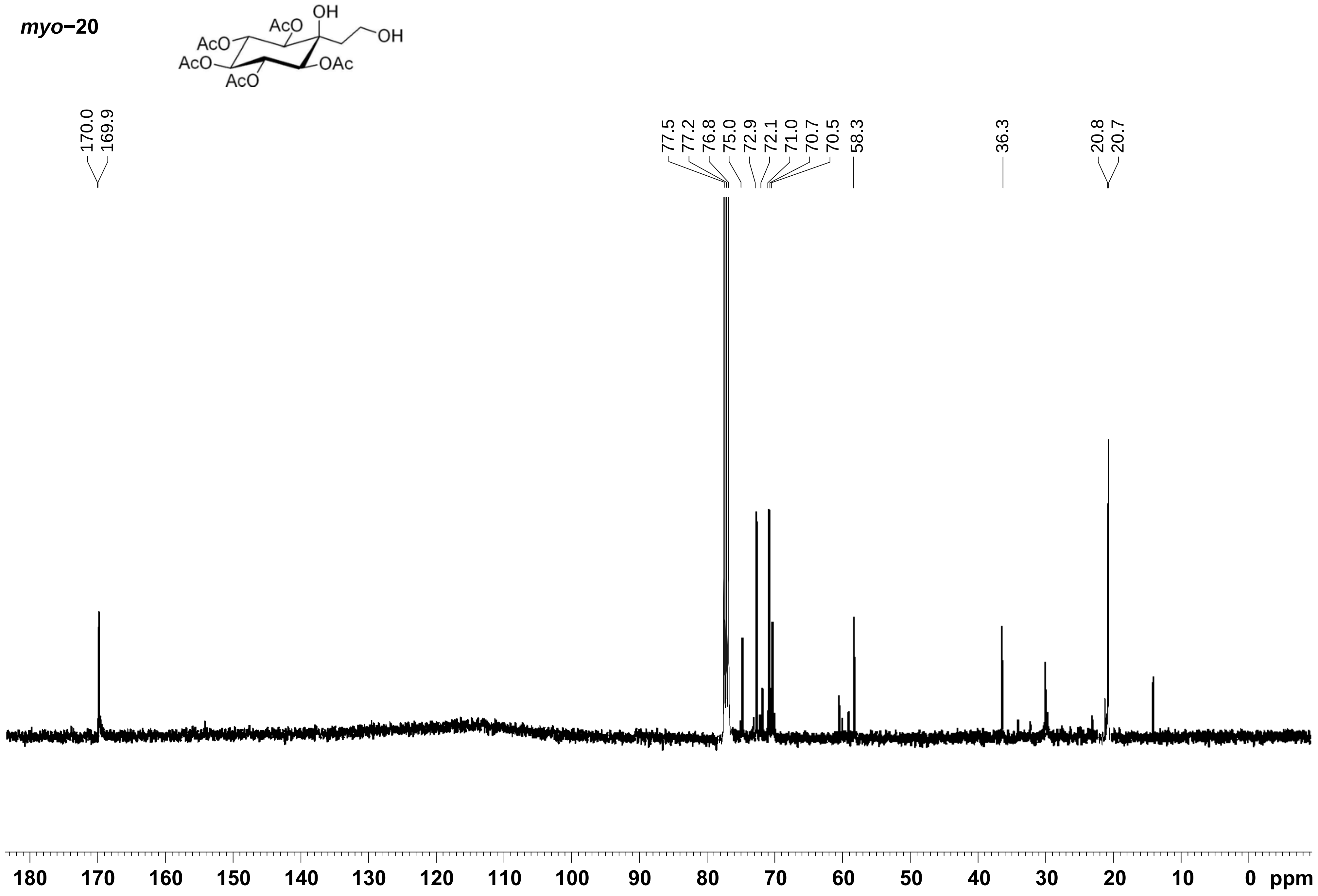

Figure 65: ^{13}C NMR spectrum

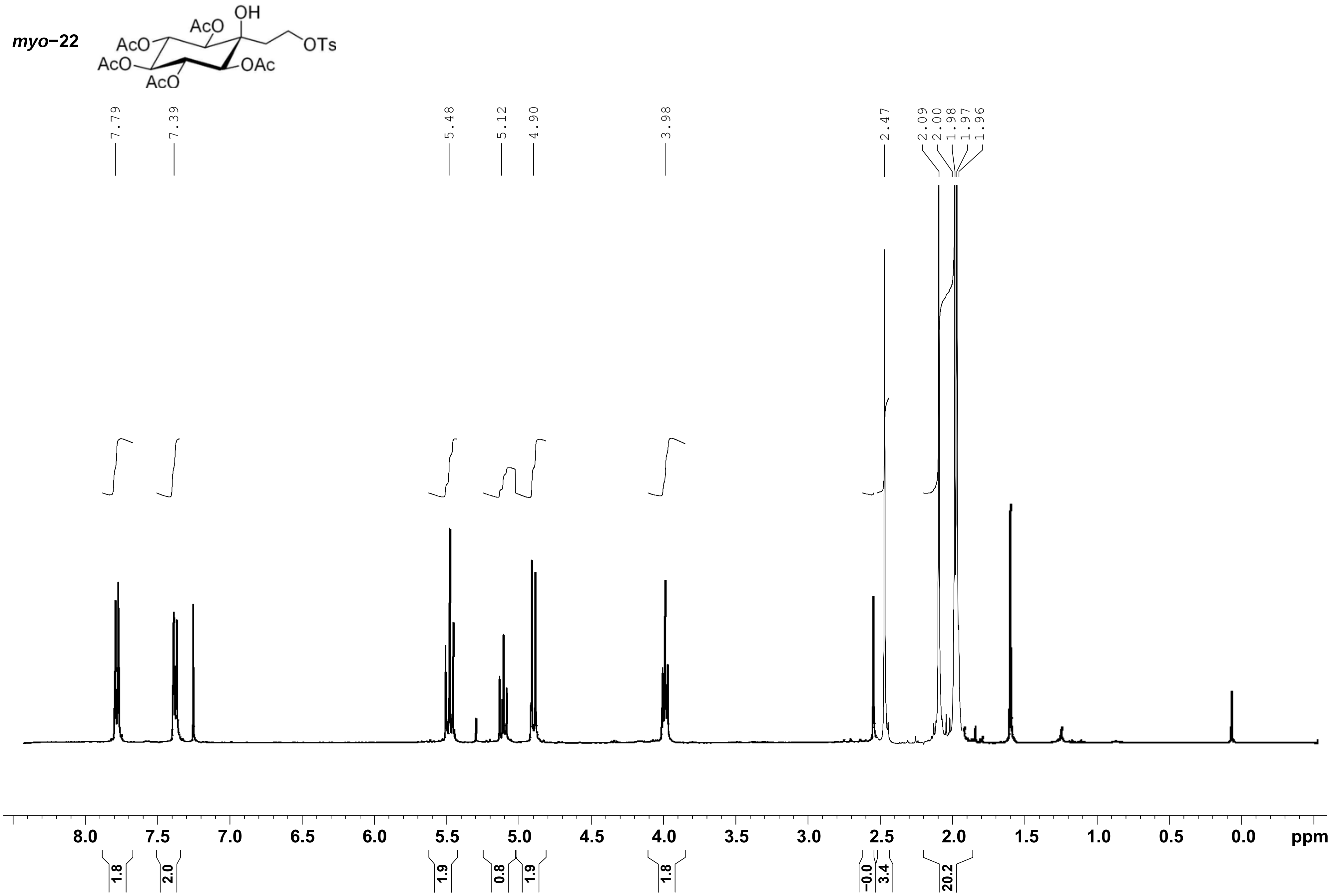

Figure 66: ^{1}H NMR spectrum

myo−22

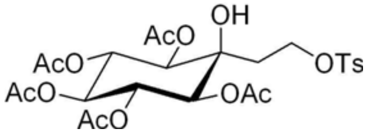

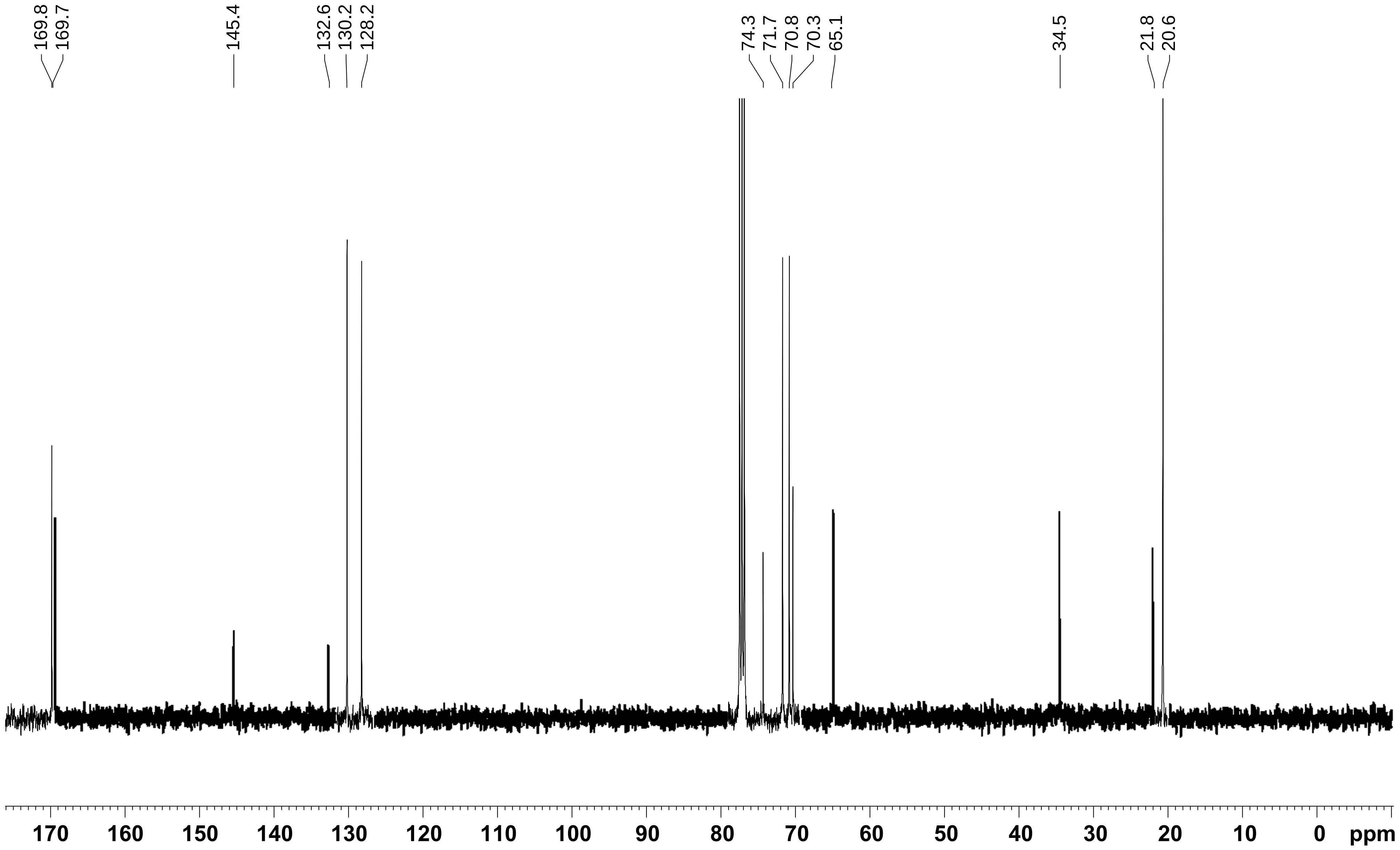

Figure 67: ^{13}C NMR spectrum

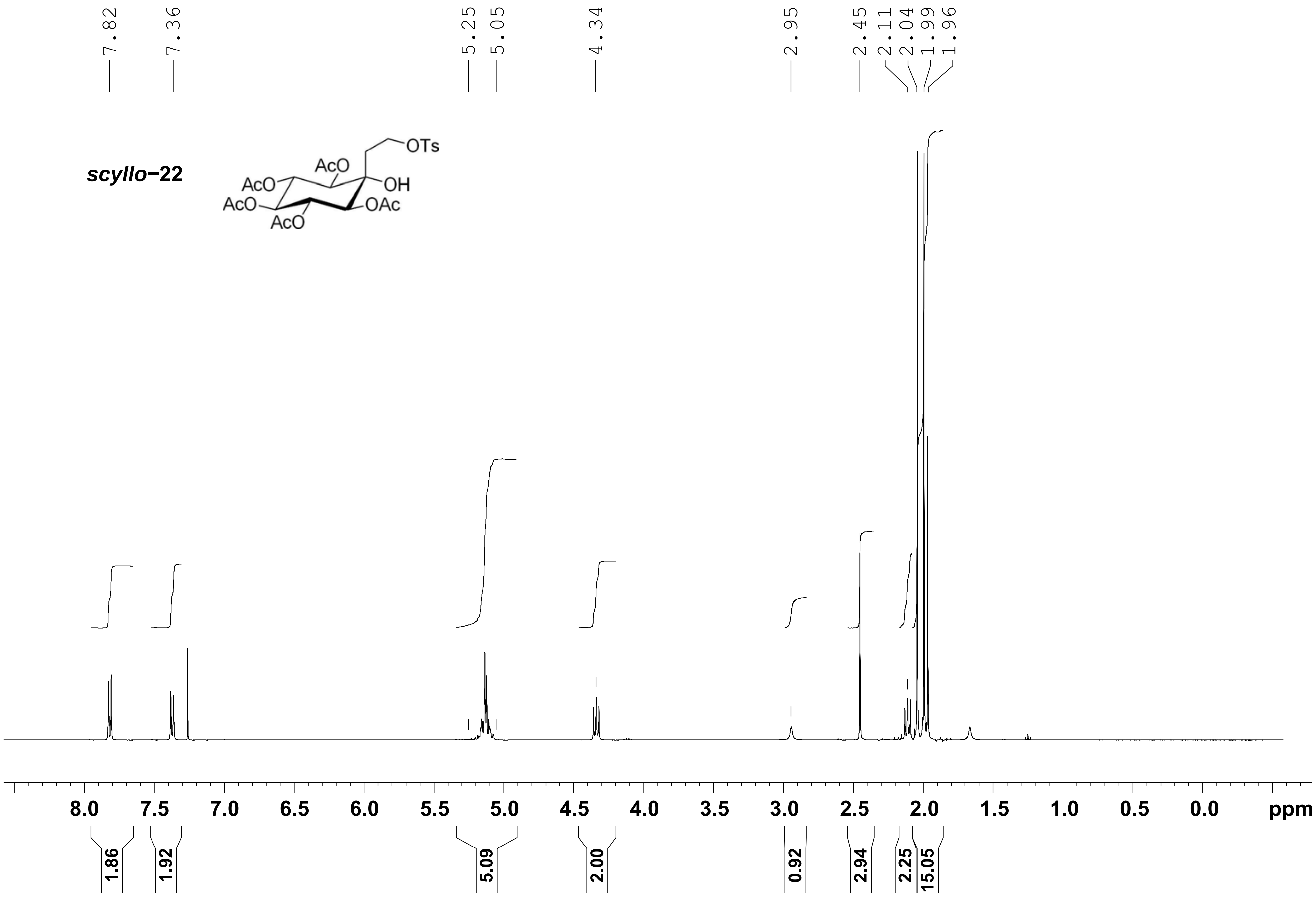

Figure 68: ^{1}H NMR spectrum

scyllo−**22**

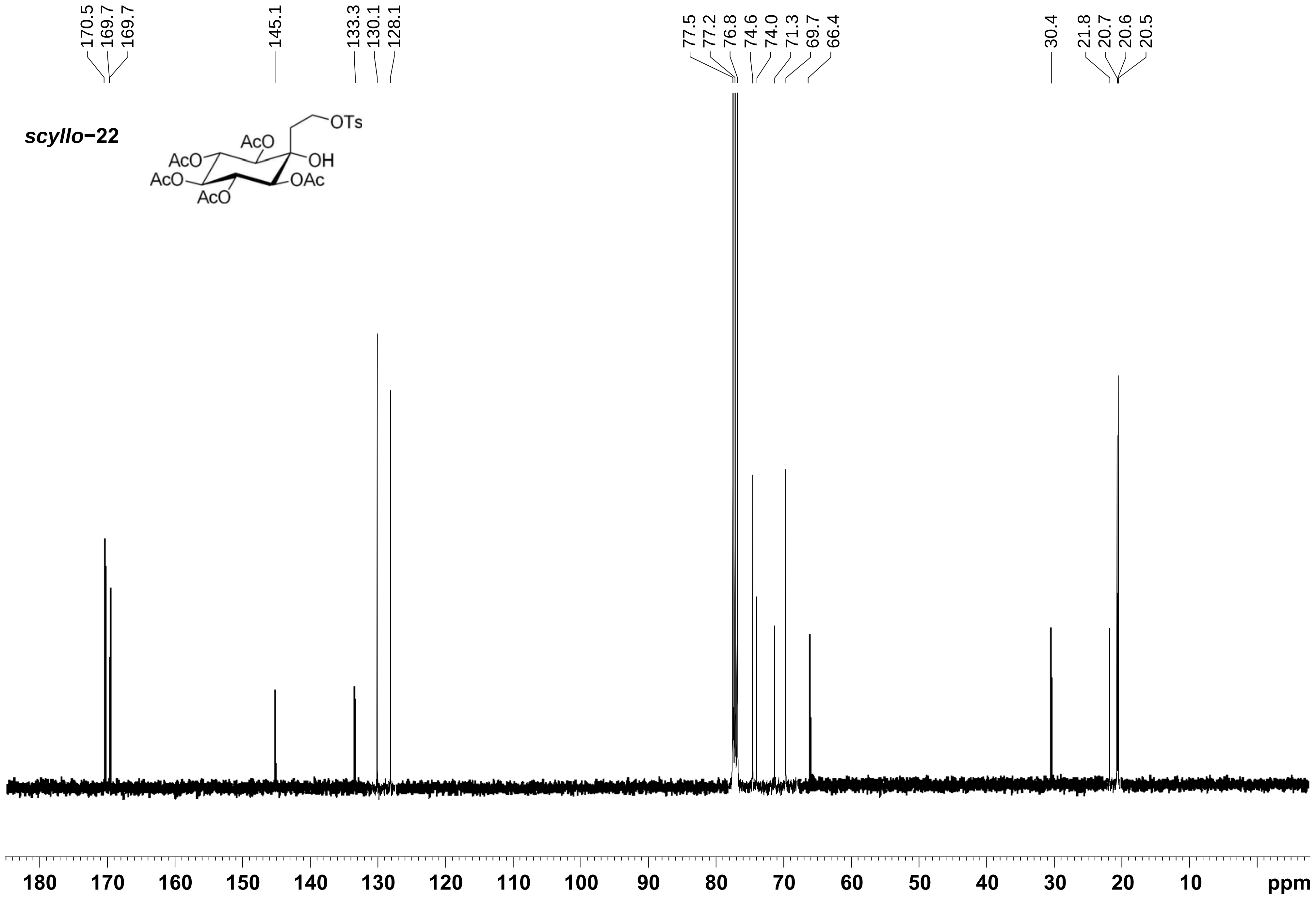

Figure 69: ^{13}C NMR spectrum

myo−**23**

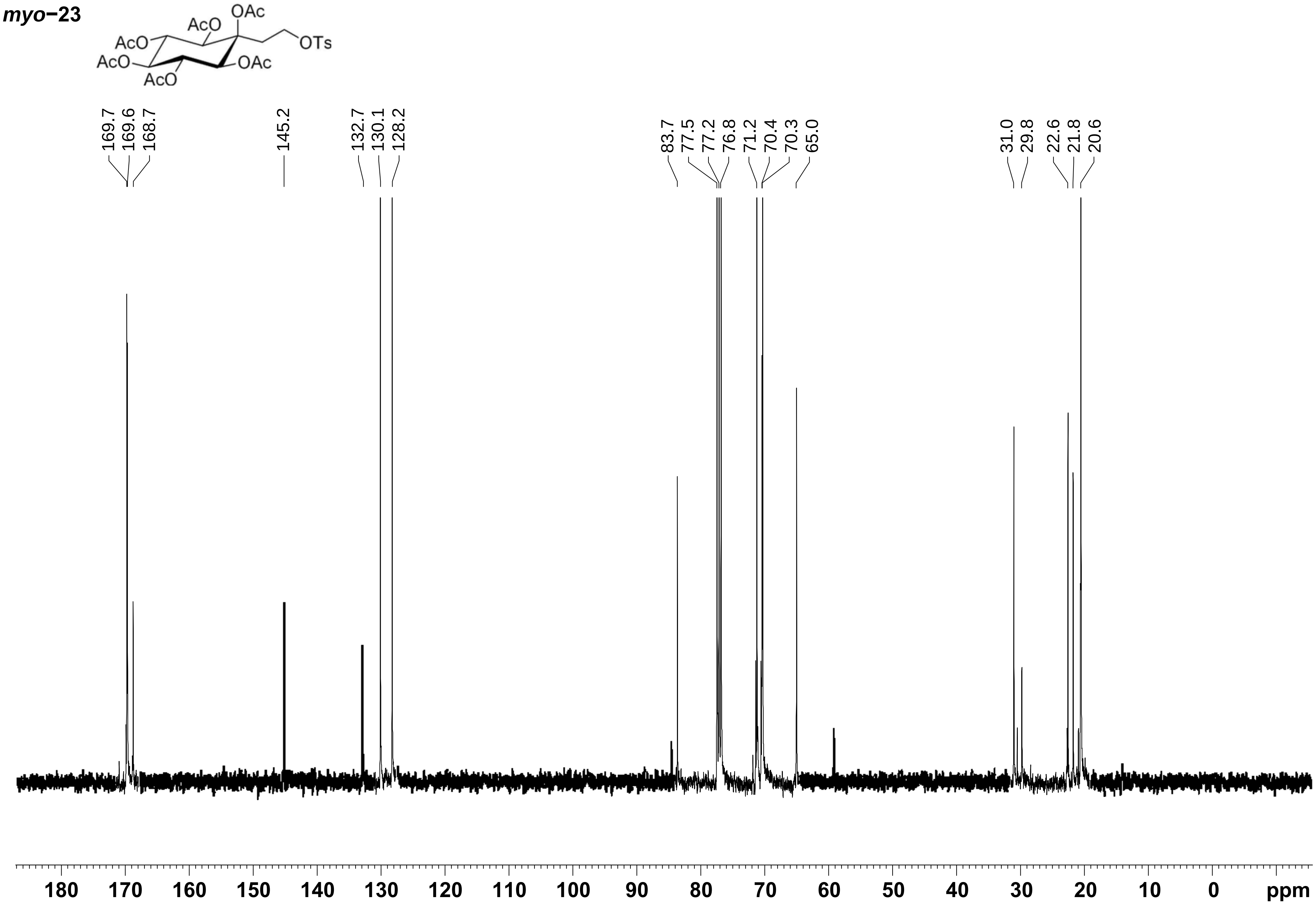

Figure 70: ^{13}C NMR spectrum

myo-**23**

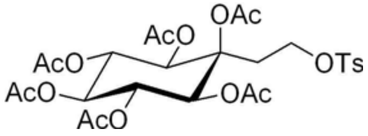

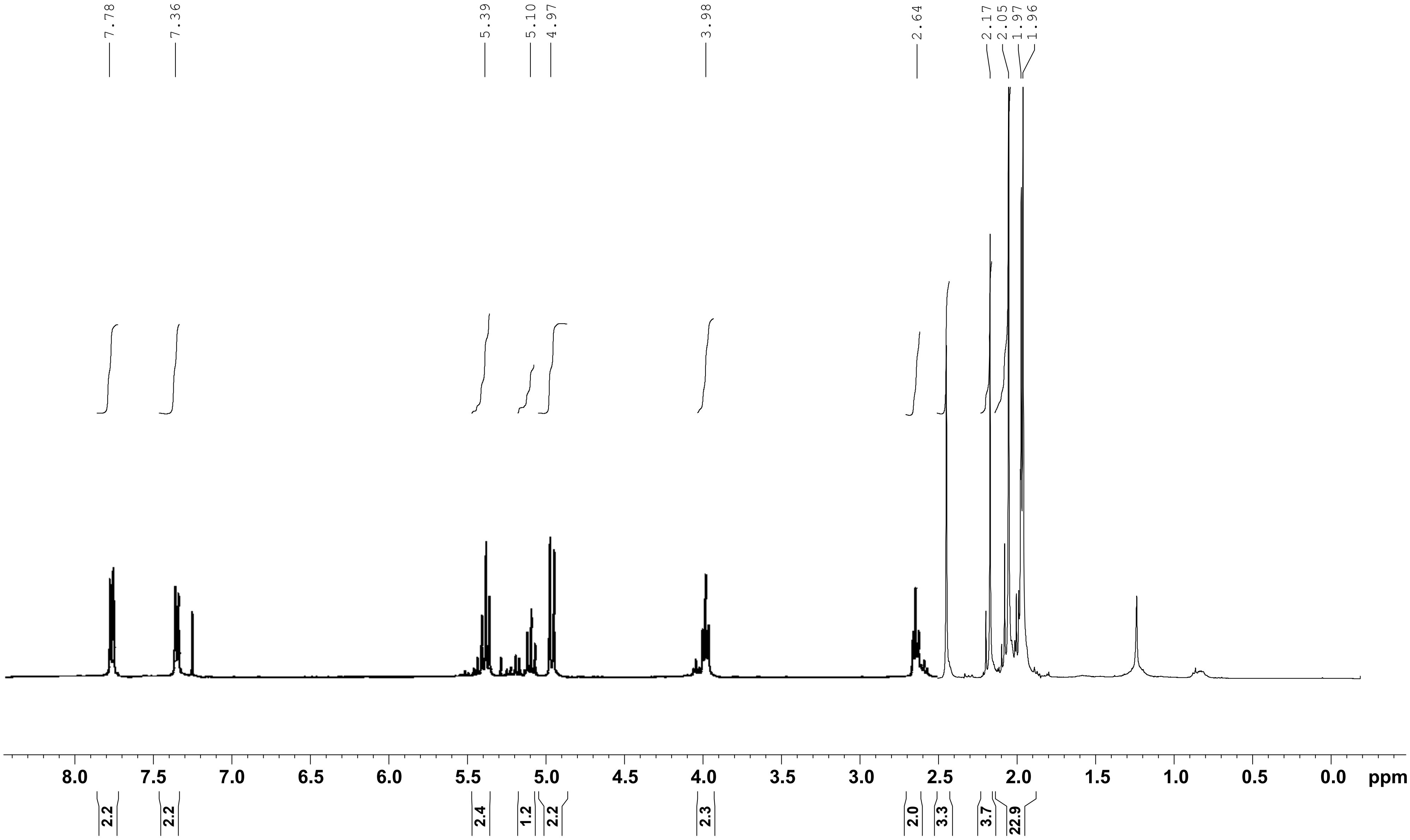

Figure 71: ^{1}H NMR spectrum

scyllo−**23**

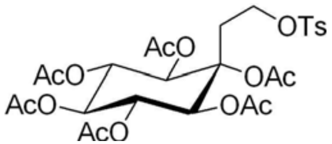

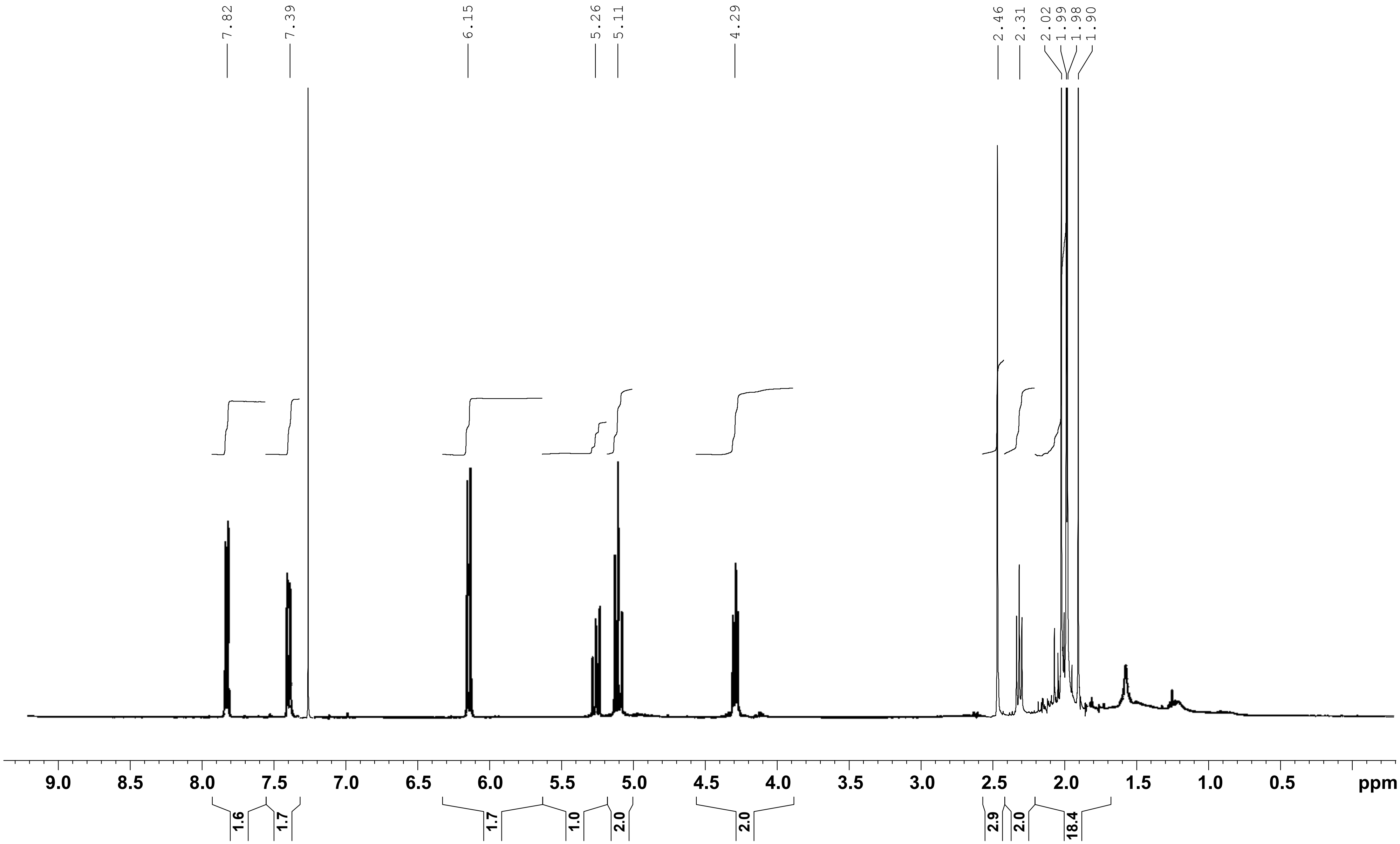

Figure 72: ^{1}H NMR spectrum

scyllo−**23**

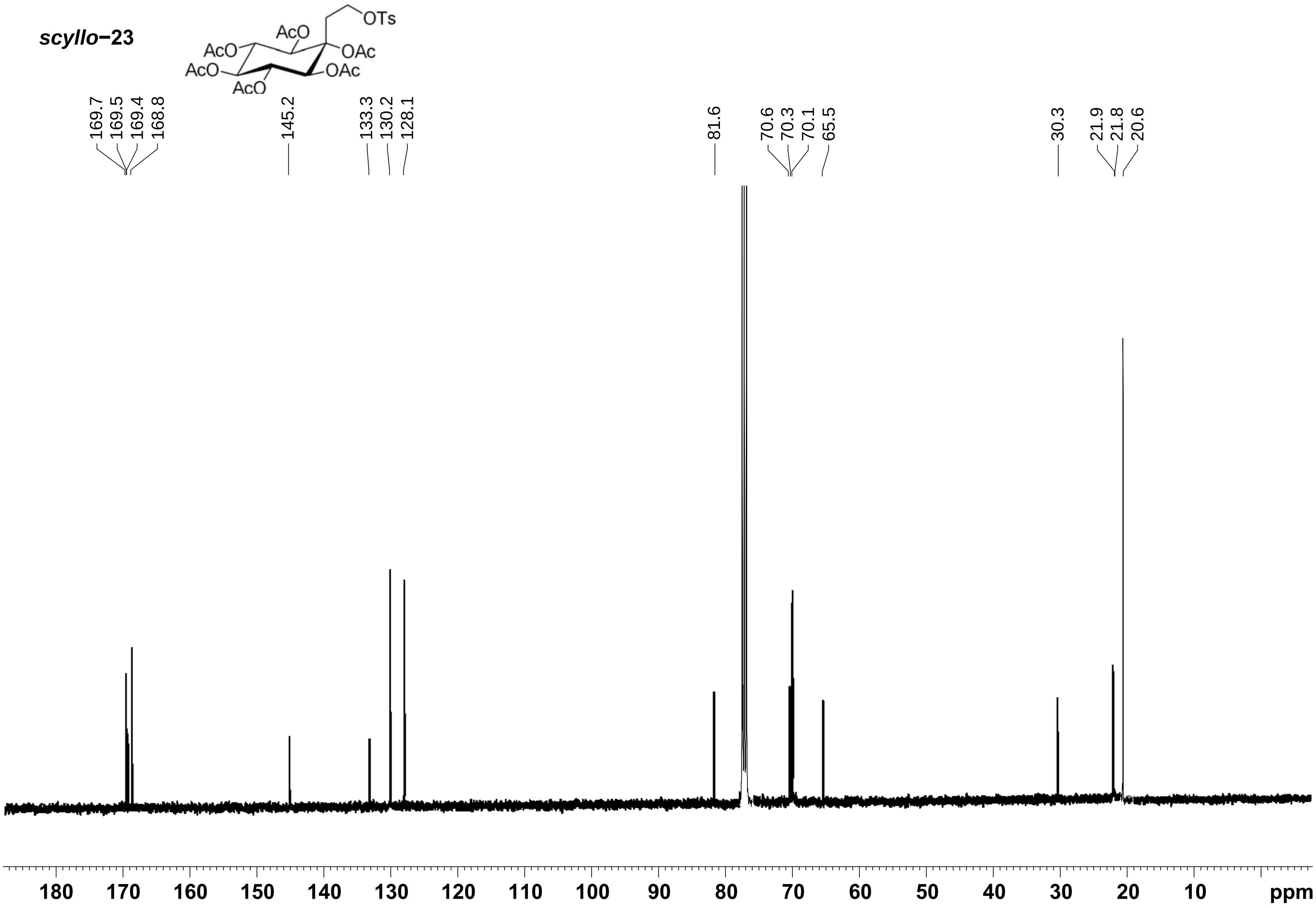

Figure 73: ^{13}C NMR spectrum

myo−**25**

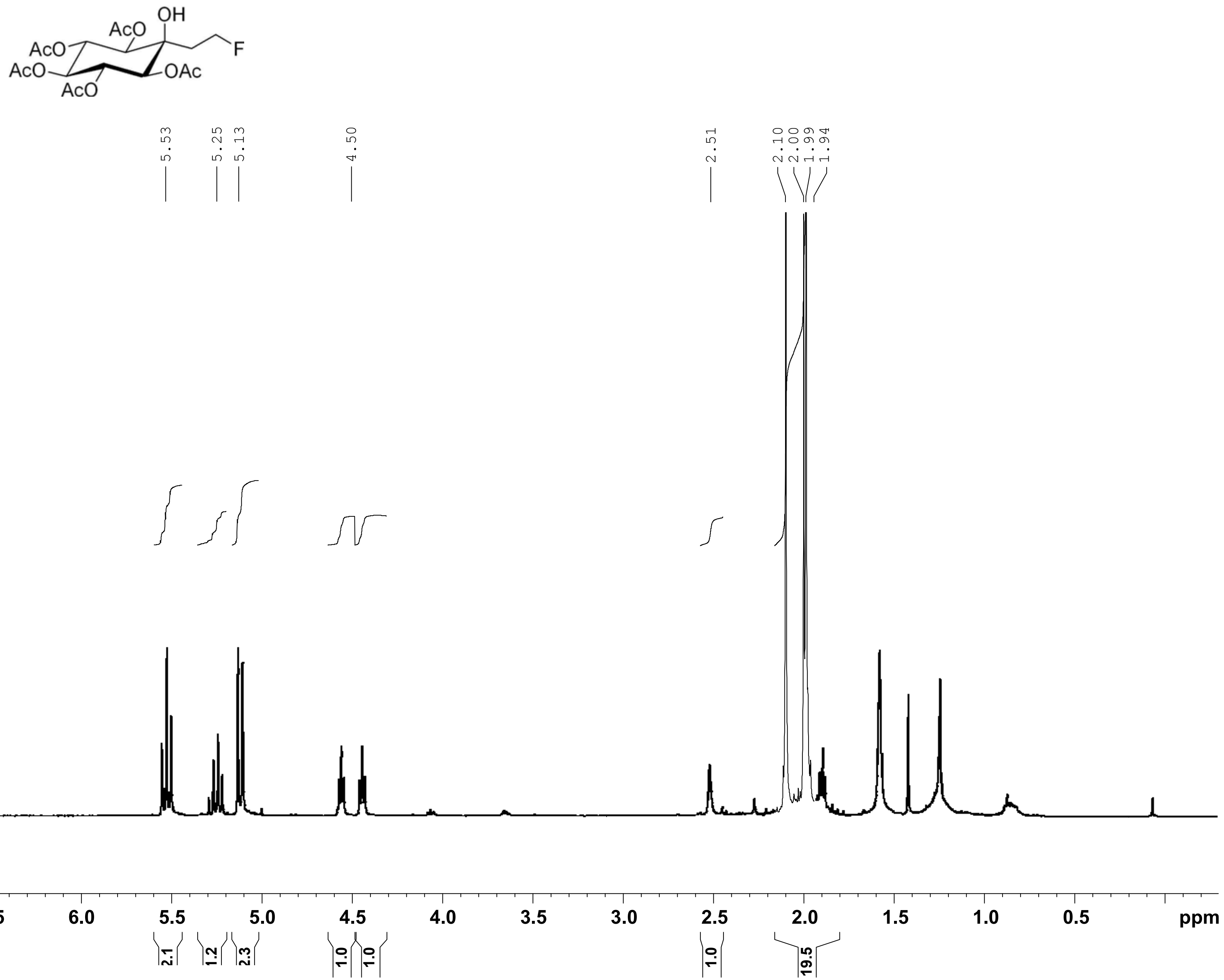

Figure 74: ^{1}H NMR spectrum

***myo*−25**

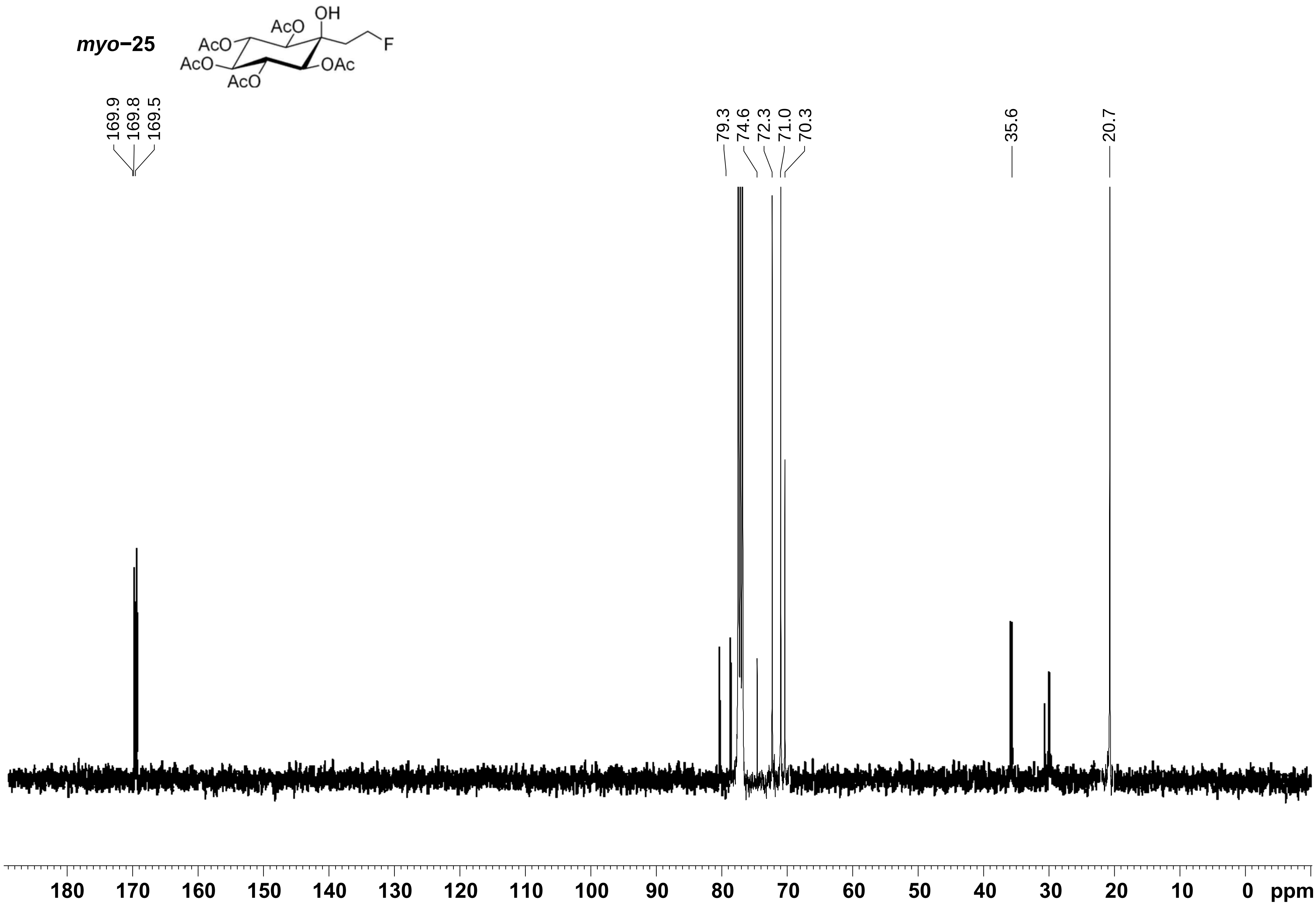

Figure 75: ^{13}C NMR spectrum

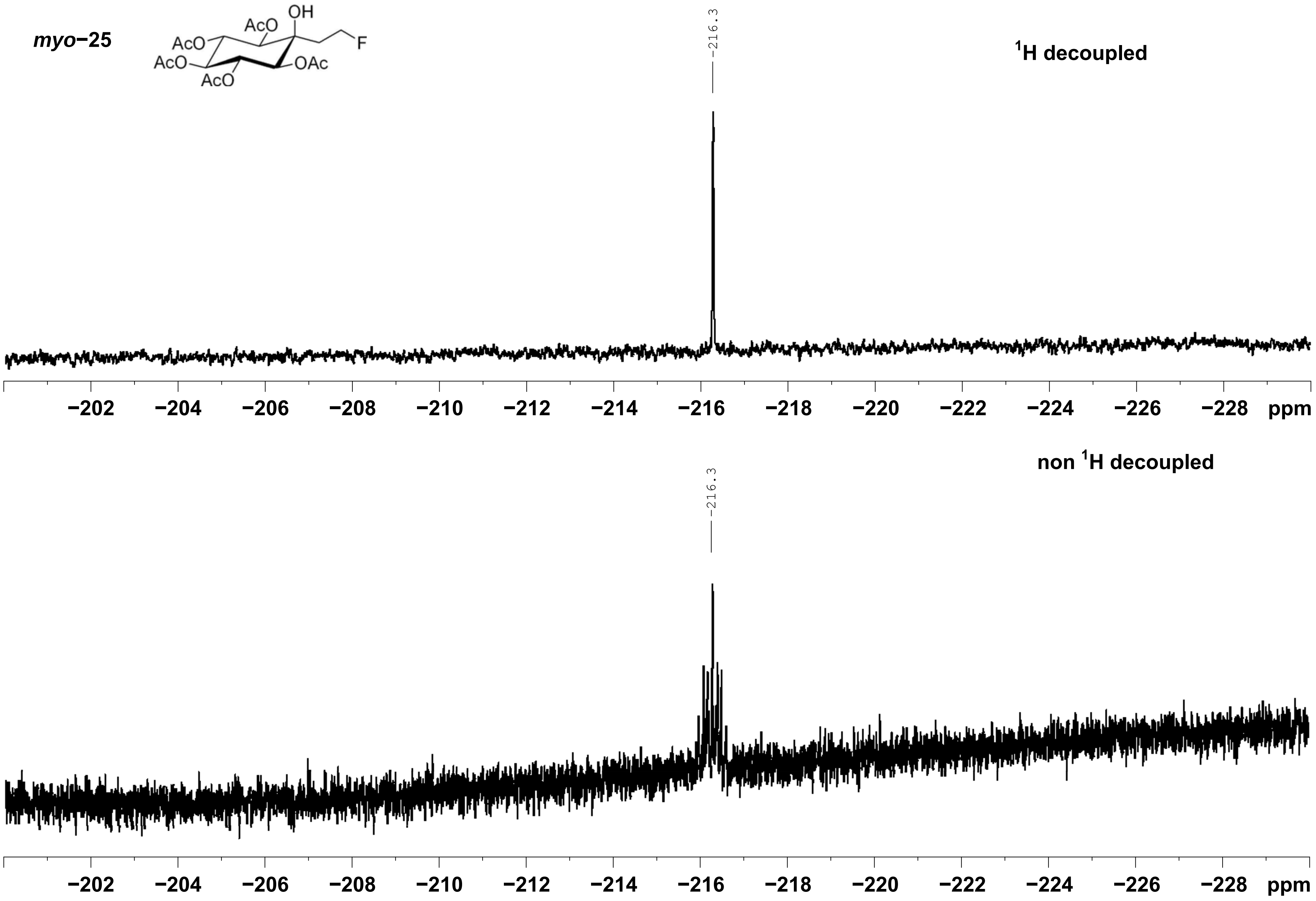

Figure 76: ^{19}F NMR spectrum

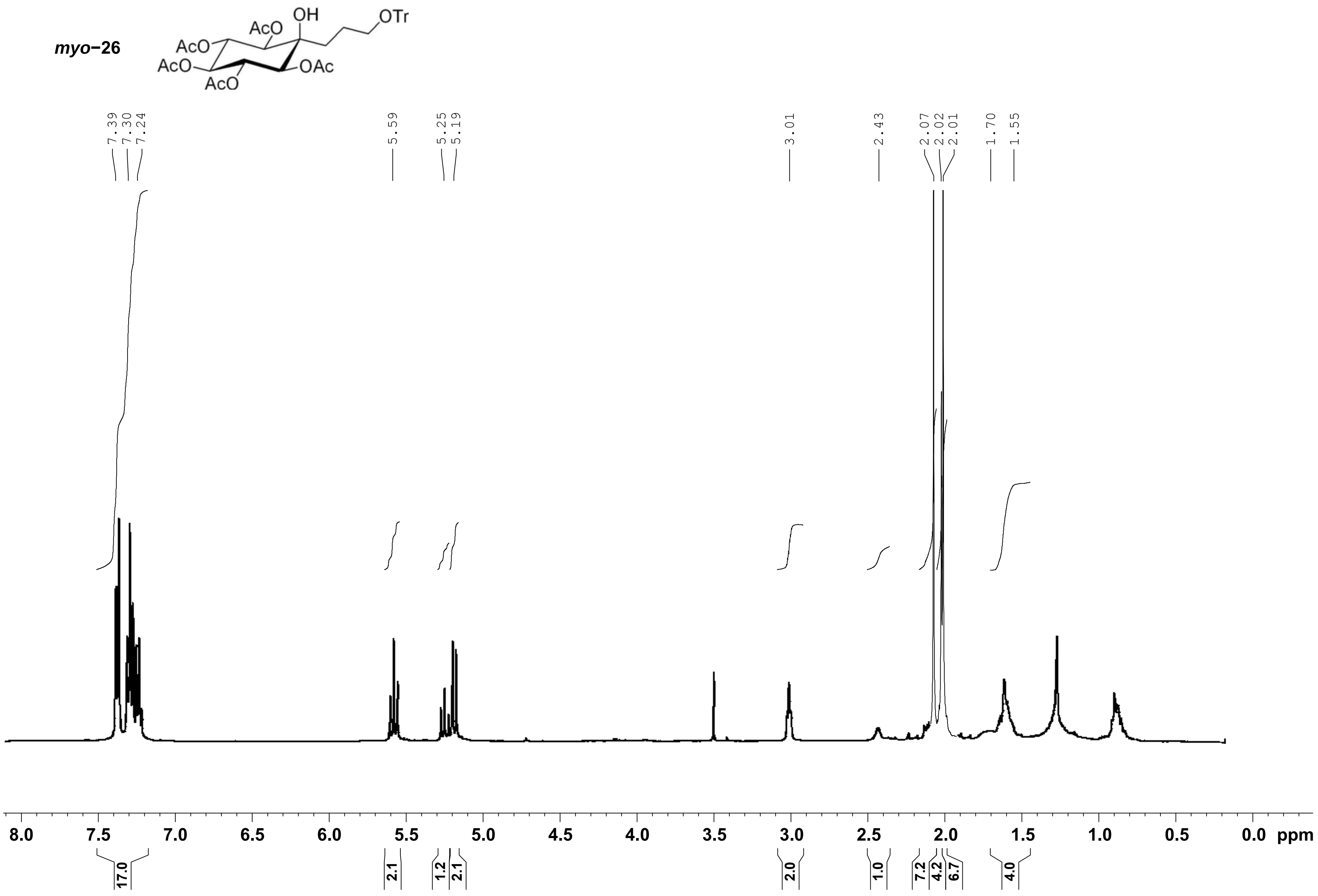

Figure 77: ^{1}H NMR spectrum

myo−**26**

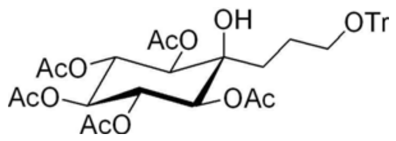

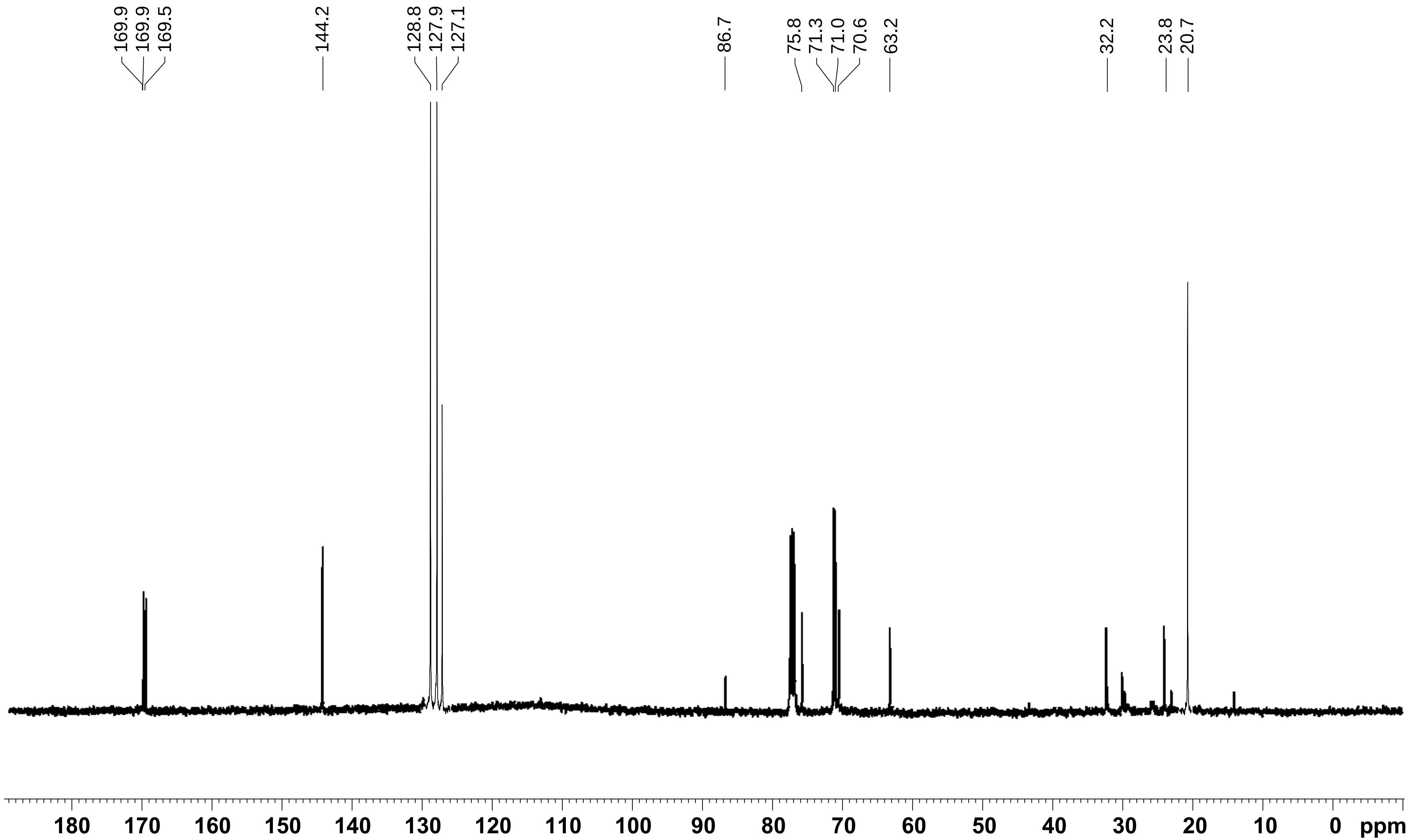

Figure 78: ^{13}C NMR spectrum

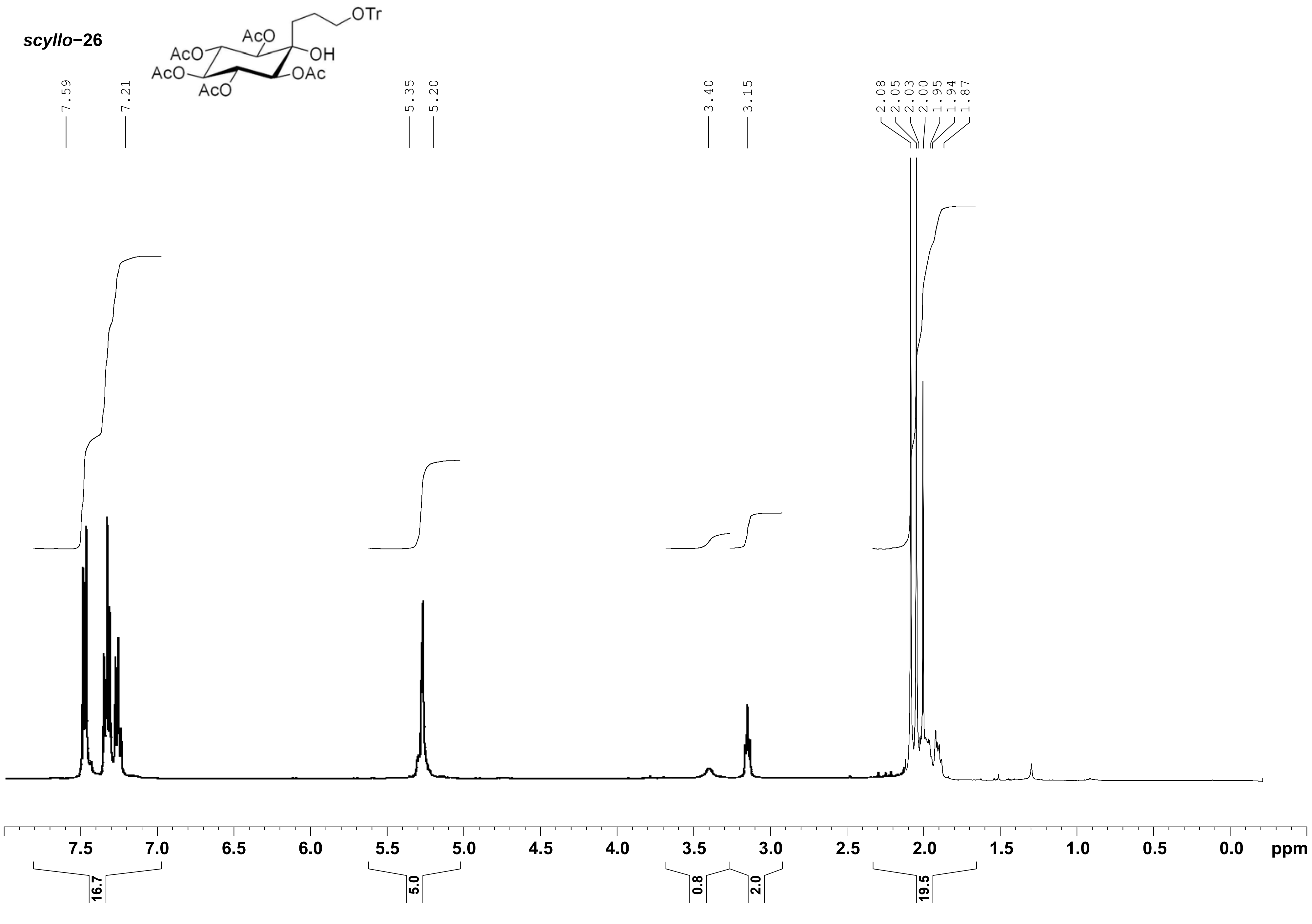

Figure 79: ^{1}H NMR spectrum

***scyllo*−26**

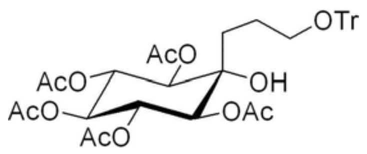

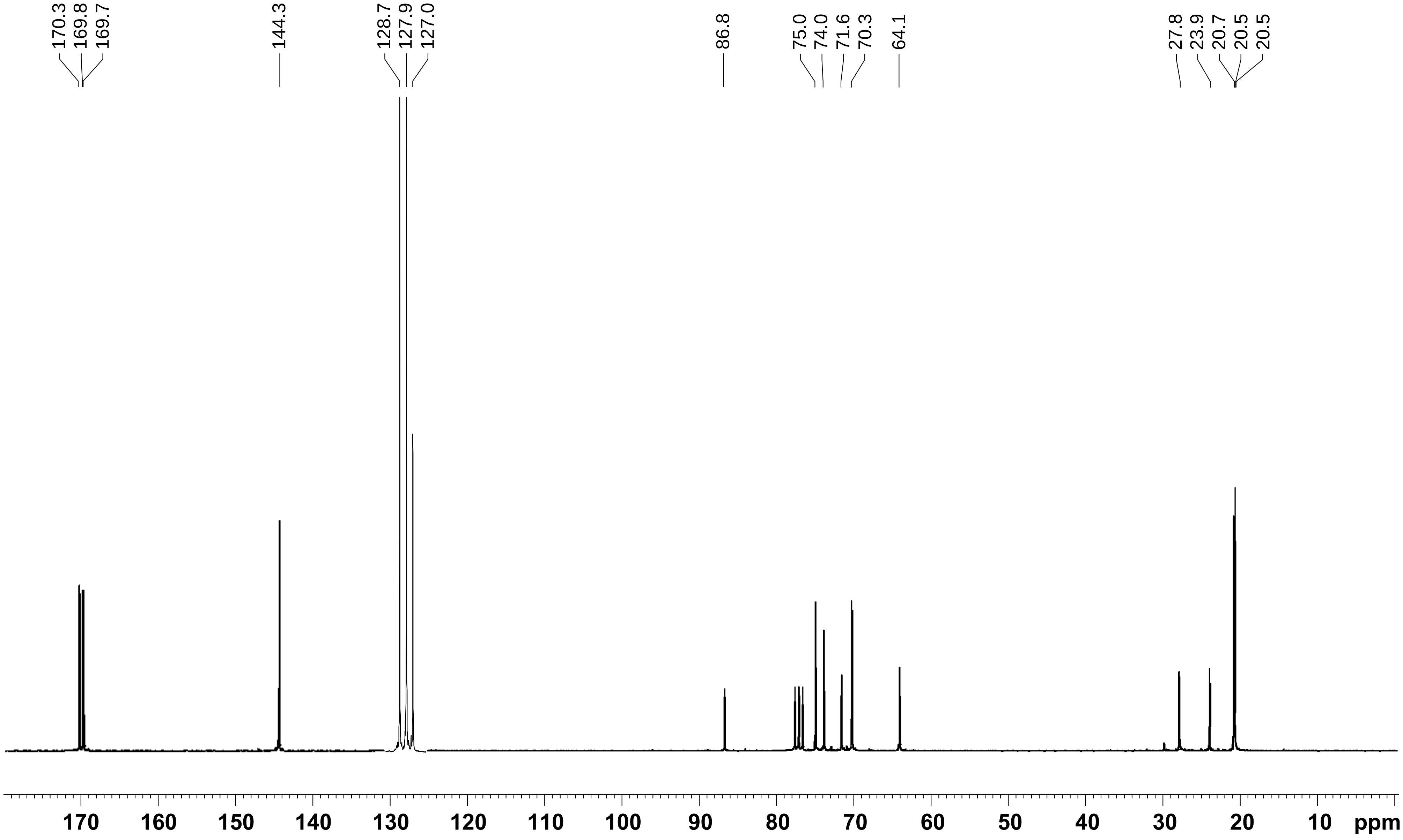

Figure 80: ^{13}C NMR spectrum

myo−**27**

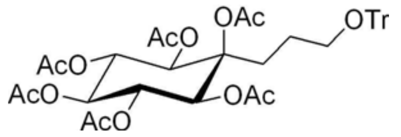

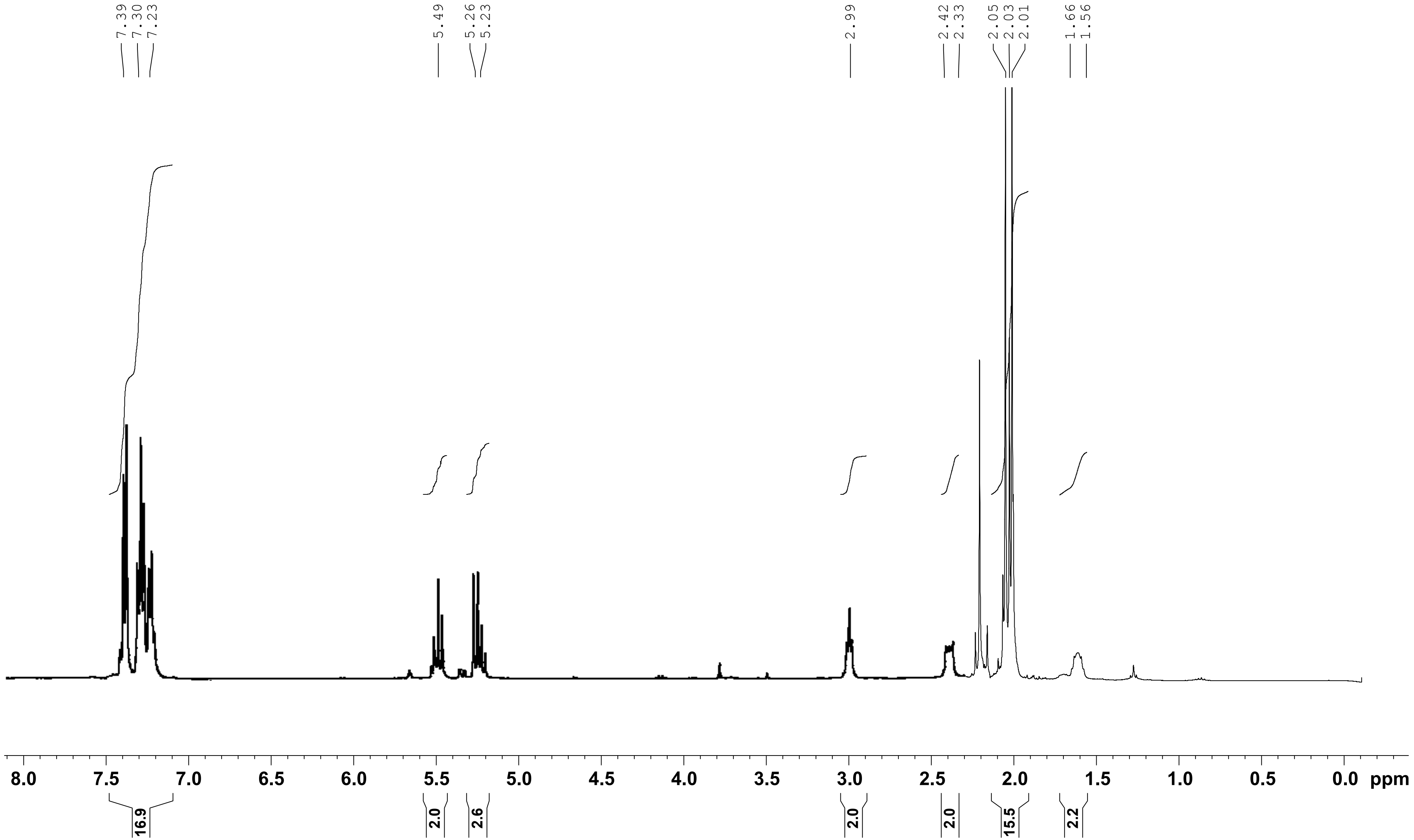

Figure 81: ^{1}H NMR spectrum

myo−27

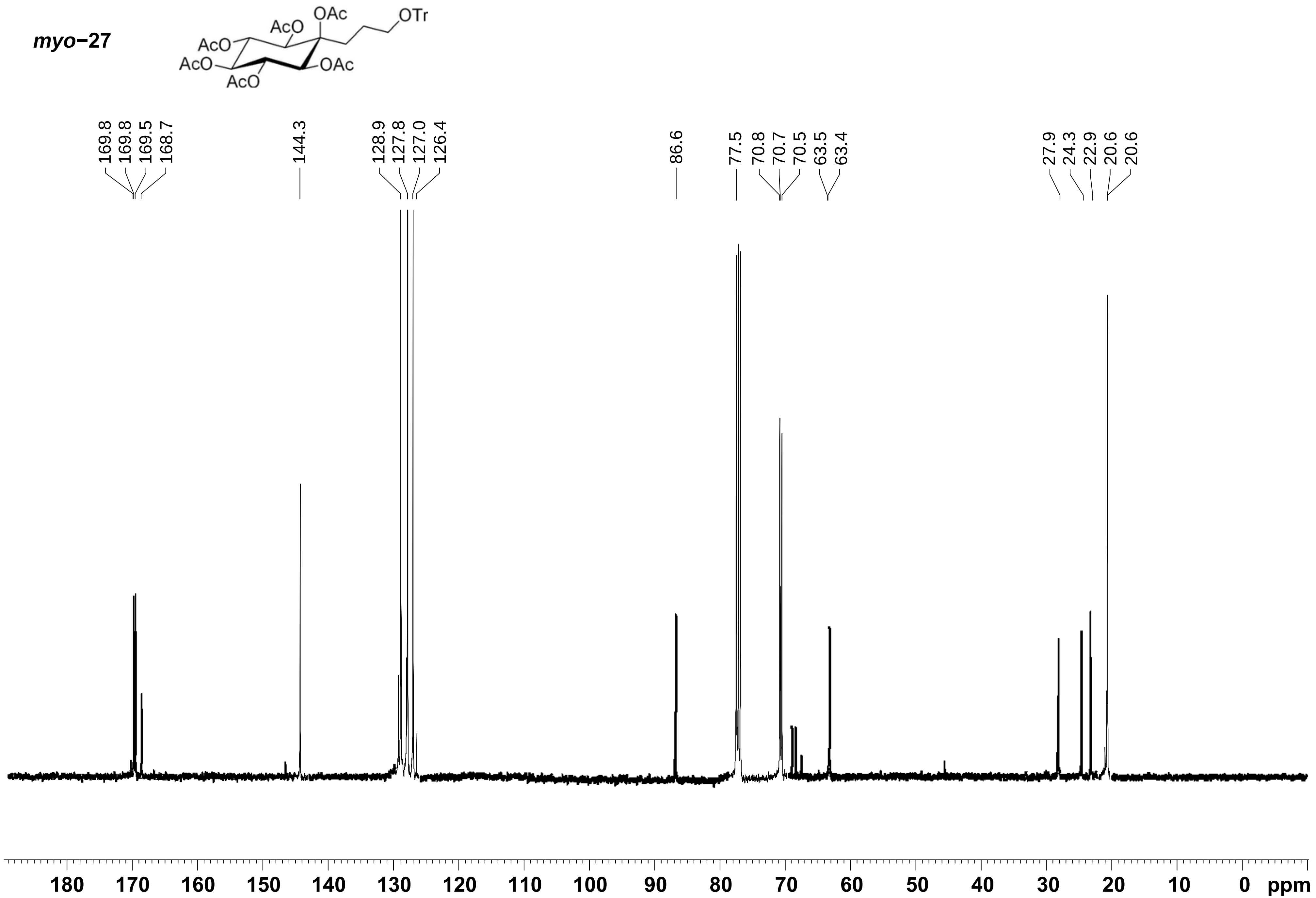

Figure 82: ^{13}C NMR spectrum

scyllo−**27**

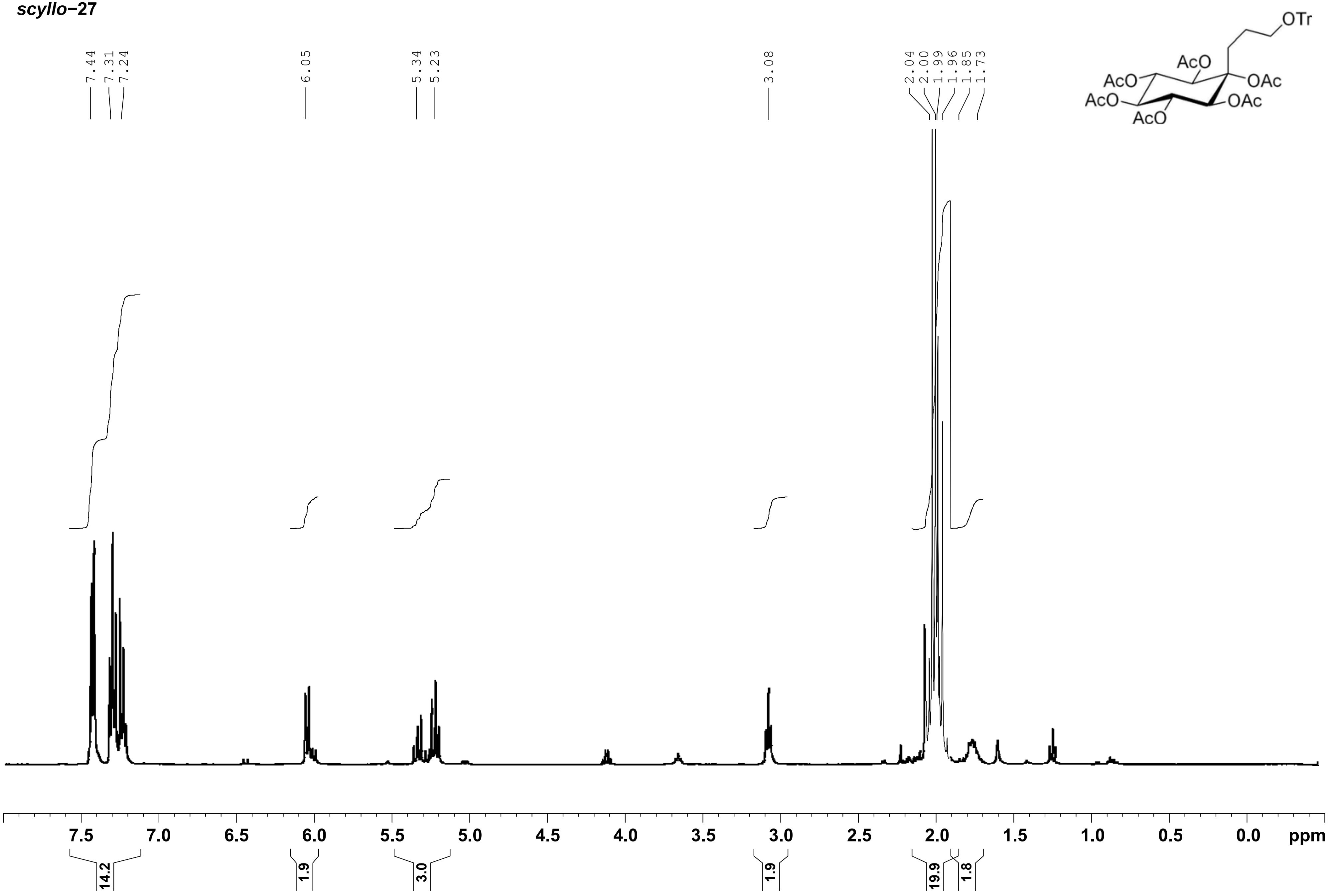

Figure 83: ^{1}H NMR spectrum

scyllo−**27**

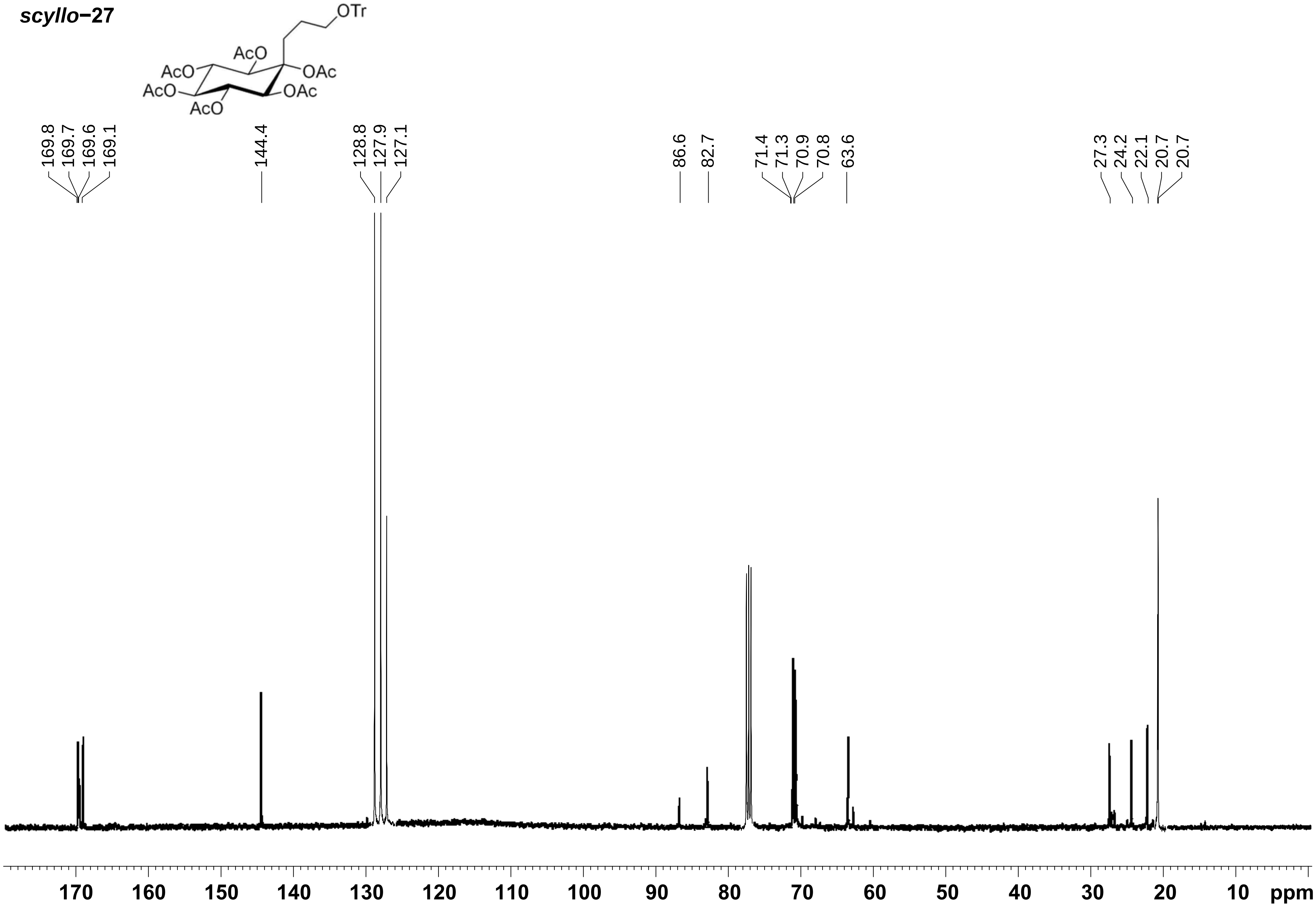

Figure 84: ^{13}C NMR spectrum

myo−28

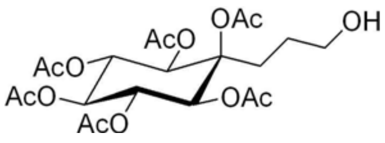

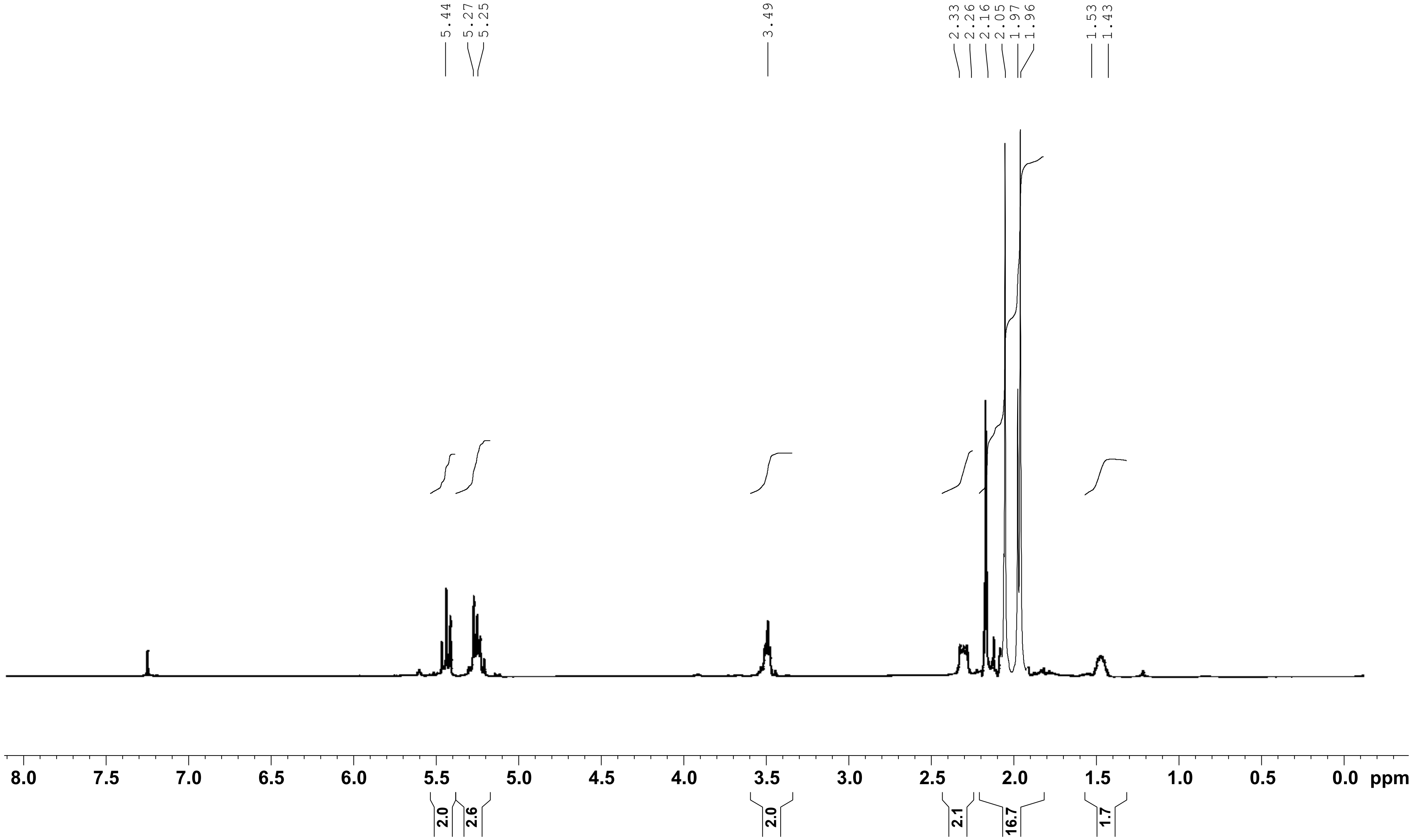

Figure 85: ^{1}H NMR spectrum

myo−**28**

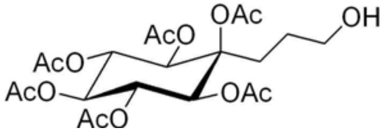

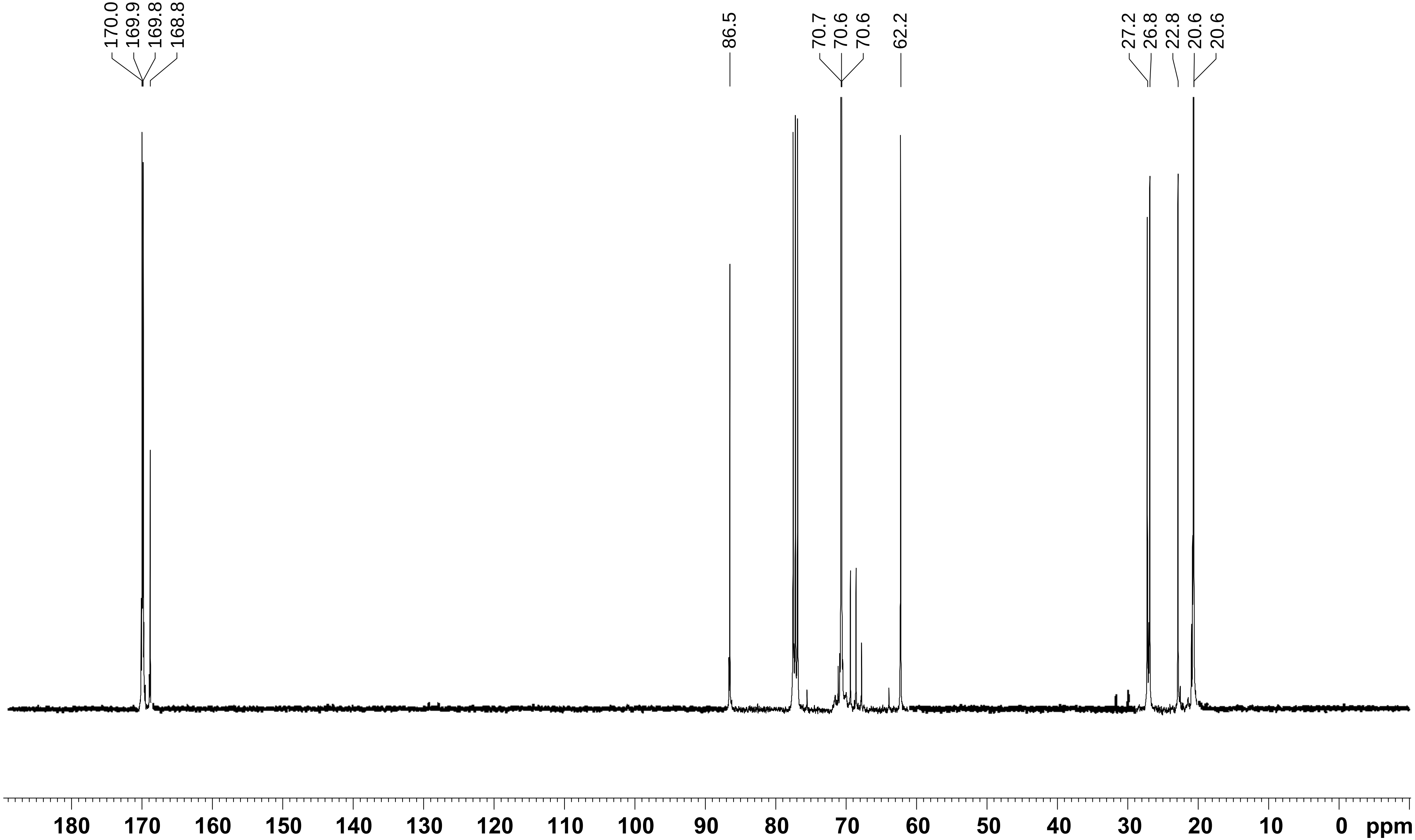

Figure 86: ^{13}C NMR spectrum

scyllo−**28**

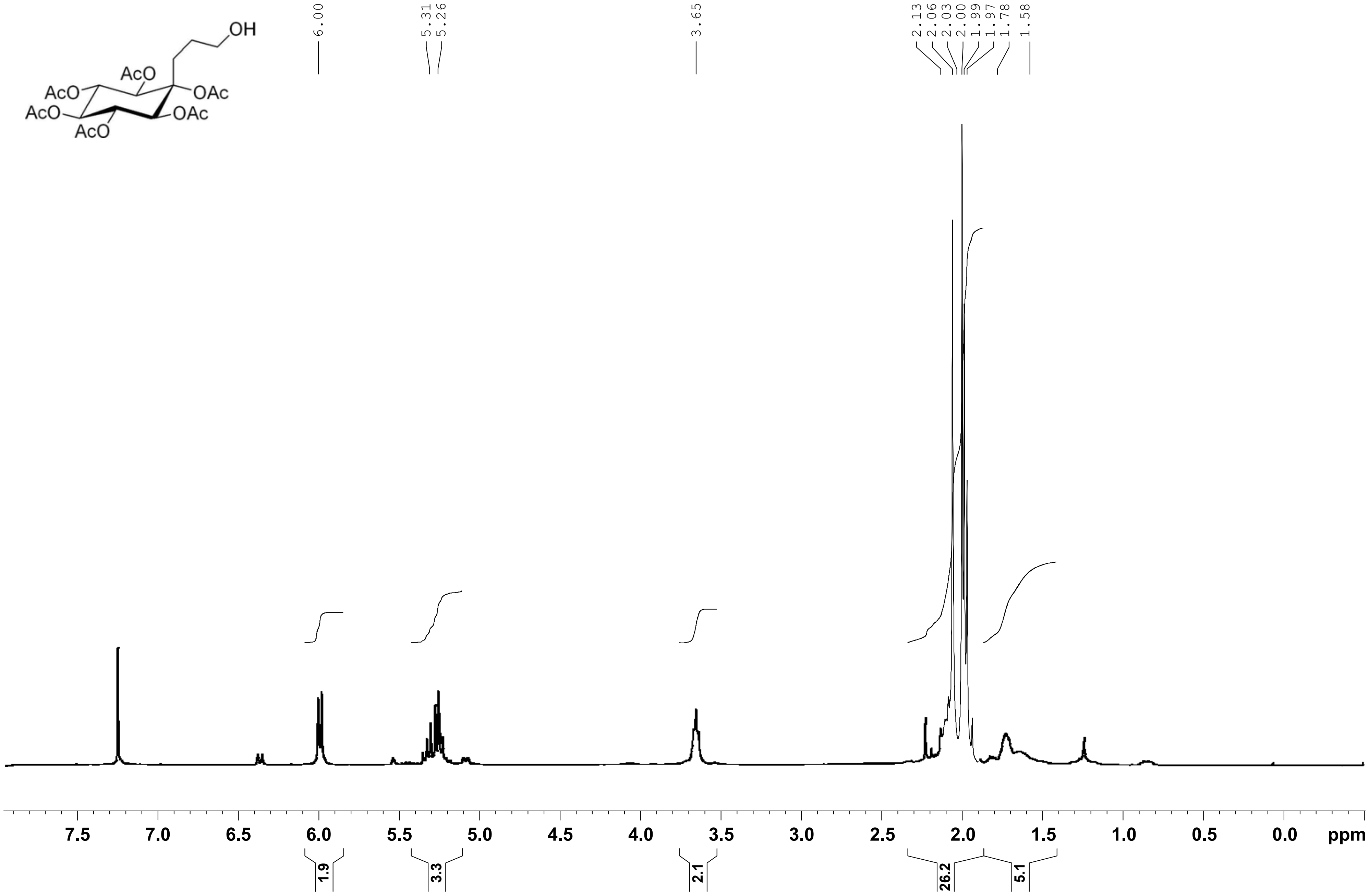

Figure 87: ^{1}H NMR spectrum

***scyllo*−28**

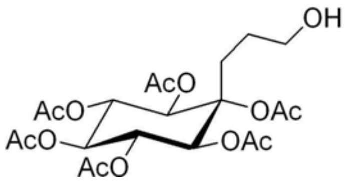

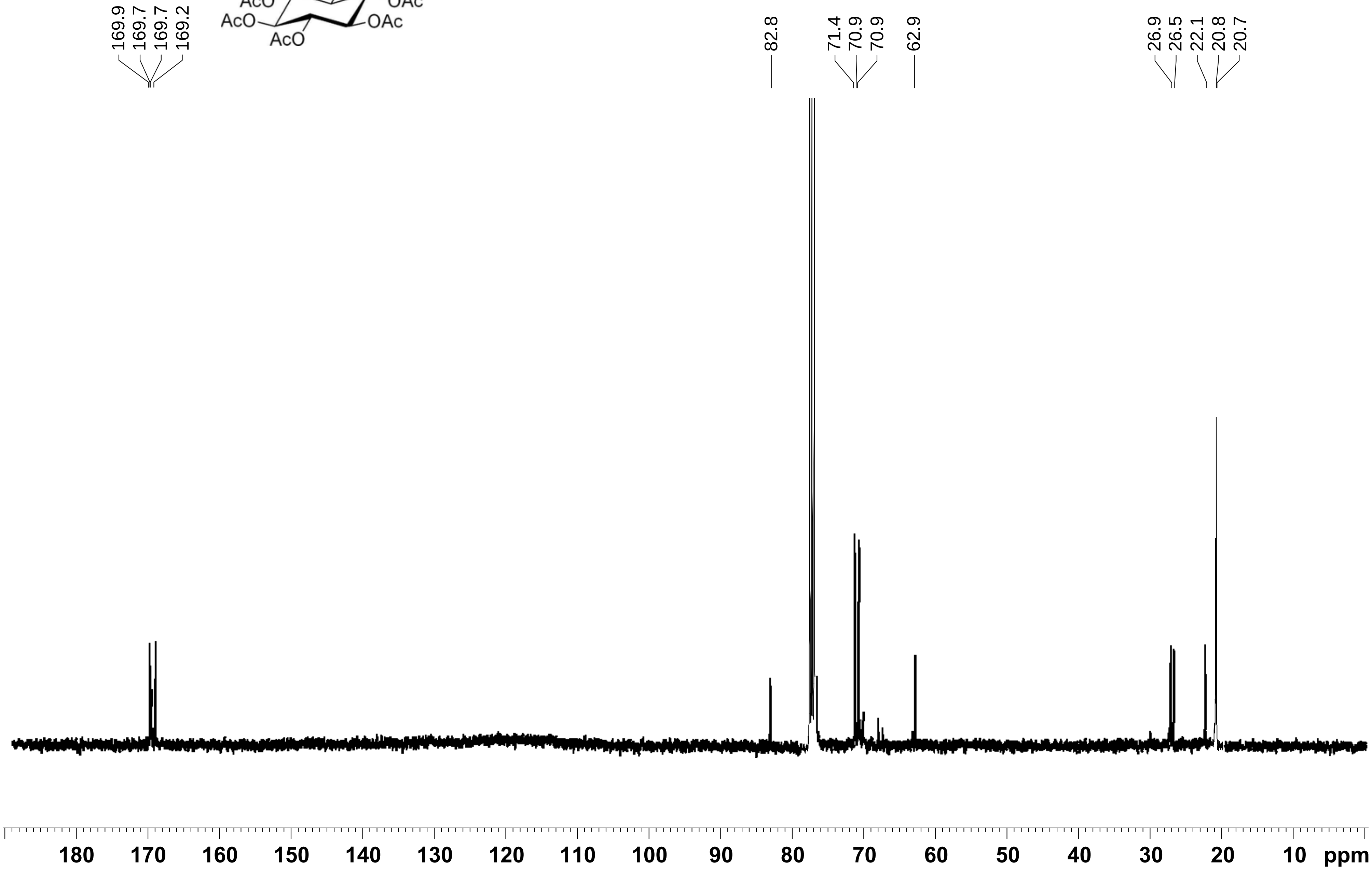

Figure 88: ^{13}C NMR spectrum

***myo*−29**

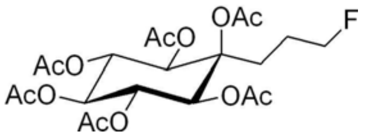

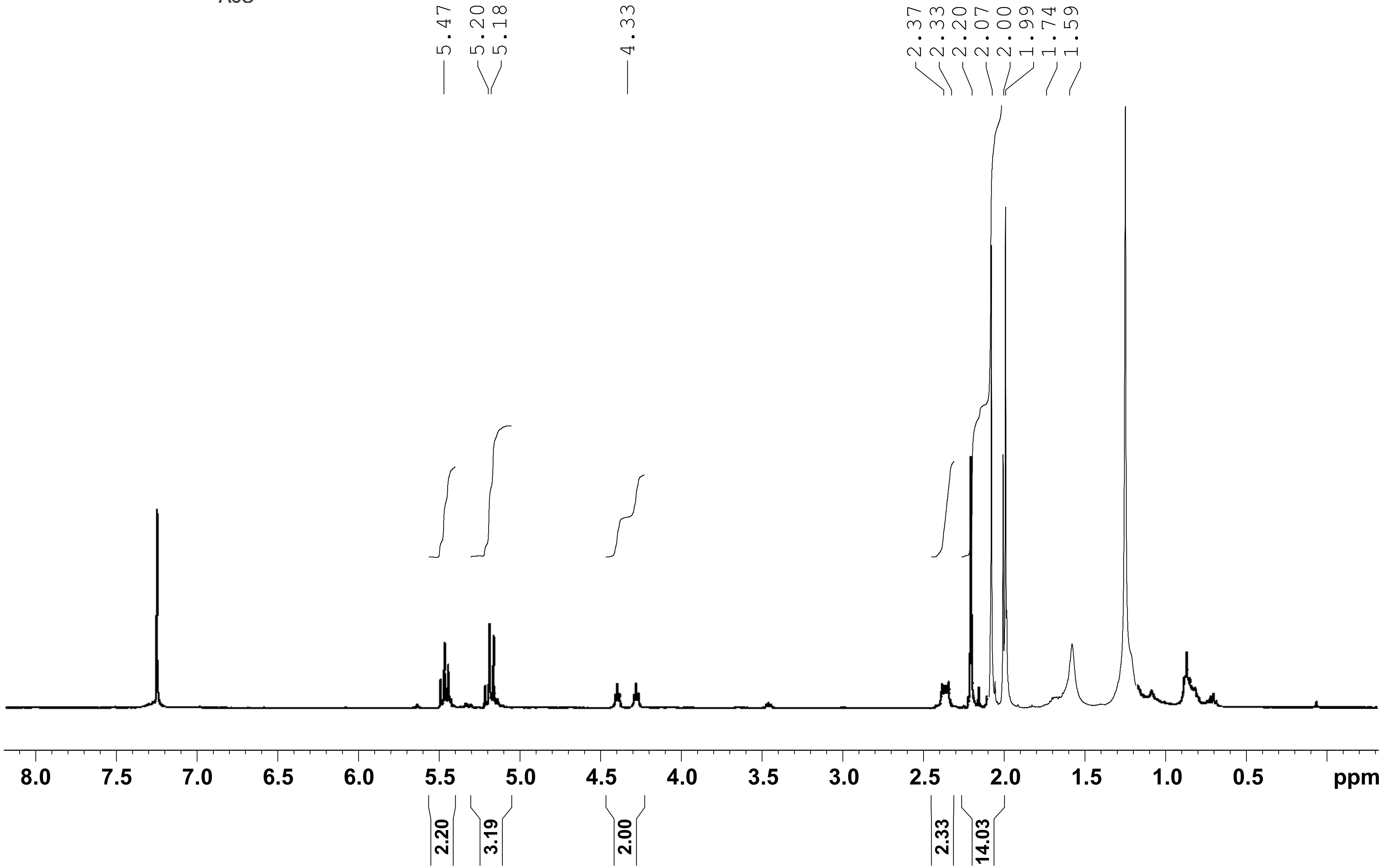

Figure 89: ^{1}H NMR spectrum

***myo*−29**

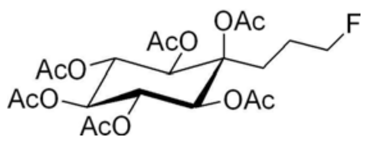

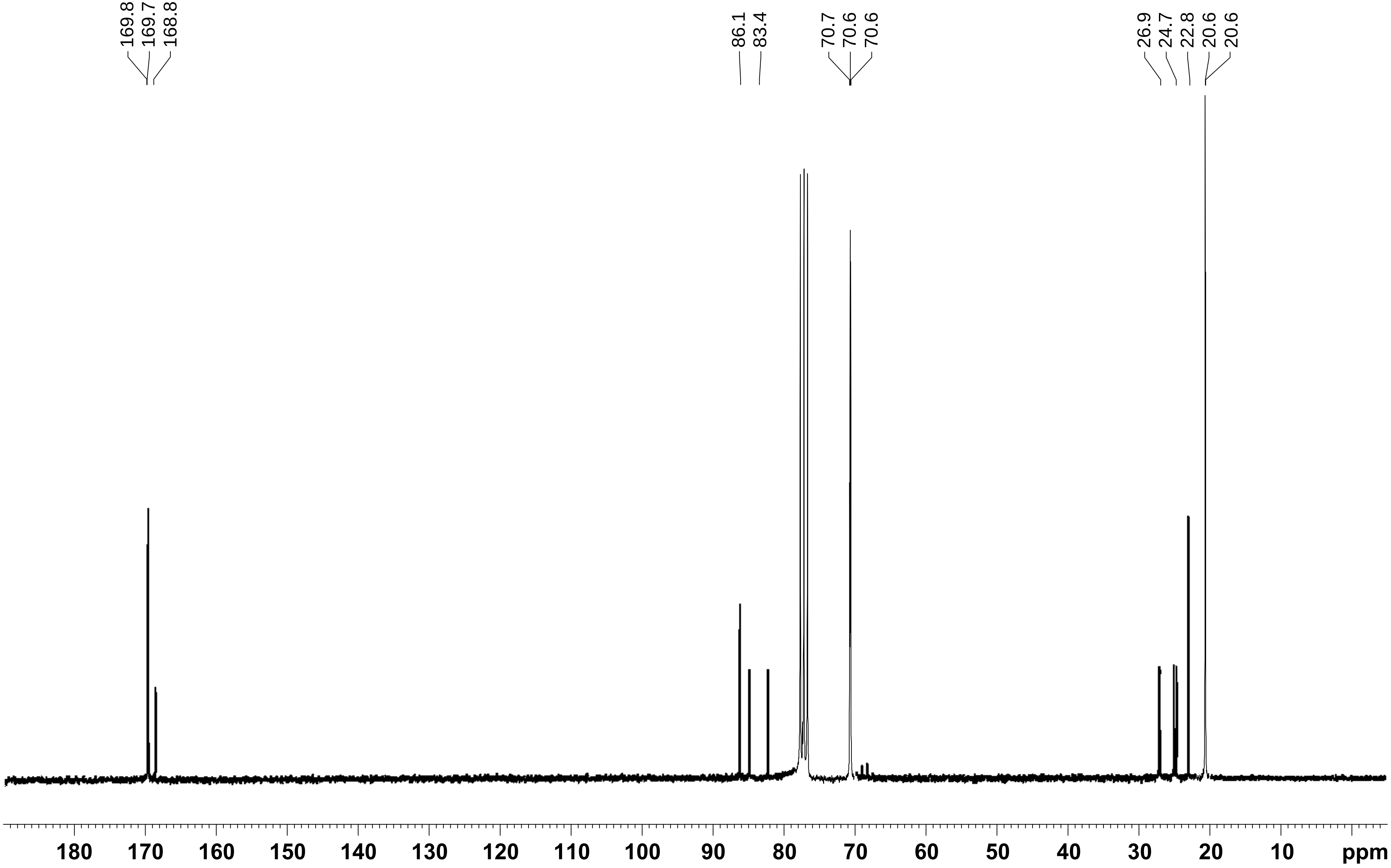

Figure 90: ^{13}C NMR spectrum

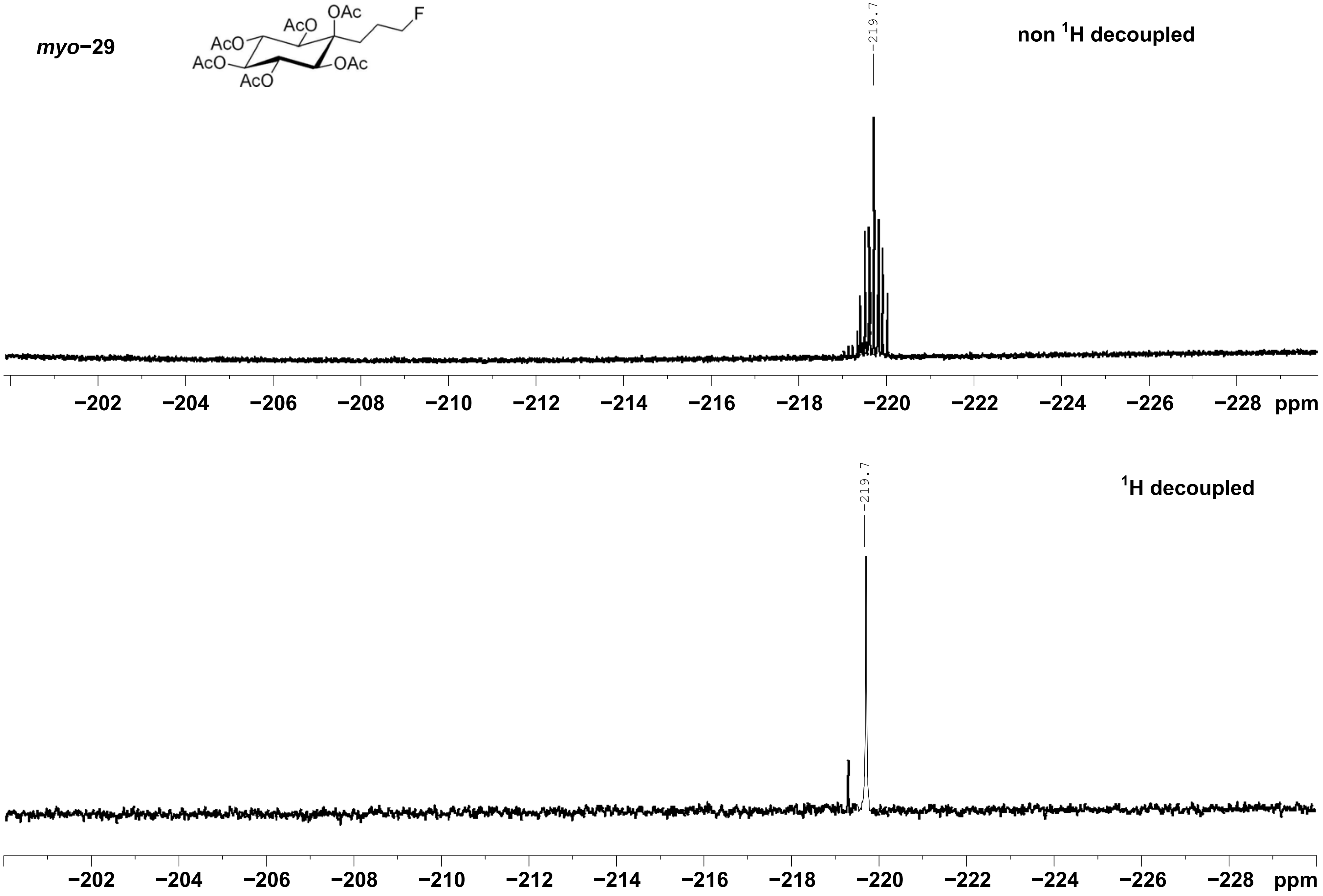

Figure 91: ^{19}**F NMR spectrum**

***scyllo*−29**

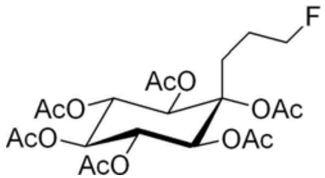

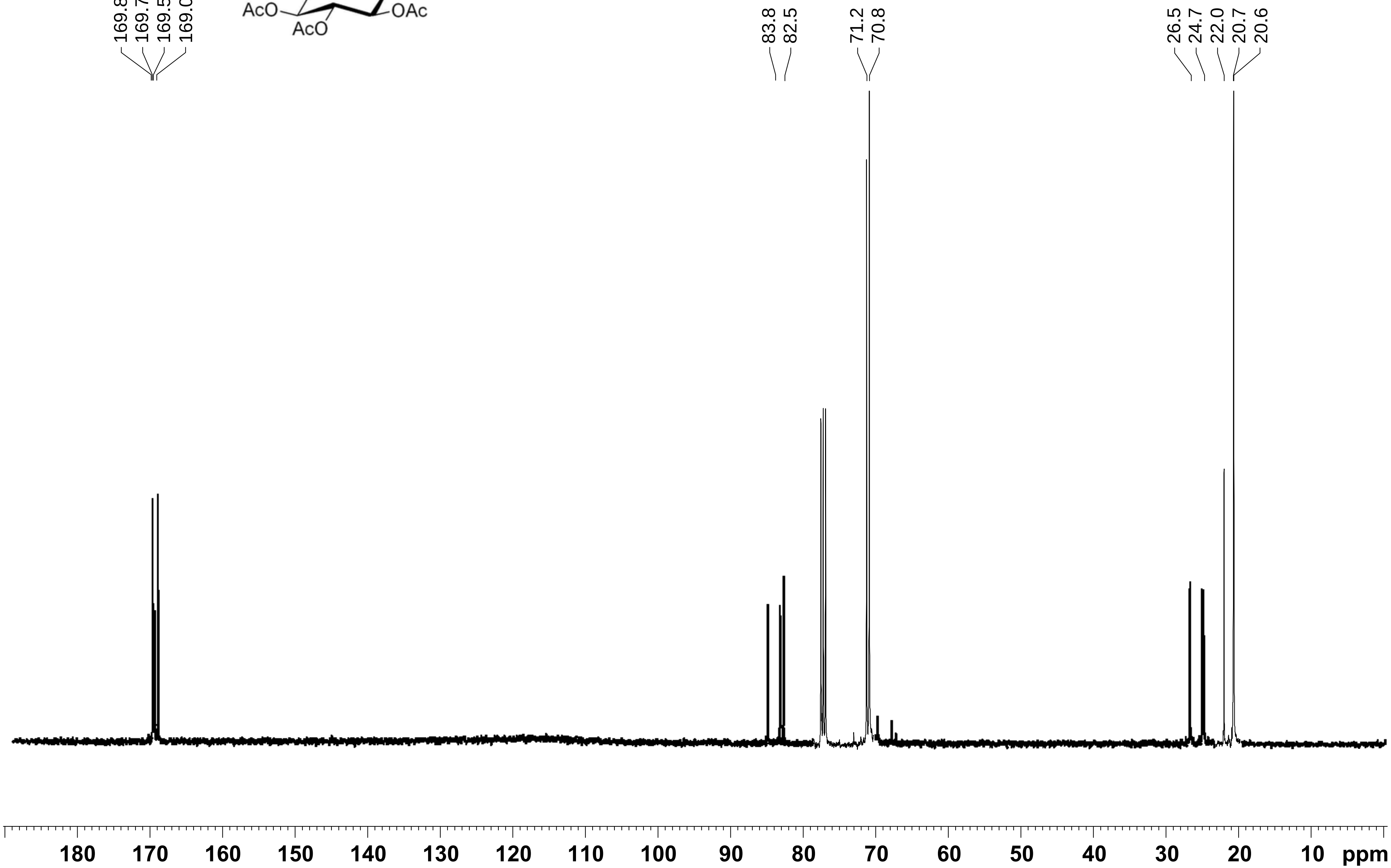

Figure 92: ^{13}C NMR spectrum

scyllo−**29**

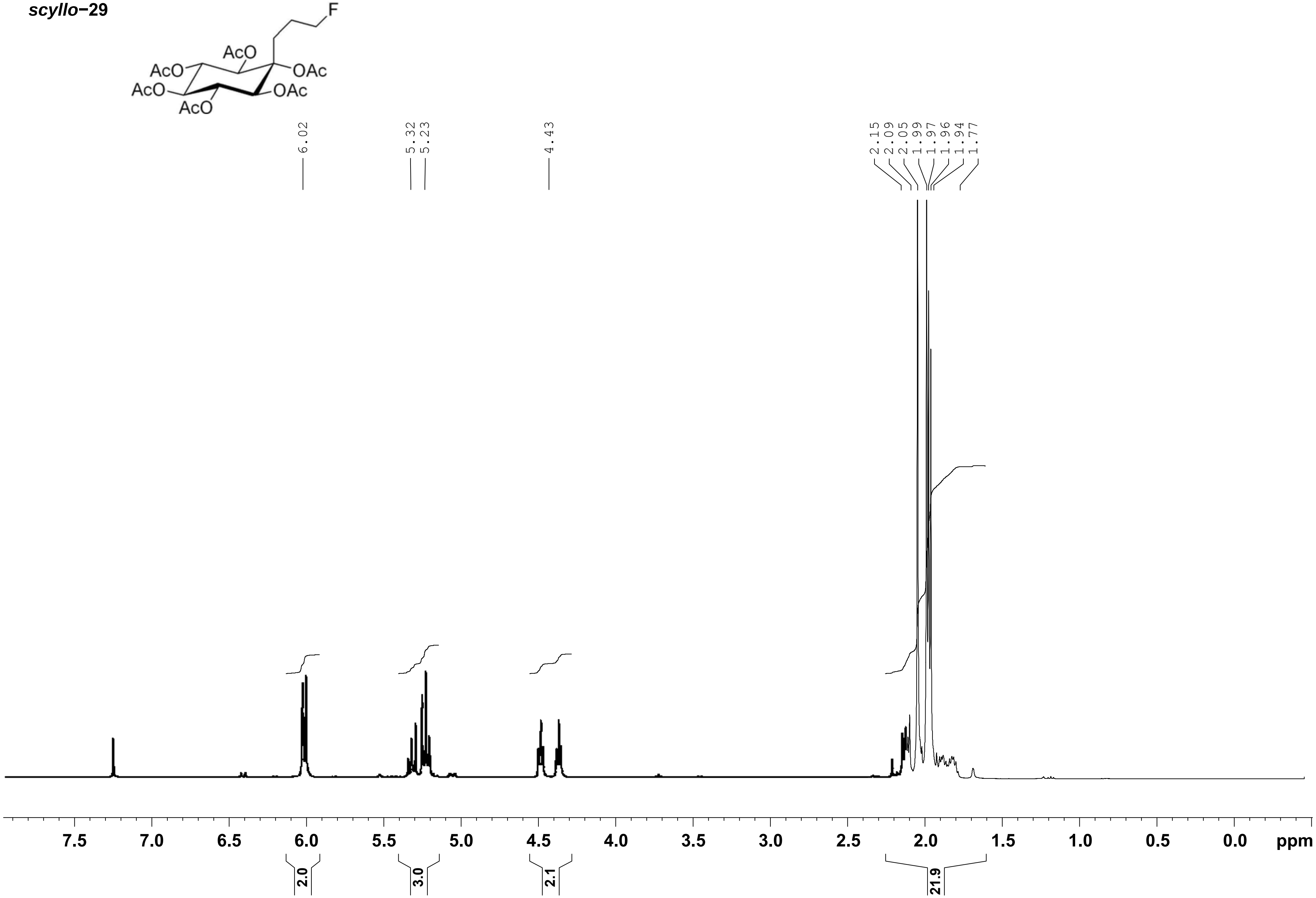

Figure 93: ^{1}H NMR spectrum

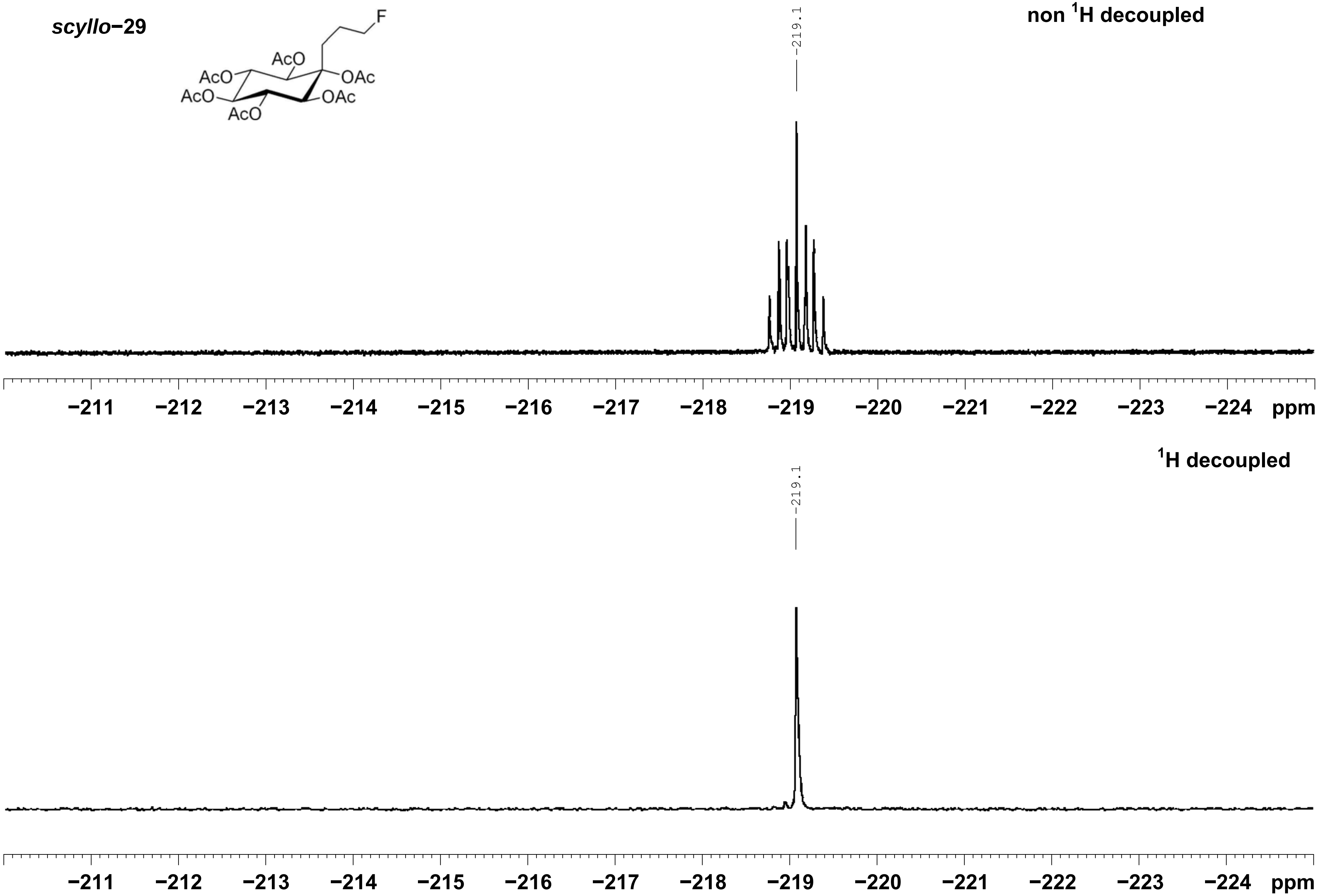

Figure 94: ^{19}F NMR spectrum
